# The Elusive Ternary Intermediates of Chiral Phosphoric Acids in Ion Pair Catalysis—Structures, Conformations, and Aggregation

**DOI:** 10.1021/jacs.4c14096

**Published:** 2025-01-09

**Authors:** Maximilian Franta, Aryaman Pattanaik, Wagner Silva, Kumar Motiram-Corral, Julia Rehbein, Ruth M. Gschwind

**Affiliations:** Institute of Organic Chemistry, University Regensburg, Universitätsstr. 31, 93053 Regensburg, Germany

## Abstract

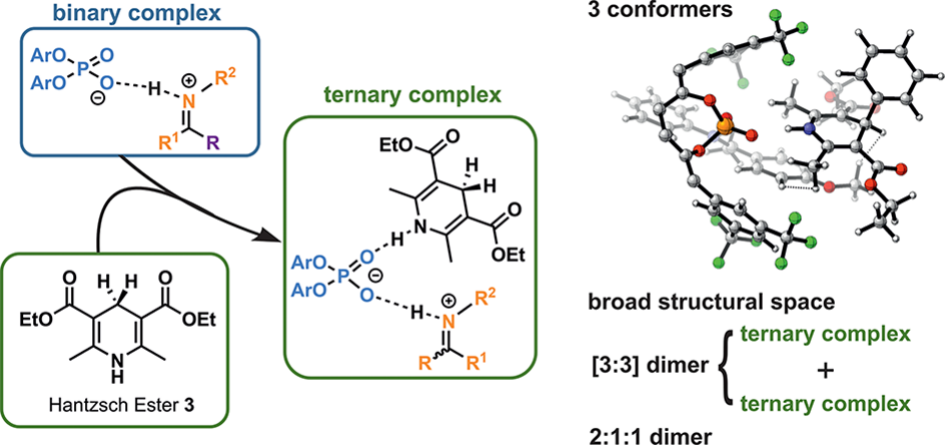

In ion-pair catalysis, the last intermediate structures prior to the stereoselective transition states are of special importance for predictive models due to the high isomerization barrier between *E*- and *Z*-substrate double bonds connecting ground and transition state energies. However, in prior experimental investigations of chiral phosphoric acids (CPA) solely the early intermediates could be investigated while the key intermediate remained elusive. In this study, the first experimental structural and conformational insights into ternary complexes with CPAs are presented using a special combination of low temperature and relaxation optimized ^15^N HSQC-NOESY NMR spectroscopy to enhance sensitivity. Combined NMR investigations and theoretical calculations revealed three conformers of the ternary complex, of which one also closely resembles the previously calculated transition states. In addition, a 2:1:1 ternary complex as well as an unprecedent [3:3] dimeric species consisting of two ternary complexes was revealed. Given the importance of the ground state energies for the transition state interpretation in ion pair catalysis we believe that the presented experimental insight into the structural and conformational variety of the ternary complexes is a key to the future development of predictive models in ion pair catalysis.

## Introduction

**Figure 1:**
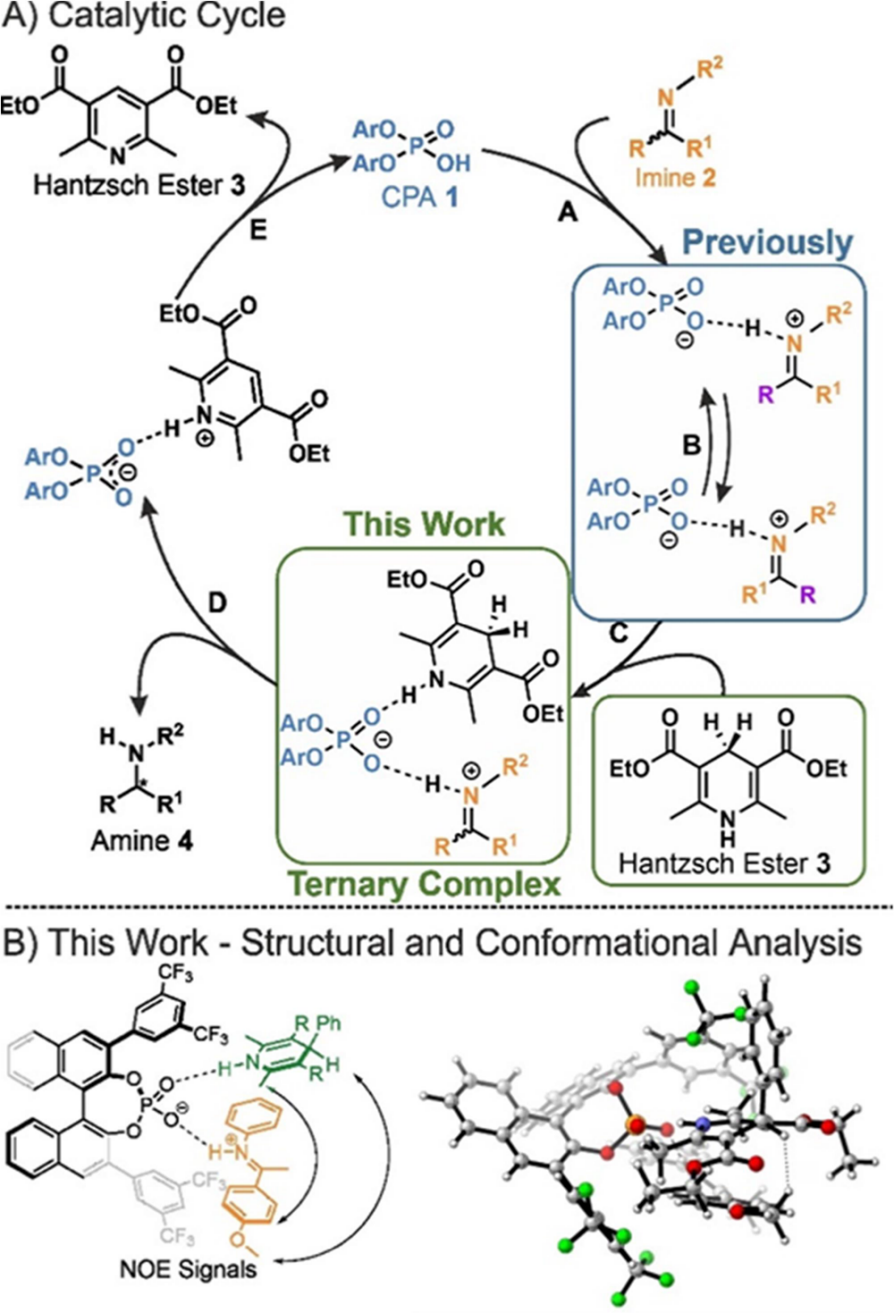
(A) Proposed catalytic cycle for the CPA-catalyzed transfer hydrogenation of imines. Prior studies elucidated structural details of the binary complexes but the ternary complex remained elusive for nearly a decade. (B) In this work, we accessed the ternary complex by using a HE derivate which is soluble even at 180 K needed for NMR investigations. The gathered measurements were used for a structural and conformational analysis in collaboration with computational methods.

The detection and identification of late reaction intermediates is pivotal for an in-depth understanding of mechanisms and thus predicting the reaction outcome. However, accessing these intermediates through experimental and computational methods often proves to be challenging. Especially for the BINOL (1,1′-binaphtol) derived chiral phosphoric acids (CPA), which have emerged as a prominent catalyst class with excellent stereoselectivities over the past decades,^1−12^ late intermediates have been experimentally elusive until now. Due to their remarkable success in organocatalysis, extensive theoretical, and analytical studies have already been conducted.^13−20^ In contrast, despite years of effort, experimental studies have only been able to investigate early intermediates such as the binary complex (see Figure 1A, blue). The important last intermediate prior to the stereoselective step had been inaccessible due to solubility issues of the hydrogenation agent as well as the complexity of the systems. Only theoretical calculations have been capable of establishing models for transition states and were able to derive complex data-driven prediction models.^21−23^

Nevertheless, despite the challenges associated with identifying reaction mechanisms involving hydrogen bonds and ion-pair character,^24,25^ experimental studies combined with calculations have provided a plethora of valuable insights into the early intermediates of the catalytic cycle originally proposed by Rueping et al. (see Figure 1A).^26^ Among them, an *E*/*Z*-isomerization was revealed during step A in which a binary complex is formed via a hydrogen bond-assisted ion-pair between CPA and imine.^17,25,27,28^ In addition, the 3,3′-substituents were found to play a crucial role in controlling the stereoselectivity.^26,29−35^ To classify the influence of the 3,3′-substituents, Goodman et al. introduced two parameters: proximal sterics described by the rotational barrier of the 3,3′-substituent and the cone angle AREA (θ) of the binding pocket.^13^ Furthermore, Goodman et al.*’s* computations postulated four distinct stereochemical arrangements for the CPA/imine binary complex,^13^ which were later experimentally confirmed by NMR studies.^31,32^ Of these four calculated conformations, two correspond to an *E*-imine species (Type I *E* and Type II *E*) and two to a *Z*-imine species (Type I *Z* and Type II *Z*), with Type I and Type II differing in the arrangement of the imine to the binaphthyl moiety of the CPA. Notably, one of each imine isomer leads to the correct enantioselectivity (Type I *E* and Type II *Z*).

Besides the structural investigations of the binary complex, the aggregation behavior of CPAs was extensively investigated. Despite the strong hydrogen bonds and sterically hindrance of the 3,3′-substituents, a variety of structural arrangements could be possible due to the ion-pair character of CPAs. Such aggregates could influence reactivity and selectivity by enabling new reaction pathways or serve as off-cycle equilibria. Indeed, several higher aggregates were identified by NMR spectroscopy.^31,36^ On the one hand, an off-cycle [2:2] dimeric species was revealed consisting of two binary complexes that enclose the imines in between two CPAs.^31^ Similar [CPA/imine]_2_ dimers are also observed using *N*-(*ortho*-hydroxyaryl) imines which lead to a bidentate binding that might contribute to the high stereoselectivity with these substrates.^37^ On the other hand, a 2:1 CPA/CPA/imine dimer was discovered with quinolines as substrate, for which calculations proposed that the quinoline forms a hydrogen bond to one CPA while the Hantzsch ester attaches to the second CPA.^38^ Here, experiments showed an inversion in stereoselectivity in comparison to the classic monomeric pathway induced by a secondary dimeric pathway.^36^

For now, however, prediction models are based on theoretical calculations of the energetically most probable monomeric CPA/imine/HE transition states.^14,15,23,39,40^ Even in the case of monomeric reaction pathways the high activation barrier of the substrate double bond is expected to create a special importance of the relative energies of the last, the ternary intermediate prior to the stereoselective step. Usually, only the relative transition state energies have to be considered. In case of a slow *E*/*Z*-isomerization the ground state energies of the last intermediate have to be taken into account, since *E*- and *Z*- pathways are mainly decoupled.^25^ Hence, the structures, the conformations and the potential aggregation of the elusive ternary complexes are of special interest for predictive models and the rational understanding of ion pair catalysis with CPAs.

However, experimentally advancing the ternary complexes with CPAs is deemed extremely challenging due to additional exchange processes that cause line broadening and shorten the transverse relaxation times (*T*2), worsening the resolution and signal intensities of 2D NMR spectra. Hence, our group performed studies of the ternary complex featuring a BINOL-derived chiral disulfonimide (DSI) catalyst, which is closely related to CPAs but better accessible for NMR measurements. Here, the first insights into the aggregation behavior of ternary complexes were provided based on NMR spectroscopy.^41^ Subsequently, we transferred this knowledge to CPAs and showed in a London-Dispersion study for the first time that ternary complexes of CPAs can also be detected by NMR spectroscopy.^42^ In consequence, the focus of our NMR investigations shifted toward the ternary complex of CPAs for more in-depth details on its structure and aggregation behavior. Nevertheless, structural and conformational investigations were still elusive based on standard methods for small molecules. Therefore, NMR methods for proteins could be used which are optimized for short *T*2 relaxation times and slow tumbling.^35^ Both are crucial in mechanistic studies of ternary complexes of CPAs, especially at low temperatures which also push the system in the slow tumbling regime.

Thus, this study aimed to provide experimental access to the late intermediates of CPAs using NMR spectroscopy. Therefore, we employed the ^15^N HSQC-NOESY pulse sequence under optimized NMR conditions for our systems, thereby enhancing sensitivity and yielding more comprehensive data for in-depth investigations. Based on this approach, the structural and conformational variety of ternary complexes with CPAs was investigated for the first time. The structural space was especially examined in regard to previously reported structures, such as the 2:1 dimeric species. Here, we observed the 2:1:1 ternary complex for the first time and confirmed that the Hantzsch ester forms a hydrogen bond to the second CPA, as proposed by calculation.^36^ Furthermore, an unprecedented [3:3] dimeric species was revealed in which imine and HE are enclosed by two CPAs leading to the first observed additional separated hydrogen bond signal of the HE. Besides the structural investigations, the first conformational analysis of the 1:1:1 ternary complex of CPAs was conducted by NMR spectroscopy and combined with calculations revealing three conformers. Notably, the most stable conformer also closely resembles the calculated transition states, thereby validating these for the first time experimentally. This structural and conformational variety of late intermediates in CPA catalysis was not unraveled in prior theoretical studies, underscoring the crucial role of experimental studies in deepening our comprehension of complex structures’ conformational space.

## Results and Discussion

### Model Systems and Experimental Setup

**Figure 2:**
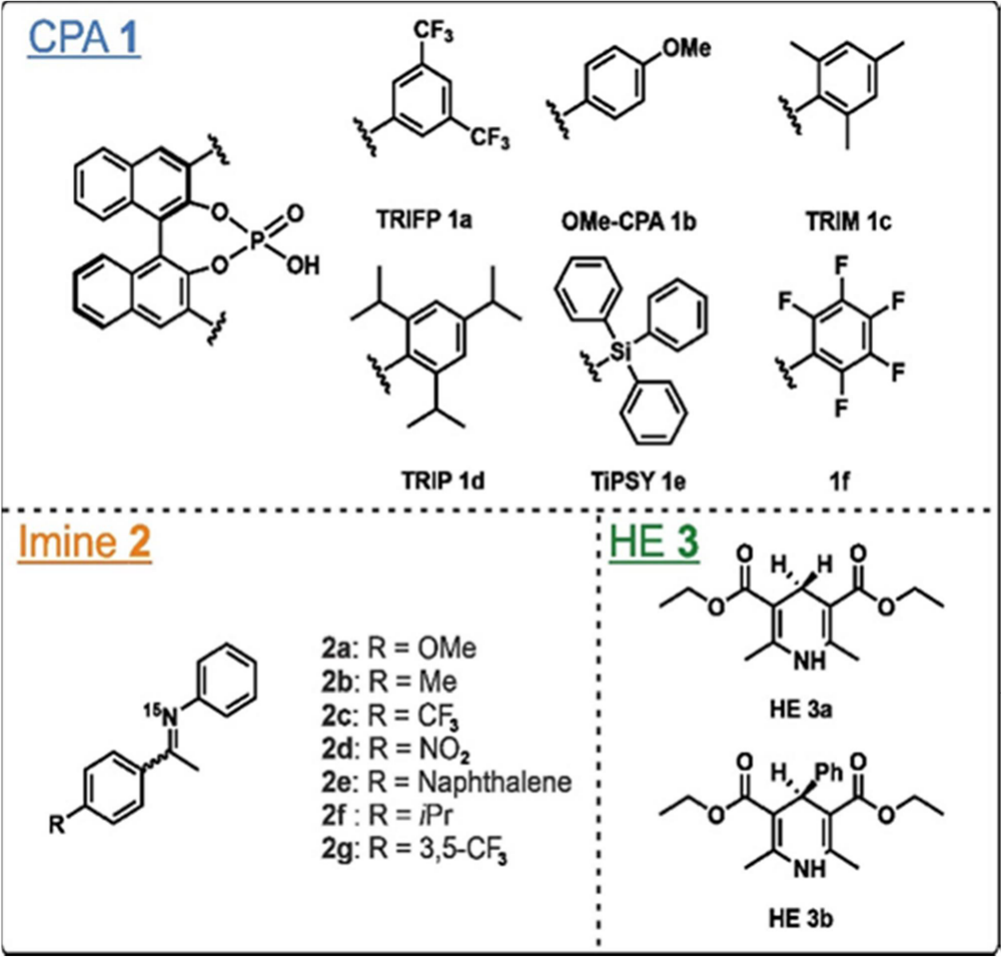
Model compounds used in the screening for ternary complexes comprised of (*R*)-CPAs **1**, imines **2** and HE **3**. Twenty-five combination of imines **2a–g** with CPAs **1a–f** and **3b** were screened.

In order to investigate transfer hydrogenation reactions of CPAs the last intermediates before the stereoselective step/transition state, 25 ternary complexes comprised of different CPA **1**/imine **2**/HE **3** combinations were screened in a 1:1:1 stoichiometry at 180 K (see Figure 2). This low temperature is crucial to slow down exchange processes, and enables the detection of hydrogen bonds. Therefore, we used dichloromethane (DCM) as solvent which combines low temperature accessibility, excellent NMR properties of the complexes and very high ion pair formation trends.^43^ In prior attempts of accessing the ternary complex by NMR spectroscopy, the poor solubility of the HE **3a** at 180 K as well as product formation during the sample preparation hindered any structure elucidation for several years. In the study of DSIs,^41^ the HE derivate **3b** with a phenyl-group, substituting one of the hydrogen atoms distant from the binding site, was successfully employed to improve solubility and to reduce reactivity. Also, for complexes with CPAs the HE derivate **3b** proved itself to be superior to **3a** and allowed us to investigate the ternary complex with concentrations of up to 50 mM in a 1:1:1 stoichiometry of CPA **1**/ imine **2**/ HE **3**. In addition, both the imine **2** and HE **3** were ^15^N-labeled (^15^N spin number = ) for easier identification of hydrogen bonds between the CPA **1** and imine **2** (PO^–^–H–N^+^) as well as between the CPA **1** and HE **3** (PO–H–N) due to their doublet splitting. Different CPAs **1a–f** and imines **2a–g** were selected with varying steric and electronic properties due to their significant influence on the NMR properties of the complexes observed in prior studies.^44^ The screening of 25 systems revealed some general characteristics of the formation of ternary complexes. Despite the steric hindrance of the 3,3′-substituents, the ternary complex was detected for each CPA (see SI chapter 6.1). It also revealed that CPAs with a relatively high AREA (θ) value and low rotational barrier,^13^ such as TRIFP **1a** (AREA (θ) = 4.03; rotation barrier = 2.02 kcal mol^–1^) and OMe-CPA **1b**, result in sharper spectral lines, better suited for NMR investigations and a higher formation trend of ternary complexes.

The combination of TRIFP **1a**/**2a**/**3b** showed the best NMR properties (high ternary complex formation, acceptable line widths, CF_3_ groups for ^1^H–^19^F-HOESY) and was therefore selected as model system. Besides the hydrogen bond signals of the ternary complex, additional yet unknown signals were observed in the hydrogen bond area of the ^1^H-spectrum during system screening. Here, one signal pattern of two doublets was observed in several systems, which hinted toward a potential new aggregate of the ternary complex. To study these signals, CPA **1b** with a *p*-methoxy-phenyl as 3,3′-substituent (OMe-CPA **1b**) was selected as the second model system as it showed the highest preference of this new species out of all screened systems.

### Structural Space

**Figure 3:**
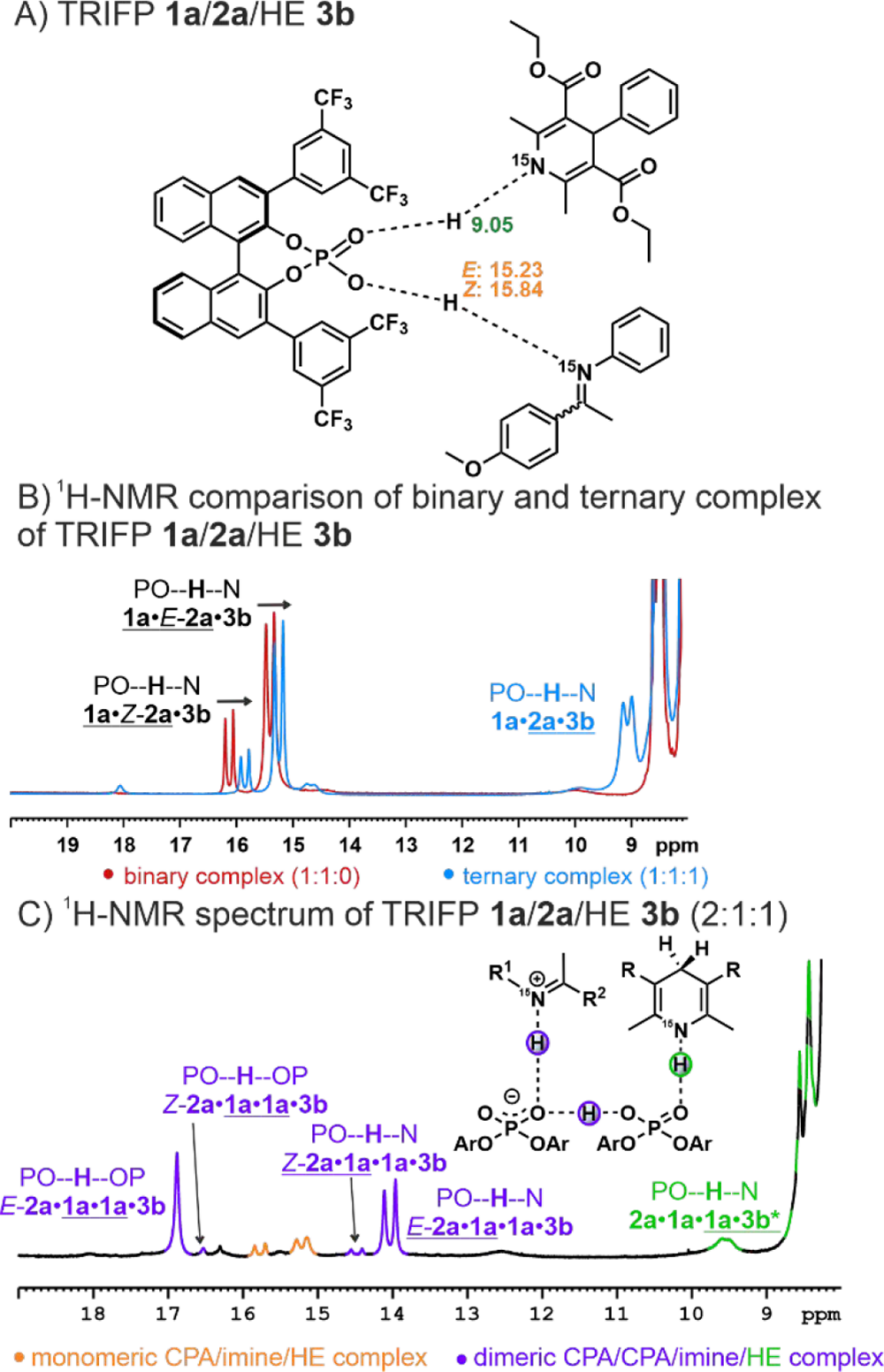
(A) Structure of the ternary complex of **1a**/**2a**/**3b** with the proton chemical shifts of the hydrogen bond signals. (B) ^1^H NMR spectrum comparison of the binary (red, 1:1:0 stoichiometry, 40 mM, CD_2_Cl_2_, 180 K) and the ternary complex (blue, 1:1:1 stoichiometry, 40 mM, CD_2_Cl_2_, 180 K) of the system **1a**/**2a**/**3b**. Upon addition of **3b**, a highfield shift of both CPA/imine hydrogen bond signals is observed. (C) ^1^H NMR spectrum of the **1a**/**2a**/**3b** system in a 2:1:1 stoichiometry (40:20:20 mM, CD2Cl2, 180 K). This sample demonstrated that also for the dimeric 2:1 species (purple) it is possible to form a complex with **3b**. The respective hydrogen bonds are underlined for each species. *3b hydrogen bond signals cannot be unambiguously assigned due to an overlap with other signals (marked in green).

First, the hydrogen bond area in the ^1^H-spectrum (from 9 to 19 ppm) of TRIFP **1a**/**2a**/**3b** was evaluated to identify the ternary complex (see Figure 3). While the presence of a ternary complex is indicated by a new CPA/HE hydrogen bond signal (9.05 ppm) only one signal set for the CPA/imine hydrogen bond (two doublets, one for each imine isomer) is observed suggesting a fast exchange of the binary and ternary complex on the NMR time scale even at 180 K. This was corroborated by a chemical shift mapping in which both signals of the binary complex are highfield shifted upon the addition of **3b** to the system of **1a**/**2a** (see Figure 3B and SI chapter 3.7). Similarly, only one CPA/HE signal is observed for the ternary complexes in all ^1^H ^15^N correlation spectra. This indicates that this signal is an averaged HE signal of both *E*- and *Z*-ternary complexes as well as the free HE. Hence, these species are also in fast exchange. Additionally, further analysis of the CPA/imine hydrogen bonds shows that the CPA/*Z*-imine signal is further highfield shifted (−0.27 ppm) than the CPA/*E*-imine (−0.16 ppm). From the fitting of the chemical shift mapping data the binding constants were calculated showing that **3b** is only weakly bound to the binary complex (*Z*-**2a**: *K*_a_ = 22.2; *E*-**2a**: *K*_a_ = 19.8) similar to the situation in DSIs.^41^

For quinolines, complexes with dimeric catalyst structures were found to influence the stereoselectivity,^36^ therefore next the potential presence of catalyst dimers in the ternary complexes was tested. So far, exclusively the binary complex of the 2:1 dimer has been experimentally investigated, supported by calculations that proposed the HE forms a hydrogen bond to the second CPA. However, so far, there has been no experimental evidence of whether the HE binds to the second CPA or to the same CPA as the substrate. Therefore, a 2:1:1 stoichiometry of **1a**/**2a**/**3b** was used to force the system into this dimeric species (which is not observed in a 1:1:1 stoichiometry) and to investigate the binding behavior of the HE in this complex. In accordance with the 2:1 binary complex signal pattern, two singlets representing the CPA **1**/CPA **1** (PO···H···OP) hydrogen bonds of both imine isomers and two doublets for the CPA **1**/CPA **1**/imine **2** (PO···H···N) hydrogen bonds of both imine isomers were detected (see Figure 3C, purple). In addition, for the first time a signal for the hydrogen bond between HE and CPA in such a **1a**/**1a/2a**/**3b** complex was detected (see Figure 3C, green). Similar to the 1:1:1 ternary complex, a fast exchange process between 2:1:1 complexes with and without **3b** was observed and corroborated by a chemical shift mapping (see SI chapter 3.6.2). Moreover, the chemical shift mapping revealed a significantly higher shift for the CPA/CPA (PO···H···OP; up to 0.38 ppm) hydrogen bond signals compared to the CPA/CPA/imine signals (PO···H–N; up to −0.08 ppm). This observation indicates that the chemical environment of the CPA **1**/CPA **1** hydrogen bond is more impacted by the addition of the **3b** than the CPA **1**/CPA **1**/imine **2** hydrogen bond. This finding supports Grimme et al.'s calculations in which the substrate is attached to one CPA while the HE is attached to the other CPA of the dimer.^36,38^ Thus, this is the first time the 2:1:1 complex is experimentally detected and chemical shift mapping indicates that imine and Hantzsch ester bind to different catalyst molecules.

Next, the structural space of the 1:1:1 ternary complex was further investigated. In prior studies, no additional structures of ternary complexes were considered.^15,42^ However, in all 25 systems screened multiple additional hydrogen bond signals were detected. Considering the remarkable impact of the dimeric pathway with quinolines which was only detected as a small populated species by NMR,^36^ the elucidation of such additional late reaction intermediates could be crucial for any prediction model.

**Figure 4:**
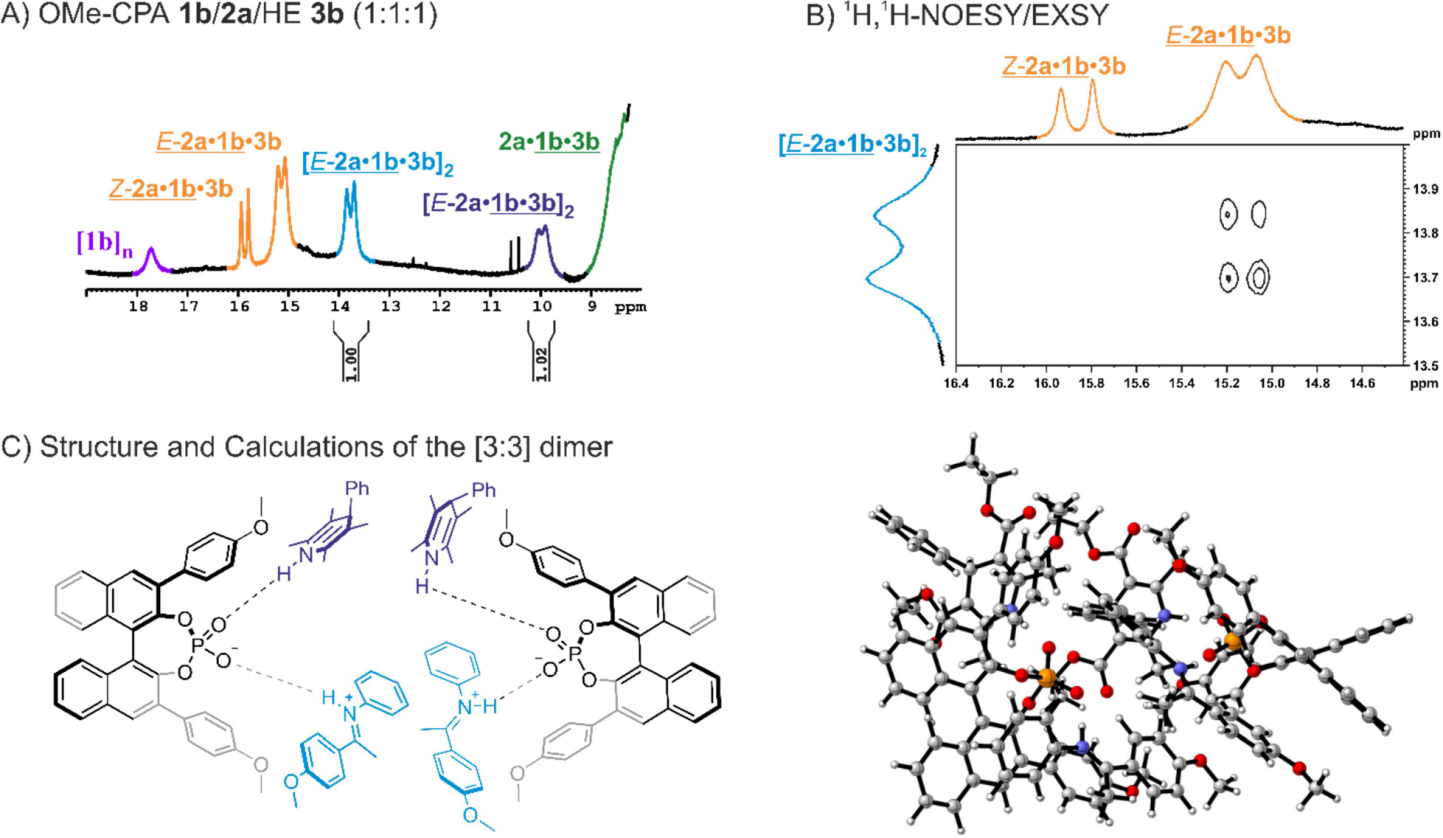
(A) ^1^H NMR spectrum of the OMe-CPA **1b**/**2a**/**3b** system (1:1:1 stoichiometry, 40 mM, CD_2_Cl_2_, 180 K). The hydrogen bonds between CPA **1**/imine **2** (orange) and CPA **1**/**3b** (green) can be assigned to the ternary complex. The [CPA]_*n*_ aggregate is highlighted in pink. Additionally, two new hydrogen bond signals are observed ([*E*-**2a**/**1a**/**3b**]_2_ in blue; [*E*-**2a**/**1b**/**3b**]_2_ in purple; the respective hydrogen bond is underlined), which can be identified as a [3:3] dimeric species consisting of two ternary complexes. (B) One part of the ^1^H–^1^H NOESY/EXSY of this system is displayed showing the exchange between the [3:3] dimer and the **1b**/*E*-**2a** hydrogen bond signal which reveals the [3:3] dimer as an *E*-imine species. (C) Schematic display of the [3:3] dimer (left) and calculated structure of the [3:3] dimer (right; see SI chapters 4.1 and 7.5).

Besides a singlet at approximately 18 ppm (see Figure 4a, purple), which appeared in most systems and seems to be related to a CPA aggregate [CPA]_*n*_, an additional signal pattern was observed regularly, twice even as the major species (see SI chapter 3.6.1). For these investigations, the **1b**/**2a**/**3b** system was selected as a model system due to the high formation of this species (see Figure 4A, blue). In general, both signals are doublets and appear together indicating that they belong to the same species. To identify the underlying hydrogen-bonded substrates of each signal, samples were prepared in which either the imine **2a** or the **3b** was ^15^N-labeled. Thereby, the signal at 13.77 ppm (blue) was identified as a CPA/imine hydrogen bond which was later elucidated as an *E*-imine species by EXSY correlations (see Figure 4B). Furthermore, the signal at 9.98 ppm was identified as a separate CPA/HE hydrogen bond (see SI chapter 3.6.1). In contrast to prior studies where only averaged signals for the **3b** were observed due to fast exchange processes,^41,42^ this separated CPA/HE hydrogen bond indicates a species, in which the exchange processes have to be slowed down possibly caused by a locked system that hinders the **3b** from a fast exchange with the binary and ternary complexes.

Besides these initial observations, the **1b**/*E*-**2a** hydrogen bond signal is high field shifted in comparison to other CPA/imine hydrogen bond signals (see Figure 4A). Similar chemical shifts were previously reported only for a [2:2] dimer of two CPAs that lock two imines between them, which also occurred exclusively as an *E*-imine species.^31^ Considering the integral ratio of 1:1, the same amount of CPA/*E*-imine hydrogen bonds as CPA/HE hydrogen bonds within this complex has to occur (see Figure 4A). Hence, a first assumption would be a [3:3] dimer consisting of two ternary complexes ([CPA/imine/HE]_2_) where the CPAs enclose both imine **2a** and **3b** (see Figure 4C). To validate that the signals correspond to a dimeric species, dilution experiments were performed. Here, a significant decrease in both [3:3] dimer signals was observed compared to the hydrogen bond signals of the binary/ternary complex (see SI chapter 3.6.1), supporting the [3:3] dimer hypothesis. Furthermore, the uniform decrease of both the CPA/imine as well as the CPA/HE hydrogen bond signals supports the observation that both are related to the same complex. Next, diffusion ordered spectroscopy (DOSY) measurements were conducted for validation of a higher aggregation (see SI chapter 3.8). For DOSY evaluation, the hydrogen bond signals cannot be used, so other well-separated signals, such as the OMe-group signals for **1b**, were utilized. However, for these signals, the exchange between binary/ternary complexes has to be considered which results in an averaged signal set for the aliphatic and aromatic signals for the proposed [3:3] dimer. Hence, using these signals for DOSY results in an averaged volume of all species for each substrate, rather than the volume of one specific complex. For comparison purposes, separate samples with a 1:1 stoichiometry were measured for the binary complex and for **3b** alone. Here, a volume of 1625 Å^3^ was determined for the binary complex and 506 Å^3^ for **3b**. Combining these values yields an estimated volume of 2131 Å^3^ for the ternary complex. However, the average volume of all CPA species inside the 1:1:1 sample is 2331 Å^3^, significantly exceeding the estimated volume of the ternary complex (2131 Å^3^). Thus, a higher aggregate has to be responsible for this significant increase in volume, which also supports the [3:3] dimer hypothesis. Besides NMR experiments, calculations were performed to verify a [3:3] dimer. Despite separate hydrogen bond signals, the exchange processes identified by EXSY signals prevent a NOESY analysis of the [3:3] dimer due to its similarities to the ternary complex, making it difficult to differentiate NOE correlations clearly. Nevertheless, calculations resulted in a [3:3] structure that encloses imine **2a** and **3b** which supports the initial assumption of a locked system based on the additional separated hydrogen bond signal for **3b** (for further details see SI chapters 4.2.2 and 7.5). Thus, the [3:3] dimer model is supported by both computations as well as NMR data, including the integral ratio of approximately 1:1 of the **1b**/*E*-**2a** to **1b**/**3b** hydrogen bond signals, chemical shifts, DOSY and dilution experiments (for further discussion see SI chapter 3.6.1). In conclusion, we were able to experimentally confirm the ternary formation of the ternary complex, we were also for the first time able to identify two higher aggregates as late intermediates: a 2:1:1 dimeric species and a previously unknown [3:3] dimeric species consisting of two ternary complexes.

### Accessing the Ternary Complex

**Figure 5:**
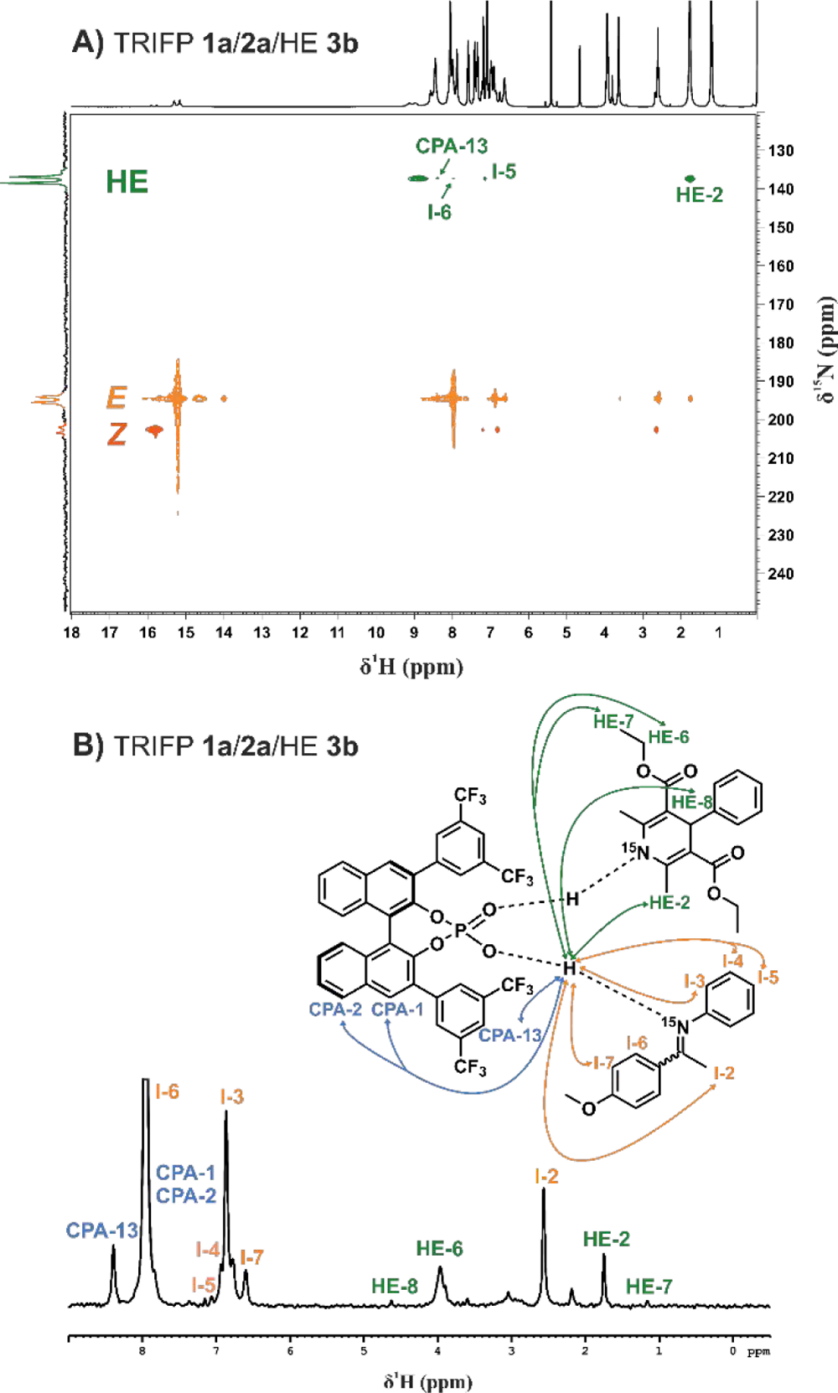
(A) ^15^N-HSQC-NOESY spectrum of **1a**/**2a**/**3b** (1:1:1 stoichiometry, 40 mM, CD_2_Cl_2_, 180 K). Separated rows for the CPA/*E*-imine (orange), CPA/*Z*-imine (red) and CPA/HE (green) hydrogen bonds can be observed. (B) Single rows can be displayed as 1D spectra for more detailed information to reveal the structural environment of each hydrogen bond, here exemplary displayed for the CPA/*E*-imine hydrogen bond (δ (^15^N) = 195 ppm).

Next, we analyzed the structural arrangement of ternary complexes with CPAs. Here, we focused on the 1:1:1 ternary complex of our model system TRIFP **1a**/**2a**/**3b**.^45^ For the classic approach known from the binary complexes, ^1^H,^31^P-HMBC is used as a starting point for the chemical shift assignment of any CPA structures. This method provides information about the central region of the ternary complex and the connection of all substrates. However, in the **1a**/**2a**/**3b** system binary and ternary complexes are in fast chemical exchange even at 180 K resulting in signal broadening and reduced chemical shift resolution. These exchange processes are particularly pronounced in the phosphorus dimension resulting in poor chemical shift resolution in the ^1^H,^31^P-HMBC spectra which deems the classic approach infeasible. Therefore, a variety of NMR methods were tested and finally optimized to enable an improved approach for the chemical shift assignment. Here, especially the center of the ternary complex which consists of a PO^–^–H–N^+^ (CPA/imine) and PO–H–N (CPA/HE) hydrogen bond was of interest. The possibility of ^15^N-labeling both imine **2a** as well as the **3b** moved ^15^N-2D-NMR spectra into the focus. At 180 K, the CPA/Imine/HE system is in the slow-tumbling regime, making the NMR pulse sequences commonly employed in protein studies suitable for our purposes. The ^15^N-HSQC-NOESY experiment, utilizing the pulse sequence from the Bruker library hsqcetgpnosp with adiabatic pulses, is a method applied in protein research. It combines two robust NMR techniques: the ^15^N HSQC (heteronuclear single quantum coherence) and the NOESY (nuclear Overhauser effect spectroscopy). In the ^15^N HSQC component, correlations between nitrogen and proton nuclei within proteins are observed, providing insights into the chemical environment. Conversely, the NOESY component detects NOE effects, uncovering interactions between nearby nuclear spins. These interactions offer valuable distance constraints, aiding in the determination of a protein’s three-dimensional structure. By integrating these techniques, the ^15^N HSQC-NOESY experiment yields comprehensive structural details about the CPA/Imine/HE (see Figure 5A). With this pulse sequence, a reduced number of cross-signals is detected in comparison to ^1^H–^1^H NOESY, which minimizes signal overlapping significantly. The unexpected higher sensitivity of the ^15^N HSQC-NOESY compared to the ^1^H–^1^H NOESY is primarily attributed to the significantly longer T2 relaxation times of the ^15^N signals relative to the ^1^H signals. This characteristic reduces magnetization loss during the evolution period (τ), thereby preserving more signal and resulting in enhanced sensitivity (for further details, see SI chapter 5). This phenomenon is already known^46,47^ and has been used in the studies of hydrogen bonding of different systems such as proteins and DNA,^48−52^ but to our knowledge this technique has not yet been applied to catalyst complexes so far. The use of one-dimensional rows of this spectrum, at the ^15^N frequency of interest, offers an enhanced method for observing inter- and intramolecular contacts (Figure 5B). Moreover, detailed knowledge about the center of the ternary complex and hydrogen bond site can be obtained. Thus, based on the initial screening, optimized sample preparation, ^15^N-HSQC-NOESY, and the selection of the **1a**/**2a**/**3b** system which has the better chemical shift resolution, after initial attempts by our group^41,42^ it is for the first time possible to achieve detailed structural information on a ternary complex of a chiral phosphoric acid.

For the chemical shift assignment ^15^N HSQC-NOESY was used to attribute signals to CPAs (see Figure 5B). Based on these signals, standard 2D spectra were used to assign the remaining protons of TRIFP **1a**. Besides ^15^N-HSQC-NOESY (see Figure 5B), ^1^H,^15^N HMBC can be used for the assignment of both imine **2a** and **3b** also. From here onward, again 2D spectra are used to assign the remaining signals. An additional benefit of TRIFP **1a** is the accessibility of ^1^H,^19^F HOESY, which provides additional structural information (see SI chapter 3.3). After completing the chemical shift assignment, the structural environment of the hydrogen bonds was subsequently analyzed. For the CPA/imine hydrogen bond, a plethora of NOE correlations were found between the hydrogen-bonded proton to TRIFP **1a** and the **3b** (see Figure 5A,B), indicating a confined arrangement of all three substrates. In addition, despite the fast exchange between the **3b** and the binary complex, a few cross signals were still detected in the ^15^N-HSQC-NOESY spectrum for the **1a**/**3b** interaction. Thus, cross signals of the **1a**/**3b** hydrogen bond to TRIFP **1a** and **2a** are observed which support the confined arrangement of the ternary complex.

The new approach for the chemical shift assignment enables unambiguous structural investigations of the ternary complex, applicable across a wide range of substrates for the first time. The resulting chemical shift assignments establish the basis for conformational investigations of the ternary complex of CPAs which were previously beyond reach.

### Conformational Investigations

So far, there has been no comprehensive conformational analysis of 1:1:1 ternary complexes of CPAs. Hence, we aimed to elucidate their conformational space based on the established NMR methods complemented by theoretical calculations. Therefore, we employed our TRIFP **1a**/**2a**/**3b** model system to utilize ^1^H,^19^F-HOESY in addition to ^1^H,^1^H NOESY and ^15^N HSQC-NOESY. Furthermore, the 1:1:1 ternary complex is the major populated ternary species in this system, thus no other ternary species are interfering with our NOE analysis. Nevertheless, the fast exchange between binary and ternary complexes, which is still present at 180 K, has to be considered for a NOE interpretation. This leads to a challenging NOE analysis as only interactions involving **3b** can be considered, since **3b** is only associated in ternary complexes (for details see SI chapter 4.2). The initial analysis of this **3b** NOE network suggested the presence of multiple conformers for the 1:1:1 ternary complex, since a single conformation could not fulfill all detected distances. Therefore, first potential conformations were calculated and then characteristic **3b** NOE correlations interpreted that are unique for each respective conformer.

**Figure 6:**
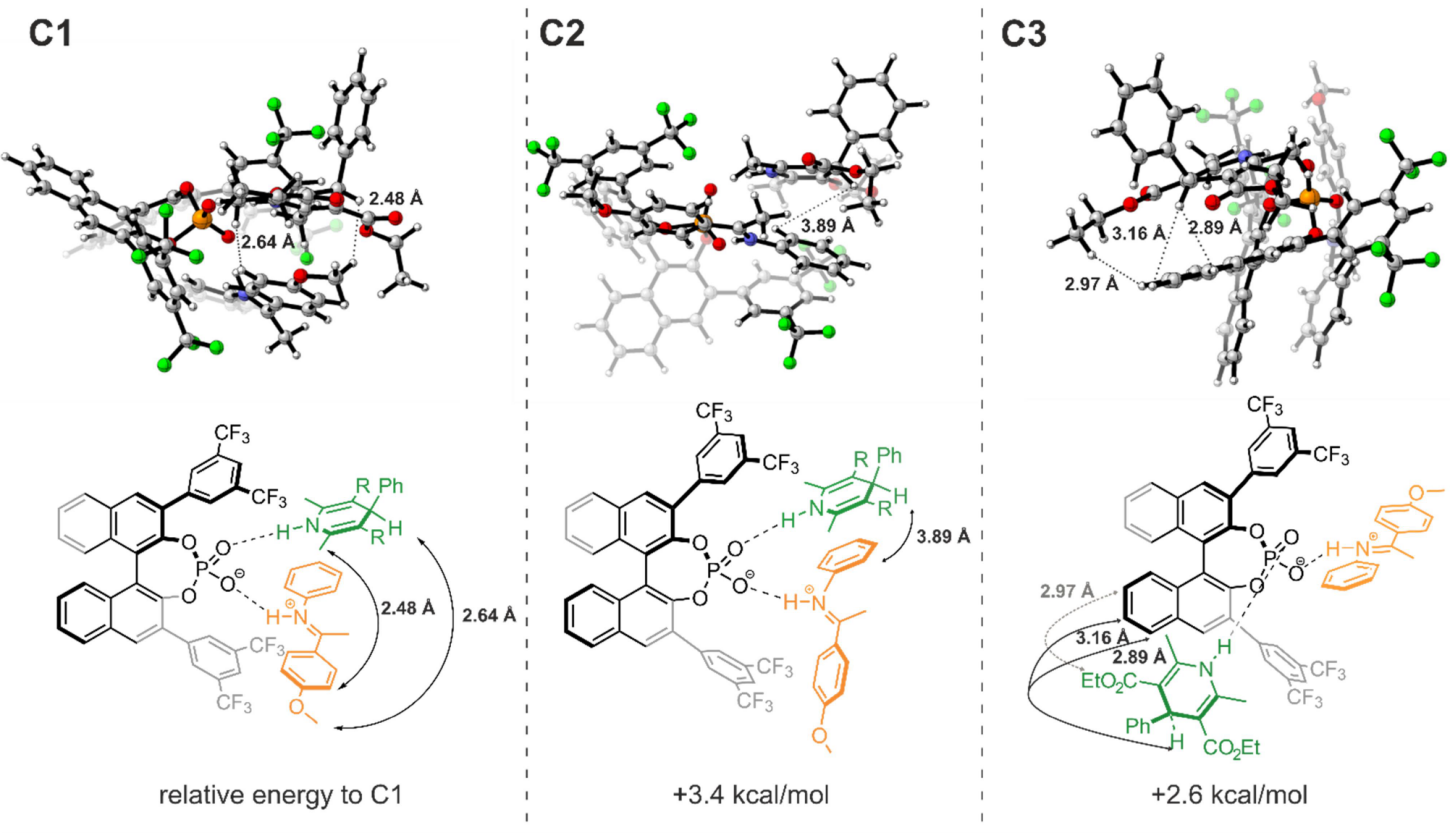
Conformational analysis of the ternary complex. Three conformers were computed and validated by NMR spectroscopy. All three conformers coexist, with energetic stability (Δ*G* values) decreasing in the following order: C1 > C3 > C2. C1 and C2 are reactive in this state, while for C3 rearrangements are necessary to lead to a hydrogenation of the imine. The conformers are validated by distinct NOE cross signals of the **3b**. For the sake of clarity out of a large NOE network only the NOEs characteristic for the individual conformation are depicted.

Thus, an initial conformational search was performed with CREST^53^ which uses meta-dynamics.^54^ The B97-D^55^ functional combined with def2-SVP^56^ basis set was used for geometry optimization and frequency analysis in ORCA 5.0.3 software package.^57^ The global and local minimum energy structures were confirmed by absence of any imaginary frequencies. To adapt the experimental conditions of 180 K and CD_2_Cl_2_ all the calculations were performed in the CPCM^58^ with the dielectric constant ε = 16.20 (see SI chapter 4.1). These calculations resulted in eight conformers, of which only the three lowest-energy conformers, with a cutoff at +4.5 kcal/mol relative to the lowest-energy conformer, were considered (see Figure 6). Based on these conformers, the aforementioned NMR approach of ^1^H,^1^H NOESY and ^15^N HSQC-NOESY was utilized and further corroborated by ^1^H,^19^F HOESY spectra.

First, the lowest energy conformer C1 with a face-to-face arrangement of **2a** and **3b** inside the binding pocket of TRIFP **1a** was analyzed by NMR spectroscopy. Especially NOE correlations of the **3b** proton (chemical shift: 4.66 ppm, referred to as *HE-8*) which is neighboring the phenyl group are indispensable as the arrangement of this proton to the imine and CPA is significantly different for all conformers. For C1 *HE-8* is close to the methoxy group of the imine. In addition, the methyl groups of the **3b** are close to the *p*-methoxy-phenyl ring of the imine. Both correlations are uniquely observed for C1 and confirmed by NOESY (see Figure 6, left). In conformer C2 (+3.4 kcal/mol relative to C1), a similar arrangement to C1 is observed with both substrates positioned inside the binding pocket of TRIFP **1a**, but the imine is slightly flipped. This results in a distinct close proximity of the *N*-phenyl-moiety of the imine to **3b** (*HE-8*), which is proven by NOE correlations (see Figure 6, middle). Hence, both C1 and C2 can be experimentally validated and clearly distinguished. Comparing both structures with the Type I *E* and Type II *E* structures of the binary complex shows similarities of both C1 and C2 to the Type I *E* structure indicating that both could originate from this conformation. Furthermore, the third conformer C3 (relative energy to C1: +2.6 kcal/mol) is also observed by the NOE network. Contrary, C3 is similar to Type II *E* with a flipped imine inside the binding pocket and further differs from C1 and C2 as the **3b** is positioned outside the binding pocket. The latter results in distinct interactions between *HE-8* and the BINOL-backbone of TRIFP **1a** which are detected by NOESY (see Figure 6, right). Thus, not only a broad structural space with three confirmed complexes but also a conformational space with three different conformers was identified. Considering the utilization of a phenyl-substituted **3b**, which is more sterically demanding than commonly employed HE **3a**, even more conformers could be observed using HE **3a**. These results demonstrate that the steric limitations of the 3,3′-substituent do not limit the aggregation of ternary complexes as much as one would expect.

To investigate if only one or more of these conformers could be reactive, the computed conformers were analyzed for possible reaction pathways. Here, for conformers C1 and C2 the reactive hydrogen atom of the **3b** was found to be close enough to the N=C bond to progress the reaction in the respective stereoselectivity which is observed for (*R*)-TRIFP **1a**. In addition, comparing conformer C1 with previously computed transition states for this reaction reveals several similarities.^39^ In particular, the face-to-face arrangement of imine **2a** and **3b** exists in both C1 and the postulated transition states for TRIFP **1a**.^14^ Therefore, it is likely that the transition states arise from conformer C1, which is the first experimental validation of the ternary complex before the transition states. Additionally, conformer C2 depicts another reactive structure, which also leads to the correct stereoselectivity. Despite C2 having relatively higher energy than C1 (+3.4 kcal/mol), the fact that C2 is populated even at 180 K demonstrates that it cannot be disregarded for the prediction models of the reaction which typically is performed at 303–333 K.^26,29^ Contrary to C1 and C2, in conformer C3 the **3b** is positioned outside of the binding pocket. Due the resulting distance between imine **2a** and **3b**, an immediate reaction is not possible. Despite being an unreactive conformer, identifying C3 contributes to the understanding of sterically demanding CPAs (e.g., TRIP **1d**, TiPSY **1e**). Normally, the presence of a plethora of structures would be expected to decrease stereoselectivity. However, despite detecting multiple additional hydrogen bond signals in TRIP **1d** and TiPSY **1e** systems, both are two of the most prominent CPAs with the highest selectivity.^29,30^ However, considering C3, it is reasonable to assume that due to the steric constraints of these CPAs, multiple structures could be formed in which the HE is outside of the binding pocket. Consequently, these structures would not affect stereoselectivity as they are unreactive, despite being detectable by NMR spectroscopy.

In summary, the combination of computations and NMR spectroscopy revealed a broad conformational space consisting of three conformers. Among them, two conformers have an arrangement of imine **2a–3b** that could facilitate the reaction. Despite the nonreactive arrangement of conformer C3, identifying this conformer contributed to the understanding of the hydrogen bond signals of sterically more demanding CPAs. In addition, the first experimental validation of the precursor of the previously calculated transition states was achieved as conformer C1 of the ternary complex was found to closely resemble the transition states.

## Conclusions

While CPAs have been under scientific investigations due to their tremendous success in organocatalysis for almost a decade, experimental investigations have struggled to provide insights into later reaction steps. Until now, the binary complex of CPAs has been the main focus for any experimental studies. While these studies have yielded crucial insights, the development of an experimental prediction model for stereoselectivities based on these early intermediates remains a formidable challenge. Consequently, attention has shifted toward investigating subsequent reaction intermediates, which are anticipated to bear greater resemblance to the transition state.

Therefore, in this study we conducted the first experimental structural and conformational analysis of ternary complexes with CPAs. First, we took advantage of the T2 relaxation properties of the TRIFP **1a**/**2a**/ **3b** model system and employed the ^15^N HSQC-NOESY pulse sequence with the appropriate acquisition parameters to investigate the ternary complex. By combining this method with standard 2D NMR spectra, an extensive structural and conformational space was revealed. Thereby, we identified not only the ternary complex of CPAs, but also a novel [3:3] dimeric species consisting of two ternary complexes, as well as a dimeric 2:1:1 ternary complex. Accessing the 2:1:1 ternary complex also confirmed experimentally for the first time that imine and Hantzsch ester are bound to different catalyst molecules as previously proposed by computational chemistry. These revelations showcase that despite the sterically hindrance of CPAs, higher aggregates are still formed in later reaction steps and should be considered in prediction models. Besides the structural investigations, we combined theoretical calculations and NMR spectroscopy to obtain and analyze conformers of the 1:1:1 ternary complex. Here, three different conformers were observed of which two could lead to a reactive transition state. One of these two even closely resembles the previously calculated transition states and therefore provides the first experimental validation for them.

In summary, a new approach to access the ternary complex of CPAs has been established. Based on this approach, a new [3:3] dimer and a ternary 2:1:1 complex were revealed along with a broad conformational space. Considering the significance of ground state energies for interpreting the transition states in ion-pair catalysis, the presented experimental insights into the structural and conformational diversity of ternary complexes could be crucial for the future development of predictive models in ion-pair catalysis.

## Supporting Information Available

Synthesis and characterization of substrates; NMR system screening; DOSY experiments; discussion of species; and calculations and NMR pulse sequences (PDF)

## Abbreviation

HE: Hantzsch Ester
CPA: Chiral Phsophoric Acid
TRIFP: (*R*)-3,3′-Bis(3,5-trifluoromethyl)-1,1′-binaphthyl-2,2′-Diylhydrogen phosphate
TRIP: (*R*)-3,3′-Bis(2,4,6-triisopropylphenyl)-1,1′-binaphthyl-2,2′-Diylhydrogen phosphate
TiPSY: (*R*)-(−)-3,3′-Bis(triphenylsilyl)-1,1′-binaphthyl-2,2′-diyl hydrogen phosphate
