# Supplementary material for: The Elusive Ternary Intermediates of Chiral Phosphoric Acids in Ion Pair Catalysis—Structures, Conformations, and Aggregation
Source: J Am Chem Soc. 2025 Jan 9;147(3):2549–58. doi: 10.1021/jacs.4c14096 (PMC11760147; doi:10.1021/jacs.4c14096)
Supplement: Supplementary file 1 — ja4c14096_si_001.pdf [file ja4c14096_si_001.pdf]

Supporting Information

The Elusive Ternary Intermediates of Chiral Phosphoric Acids in Ion Pair Catalysis – Structures, Conformations and Aggregation

Maximilian Franta, Aryaman Pattanaik, Wagner Silva, Kumar Motiram-Corral, Julia Rehbein* and Ruth M. Gschwind*

Institute of Organic Chemistry, University Regensburg, Universitätsstr. 31, 93053 Regensburg, Germany.

Table of Contents

1. Analytical Methods 4
1.1. NMR Spectrometer Data 4
1.2. Pulse Sequences and Parameters 4
2. Materials and Chemical 5
2.1. Solvents 5
2.2. Chemicals 5
2.3. Synthesis of Imines 6
2.4. Synthesis of Hantzsch Ester derivate 3b 6
3. NMR Investigations 7
3.1. Substrate Scope 7
3.2. General Procedure for Sample Preparation and NMR-Measurements (GPSP) 9
3.2.1. E-only samples 9
3.3. Chemical Shift Assignment 10
3.3.1. Assignment strategy 10
3.3.2. TRIFP 1a/E-2a/HE 3b assignment 12
3.3.3. OMe-CPA 1b/E-2a/HE 3b assignment 13
3.3.4. OMe-CPA 1b /Z-2a/HE 3b assignment 13
3.4. Exclusion of other species 14
3.5. General features of ternary complex 17
3.5.1. Binary vs. ternary complex 17
3.6. Structural Space 18
3.6.1. [3:3] dimer 18
3.6.2. 2:1:1 dimeric species 21
3.6.3. Additional species 23
3.7. Binding constant 25
3.8. Diffusion ordered spectroscopy (DOSY) 27
4. Conformational Analysis 29
4.1. Computational Details 29
4.2. NOE analysis of conformers 34
4.2.1. TRIFP 1a/2a/HE 3b 35
4.2.2. [3:3] Dimer with OMe-CPA 1b 39
5. ^{1}H noesygpph vs ^{15}N hsqcetgpnosp 40
6. Appendix 43
6.1. Screening – ^{1}H- NMR Spectrum of each sample 43
6.2. NMR spectra of TRIFP 1a/2a/HE 3b 57
6.3. NMR spectra of TRIFP 1a/*E*-2a/HE 3b 63

6.4. NMR spectra of OMe-CPA 1b/2a/HE 3b 64
6.5. Pulse program ^{15}N hsqcetgpnosp 69
6.6. Pulse program ^{1}H noesygpph 73
7. Optimized Cartesian Coordinates of all the molecular complexes: 75
7.1. CPA 1a, *E*-imine 2a, HE 3b 75
7.1.1. Conformer C1 75
7.1.2. Conformer C2 80
7.1.3. Conformer C3 85
7.1.4. Conformer C4 90
7.2. CPA 1a, *Z*-imine 2a, HE 3b 93
7.2.1. Conformer C5. 93
7.2.2. Conformer C6 96
7.3. CPA 1b, *E*-imine 2a, HE 3b 99
7.3.1. Conformer 7 99
7.3.2. Conformer C8 102
7.3.3. Conformer C9 105
7.4. CPA 1b, *Z*-imine 2a, HE 3b 108
7.3.4. Conformer C10 108
7.5. [3:3] Dimer D1 111
7.6. Filtered out conformers. (SCF Energy only) 115
7.6.1. CPA 1a, E-imine 2a, HE 3b. (C101) 115
7.6.2. CPA 1a, Z-imine 2a, HE 3b (C102) 118
7.6.3. CPA 1a, Z-imine 2a, HE 3b. 120
References 134
Author Contributions 135

1. Analytical Methods

1.1. NMR Spectrometer Data

For all NMR spectroscopic measurements on model systems a Bruker Avance III HD 600 MHz spectrometer with a TBI (Triple resonance broadband inverse) 5 mm triple resonance broadband inverse probe with 19F-selective channel (TBI-F) with z gradient and BVT unit was used. The temperature of the spectrometer was regulated by a BVT 3900 unit and liquid nitrogen. Furthermore, samples for the characterization of in this work synthesized imines were measured on a Bruker III 400 MHz spectrometer equipped with 5 mm BBFO (BB/^{19}F, ^{1}H, ^{2}H) probe head with Z-Gradients. Spectrometer control and spectra processing was performed by Bruker Software TopSpin 3.2 PL 7 and Mestrenova® 14.3.3. For data processing, preparation and presentation Microsoft Excel, ChemBioDraw 19.0 and CorelDraw 2022.
Chemical shifts of ^{1}H and ^{13}C were referenced to TMS or the solvent. The heteronuclei ^{15}N and ^{31}P were referenced, employing ν(X) = ν(TMS) · Ξreference / 100 % according to Harris et al.[1] The following frequency ratios and reference compounds were used: Ξ(^{15}N) = 10.132912 (lq. NH_3) and Ξ(^{31}P) = 40.480742 (H_3PO_4).

1.2. Pulse Sequences and Parameters

Standard pulse sequences from the Bruker pulse sequence catalogue (zg, zg30, etc.) have been used. For all ^{1}H-NMR spectra a sweep width of 22 ppm with an offset at 10 ppm were used. For ^{31}P spectra the sweep width was 40 ppm with the offset at 5 ppm and for ^{15}N a sweep width of 508 ppm with the offset at 200 ppm was used. Furthermore, the following acquisition parameter have been used:
^{1}H-NMR: Pulse program zg30, Relaxation delay = 2.00 s, Acquisition time = 2.48 s, SW = 22 ppm, TD = 66 K, ns = 1 – 64;
^{13}C NMR: Pulse program: zgpg30, Relaxation delay = 2.00 s, Acquisition time = 0.80 s, SW = 270.0 ppm, TD = 66k, NS = 1k – 4k;
^{15}N NMR: Pulse program: zg30; Relaxation delay = 3.00 s, Acquisition time = 1.06 s; SW = 508 ppm, TD = 66 K; NS = 1K – 4K;
^{31}P-NMR: Pulse program: zgpg30; Relaxation delay = 3.00 s, Acquisition time = 4.5 s, SW = 40.0 ppm, TD = 66k, NS = 256 - 512;
2D-^{1}H,^{1}H NOESY: Pulse program: noesygpph; Relaxation delay = 4.00 s, mixing time (D8) = 80-300 ms; NS = 8-16, TD = 4096; increments = 512-1024;
2D-^{1}H,^{1}H COSY: Pulse program: cosygpqf; Relaxation delay = 5.00 s, NS = 4-16, TD = 4096; increments = 512;
2D-^{1}H,^{13}C HSQC: Pulse program: hsqcedetgpsisp2.3; Relaxation delay = 3.75 s, $^{1}J_{XH}$ = 145 Hz; NS = 8-12, TD = 4096; increments = 512;
2D-^{1}H,^{13}C HMBC: Pulse program: hmbcgplpndqf; Relaxation delay = 3.90 s, $^{1}J_{XH}$ = 145 Hz, J_{XH}(long range) = 10 Hz; NS = 14-22, TD = 4096; increments = 512 - 1024;
2D-^{1}H,^{31}P HMBC: Pulse program: inv4gplrndqf; Relaxation delay = 6.00 s, NS = 16, TD = 4096; increments = 256 - 512;
2D-^{1}H,^{15}N HMBC: Pulse program: inv4gplrndqf; Relaxation delay = 4.00 s, delay for evolution of long range couplings (D6) = 20.00 ms; NS = 8-10, TD = 4096; increments = 256;
^{1}H DOSY: Relaxation delay = 2.00 s, NS = 16-128, TD = 66k, increments = 20, Diffusion time delay = 45.0 ms, gradient strength 5-95% linear, gradient pulse: 1.0 – 1.3 ms;
2D-^{1}H,^{15}N HSQC-NOE: Pulse program: hsqcetgpnosp, Relaxation delay = 3.00 s, NS = 8, TD = 66k, increments = 20, Diffusion time delay = 45.0 ms, gradient strength 5-95% linear, gradient pulse: 2.9 – 7.5 ms.

2. Materials and Chemical

2.1. Solvents

Deuterated solvents were purchased from Deutero or Sigma Aldrich. Deuterated and non-deuterated dichloromethane (DCM) were refluxed over CaH_2 under Argon atmosphere and distilled and stored over activated molecular sieves (3Å).

2.2. Chemicals

(*R*)-TRIFP **1a**, (*R*)-OMe-CPA **1b**, TRIM **1c**, TRIP **1d**, TiPSY **1e** were purchased at abcr. All synthesized imines were stored in a desiccator over anhydrous $CaCl_2$ or inside a glove box. Chemical necessary for the synthesis of the ^{15}N-enriched Hantzsch Ester (HE) **3a** and Hantzsch Ester derivate **3b** were purchased from Sigma Aldrich and stored in the glovebox. The imine was synthesized from ^{15}N-enriched aniline and the respective acetophenone-derivate which were both purchased from Sigma Aldrich.

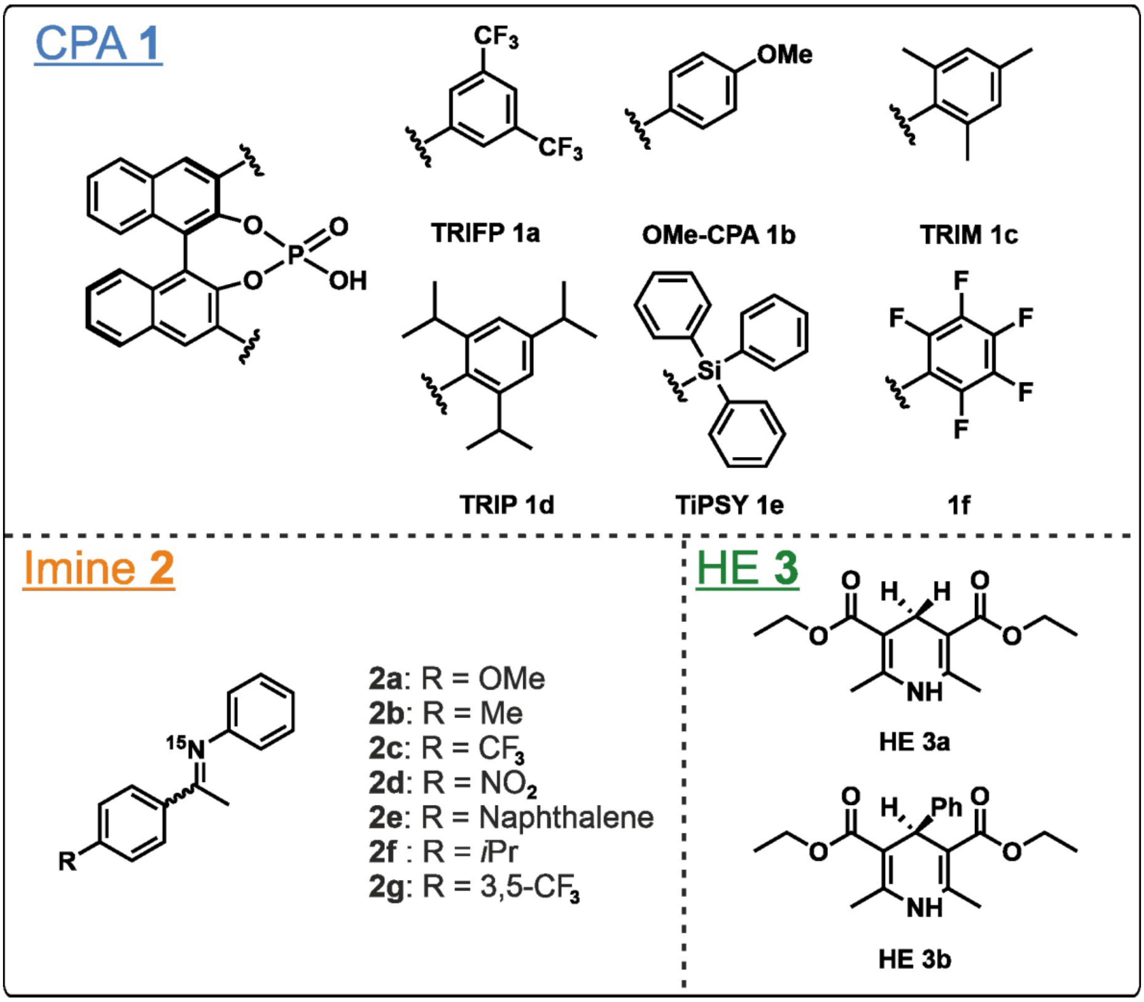

Figure S 1. Chiral Phosphoric Acids (CPAs) 1, imines 2, HE 3 used for NMR investigations in this study.

2.3. Synthesis of Imines

The imine was prepared as described in literature.[2] The characterization of the in this work synthesized imines was performed on Bruker Avance 400 MHz-Spectrometer with a 5mm BBO BB-1H/D probe head with Z-Gradients.

General Procedure for the synthesis of imines GP I [2,3]

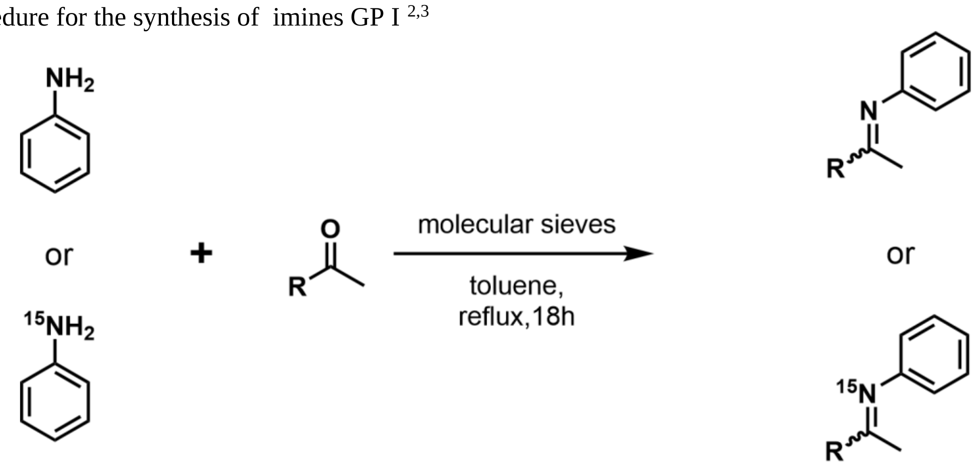

Molecular sieve (4 Å pore size, 3-5 g) was put into a 50 mL Schlenk flask and dried with a heat gun at 400°C for 30 min under reduced pressure. Aniline or Aniline (98% ^{15}N) (15.0 mmol, 1.40 g, 1.40 mL, 1.0 eq), the respective ketone (19.5 mmol, 1.3 eq.) and 25 mL anhydrous toluene were added under Argon flow. To this setup a reflux condenser was added under Argon flow and further flushed with Argon for 3 min. A drying tube, which was filled with $CaCl_2$, was attached to the reflux condenser and the solution was subsequently refluxed for 18 h. Afterwards, the heating bath was removed and the reaction mixture was allowed to cool down before being filtrated. Thereupon, the solvent was removed under reduced pressure to give a yellow solid, which was recrystallized in methanol 3-5 times to give the pure respective imine.
^{15}N-labeled Aniline was used for the synthesis to enable further investigations by NMR spectroscopy.
All substrates (**2a** – **2c**) were prepared according to literature following GP I. All NMR spectra match with the literature reports.[3–6] ^{15}N labeled imines were synthesized analogously and the NMR spectra matched with the literature reports.[4]

2.4. Synthesis of Hantzsch Ester derivate 3b

Hantzsch ester **3b** (^{15}N-diethyl 2,6-dimethyl-4-phenyl-1,4-dihydropyridine-3,5-dicarboxylate) was synthesized according to literature using ^{15}N-labeled $^{15}NH_3$ (aq.).[7]

Benzaldehyde (2.0 mL, 20 mmol), ethyl acetoacetate (5.0 mL, 40 mmol, 2 eq.) and $^{15}NH_3$ (14 M aq., 2.0 mL, 140 mmol, 7 eq.) were freshly distilled and subsequently stirred under reflux for 4 h. Then the mixture was cooled down and solidified. The resulting solid was dissolved in DCM (20 mL) and washed with saturated aq. NaCl (20 mL). After separation of the organic phase and drying with $MgSO_4$, the solvent was evaporated under reduced pressure, yielding crude HE **3b** as a yellow solid (~ 6 g, 91 % yield). Subsequently, the yellow solid was triturated with petroleum ether/EtOAc under reflux, filtered while hot, and washed with petroleum ether, resulting in the pure pale-yellow solid HE **3b** (67% yield after crystallization, >99% ^{15}N based on ^{1}H NMR integration).

^{1}H NMR (400 MHz, $CDCl_3$): δ 7.24 – 7.31 (m, 2H), 7.16 – 7.23 (m, 3H), 7.10 – 7.14 (m, 2H), 5.67 (d, $^{1}J_{NH}$ = 92.7 Hz), 4.99 (s, 1H), 4.08 (AB part of ABX_3, J_{AB} = 10.8 Hz, J_x = 7.1 Hz , 4H), 2.32 (d, $3J_{NH}$ = 3.0 Hz, 6H), 1.22 (X part of ABX_3, J_x = 7.1 Hz, 6H).

^{13}C NMR (101 MHz, $CDCl_3$): 167.6 (d, J = 3.0 Hz), 147.7 (CqAr), 143.8 (d, J = 12.0 Hz, Cq), 128.0 (CHAr), 127.8 (CHAr), 126.1 (CHAr), 104.2 (d, J = 1.7 Hz, Cq), 59.7 (CH2), 39.6 (d, J = 1.5 Hz, CH), 19.5 (d, J = 2.2 Hz, CH3), 14.2 (CH_3).

^{15}N NMR (41 MHz, $CDCl_3$): δ 131.8 (dsept, J = 92.8 Hz, 3.1 Hz).

3. NMR Investigations

3.1. Substrate Scope

Based on our previous studies on CPAs, we know that the electronic and steric properties of both CPA **1** and imine **2** play a crucial role for the spectral properties of the investigated system, especially the combination of the respective properties of CPA **1** and imine **2**. Therefore, we conducted a broad system screening of 25 different CPA/imine combinations to identify the combination of both which leads to the best NMR properties.

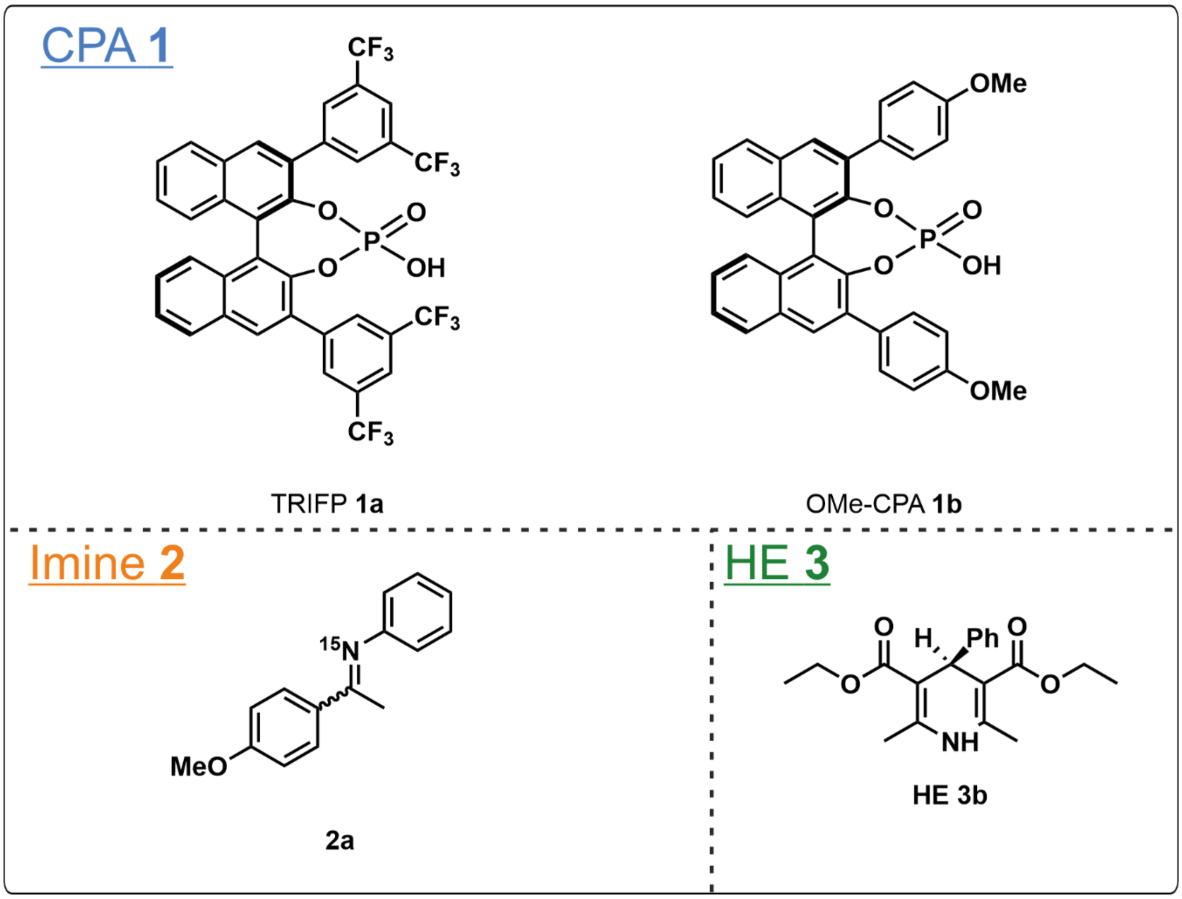

Figure S 2. CPAs 1, imine 2b and HE 3b used as model system substrates for NMR investigations in this study.

Table S1. List of investigated samples with selected model systems by NMR spectroscopy (600MHz, CD_2Cl_2, 180K).

Sample Nr.	System	Concentration CPA **1** [mM]	Concentration **2** [mM]	Concentration 3 [mM]
1	**1a•2a•3b**	40	40	40
2	**1a•2a•3b** *E-only*	40	40	40
3	**1a•2a•3b** *Z-enhanced*	40	40	40
4	**1a•2a•3b** *2:1 dimer*	100	50	50
5	**1b•2a•3b**	40	40	40
6	**1a•3b**	25	-	25
7	**2a•3b**	-	25	25
8	**1b**	25	-	-
9	**1b•2a**	40	40	-
10	**3b**	-	-	25

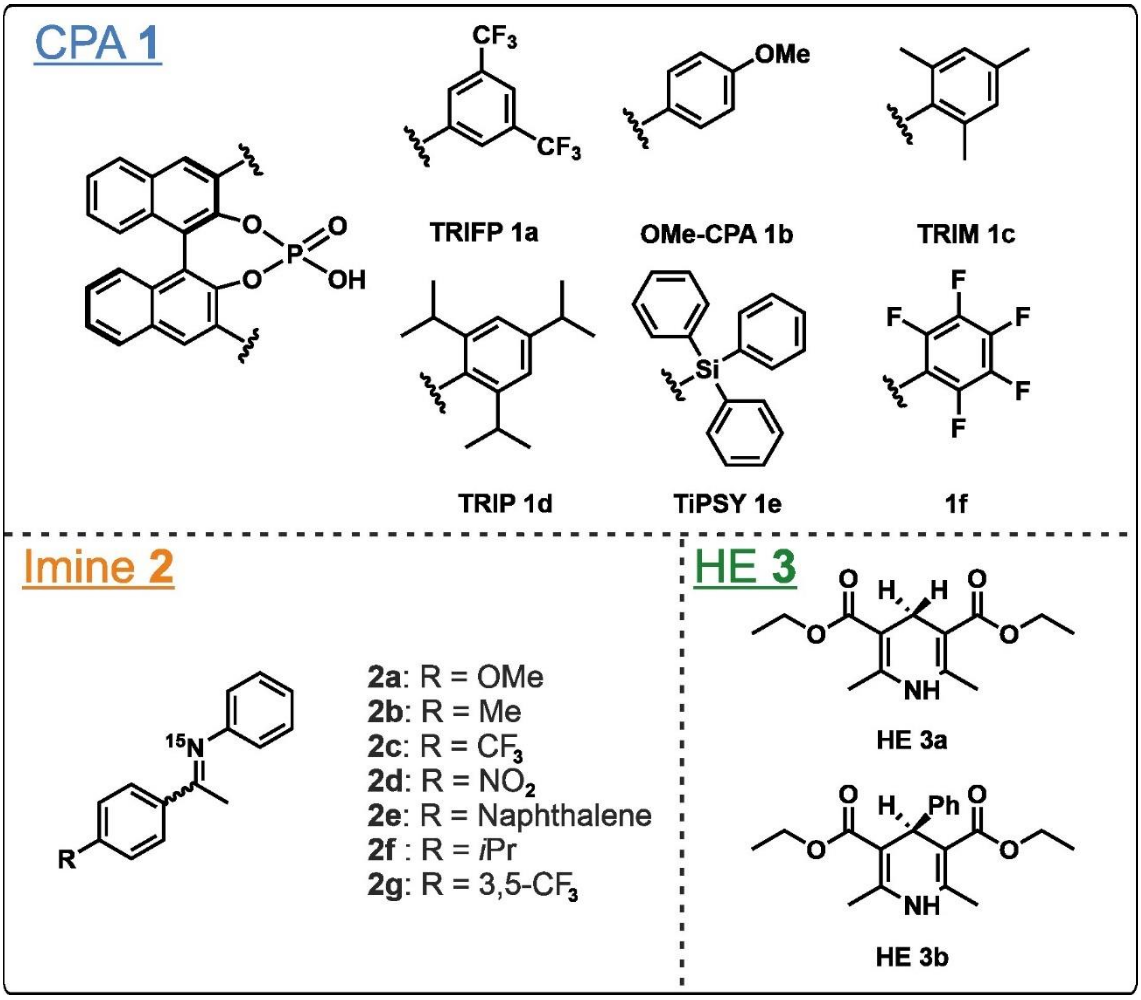

Figure S 3. CPAs 1, imines 2 and HE 3 used for screening by NMR spectroscopy.

Table S2. List of samples of all additionally screened systems to select the model systems for this study (1:1:1 stoichiometry, 600MHz, CD_2Cl_2, 180K).

Sample Nr.	System	Concentration CPA **1** [mM]	Concentration imine **2** [mM]	Concentration HE **3** [mM]
11	**1a•2a•3b**	40	40	40
12	**1a•2b•3b**	40	40	40
13	**1a•2c•3b**	40	40	40
14	**1a•2d•3b**	40	40	40
15	**1a•2e•3b**	40	40	40
16	**1a•2f•3b**	40	40	40
17	**1a•2g•3b**	40	40	40
18	**1b•2a•3b**	40	40	40
19	**1b•2b•3b**	40	40	40
20	**1b•2c•3b**	40	40	40
21	**1b•2d•3b**	40	40	40
22	**1c•2a•3b**	40	40	40
23	**1c•2b•3b**	40	40	40
24	**1c•2c•3b**	40	40	40

25	**1c•2d•3b**	40	40	40
26	**1c•2e•3b**	40	40	40
27	**1d•2a•3b**	40	40	40
28	**1d•2b•3b**	40	40	40
29	**1d•2c•3b**	40	40	40
30	**1d•2e•3b**	40	40	40
31	**1d•2f•3b**	40	40	40
32	**1e•2b•3b**	40	40	40
33	**1e•2c•3b**	40	40	40
34	**1e•2e•3b**	40	40	40
35	**1f•2a•3b**	40	40	40

3.2. General Procedure for Sample Preparation and NMR-Measurements (GPSP)

One stock solution each was prepared for the ^{15}N-labeled imine **2** (50 mM) and ^{15}N-labeled Hantzsch Ester **3** (50 mM) in dry CD_2Cl_2 (*d*-DCM). The CPA **1** (25-50 mM, 1-2 eq.) was directly weighed in an NMR tube, which was subsequently dried with a heat gun at 140 °C for 30 min under reduced pressure (CPAs are often delivered with residual moisture, thus the heating process is necessary to remove remaining traces of water). After heating the tube was cooled down to room temperature and evacuated and flushed with Argon three times. Out of each stock solution 0.3 mL were added to the NMR tube as well as TMS atmosphere as reference (0.5 mL). Thereupon, the tube was closed and sealed with parafilm and stored in the fridge at -80 °C or used immediately.

All samples used in this work were prepared following this procedure and are listed in Table S1 and Table S2.

3.2.1. *E-only samples*

A stock solution with the ^{15}N-labeled imine **2** (50 mM) in dry CD_2Cl_2 (*d*-DCM) was prepared and cooled down to -80°C. Another stock solution for the ^{15}N-labeled **3** (50 mM) in dry CD_2Cl_2 (*d*-DCM) was prepared and also cooled down to -80°C. The CPA **1** (25 mM) was directly weighed in an NMR tube, which was subsequently dried with a heat gun at 140 °C for 30 min under reduced pressure. The tube was evacuated and flushed with Argon three times and also cooled down to -80°C. Out of each stock solution 0.3 mL were taken and added to the pre-cooled NMR tube. In addition, TMS atmosphere (0.5 mL) was added to the NMR tube as a reference. Thereupon, the tube was closed and sealed with parafilm under continuous cooling and immediately investigated by NMR spectroscopy at -80°C.

3.3. Chemical Shift Assignment

For any structural investigation a chemical shift assignment is necessary first. Due to the complexity, chemical exchange and overlapping of all present species in a CPA/imine/HE sample, we measured ^{1}H,^{1}H-COSY, ^{1}H,^{1}H-TOCSY, ^{1}H,^{1}H-NOESY, ^{1}H,^{13}C-HSQC, ^{1}H,^{13}C-HMBC, ^{1}H,^{15}N-HMBC, ^{1}H,^{31}P-HMBC, ^{1}H,^{19}F-HOESY, ^{15}N-HSQC-NOESY, 1D selective NOESY and all corresponding 1D spectra (^{1}H, ^{13}C, ^{15}N, ^{19}F, ^{31}P) for both TRIFP **1a**/**2a**/HE **3b** and OMe-CPA **1b**/**2a**/HE **3b**. Furthermore, both imine **2** and HE **3b** were ^{15}N-labeled. All of these spectra were necessary to gather as much information as possible about each sample. However, due to fast exchange of binary and ternary complexes on the NMR timescale even at 180 K, only an averaged signal set for CPA and imine is detected, while the HE signals can be unambiguously assigned to the ternary complex. In this chapter, the general assignment strategy is exemplarily discussed for TRIFP **1a**/*E*-**2a**/HE **3b**.

3.3.1. Assignment strategy

Normally, ^{1}H,^{31}P-HMBC spectra are used as a starting point for any chemical shift assignment of CPA/imine complexes due to the easily accessible information about the center of the complexes that can be obtained. However, due to various exchange processes within and in between ternary and binary complexes, the ^{31}P signals appear very broad and overlapped. Hence, in the ^{1}H,^{31}P-HMBC no signals at all can be observed anymore. Besides ^{31}P, ^{15}N can also be used to obtain information about the hydrogen bonds. The two ^{1}H-signals of the hydrogen-bonded proton I1 were assigned to the *E*- and *Z*-complexes based on prior studies and known NOE contacts with the CPA.[4,7,8] Based on this assignment, the respective ^{15}N chemical shift of the nitrogen atoms were identified with the ^{1}H,^{15}N-HMBC spectrum. However, in the ^{1}H,^{15}N-HMBC only very few cross signals can be observed out of which only one can be unambiguously assigned to I2 (see Fig S4). Additionally, one cross signal for the hydrogen bond with HE **3b** is observed to a proton at 1.76 ppm. However, as no reference is available it cannot be differentiated between HE 2 and HE 7 yet.

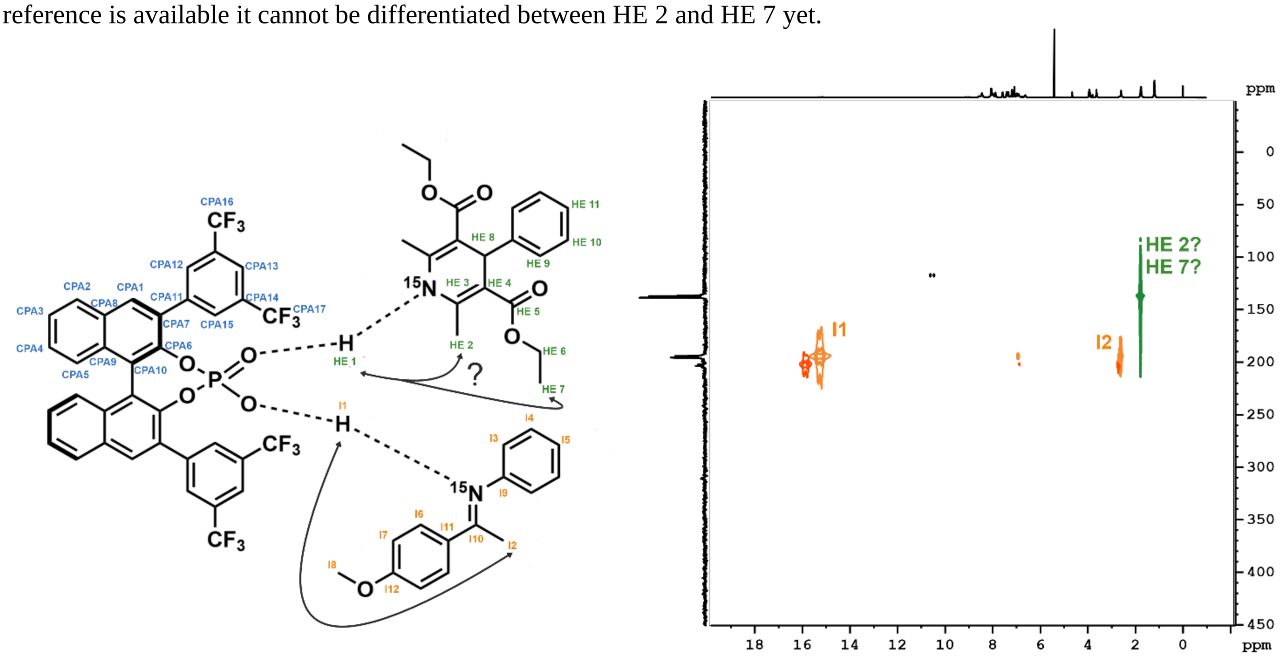

Figure S 4. ^{1}H,^{15}N-HMBC of TRIFP 1a/2a/HE 3b (right, 1:1:1 stoichiometry, 40 mM, 600MHz, CD_2Cl_2, 180K) depicting the cross signals with I 2 and either HE 2 or HE 7. TRIFP 1a/*E*-2a is marked in orange, TRIFP 1a/*Z*-2a is marked in red and TRIFP 1a//HE 3b is marked in green. The structure with corresponding ^{1}H,^{15}N-HMBC relations (arrows) is displayed on the left.

TRIFP **1a** systems represent a unique scenario in which ^{1}H,^{19}F-HOESY spectra can be employed which provide further insights (see Fig S5). In the ^{1}H,^{19}F-HOESY all surrounding hydrogen atoms of the 3,3´-substituents can be observed. Here, the same signals as in the ^{1}H,^{15}N-HMBC are detected again (2.60 ppm for I2, 1.76 ppm for HE 2 or HE 7). In addition, signals with a ^{1}H chemical shift of 1.19 ppm, 3.64 ppm, 3.93 ppm and 4.66 ppm are observed. With this information both CH_3-groups of the HE **3b** can be assigned by combining these observations with ^{1}H,^{1}H-COSY data, which also confirmed the signal at 3.93 ppm as HE 6. Subsequently, the signal at 3.64 ppm can be assigned to the methoxy-group I8 of the imine, which is confirmed by ^{1}H,^{13}C-HMBC. Furthermore, a ^{1}H chemical shift of 4.66 ppm is assumed to correspond to HE 8 after assigning I8 and HE6. This assumption is proven by NOE correlation to the already assigned HE 2, HE 6, HE 7 and later also to the phenyl-ring of HE **3b** (HE 9-11). Also, first information about the CPA can be obtained. Based on the intensity of the signals at 7.86 ppm and 8.45 ppm these signals should correspond to CPA 12 and CPA 13 respectively as

smaller distances are depicted as higher intensity in NOESY/HOESY spectra. For both signals no COSY relations can be observed, supporting the initial assumption. In addition, ^{1}H,^{1}H-NOESY cross signals between both are observed but only for 7.86 ppm cross signals to other aromatic protons are detected. Combining ^{1}H,^{1}H-COSY, ^{1}H,^{1}H-TOCSY, ^{1}H,^{13}C-HSQC, ^{1}H,^{13}C-HMBC with these NOE correlations confirms the suggested assignment of CPA 12 (7.86 ppm) and CPA 13 (8.45 ppm). As a result of this assignment, a network of signals can be examined by 2D NMR spectra which can only be related to the BINOL-backbone additionally supporting the assignment of CPA 12 and CPA 13 and lead to a full assignment of all hydrogen atoms of the CPA. The same can be achieved for the imine **2a** and the HE **3b** from the assigned signals. For the imine **2a**, the ketone-part can be fully assigned using ^{1}H,^{13}C-HMBC and ^{1}H,^{1}H-NOESY cross signals of the methoxy-group I8. The remaining *N*-phenyl moiety can then be assigned based on ^{1}H,^{13}C-HSQC, ^{1}H,^{13}C-HMBC and ^{1}H,^{1}H-NOESY cross signals with I2. To double check the assignment ^{1}H,^{1}H-COSY and ^{1}H,^{1}H-TOCSY are considered. For HE **3b** only the phenyl-ring is not assigned yet which can also be achieved by ^{1}H,^{13}C-HSQC, ^{1}H,^{13}C-HMBC and ^{1}H,^{1}H-NOESY cross signals with HE 3-8. All quaternary carbons can be assigned by ^{1}H,^{13}C-HSQC, ^{1}H,^{13}C-HMBC from the surrounding assigned protons/carbons. Then, HE 8 can finally be unambiguously confirmed by ^{1}H,^{13}C-HMBC correlations with HE 3 and HE 4. Hence, starting with ^{1}H,^{19}F-HOESY enables an assignment of all three substances: CPA, imine and HE.

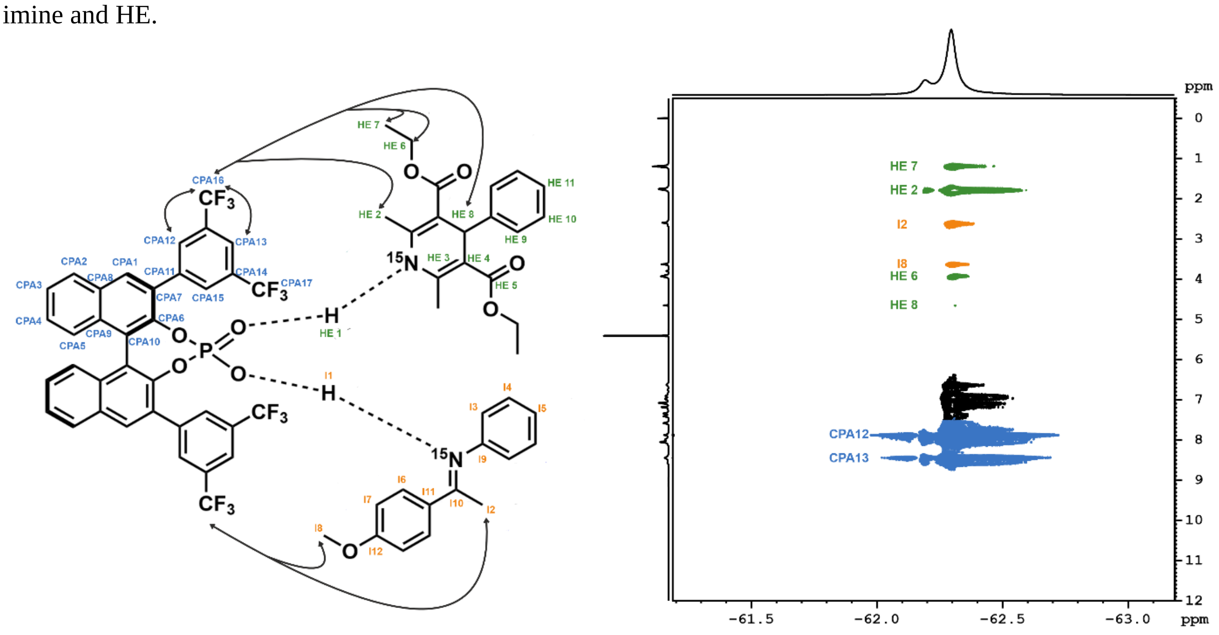

Figure S 5. ^{1}H,^{19}F-HOESY of TRIFP 1a/2a/HE 3b (right, 1:1:1 stoichiometry, 40 mM, 600MHz, CD_2Cl_2, 180K) depicting the cross signals of the CF^{3}-groups of TRIFP 1a. Cross signals to TRIFP 1a are marked in orange, cross signals to 2a are marked in orange, cross signals to 3b are marked in green. The structure with corresponding ^{1}H,^{19}F-HOESY relations (arrows) is displayed on the left.

However, if ^{1}H,^{19}F-HOESY spectra are not accessible which is the case for all other commonly used CPAs, such as TRIM **1c** (3,3'-bis(2,4,6-trimethylphenyl)-1,1'-binaphthyl2,2'diyl hydrogen phosphate), TRIP **1d** (3,3'-bis(2,4,6-triisopropylphenyl)-1,1'-binaphthyl-2,2'diyl hydrogen phosphate), and TiPSY **1e** (3,3'- bis(triphenylsilyl)-1,1'-binaphthyl-2,2'diyl, this assignment strategy is not possible. Therefore, we applied ^{15}N-HSQC-NOESY to get a new starting point for the assignment of each substrate. This method even derives significantly more information about all three substrates than ^{1}H,^{19}F-HOESY when examining the individual rows of each ^{15}N-chemical shift separately (see Fig S6). Again, HE 2 and HE 6-8 are observed which can be assigned similarly as in the ^{1}H,^{19}F-HOESY assignment strategy. The remaining HE **3b** signals can then also be assigned by combining ^{1}H,^{1}H-COSY, ^{1}H,^{1}H-TOCSY, ^{1}H,^{13}C-HSQC, ^{1}H,^{13}C-HMBC and ^{1}H,^{1}H-NOESY as described above. For imine **2a**, different signals can be observed. In contrast to ^{1}H,^{19}F-HOESY, the methoxy-group I8 is not detected. However, most other imine signals can be directly observed by ^{15}N-HSQC-NOESY. Checking and combining the information derived by ^{1}H,^{1}H-COSY and ^{1}H,^{1}H-TOCSY spectra for these signals, leads to an unambiguous assignment of the aromatic protons of the imine, which is further supported by ^{1}H,^{13}C-HSQC, ^{1}H,^{13}C-HMBC and ^{1}H,^{1}H-NOESY. Subsequently, the methoxy-group I8 can be assigned based on ^{1}H,^{1}H-NOESY cross signals to I7. The assignment of CPA 12 and CPA 13 of TRIFP **1a** is similar to the above described assignment strategy as the ^{15}N-HSQC-NOESY results in the same information as the ^{1}H,^{19}F-HOESY and enables the full assignment of the BINOL-backbone. For other CPAs, depending on the 3,3´-substituent, different assignment strategies have to be employed. For example, OMe-CPA **1b** has a distinct methoxy-group at the 3,3´-substituent which shows cross signals to the CPA/imine nitrogen leading to a starting point for the CPA assignment.

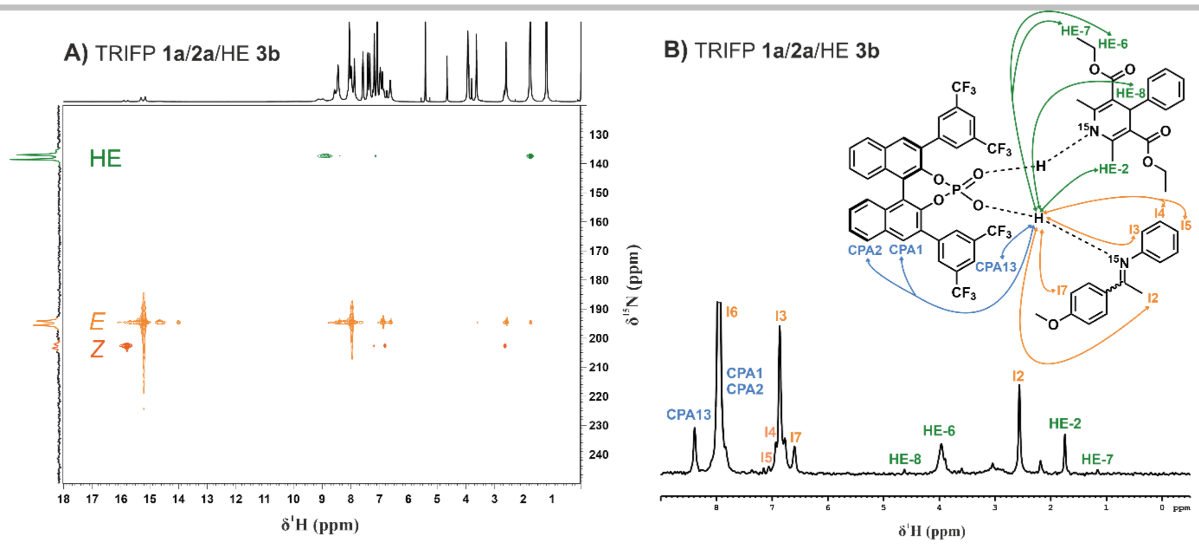

Figure S 6. **^{15}N-HSQC-NOESY spectrum of TRIFP 1a/2a/ HE 3b (1:1:1 stoichiometry, 40 mM, CD_2Cl_2, 180K). Separated rows for the TRIFP 1a/*E*-2a (orange), TRIFP 1a/*Z*-2a (red) and TRIFP 1a/HE 3b (green) hydrogen bonds can be observed. B) Single rows can be displayed as 1D spectra for more detailed information to reveal the structural environment of each hydrogen bond, here exemplary displayed for the TRIFP 1a/*E*-2a hydrogen bond. Cross signals to TRIFP 1a are marked in orange, cross signals to 2a are marked in orange, cross signals to 3b are marked in green.**

3.3.2. TRIFP 1a/E-2a/HE 3b assignment

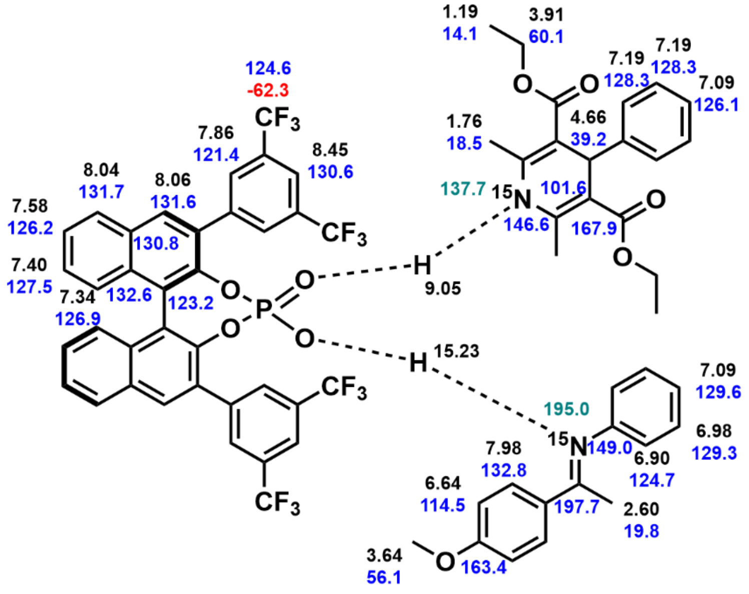

3.3.3. OMe-CPA 1b/E-2a/HE 3b assignment

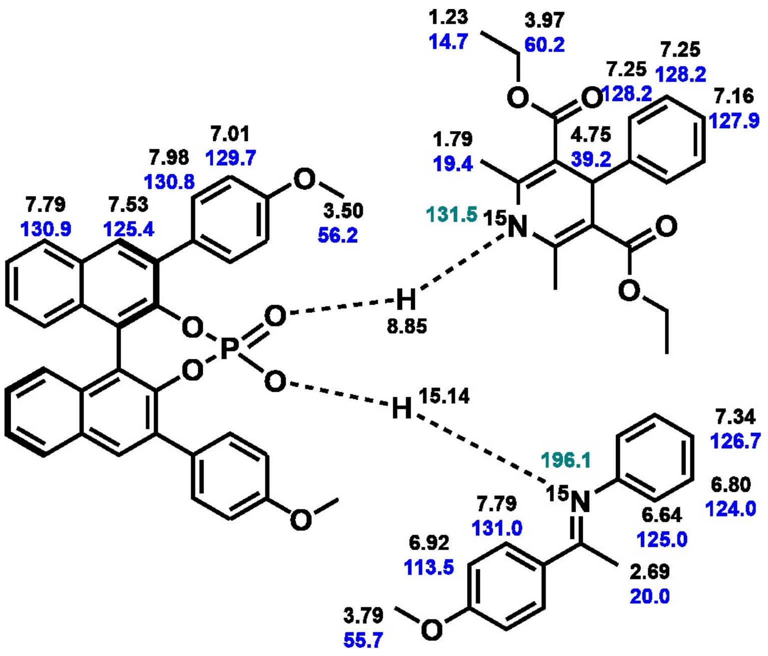

3.3.4. OMe-CPA 1b /Z-2a/HE 3b assignment

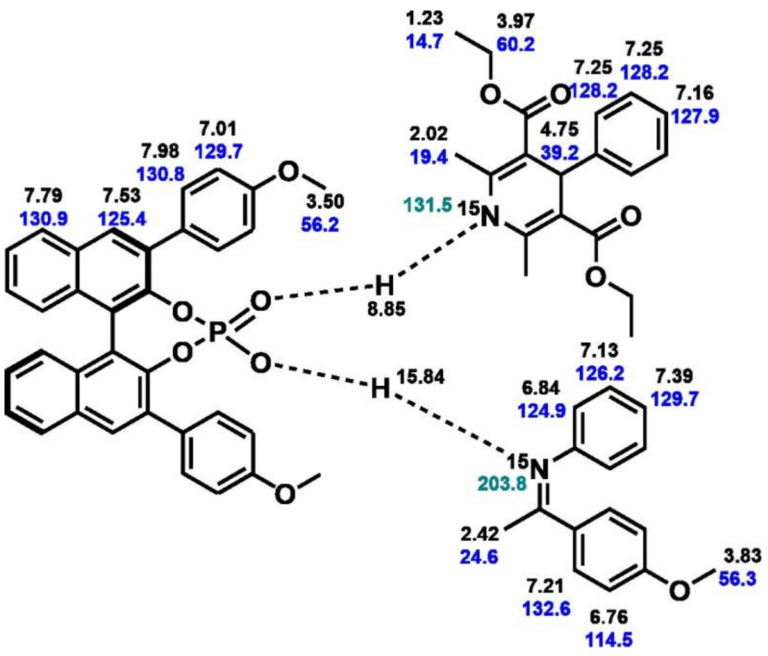

3.4. Exclusion of other species

To confirm the association of the hydrogen bond signals of the ternary complex and exclude other possibilities, separate samples containing TRIFP **1a**/HE **3b** or **2a**/HE **3b** were evaluated and compared (see Fig S7 and Fig S8).
First, HE **3b** was measured alone. Here, the NH-hydrogen can be seen at 6.71 ppm (green). After addition of the imine **2a** this signal shifts slightly to 7.03 ppm (green). Upon further addition of the CPA (TRIFP **1a**) the signal shifts significantly to 9.05 ppm (green). Similarly, a shift of the aliphatic signals of HE **3b** can be observed. HE-1 (skin tone) is shifting from HE **3b** solo at 4.03 ppm to 3.93 ppm in the ternary complex. HE-2 (pink) is shifting from HE **3b** solo at 1.26 ppm to 1.19 ppm in the ternary complex. HE-3 (orange) is shifting from HE **3b** solo at 2.30 ppm to 1.76 ppm in the ternary complex. Hence, HE-3 shifts further in highfield direction in comparison to HE-1 and HE-2 which is corresponding with the distance to the hydrogen bonded NH. For both the HE **3b** and **2a**/HE **3b** sample, a different set of chemical shifts compared to the ternary complex can be observed. Also, the CPA/imine/HE hydrogen bond signal for HE **3b** is displayed as a doublet when both substances are ^{15}N labeled, or as a singlet when only the imine is ^{15}N labeled, indicating that the imine is not participating. Therefore, an imine/HE complex contribution can be excluded for the ternary complex similar to the DSIs.

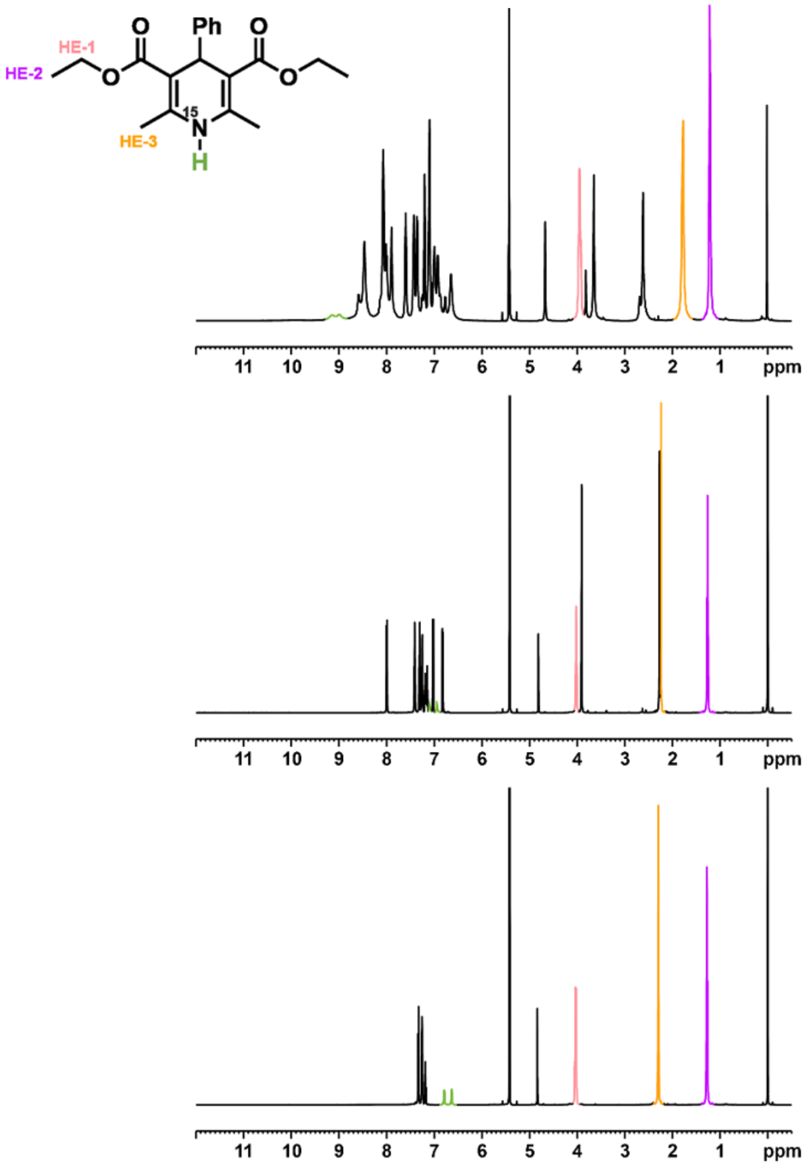

Figure S 7. Comparison of different samples to validify the CPA/HE hydrogen bond of the ternary complex. Top: TRIFP 1a/2a/HE 3b (1:1:1 stoichiometry, 40 mM, 600MHz, CD_2Cl_2, 180K). Mid: 2a/HE 3b (1:1 stoichiometry, 25 mM, 600MHz, CD_2Cl_2, 180K). Bottom: HE 3b (25 mM, 600MHz, CD_2Cl_2, 180K). The NH hydrogen atom of the HE 3b is marked in green. In addition, the aliphatic signals of HE 3b are also coloured corresponding to the molecule shown on the top left.

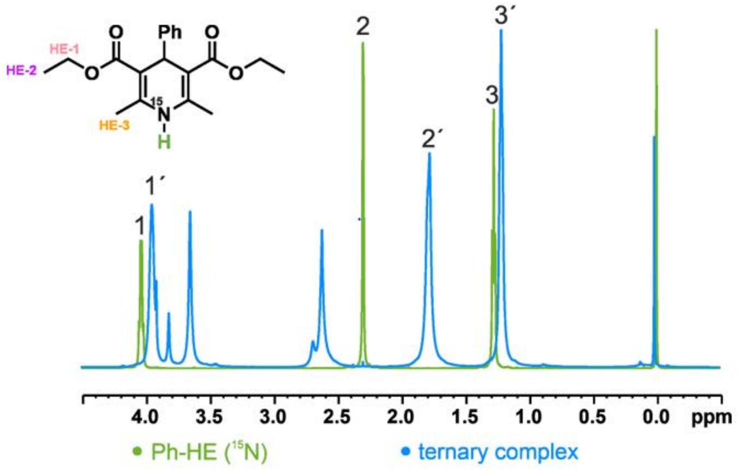

Figure S 8. Comparison of the aliphatic signals of HE 3b when measured alone (green, 25 mM, 600MHz, CD_2Cl_2, 180K) and in the ternary complex of TRIFP 1a/2a/HE 3b (blue, 1:1:1 stoichiometry, 40 mM, 600MHz, CD_2Cl_2, 180K). A highfield shift can be observed for all signals upon addition of TRIFP 1a and 2a, which is even more pronounced when the respective hydrogen atom is closer to the hydrogen bonded NH.

In addition, a sample with TRIFP **1a** and HE **3b** was investigated to exclude a simple CPA/HE species being responsible for the observed hydrogen bond signals of the HE **3b**. Here, a doublet was detected at 6.37 ppm which vanishes upon addition of the imine to form the ternary complex (see Figure S9). The CPA/HE hydrogen bond of the ternary complex is not observed in the TRIFP **1a**/HE **3b** sample. This additionally supports, that a CPA/HE species can be excluded for the signal at 9.05 ppm.

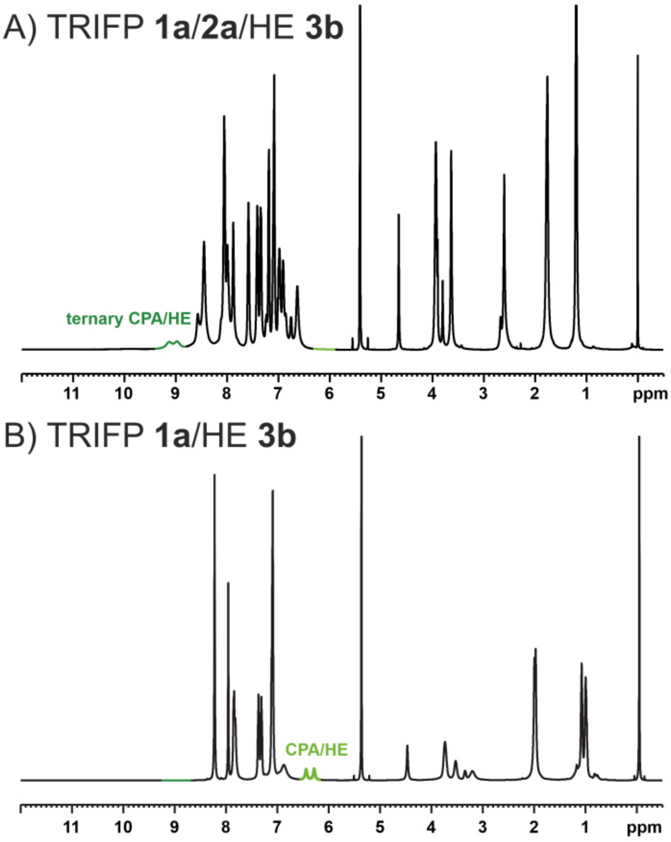

Figure S 9. Comparison of different samples to validify the CPA/HE hydrogen bond of the ternary complex. A) TRIFP 1a/2a/HE 3b (1:1:1 stoichiometry, 40 mM, 600MHz, CD_2Cl_2, 180K). B): TRIFP 1a/HE 3b (1:1, 40 mM, 600MHz, CD_2Cl_2, 180K). The CPA/HE hydrogen bond of the ternary complex does not appear in the other sample, excluding a simple CPA/HE species. Also, the observed hydrogen bond signal in B) vanishes in A), excluding a contribution to the NOE correlations.

Additionally, to rule out the possibility that the Overhauser effects originate from binary complexes (CPA/HE or imine/HE), we investigated the respective 1:1 mixtures. The combination of TRIFP **1a** and Hantzsch ester **3b** showed a shift of the NH-group of the HE **3b** to 6.37 ppm in comparison to a sample with only the HE **3b**. This signal of the TRIFP **1a**/HE **3b** complex vanished upon the addition of imine in a 1:1:1 mixture (see Fig 9A). Therefore, the binary complex of TRIFP **1a** and HE **3b** is in slow exchange with the ternary complex, and the corresponding binding constant for ternary complex formation is significantly higher than the binding of the Hantzsch ester to the catalyst, similarly to DSI complexes.[7] Thus, any contribution to the Overhauser effects in the ternary complex can be unambiguously eliminated.

The formation of the complex between imine **2a** and HE **3b** in the ternary complex mixture is improbable due to the considerably higher acidity of the CPA. Indeed, a 1:1 mixture of imine **2a** and HE **3b** exhibited sharp ^{1}H-NMR signals with a distinct set of chemical shifts compared to the ternary complex. Once again, this rules out the contribution of the imine/HE complex to the intermolecular NOEs observed in the ternary mixtures.

As stated above, the ternary complexes are in fast exchange with the binary complexes, in which rotation can occur due to the large binding pocket of the catalyst, even with intact hydrogen bonds.[9] Even within CPA complexes featuring strong hydrogen bonds, hydrogen bond switching can take place, which is fast on the NMR timescale. To eliminate any potential impact of these processes in the binary complexes on the structure determination of the ternary complex, interactions exclusively with the Hantzsch ester were interpreted.

3.5. General features of ternary complex

CPA **1**: All CPAs are symmetric along the C2 axis, only the 3,3'-substituents are not symmetric and have distinct signals for the aromatic protons, carbons or e.g. for TRIFP **1a** two distinct 3,5-CF_3 groups due to the restricted rotation along the main substituent axis.

Imine **2**: For the CPA/imine hydrogen bond one set of signals for *E*-complex and one set of signals for Z-complex can be observed. In contrast to the binary complex, the CPA/imine hydrogen bond signals are slightly highfield shifted but no separate hydrogen bond signal for the ternary complex is detected. This indicates a fast exchange between both complexes leading to one signal set. Furthermore, according to the Steiner-Limbach curve and the results of the binary complex, this highfield shift of the hydrogen bonds reveal a weaker hydrogen bond and hence, a stronger ion-pair character.[8] The additional chemical exchange leads to worse line broadening and worse resolution of ^{15}N- and ^{31}P-NMR spectra for the ternary complex samples in comparison to the binary complex samples to the point that no ^{1}H,^{31}P-HMBC signals can be detected and only a few ^{1}H,^{15}N-HMBC signals are observed. Both imine isomers show NOE correlations to the HE **3b**, which are used to analyze the conformers. However, only for the *E*-isomer species enough signals are detected for a full chemical shift assignment and conformer analysis. Despite of sharper ^{1}H signals of the *Z*-isomer species, which should benefit 2D spectra, due to the lower population only a few signals can be unambiguously identified which hinders deeper analysis of the *Z*-species.

HE **3b**: For the CPA/HE hydrogen-bonded species only one set of signals is observed. The ternary complex is confirmed by ^{15}N-HSQC-NOESY spectra in which NOE correlations between the CPA/imine hydrogen bond and HE **3b** are detected. The same is observed for the CPA/HE hydrogen bond to imine **2a**.

3.5.1. Binary vs. ternary complex

In the hydrogen bond area of the ternary complex, only one set of signals for the CPA/imine hydrogen bond is detected. This indicates a fast exchange between binary and ternary complex, as otherwise two separate signal sets would have to be observed in the 1:1:1 sample of the ternary complex. This is supported by a chemical shift mapping of these signal upon addition of the HE **3b** to the binary complex (see Fig S17).
Comparing the CPA/imine hydrogen bonds of the binary and ternary complex, reveals a highfield shift of the hydrogen bonds upon addition of the HE **3b** (see Fig S10). Exemplary for TRIFP **1a**/**2a**/HE **3b**, for the CPA/*Z*-imine hydrogen bond a highfield shift from 16.11 ppm (binary complex) to 15.84 ppm (ternary complex) is observed, while for the CPA/*E*-imine hydrogen bond a highfield shift from 15.39 ppm (binary complex) to 15.23 ppm (ternary complex) is observed. In general, based on the Steiner-Limbach curve this indicates a weakening of the hydrogen bond upon addition of the HE.[8]

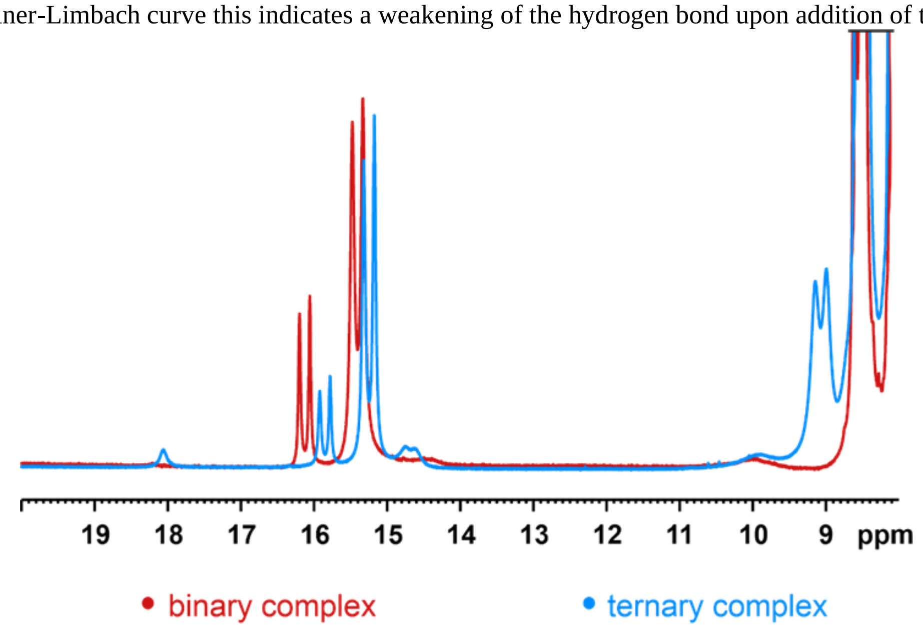

Figure S 10. Comparison of the binary complex with TRIFP 1a/2a (red; 1:1 stoichiometry, 40mM) and the ternary complex (blue; 1:1:1 stoichiometry, 40 mM, 600MHz, CD_2Cl_2, 180K).

3.6. Structural Space

Besides the ternary complex, other hydrogen-bonded species can be observed in the hydrogen bond area of the ^{1}H-spectrum of all screened systems, which vary in population depending on the system (see Figure S11).

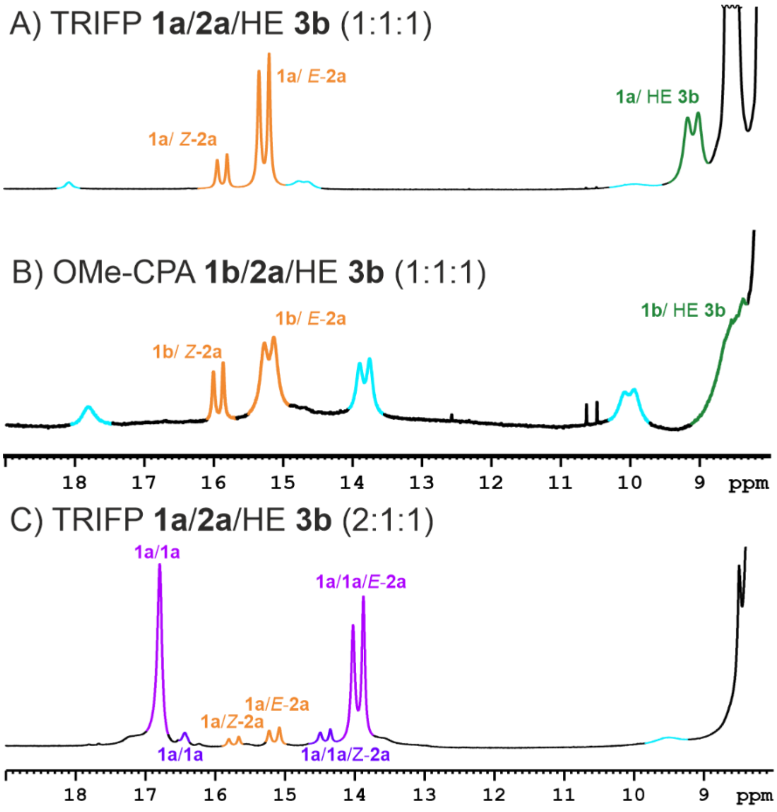

Figure S 11. A) ^{1}H-spectrum of the ternary complex of TRIFP 1a/2a/HE 3b (1:1:1 stoichiometry, 600MHz, CD_2Cl_2, 180K). All additional hydrogen bond signals are colored in light blue. B) ^{1}H-spectrum of the ternary complex of TRIFP 1b/2a/HE 3b (1:1:1 stoichiometry, 600MHz, CD_2Cl_2, 180K). All additional hydrogen bond signals are colored in light blue (see SI 3.6.1). C) ^{1}H-spectrum of the of TRIFP 1a/2a/HE 3b in a 2:1:1 stoichiometry (stoichiometry, 600MHz, CD_2Cl_2, 180K). The dimeric species is marked in pink (CPA/CPA/*E*-imine) or purple (CPA/CPA/*Z*-imine), with each having one singlet for the CPA/CPA hydrogen bond and one doublet for the CPA/CPA/imine hydrogen bond, which are in a 1:1 integral ratio (see SI 3.6.2).

3.6.1. [3:3] dimer

Next to the hydrogen bond signals of the ternary complex (orange), an additional marginal populated hydrogen-bonded species (blue) can be observed in the TRIFP **1a**/**2a**/HE **3b** system (see Fig S9). Here, their contribution to NOESY spectra can be neglected due to their extremely low population even compared to the *Z*-imine species, for which a NOE analysis was already only partly possible. These signals are higher in population in the second model system of OMe-CPA **1b**/**2a**/HE **3b** and can also be observed in various other CPA/imine/HE systems. A total of three additional hydrogen bond signals can be seen, one singlet and two doublets.
Further investigations were deducted to investigate the origin of these unknown signals. Comparing all systems of TRIFP **1a** with several different imines **2a-g** revealed that the low-field shifted singlet (18.04 ppm) stays at the same chemical shift in all system. Only upon using a different CPA, the singlet shifts indicating its dependency solely on the CPA. Noteably, this signal only appears upon addition of the imine, which might be attributed to an interplay of stabilization between the different species.[10] Given the data and limited experimental access to this species, we assume that this system stems from a phosphoric acid aggregate.

For further investigations of the new hydrogen bond signals observed in the ^{1}H-spectra samples, the OMe-CPA **1b**/**2a**/HE **3b** system was used due to a higher population of these hydrogen bond species in comparison to TRIFP **1a**/**2a**/HE **3b** (see Fig S12). Samples with either a ^{15}N-labeled or unlabeled imine **2a** were measured to reveal the respective species. Here it was shown that the doublet at 13.76 ppm is corresponding to an [CPA/imine] **2a** hydrogen bond while the doublet at 9.99 ppm is related to a [CPA/HE **3b**] species (see Fig S12).

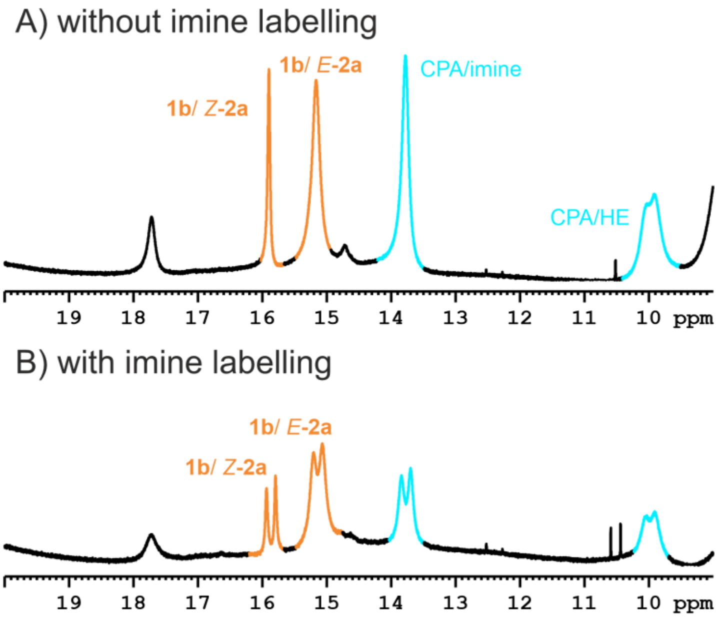

Figure S 12. A) ^{1}H-spectrum of the ternary complex of 1b/2a/HE 3b. Here, imine 2a is not ^{15}N-labeled, HE 3b is ^{15}N-labeled (1:1:1 stoichiometry, 600MHz, CD_2Cl_2, 180K). B) ^{1}H-spectrum of the ternary complex of 1b/2a/HE 3b. Both imine 2a and HE 3b are ^{15}N-labeled (1:1:1 stoichiometry, 600MHz, CD_2Cl_2, 180K).

Although a separate set of hydrogen bonds is detected, the CPA **1b** and imine **2a** signals have an averaged signal set with the binary and ternary complexes due to exchange processes. This indicates a locked system similar to the previously reported [2:2] dimeric species consisting of two binary complexes.[11] Also, for the [2:2] dimer, the signals were characteristically more high-field shifted than the CPA/imine hydrogen bonds of the binary complex similarly to the newly observed CPA/imine hydrogen bond signal. In addition, the integral ratios of the hydrogen bond signals in the ^{1}H-NMR spectrum gave a ratio of 1:1 of the CPA/imine signal to the CPA/HE signal. From this a first hypothesis of a [**3**:**3**] dimeric species consisting of two ternary complexes (CPA/imine/HE=**3**) was developed.
This [3:3] dimer was then further investigated by dilution experiments and diffusion ordered spectroscopy (DOSY). Diluting a 40 mM sample to a 20mM or a 10 mM sample demonstrated that these signals are corresponding to a dimeric species and again showed that both doublets belong to the same species as both were diminishing at the same rate (see Fig S13).
In contrast, the relative population of the 1b/*E*-2a/3b hydrogen bond signal of the ternary complex is increasing with smaller concentration. This observation can be explained by the decrease of the [3:3] dimer which releases *E*-imine complexes only. Hence, the relative population of the *E*-imine species of the ternary complex is increasing in comparison to the other species.

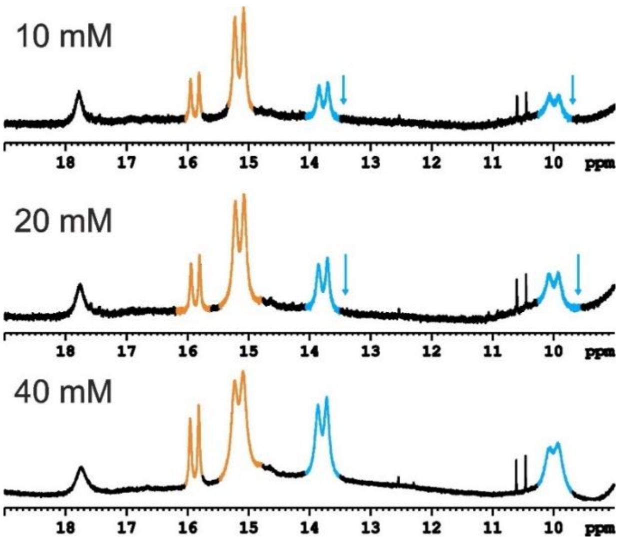

	Relative Integrals				
Concentration / mM	**1b/1b**	*Z*-2a•1b•He 3b	*E*-2a•1b•3b	[*E*-2a•1b•HE 3b]$_2$	Sum
40	0.091 ↑	0.18 ↑	0.3 ↓	0.43 ↑	1.00
20	0.089	0.14	0.55	0.22	1.00
10	0.081	0.12	0.65	0.15	1.00

The arrows in the table indicate wheter the relative integral increases or decreases with increasing concentration

Figure S 13. ^{1}H-spectrum of the ternary complex of 1b/2a/HE 3b (1:1:1 stoichiometry, 600MHz, CD_2Cl_2, 180K). The sample was diluted from bottom to top starting at 40 mM to 20 mM to 10 mM. The signals of the [3:3] dimeric species (blue) diminished each time in comparison to the ternary/binary complex hydrogen bond signal indicating a dimeric species. In the table below, the relative integrals of each hydrogen bond signal are listed by using the sum of the integrals. The integrals were obtained using Mestrenova® 14.3.3 with linear baseline correction. The spectra were processed using the Bernstein Polynomial Fit (polynomial order: 3), with baseline correction applied to the zoomed region.

DOSY experiments (for details, refer to SI 3.9) further confirmed the presence of larger aggregate species, as indicated by the significantly higher volumes calculated from the diffusion coefficients compared to those of the binary complex and the anticipated volume of the ternary complexes (refer to Table S3). CPA **1b** exhibits a volume of 2331 $Å^3$ in the **1b**/**2a**/HE **3b** sample (1:1:1 stoichiometry). Since this volume represents the average volume of all CPA species, it significantly surpasses the volume of the binary complex (1625 $Å^3$). Moreover, when calculating the volume of the ternary complex by adding the measured volume of the free HE **3b** to the binary complex (1625 $Å^3$ + 506 $Å^3$ = 2131 $Å^3$), the determined value for the CPA in the 1:1:1 **1b**/**2a**/HE **3b** sample still significantly exceeds this value. This is notable considering that the ternary complex is expected to be smaller than 2131 $Å^3$ because the HE **3b** is located inside the binding pocket of the CPA next to the imine **2a**. Additionally, based on the binding constant of the ternary complex (see SI chapter 3.7), only a small percentage of the CPA should populate the ternary complex. This would result in a partial increase of the calculated volume due to the exchange with the binary and ternary complex. Hence, the volume of 2331 $Å^3$ is a significant increase in comparison to both binary and ternary complex hinting towards bigger aggregate.
In addition, a sample with only CPA **1b** was investigated revealing a volume of 1748 $Å^3$. This relatively high volume is a result of the dimerization behavior of CPAs. Nevertheless, this demonstrates that even for a CPA/CPA-dimer only a volume of 1748 $Å^3$ can be determined.
Hence, a by far larger aggregate than the ternary complex or a single CPA-dimer has to be responsible for the significant increase in volume from 1625 $Å^3$ to 2331 $Å^3$. This again supports the proposed [3:3] dimeric species.

3.6.2. 2:1:1 dimeric species

Based on these results, we tried to investigate if other dimeric structures such as the 2:1 dimeric species are also observed as ternary complex. Here, a characteristic singlet for the CPA/CPA hydrogen bond along with a doublet for the CPA/CPA/imine hydrogen bond should be detected for each imine isomer. Similar to the ternary complex an additional CPA/CPA/HE hydrogen bond signal is expected upon addition of the HE **3b**, in case of a dimeric CPA/CPA/imine/HE complex.[12] To investigate the 2:1 dimeric species, a 2:1:1 stoichiometry of CPA/imine/HE is necessary to detect any 2:1 dimers. Hence, a sample with a 2:1:1 stoichiometry of TRIFP **1a**/**2a**/HE **3b** was prepared and measured at 180 K to force the system into a possible CPA/CPA/imine/HE.

Here, the above described typical pattern for the dimeric species was clearly observed for both the *Z*- and *E*- imine isomers (see Figure S11 C). Nevertheless, similar to the highfield shift of the 1:1:1 ternary complex, a slight highfield shift of the dimeric species CPA/imine signals is observed when comparing those with a 2:1 sample (CPA/imine) without HE **3b** (TRIFP **1a**/*Z*-**2a**: -0.15 ppm; TRIFP **1a**/*E*-**2a**: -0.12 ppm). Furthermore, the CPA/CPA hydrogen bond signals are shifting towards low-field upon addition of the HE **3b** (for TRIFP **1a**/TRIFP **1a**/*Z*-**2a**: +0.26 ppm; for TRIFP **1a**/TRIFP **1a**/*E*-**2a**: +0.30 ppm) and a new low populated signal is detected in the CPA/HE hydrogen bond region (9-10 ppm). Given the increasingly complex spectra, a chemical shift mapping is the most effective method for confirming the formation of a ternary complex. Therefore, a chemical shift mapping was performed starting from a 2:1:0 stoichiometry of CPA:imine:HE and adding HE **3b** in 0.5-2 eq. relative to the imine. Here, a clear shift of all mentioned signals was observed with increasing HE **3b** concentration (see Fig S14). Hence, although not present in a 1:1:1 stoichiometry, the dimeric CPA/CPA/imine/HE complex can be observed using a 2:1:1 stoichiometry.

Regarding the difference between the spectra of a 1:1:1 stoichiometry and a 2:1:1 stoichiometry. Again, we think the solubility issue is here one main point. The CPAs alone do not dissolve properly at these concentrations and there might be in addition a so far not understood interplay of stabilization in between the different species. Something similar had been observed for palladium phosphoramidite complexes.[10] In addition, there is a significant exchange line broadening for the 2:1:1 sample in comparison to the 1:1:1 sample (97.75 Hz to 61.36 Hz). Our explanation is that the phosphoric acid aggregate exchanges better with the 2:1:1 complex than with the 1:1:1 complex.

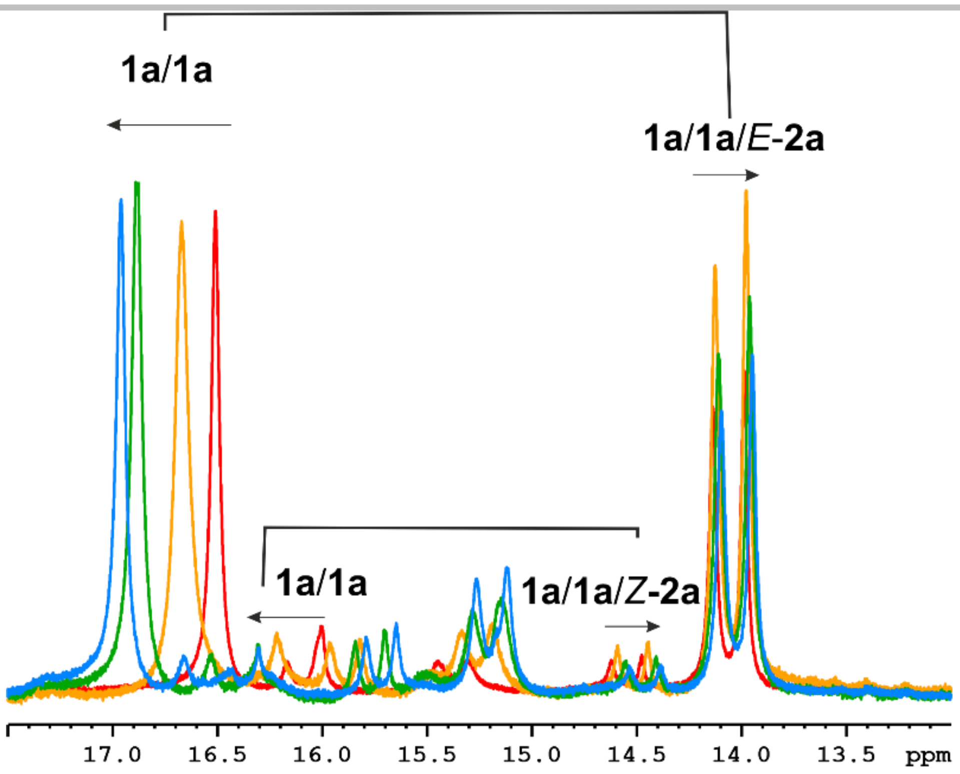

Figure S 14. **^{1}H-NMR chemical shift mapping of the hydrogen bond signals of the dimeric species upon addition of 0-2 eq. HE 3b (2:1:X stoichiometry, 600MHz, CD_2Cl_2, 180K). A general highfield shift is observed for the CPA/CPA/imine hydrogen bonds upon addition of the HE 3b, while a low field shift is observed for the CPA/CPA hydrogen bonds (order: red, orange, green, blue).**

3.6.3. Additional species

Furthermore, also for other CPAs the structural space was investigated. Especially the sterically more hindered TRIP **1d** and TiPSY **1e** were assumed to have a smaller structural space. Astonishingly the opposite trend was observed. While for TRIM **1c** a relatively similar structural space to TRIFP **1a** and **1b** was found (see Fig S15A), in samples containing TRIP **1d** and TiPSY **1e** a lot of additional hydrogen bond signals were detected. For all TRIP **1d** systems a plethora of signals was detected in the region of 14.5-16.0 ppm (see Fig S15B). For TiPSY **1e** highly populated species are observed in a chemical shift range of 10.0-12.0 ppm with imine **2b** which is not the case for any other system (see Fig S15C). However, none of these signals could be assigned as no signals were observed by 2D-NMR. Nonetheless, these results again demonstrate that steric limitations are by far less significant for the structural space of the ternary complex than anticipated based on the steric hinderance.

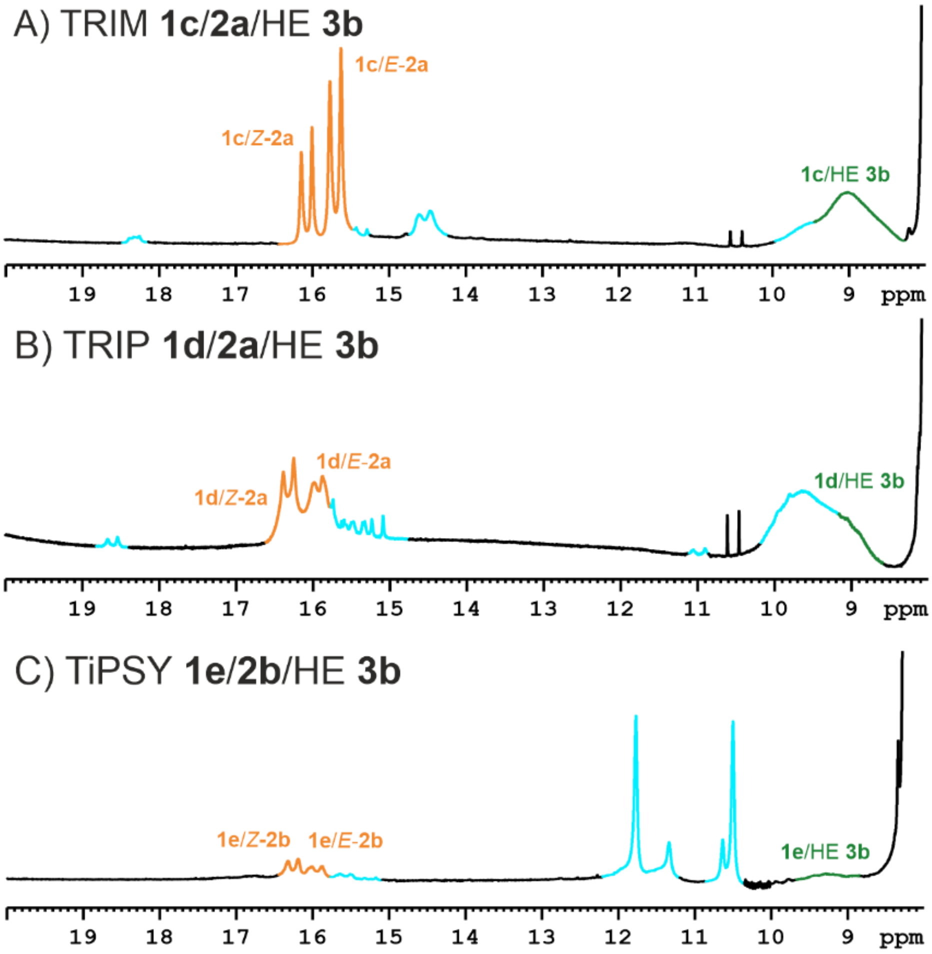

Figure S 15. A) ^{1}H-spectrum of the ternary complex of TRIM 1c/2a/HE 3b (1:1:1 stoichiometry, 600MHz, CD_2Cl_2, 180K). All additional hydrogen bond signals are colored in light blue. B) ^{1}H-spectrum of the ternary complex of TRIP 1d/2a/HE 3b (1:1:1 stoichiometry, 600MHz, CD_2Cl_2, 180K). All additional hydrogen bond signals are colored in light blue. C) ^{1}H-spectrum of the of TiPSY 1e/2a/HE 3b in a 2:1:1 stoichiometry (stoichiometry, 600MHz, CD_2Cl_2, 180K). All additional hydrogen bond signals are colored in light blue.

In this context, especially the combination of TiPSY **1e** with **2e**, which also has a sterically hindering substituent with its naphthalene group at the ketone-part of the imine, showed that a steric limitation seems to play no role for the ternary complex as well as the [3:3] dimer. Here, again a high population of the [3:3] dimeric species is detected (see Fig S16, blue).

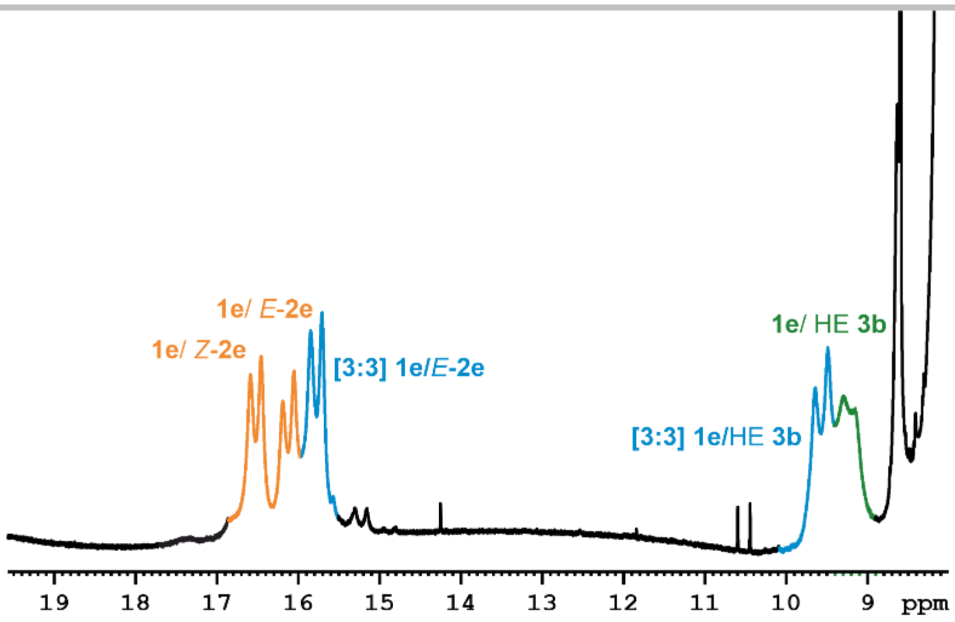

Figure S 16. **^{1}H-spectrum of the ternary complex of TiPSY 1e/2e/HE 3b (1:1:1 stoichiometry, 600MHz, CD_2Cl_2, 180K). All additional hydrogen bond signals are colored in blue.**

3.7. Binding constant

To investigate the binding of HE **3b** to the binary complex TRIFP **1a**/**2a**/HE **3b**, the following approach was employed for chemical shift mapping using NMR-spectroscopy and binding isotherms:
An NMR sample was prepared with TRIFP **1a** (20 mM) and imine **2a** (20 mM) following **GPSP** but without the addition of HE **3b**, which corresponds to a sample of the binary complex. Then ^{1}H and ^{1}H,^{13}-HSQC NMR spectra were recorded at 180 K. Subsequently, the sample was equilibrated at room temperature, and HE **3b** (0.5 eq.) was added under argon and NMR spectra were recorded again. This procedure of adding Hantzsch ester equivalents (0.5 – 10 eq.) to the sample was repeated.
The spectra were calibrated to TMS and the chemical shifts monitored. The most significant changes were observed for the CPA/*Z*-**2a** hydrogen bond. These changes were manually fitted in an Excel spreadsheet to a binding isotherm fitting curve corresponding to a 1:1 binding model:[13,14]

$$(1)\quad \Delta\delta = \Delta\delta_{max} \frac{([P_T] + [L_T] + K_d) - \sqrt{([P_T] + [L_T] + K_d)^2 - 4[P_T][L_T]}}{2[P_T]}$$

Both the dissociation constant K_d and maximum chemical shift difference $\Delta\delta_{max}$ (representing the assumed "ternary complex" chemical shift) values were fitted iteratively.
$[P_T]$: Describes the concentration of the binary complex ("protein") and was set to 20 mM, which is constant over all measurements.
$[L_T]$: Describes the total HE **3b** concentration ("ligand"), which was determined by integration of the ^{1}H-NMR spectra relative to the imine signals.
The K_d and δ_f values from selected chemical shift changes were fitted in Dynafit using 1:1 binding model.[15]

In general, the signals of the CPA/*Z*-**2a** showed a significantly larger highfield shift than the CPA/*E*-**2a** (see Fig S17). Fitting the chemical shift mapping revealed a binding constant of K_d = 22.2 indicating a weak binding of the HE **3b** to the binary complex. Despite the fact that the absolute chemical shift differences for the CPA/*E*-**2a** hydrogen bond between ternary and binary complex are significantly smaller than the respective CPA/*Z*-**2a** hydrogen bond, the nearly linear change suggests a weaker binding of the *E*-complex.

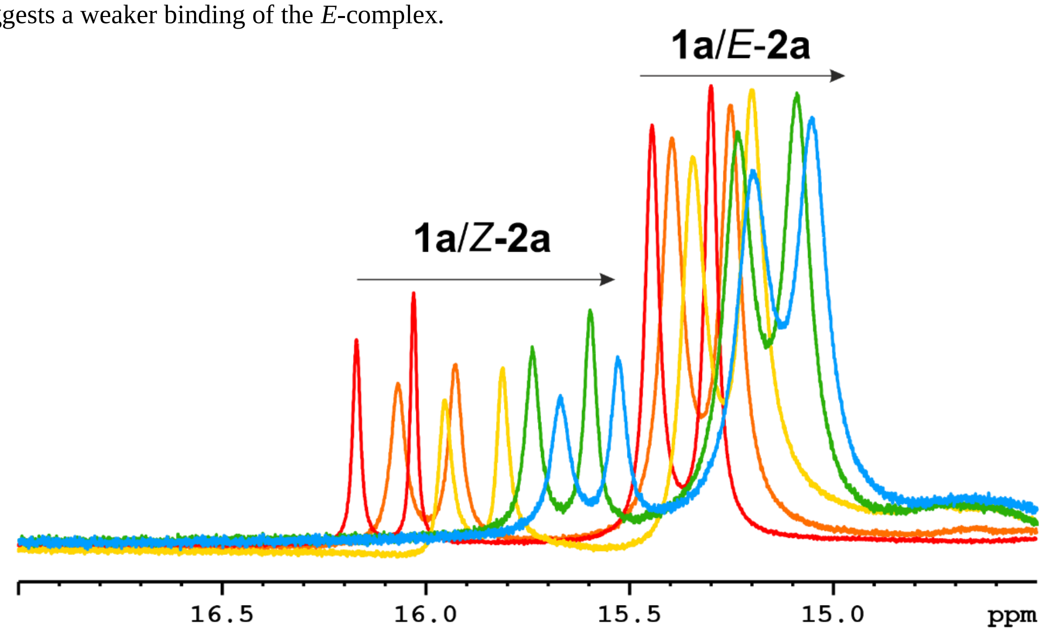

Figure S 17. ^{1}H-NMR chemical shift mapping of the TRIFP 1a/Z-2a (left) and TRIFP 1a/*E*-2a (right) hydrogen bond signals upon addition of 0-10 eq. HE 3b (600MHz, CD_2Cl_2, 180K). A general highfield shift is observed upon addition of the HE 3b (order: red, orange, yellow, green, blue).

1H:

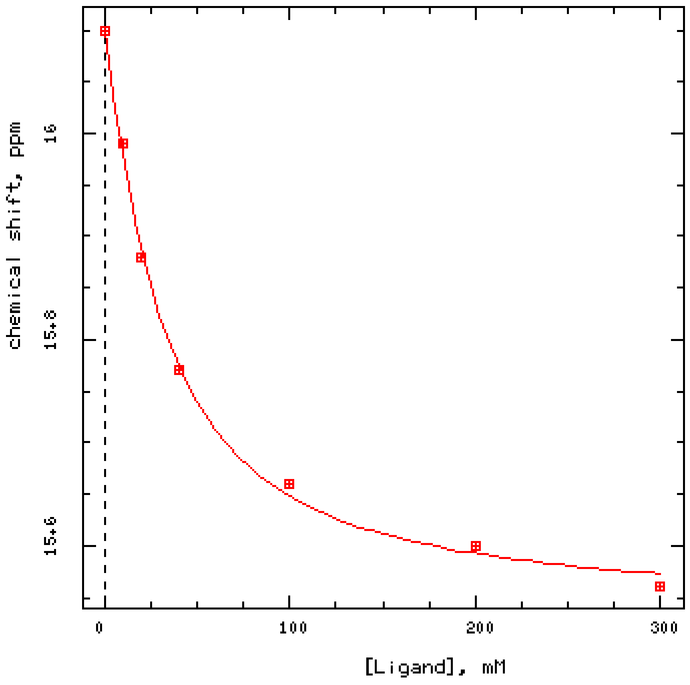

Figure S 18. TRIFP 1a/Z-2a hydrogen bond signal binding isotherm (7 titration points). K_d **= 22.2 ± 1.8 mM;** $\Delta\delta_{max}$ **= 15.524 ppm;** $K_a = 1 / K_d$ **= 45.05** M^{-1}**.**

1H:

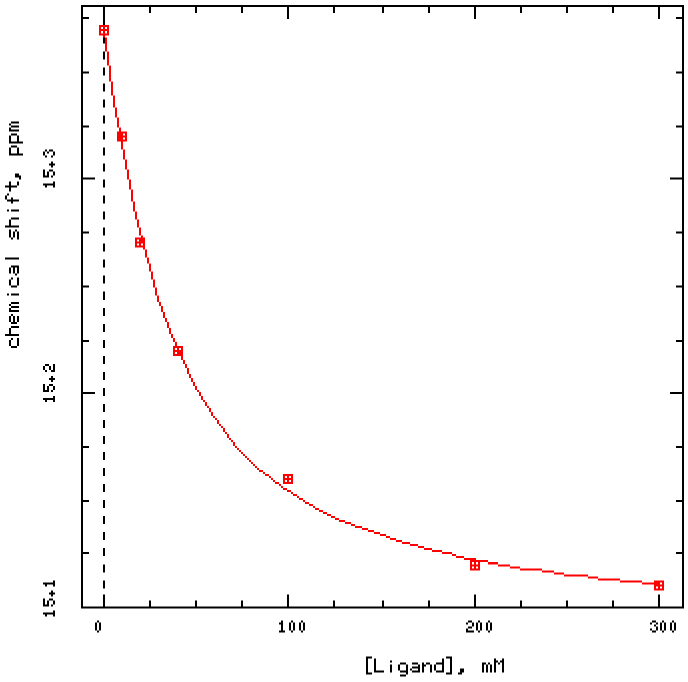

Figure S 19. TRIFP 1a/E-2a hydrogen bond signal binding isotherm (7 titration points). K_d **= 19.8 ± 3.9 mM;** $\Delta\delta_{max}$ **= 15.099 ppm,** $K_a = 1 / K_d$ **= 50.50** M^{-1}**.**

3.8. Diffusion ordered spectroscopy (DOSY)

Diffusion ordered NMR spectroscopy (DOSY) experiments were performed using a Bruker Avance III HD 600 MHz spectrometer with a TBI (Triple resonance broadband inverse) 5 mm CPPBBO 1H/19F-BB or 1H/31P-BB probe head with Z-gradient and BVT unit was used. The temperature of the spectrometer was regulated by a BVT 3900 unit and liquid nitrogen. All measurements were performed at 298 K. Temperature was certified by internal NMR calibration samples from Bruker®. NMR Data was processed, evaluated, and plotted with TopSpin 3.2 software. Further analysis of the measurements was performed with Microsoft Excel (Version 16.0.10359.20023 64 Bit).
All DOSY measurements were performed with the convection suppressing DSTE (double stimulated echo) pulse sequence developed by Jerschow and Müller in a pseudo 2D mode.[16] Therefore, TMS was added to the sample and used as reference for the ^{1}H chemical shifts and for temperature as well as the viscosity of the solvent. For the measurement a set of 4 dummy scans and 8-32 scans was used for all samples. A relaxation delay of 3.5 s was used for all samples. The diffusion time delay was set to 40 ms and the gradient pulse lengths (p30, SINE.100 pulse shape) were optimized for each species to give a sigmoidal signal decay for varying gradient strengths between 5% and 95%. Optimal pulse lengths of 1.0 – 1.3 ms were used at 298 K for TMS and the formed aggregates. For each species, 32 spectra with linear varying gradient strength from 5% to 95% were measured. For integration, all separated signals were used for each substrate. Thereby, no line broadening occurred for increased gradient strengths.

The signal intensities of these groups in the DOSY spectra were classically analyzed as a function of the gradient strength by the in Bruker TopSpin 3.2 included software T1/T2 relaxation package by employing the Stejskal-Tanner equation.[17] Based on the obtained translational diffusion coefficients, the hydrodynamic radii of the analytes r_H were estimated following the Stokes-Einstein equation (S6), with D_i = self-diffusion coefficient k = Boltzmann constant, T = temperature, η = viscosity of the sample, c = correcting factor, F = shape factor:[18]

$$D_i = \frac{k_B T}{F c \pi \eta r_H} \qquad \text{(S1)}$$

The shape factor F was set to 1 for a spherical shape. The semi-empirical modification by Chen (S7) was used to calculate the correction factor c. Therefore, a from literature known value for the radius of the corresponding solvent was used ($r_{CD_2Cl_2}$ = 2.46 Å[19]).[20]

$$c_{Chen} = \frac{6F}{1 + 0.695\,(\frac{r_{solv}}{r_{ref}})^{2.234}} \qquad \text{(S2)}$$

Viscosity calibration of the derived D_i values was performed with literature known values for the radii of TMS (r_{ref} = 2.96 Å, calculated from hard-sphere increments[21]) and the experimentally determined diffusion coefficient D_{ref} of TMS, which is determined individually for each sample.

$$\eta\,[kg/ms] = \frac{kT\,(1 + 0.695\,\left(\frac{r_{solv}}{r_{ref}}\right)^{2.234})}{6\pi D_{ref}\, r_{ref}} \qquad \text{(S3)}$$

After including all correction and calibration equations in the Stokes equation (S6), the equation was rearranged for the hydrodynamic radii r_H (S9). The corresponding volumes V_A were calculated with the assumption of a spherical shape.

$$D = \frac{kT\,(1 + 0.695\,\left(\frac{r_{solv}}{r_{ref}}\right)^{2.234})}{6\pi\eta r_H} \qquad \text{(S4)}$$

The experimental self-diffusion coefficients D_i, the viscosity corrected hydrodynamic radii r_H and the resulting volumes V_A of all samples are depicted in Table S3. TMS was used as viscosity reference in each sample separately with the variation in its experimental diffusion coefficients reflecting the different viscosities depending on the substrate concentration. The average D_i values were derived by using all baseline separated signals that were referring to the same species. As stated above the hydrodynamic values and the volumes are viscosity corrected and therefore the only values, which can be directly compared with each other.

Table S3. Experimental self-diffusion coefficients D_i, viscosity corrected hydrodynamic radii r_H and resulting volumes V_A of various 1b/2a/3b combinations (40 mM). TMS was used as viscosity reference for the experimental self-diffusion coefficients D_i to allow for a comparison of hydrodynamic radii r_H and resulting volumes V_A. The corresponding self-diffusion coefficients D_i of TMS are given for each sample. Entry 1-5: SW = 22 Hz, O1P = 10.0 ppm, gradient strength 5-95% linear. Samples were measured at room temperature.

Entry	Species	Diffusion coefficient D_i [$m^2.s^{-1}$]	Hydrodynamic radius r_H [Å]	Volume V_A [$Å^3$]
1	OMe-CPA **1a**<br>*TMS*	7.12E-10<br>2.48E-09	7.47	1748
2	**2a**<br>*TMS*	1.53E-09<br>2.42E-09	3.97	262
3	HE **3b**<br>*TMS*	1.26E-09<br>2.68E-09	4.94	506
4	OMe-CPA **1b**/ **2a**<br>*TMS*	6.81E-10<br>2.30E-09	7.28	1625
5	OMe-CPA **1b**/ **2a** /HE **3b**<br>*TMS*	3.11E-10<br>1.21E-09	8.23	2331

4. Conformational Analysis

4.1. Computational Details

The initial conformational search was performed by using CREST[22] which uses meta-dynamics[23]. A few of these pre-optimized structures were chosen based on chemical intuition for further optimization in QM level of theory. The B97-D[24] functional combined with def2-SVP[25] basis set was used for geometry optimization and frequency analysis in ORCA 5.0.3 software package[26–28]. The global and local minimum energy structures were confirmed by 3n-6 non-zero positive normal modes in a frequency analysis using the harmonic oscillator approach. To adapt the experimental conditions of 180K and CD_2Cl_2 all the calculations were performed in CPCM [29] with the di-electric constant ε=16.20.
The catalyst of the reaction is an acidic CPA, whereas the other two reactants are bases (imine and Hantzsch ester). The imine is more basic than the HE (Hantzsch ester) and hence reacts with the acid (CPA catalyst) first to form the CPA/imine binary complex.[4] The imine binds inside the cavity of the CPA and can also rotate inside to give rise to more than one conformer of the binary complex.[30] The HE then binds to these possible binary complexes to form the pre-TS ternary complex (see Figure S20).

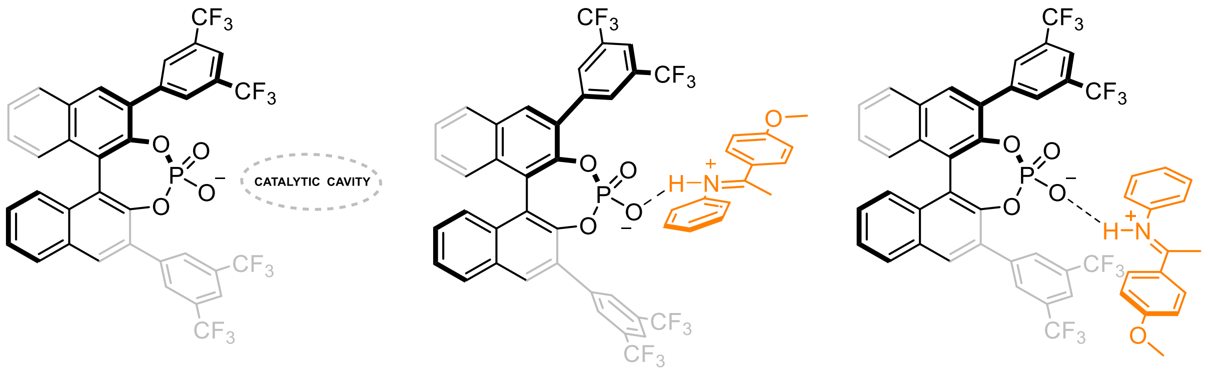

Figure S 20. The two different conformers of the binary complex for the *E*-imine resulting from the rotation of imines inside the cavity.

Four conformers of the ternary complex of TRIFP **1a**/ *E*-imine **2a**/ HE **3b** are identified within the limit of 5 kcal/mol energy with respect to the most stable conformer.

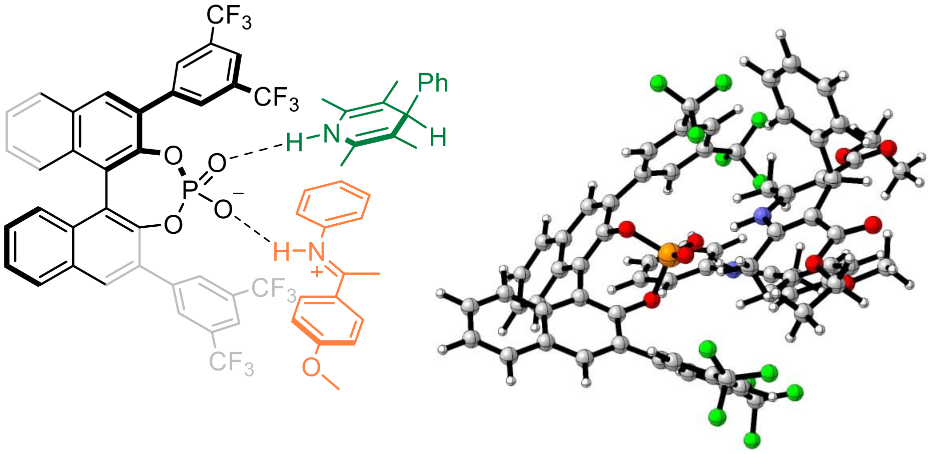

Figure S 21. Lowest energy conformer, C1. The Hatzsch ester is inside the cavity.

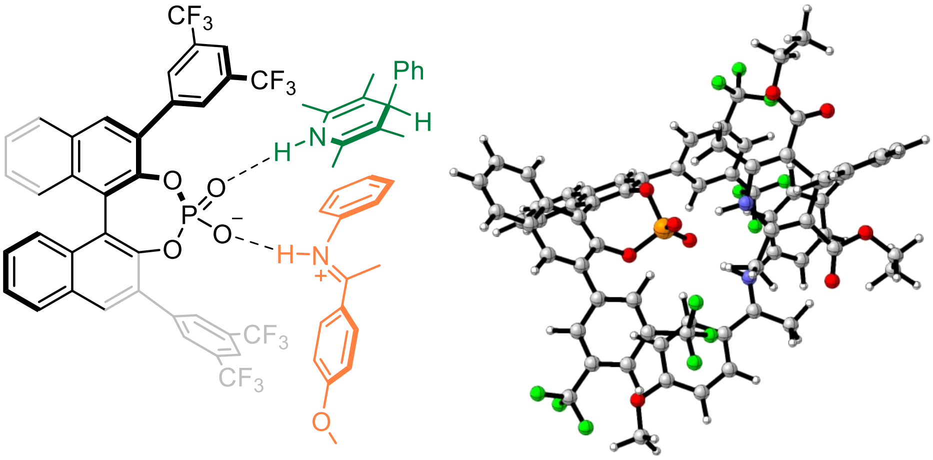

Figure S 22. Conformer C2. The Hantzsch ester is inside the "pocket", but the cavity is a bit more open than in C1.

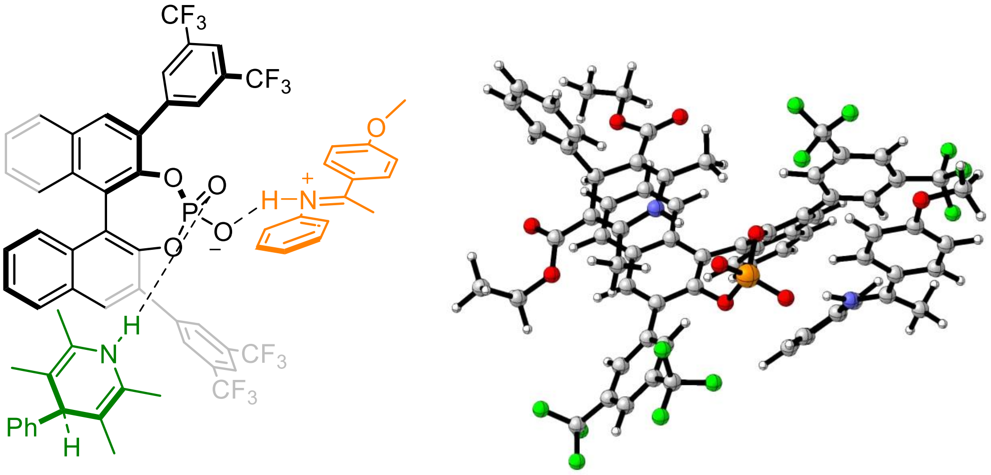

Figure S 23. Conformer C3. The Hantzsch ester is outside the cavity and is close to the naphthyl rings of the CPA.

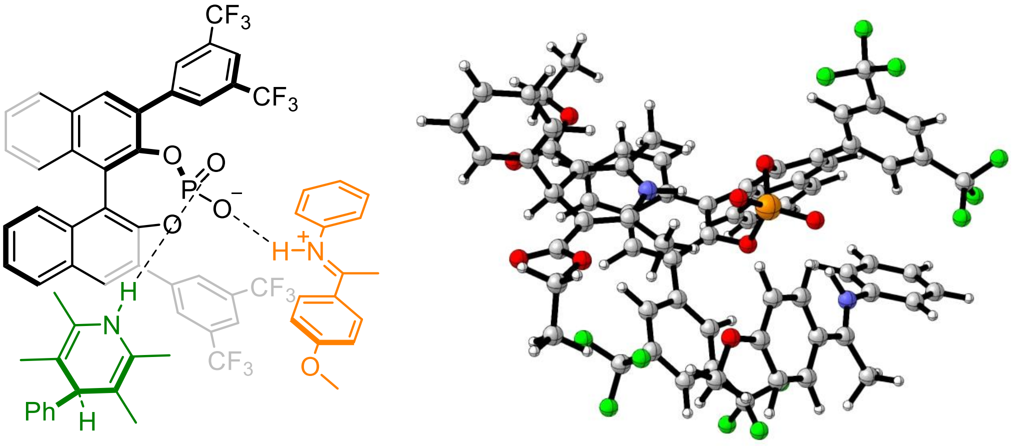

Figure S 24. Conformer C4. The Hantzsch ester is outside the cavity and is close to the naphthyl rings of the CPA. The imine is flipped in comparison to C3.

Table S4. Relative free energies of the conformers and the respective hydrogen bond strengths. (Level of theory: B-97D/def2-SVP, Solvent model: CPCM ε=16.2, Thermochemistry at 180K).

Conformer	ΔG [kcal/mol]	Imine H-Bond length (Å)	Imine H-Bond B.E. ΔG [kcal/mol]	Hantzsch Ester H-Bond length (Å.)	Hantzsch H-Bond B.E. ΔG [kcal/mol]
**C1**	0.0	1.57	-22.0	1.90	-5.5
**C2**	3.6	1.68	-16.8	1.82	-7.0
**C3**	2.5	1.67	-17.5	1.95	-5.1
**C4**	4.6	1.65	-7.1	1.77	-7.6

The conformers where the HE and the imine both are inside the cavity could potentially lead to the observed products. In conformers C1 and C2, both the HE and imine are in the cavity, and the HE is above the re-face of the imine. Hence, these two conformers are both possible pre-TS assemblies. Whereas conformers C3 and C4 are not productive ternary complexes although they apparently also exist in the solution.

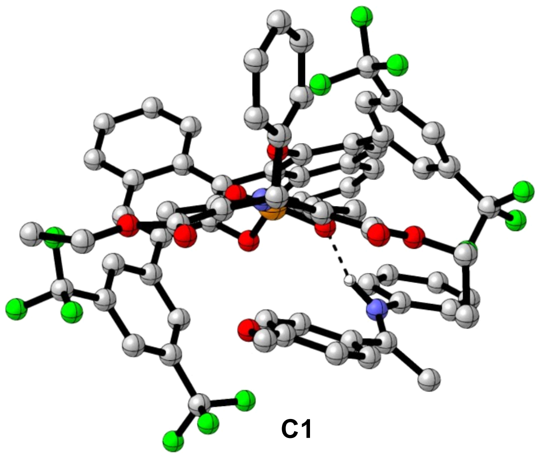

C1

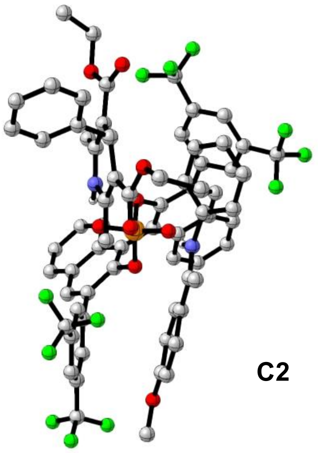

C2

Figure S 25. The aryl substituents of the CPA are parallel to each other in C1, but not in C2 (the C-H hydrogens are omitted for clarity).

The key difference between C1 and C2 are the aryl groups of the CPA, as shown in Figure S24. There is a noticeable change in relative conformations of HE and the imine with respect to the CPA in both conformers.
The binding energy of each hydrogen bond (see Table S4) is estimated by the equations (BE in kcal/mol = -223.08 x ρ_{BCP} + 0.7423 for neutral molecules and BE in kcal/mol -332.08 x ρ_{BCP} – 1.0661 for charged molecules, where ρ_{BCP} is the electron density in a.u. at the critical points) given by Lu et al. based on AIM analysis.[31] The hydrogen bond between the phosphate and protonated imine is stronger than that of HE because of stronger electrostatic interaction.

Stability of Ternary complexes at 298K: The dielectric constant of DCM at room temperature is 8.93 as opposed to 16.20 at 180 K. The conformers C1, C2 and C3 were recalculated in B97D/def-2SVP level of theory and CPCM model was used for implicit solvation with ε = 8.93. There was no appreciable change in the geometries of the conformers (below

1% change), but the electronic energy and the free energy changed. Each of the conformers have lower free energies at 298 K than in 180 K. While qualitative trend of free energies of conformers at 180 K and 298 K is unchanged, the relative differences change slightly.

Table S5. Relative free energies of C1, C2 & C3 at 298 K with ε = 8.93.

Conformers	Relative free energies at 180K	free energy at 298K w.r.t C1 at 180K (w.r.t C1 at 298K)
**C1**	0.0	-33.6 (0.0)
**C2**	3.4	-30.8 (2.8)
**C3**	2.6	-31.8 (1.8)

The same computational protocol was employed to get several ternary complexes of CPA **1b** (OMe-CPA), imine **2a** and HE **3b**. Similar ternary complexes with the *Z*-imine (with both CPAs **1a** and **1b**) were also optimized and confirmed by frequency analysis. The [3:3] molecular complex of two CPA **1b**, two *E*-imine **2a** and two HE **3b** was also optimized.

Process of ranking of the conformers:

Since the frequency calculation is computationally demanding, at first only geometry optimizations were performed on 9 ternary complexes (including both *E*- and *Z*- imines). They were sorted according to their electronic energies (SCF energies). The conformers within a threshold of 5 kcal/mol relative to the most stable conformer were chosen for further frequency analysis. The full list of conformers with their electronic energies can be found in the table below.

Table S6. Relative electronic energies of the conformers. The ones highlighted in red are not considered for thermochemistry calculations. (Level of theory: B-97D/def2-SVP, Solvent model: CPCM ε = 16.2).

Conformer	Components	Relative SCF energies (kcal/mol)
**C1**	CPA **1a**, *E*-Imine **2a**, HE **3b**	0.0
**C2**	CPA **1a**, *E*-Imine **2a**, HE **3b**	3.1
**C3**	CPA **1a**, *E*-Imine **2a**, HE **3b**	2.5
**C4**	CPA **1a**, *E*-Imine **2a**, HE **3b**	4.6
**C101**	CPA **1a**, E-Imine **2a**, HE **3b**	5.9
**C5**	CPA **1a**, Z-Imine **2a**, HE **3b**	1.1
**C6**	CPA **1a**, Z-Imine **2a**, HE **3b**	5.3
**C102**	CPA **1a**, Z-Imine **2a**, HE **3b**	7.4
**C103**	CPA **1a**, Z-Imine **2a**, HE **3b**	8.2

[3:3] Molecular Complex:

The [3:3] molecular complex containing two CPA **1b**, two *E*-imines **2a** and two HEs **3b** was pre-optimized at the GFN2-xTB level of theory with DCM as solvent using the ALPB model integrated in xTB version 6.4.1.[32–34] This tight-binding semi-empirical method yields reasonably good hydrogen bonded molecular complexes considering dispersion and is designed for larger systems.[34] This complex is presumed to be the dimer of the most stable corresponding ternary complex C7 (See Figure S27). Three possibilities of combinations were explored as represented schematically in Figure S26.

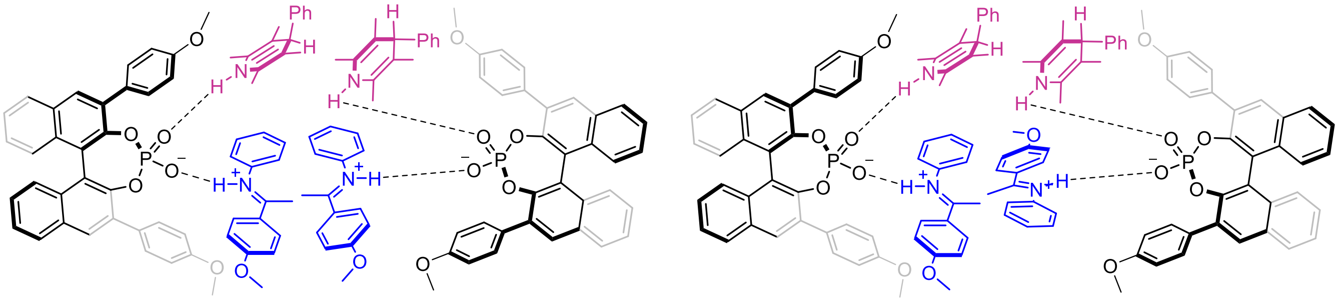

D1: Head-to-Head [3:3] dimer-1

D2: Head-to-Head [3:3] dimer-2

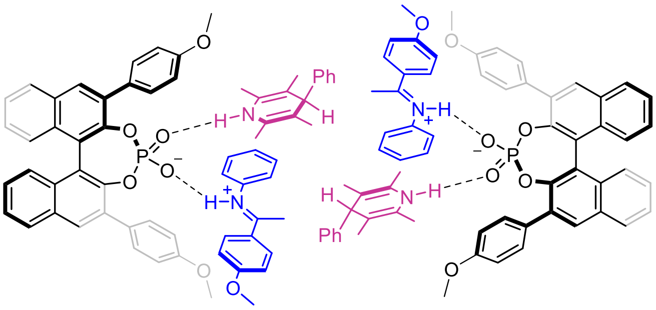

D3: Head-to-Tail [3:3] dimer

Relative SCF Energy

D1 0.0 kcal/mol

D2 3.3 kcal/mol

D3 7.0 kcal/mol

Level of theory: GFN2-xTB

Figure S26. The three [3:3] dimers pre-optimized to explore the conformational space.

The pre-optimization of the three [3:3] dimers revealed D1 to be the most stable. Hence, due to extremely high computational cost, only D1 was optimized in the DFT further to compare it to the most stable ternary complex (C7) to compare the change in hydrogen bond lengths. The lengths of the two hydrogen bonds of the two HEs-**3b** with the phosphoryl oxygen of CPA **1b** decreased by 5.6% (1.82 Å in D1, 1.93 Å in C7) and 3.1% (1.87 Å in D1, 1.93 Å in C7). The hydrogen bond lengths of the two *E*-imines **2a** remained largely unchanged with 1.7% (1.73 Å in D1, 1.70 Å in C7) and -0.6% (1.69 Å in D1, 1.70 Å in C7).

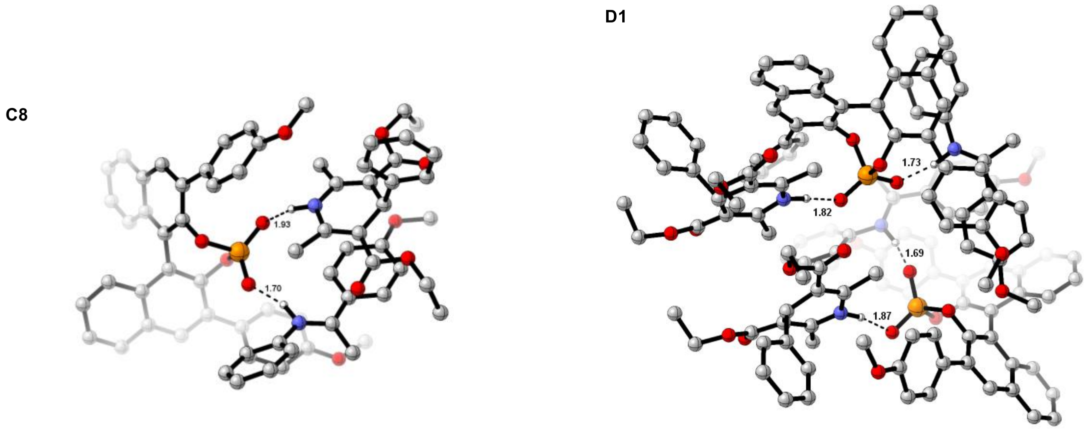

Figure S27. Hydrogen bonds in C7 and D1. Level of theory: B97-D/def2-SVP, solvent model: CPCM ε=16.2.

The iminium hydrogens in **D1** are interacting with the aromatic rings as highlighted in Figure S28 and the NCI surface in Figure S29. This rationalizes why this correlated well the up-field NMR shifting of the signal.

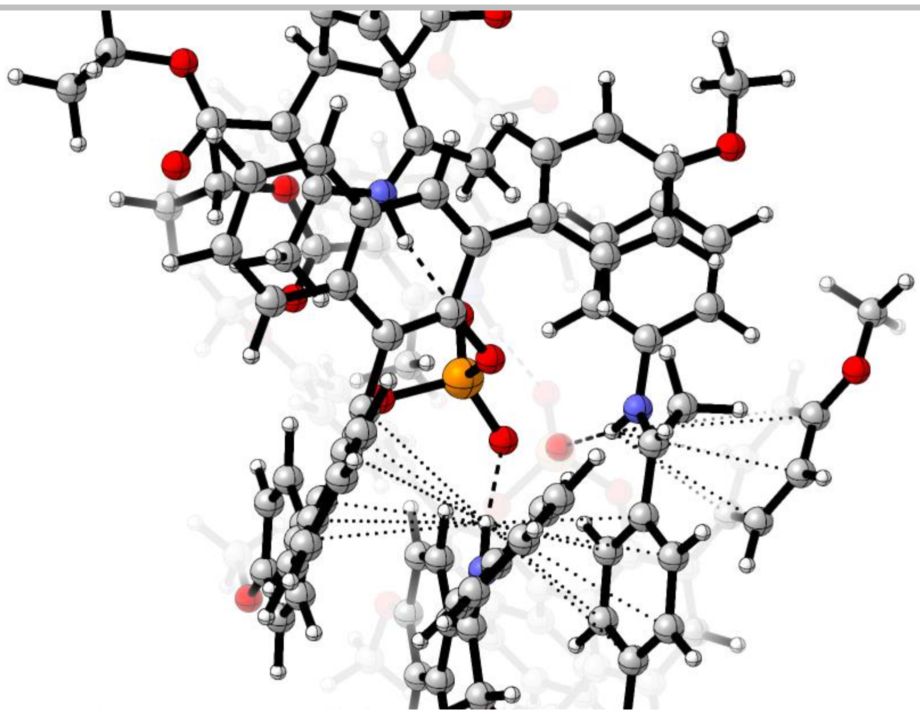

Figure S28. Zoomed-in view of an *E*-imine 2a in [3:3] dimer D1.

The primary driving force behind the formation of the [3:3] dimer is the π-stacking of the aryl rings in the whole system as visualized by NCI surfaces.[35]

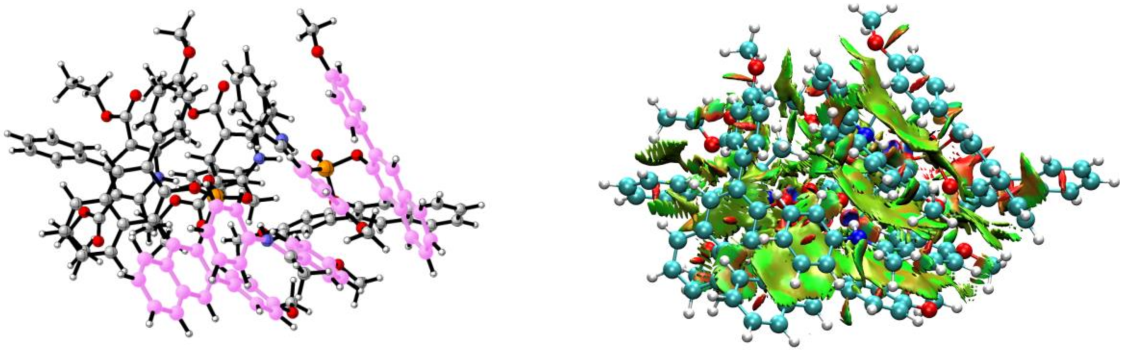

Figure S29. The aryl rings of CPA 1b, *E*-imine 2a stack on each other via dispersion interactions.

4.2.NOE analysis of conformers

The calculated conformers were compared to the NMR data, especially the NOE signals. For this analysis, only NOE correlations of the HE **3b** can be used. This is a result of the fast exchange of binary and ternary complexes leading to an averaged signal set for both the CPA **1** and the imine **2a**. Therefore, a distinction between both complexes is not feasible, but the HE **3b** signals can be unambiguously assigned to the ternary complex. The [3:3] dimer does also not interfere with this analysis, as for the TRIFP **1a** system the [3:3] species is negligible populated and does not result in any observable NOESY signals, simplifying the analysis. In addition, in the OMe-CPA **1b** system a separate signal set for the HE **3b** is detected indicating a slow exchange on the NMR timescale. Hence, the HE **3b** NOE signals can be interpreted without restrictions.

In addition, to distinguish between various conformers only the distinct NOE correlations for each conformer can be used. This means, the calculated conformers were first analyzed regarding the distances between HE **3b** and the CPA **1** as well as imine **2a**. Next, these findings were compared between all conformers and the for each conformer distinct close distances between CPA/HE and imine/HE were noted. These close distances should be observable in NOESY spectra and therefore reveal if the analyzed conformers are existing or not. Contrary, calculated long distances between CPA/HE or imine/HE cannot be used. In case of such distinct long distances, a signal for other conformers with shorter distances would still be observed, making any evaluation inaccessible. Hence, although a huge NOE network is detected, only very

few signals can be used to identify the conformers. Nevertheless, this confined assessment of only the distinct NOEs is indispensable since most signals involve contributions from at least two conformers, preventing the unambiguous identification of a single conformer.

4.2.1. *TRIFP 1a/2a/HE 3b*

The system TRIFP **1a**/**2a**/HE **3b** was selected as model system for the conformer analysis due to a high population of the ternary complex in comparison to other species as well as sharper signals which lead to better NOESY resolution. Here, especially the *E*-imine **2a** complex was used due to its higher population which enabled a full chemical shift assignment. After excluding various conformers based on the NOESY spectra, three conformers were left which are supported by NOE correlations. The three lowest energy conformers (C1, C2, C3) and their respective NOE cross signals are displayed below (S30-S33). Other from calculations expected correlations are also observed between **1a**/HE **3b** and **2a**/HE **3b** but are not shown here, because they are not distinct for the respective conformer.

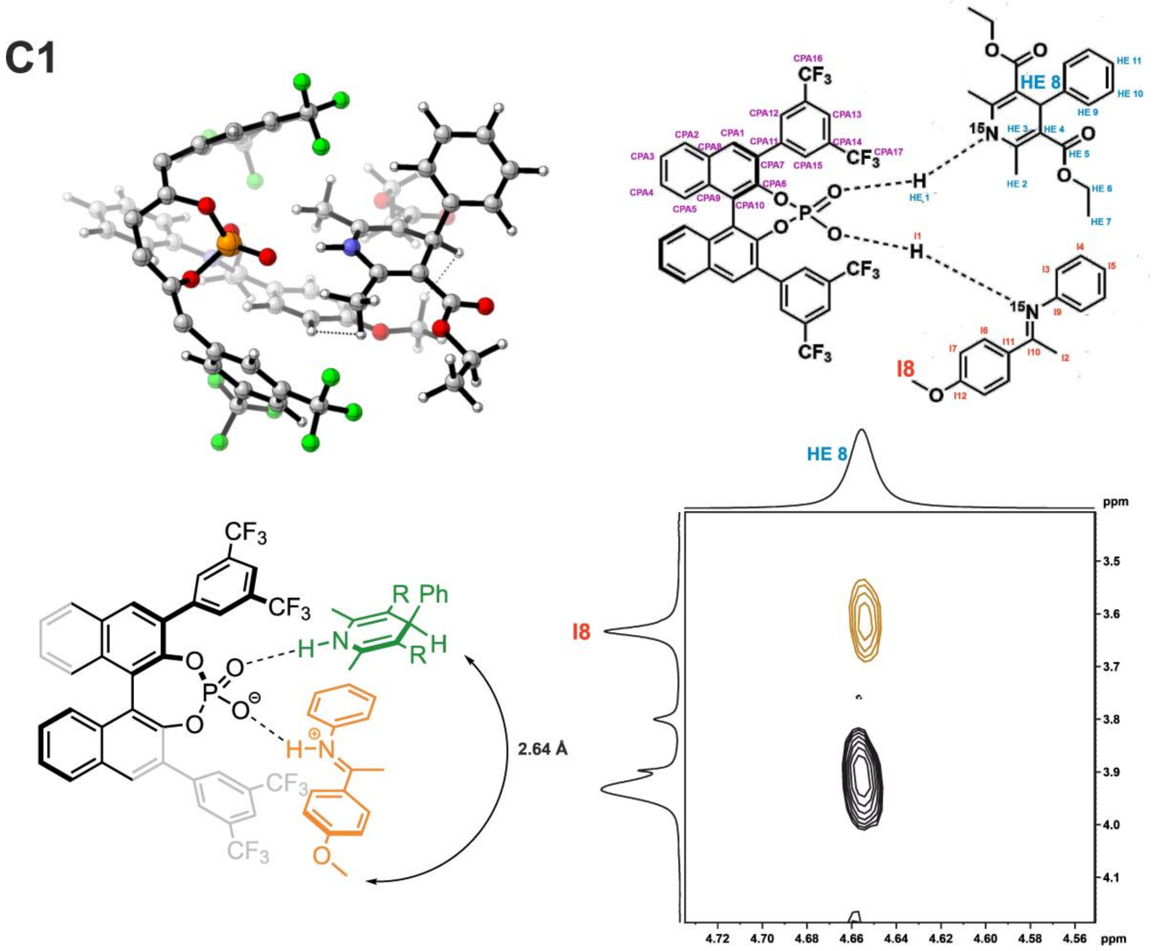

Figure S 30. Conformer 1 NOE analysis. Left right corner the calculated conformer C1 is displayed. One of the distinct signals for this conformer is the NOE correlation between the HE 3b hydrogen atom at 4.66 ppm with the OMe-group of the imine 2a at 3.64 ppm.

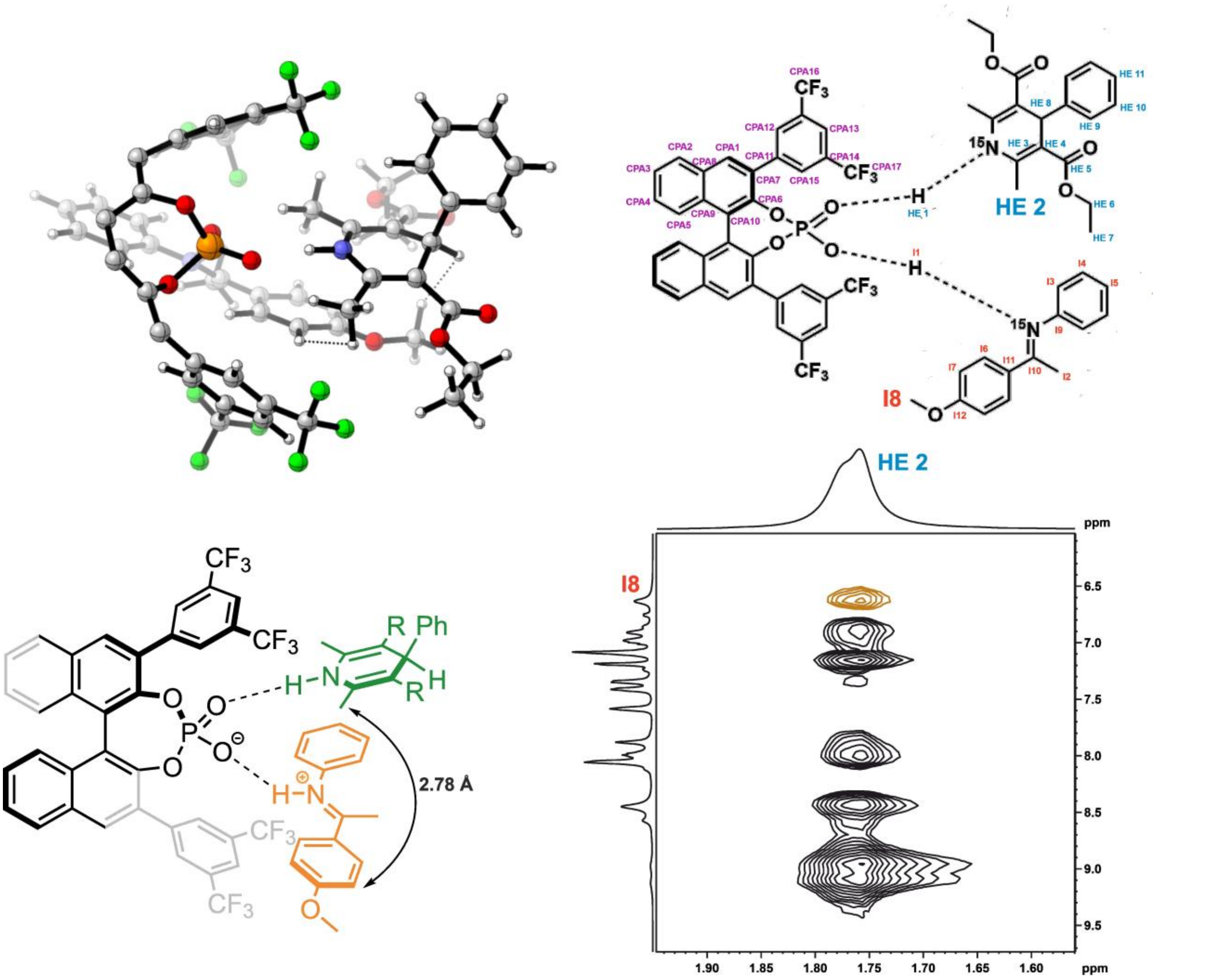

Figure S 31. Conformer 1 NOE analysis. Left right corner the calculated conformer C1 is displayed. One of the distinct signals for this conformer is the NOE correlation between the HE 3b CH_3-group at 1.76 ppm with the *p*-OMe-phenyl-ring of the imine at 6.64 ppm.

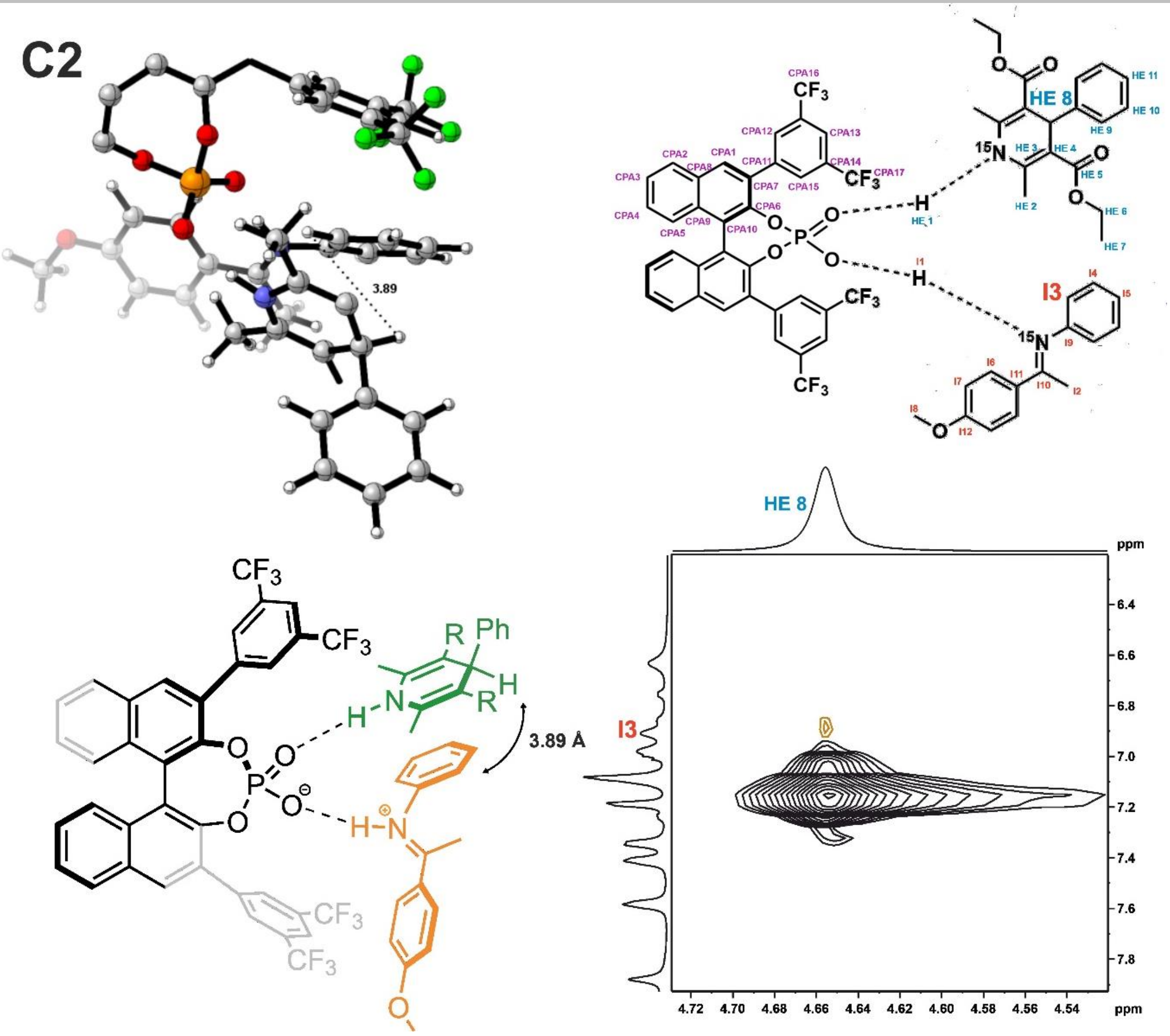

Figure S 32. Conformer 2 NOE analysis. Left right corner the calculated conformer C2 is displayed. One of the distinct signals for this conformer is the NOE correlation between the HE 3b hydrogen atom at 4.66 ppm with the phenyl-group of the imine at 6.90 ppm.

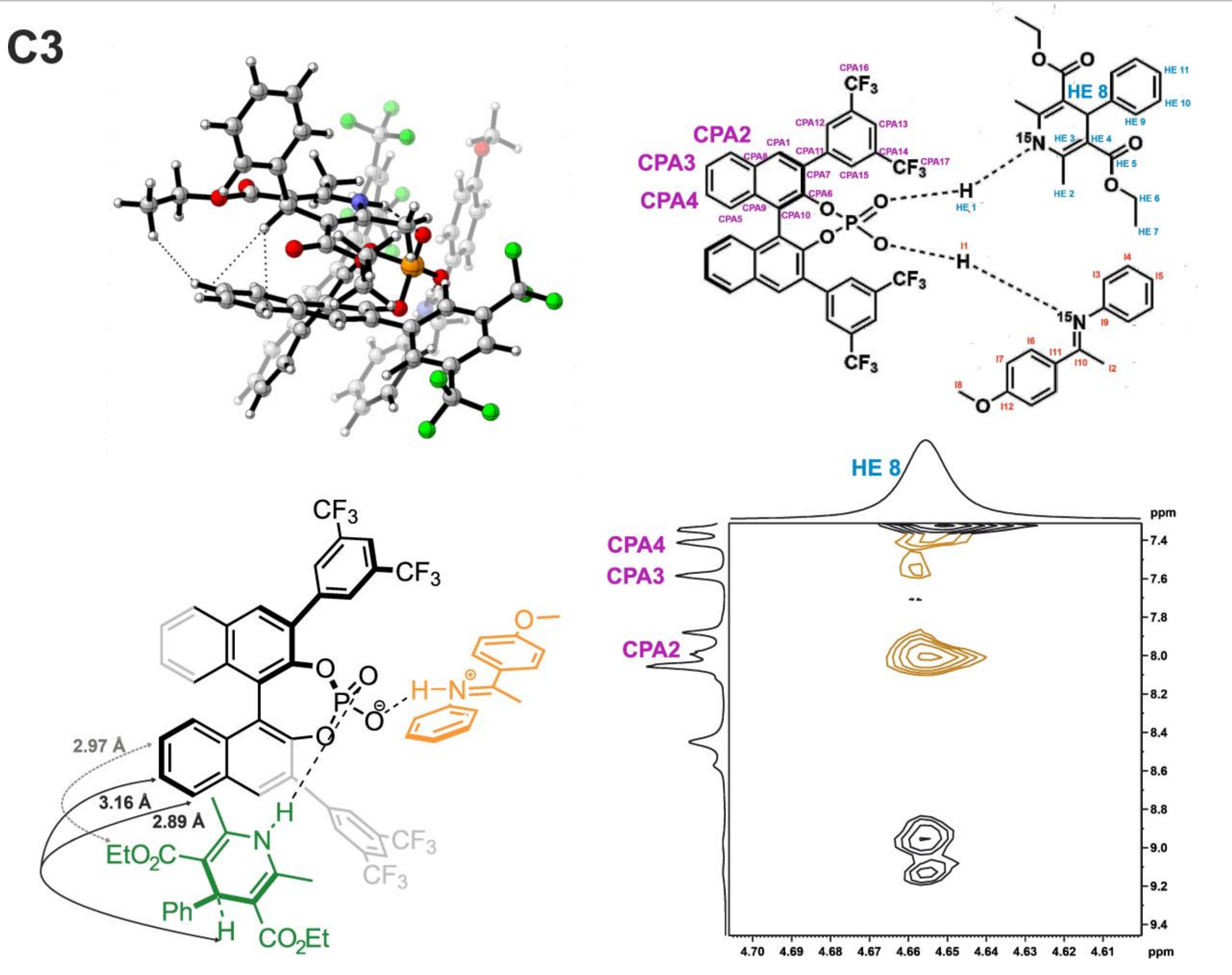

Figure S 33. Conformer 3 NOE analysis. Left right corner the calculated conformer C3 is displayed. The distinct signals for this conformer are the NOE correlation between the HE 3b hydrogen atom at 4.66 ppm with the BINOL-backbone.

4.2.2. [3:3] Dimer with OMe-CPA 1b

For the analysis of the [3:3] dimer, the second model system OMe-CPA 1b/2a/HE 3b was selected due to its high population. Here, nearly a 1:1 ratio between the population of the hydrogen bond signals of the binary/ternary complex and those of the [3:3] dimer. In addition, in this system a clearly observable separate signal set was detected for HE **3b** for the [3:3] species (see Fig S34). However, in NOESY/EXSY spectra exchange processes with the binary and ternary complexes are still observed. Therefore, the calculated structures of the [3:3] dimer were compared with the ternary complexes to find distinct NOE interactions which could still be interpreted but both are too similar to differ.

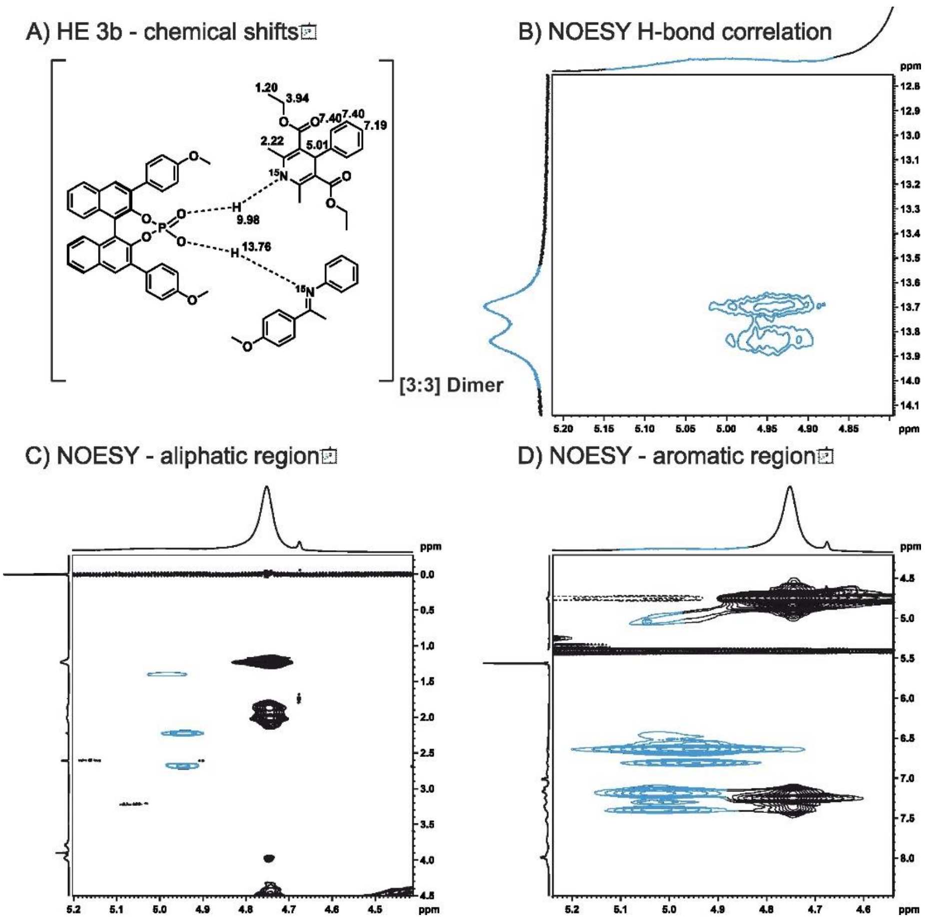

Figure S 34. A) Chemical Shift assignment for the separate signal set of the HE 3b. B) ^{1}H-^{1}H NOESY spectrum with focus on the NOE correlation of the H-bond to the HE 3b. C) ^{1}H-^{1}H NOESY spectrum with a zoom on the NOE correlation of the HE 3b signal at 5.01 ppm in the aliphatic region. D) ^{1}H-^{1}H NOESY spectrum with a zoom on the NOE correlation of the HE 3b signal at 5.01 ppm in the aromatic region.

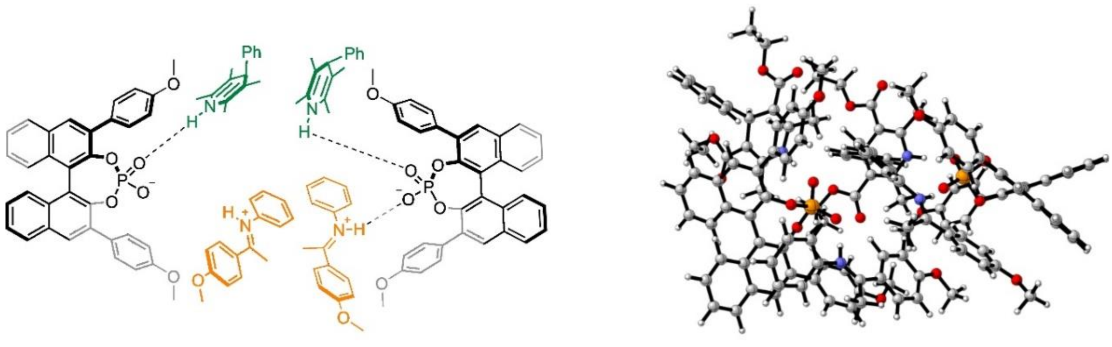

Figure S 35. Scheme of the [3:3] dimeric species (left). Calculated structure of the [3:3] dimeric species that was confirmed by NMR spectroscopy.

5. ^{1}H noesygpph vs ^{15}N hsqcetgpnosp

To estimate the transverse relaxation time T_2 from the linewidth of an 1D NMR spectrum, we use the following relationship:

$$T_2 = \frac{1}{\pi \Delta v_{1/2}} \qquad (1)$$

Where $\Delta v_{1/2}$ is the full width at half maximum of the resonance peak in Hz.
The 1D ^{1}H and ^{15}N spectra of the sample containing TRIFP **1a**/**2a**/HE **3b** were used to obtain the $\Delta v_{1/2}$ of the spins involved in the hydrogen bond (see SI Table 7). The data are showed in the Table 1 with the respective T2 relaxation times. For the three signals evaluated CPA/Z, CPA/E and CPA/HE the ^{15}N T2 relaxation are longer than the ^{1}H T2.

Table S7. Calculated T2 relaxation for the ^{1}H and ^{15}N hydrogen bond signal using the spectral data at 180K (see SI 6.2 and the equation 1.

Hydrogen bonding Signal	^{1}H signal		^{15}N signal	
	$\Delta v_{1/2}$ / Hz	T2 / ms	$\Delta v_{1/2}$/ Hz	T2 / ms
CPA/Z	113	2.8	26	12.2
CPA/E	121	2.6	27	11.9
CPA/HE	189	1.7	14	23.3

On 2D NMR experiments, the increment delay (IN_F) is dependent of the spectral width (SW) of the indirect dimension (SWF_{F1}) as equation 2.

$$IN\_F = 1/SWF_{F1} \qquad (2)$$

In the pulse sequence ^{15}N hsqcetgpnosp (see SI 6.5), the longest evolution time (τ_1) is determined as equation 3.

$$\tau_{1\,HSQC-NOESY} = 3\mu s + \left(TDF1 * \frac{IN\_F}{2}\right) \qquad (3)$$

Where $TDF1$ is the number of points to be acquired to generate the FID signal.
In the pulse sequence of ^{1}H noesygpph (see SI 6.6), the longest evolution time ($\tau_{1\,NOESY}$) is determined as:

$$\tau_{1\,NOESY} = TDF1 * \left(\frac{IN\_F}{2} - \frac{P1 * 4}{\pi}\right) \qquad (4)$$

Where $P1$ is the calibrated 90° pulse length for ^{1}H.

With the T2 Relaxation showed on table 2 for CPA/HE signals and equation 3 and 4, is possible to visualize the increase rate of τ (evolution time) for different spectral width (SW) for the ^{1}H noesygpph and ^{15}N hsqcetgpnosp pulse sequences (see Figure 36a and 36b).
For each τ progression (evolution time), there is a loss of the stored magnetization at M_{xy} due T2 relaxation time, if we consider the T2 relaxation as a monoexponential decay, we have equation 5.

$$M_{xy} = 1\left(e^{\frac{-\tau}{T2}}\right) \qquad (5)$$

Applying the evolution time for each Increment at equation 5, we obtain the Relative stored magnetization at M_{xy} at the end of a determined evolution period.
The sample containing TRIFP **1a**/**2a**/HE **3b** has specific T2 relaxation characteristics. Taking the signal of CPA/HE as example, ^{1}H T2 = 1.7ms and ^{15}N T2 = 23.3ms (see Table 1). Using these values in equation 5 with the respective evolution times for ^{1}H noesygpph and ^{15}N hsqcetgpnosp, we obtain the relative stored magnetization at M_{xy} for each evolution period for different spectral width (SW) (see Figure 36c and 36d).

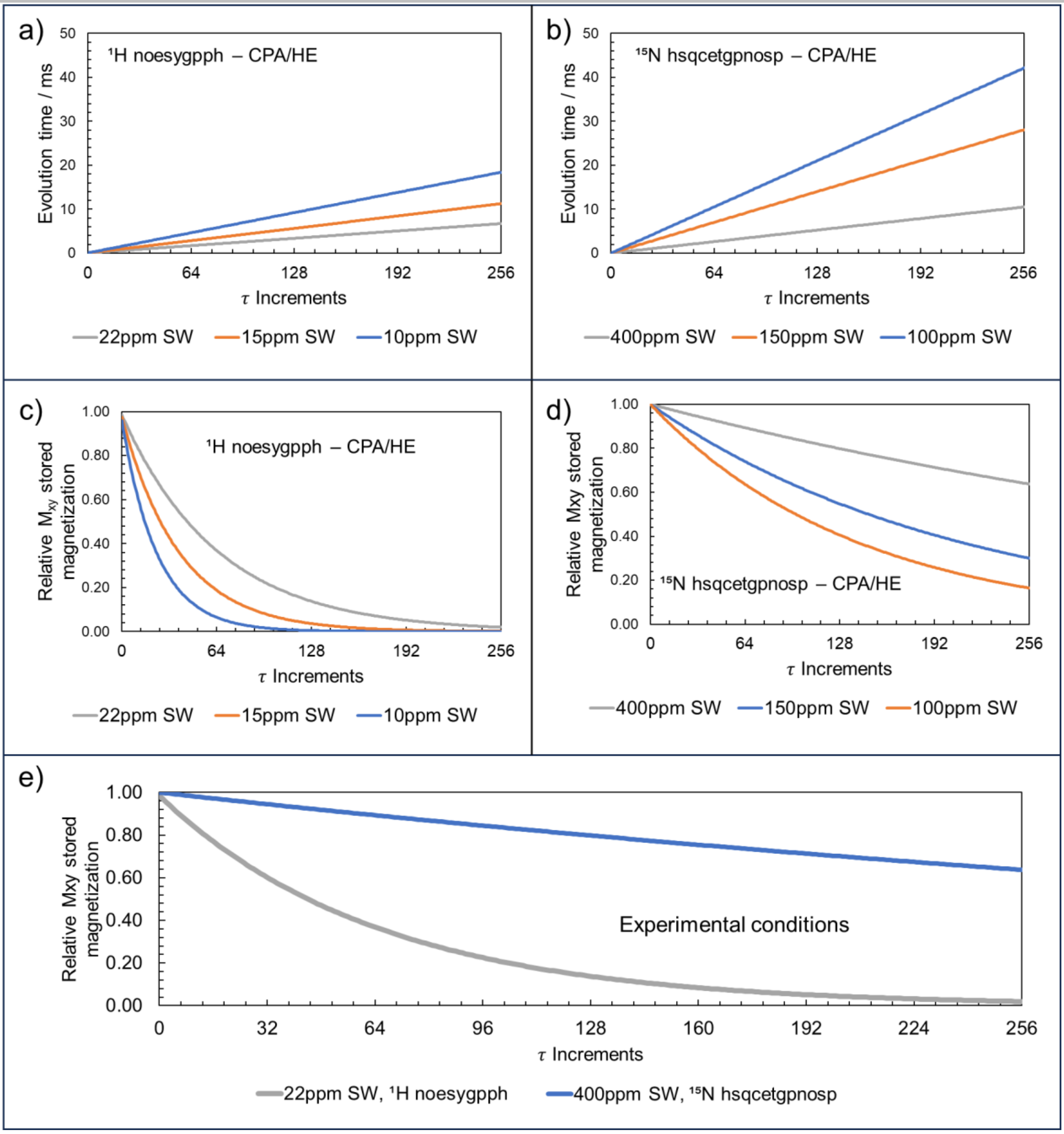

Figure S 36. Calculated evolution time (τ) dependence of the number of τ increments for different spectral widths (SW) for the hydrogen bond CPA/HE. Panels show: (a) ^{1}H noesygpph and (b) ^{15}N hsqcetgpnosp. The calculated relative stored magnetization at Mxy as a function of τ progression for the hydrogen bond CPA/HE is shown for ^{1}H noesygpph and (b) ^{15}N hsqcetgpnosp. for different SW. Panel (e) compares the experimental acquisition conditions for the hydrogen bond CPA/HE between ^{1}H noesygpph and ^{15}N hsqcetgpnosp.

Under the specific condition of the sample TRIFP **1a**/**2a**/HE **3b**, the 2D ^{15}N hsqcetgpnosp results in better sensitivity than the^{1}H noesygpph, which is contradictory when compared with the ^{1}H magnetic susceptibility relative to ^{15}N.

In the Figure 32c and 32d, the calculated dependence of relative stored magnetization at M_{xy} as a function of τ progression for the hydrogen bond CPA/HE is showed. It is evident that increasing the spectral resolution (by decreasing the spectral width) leads to a loss of stored magnetization.

Figure 32e compares the experimental conditions of both pulse sequences ^{1}H noesygpph and ^{15}N hsqcetgpnosp for the CPA/HE hydrogen bond, demonstrates that at the end of each τ period, the magnetization in the xy plane is significantly stronger in ^{15}N hsqcetgpnosp compared to ^{1}H noesygpph. This explains the unusual bigger sensitivity observed in the ^{15}N hsqcetgpnosp than in the ^{1}H noesygpph (see Figure S37).

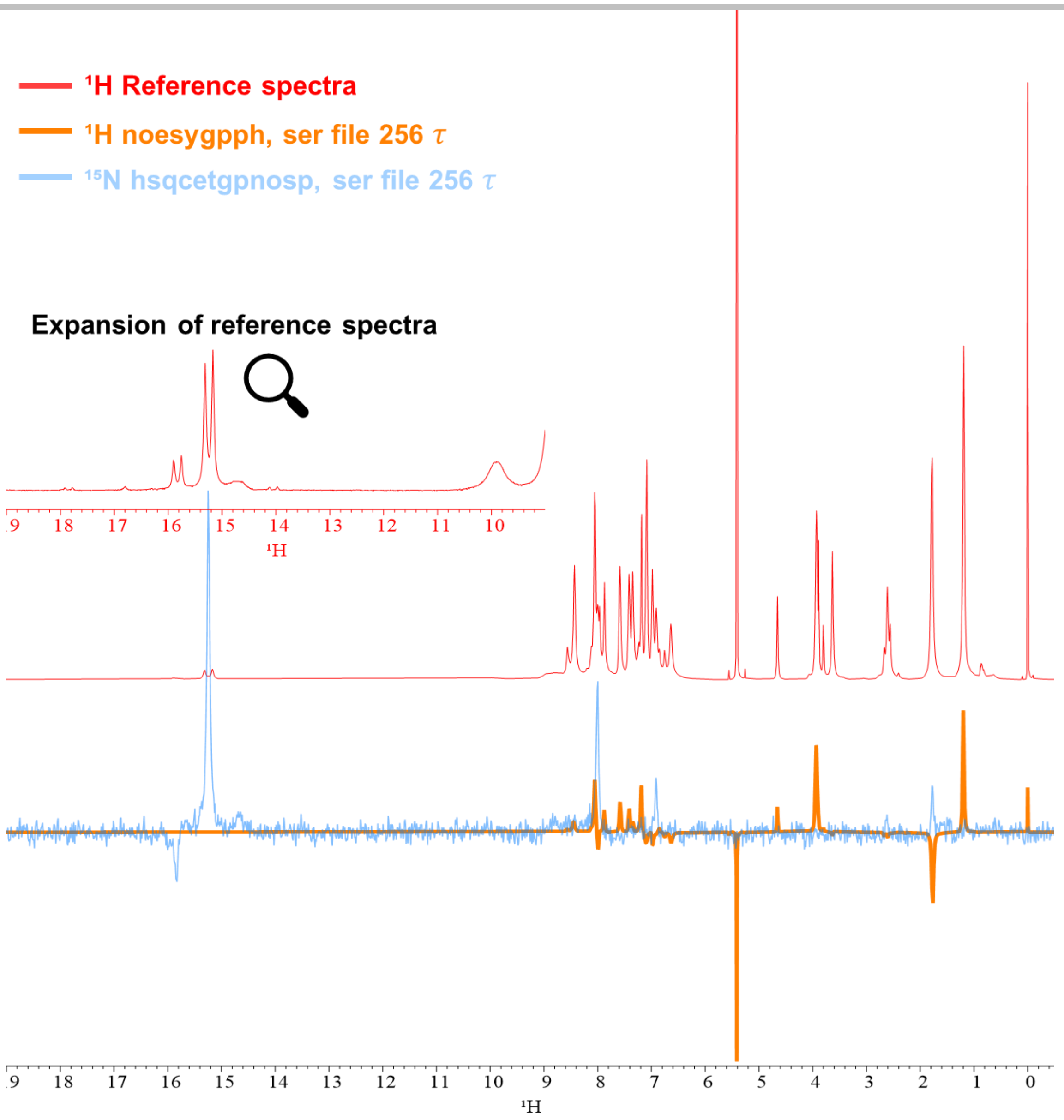

Figure S 37. Comparison of ser file after 256 τ increments of ^{15}N hsqcetgpnosp and ^{1}H noesygpph of the sample contents TRIFP 1a/2a/HE 3b at 180 K. The noesygpph experiment was acquired with 24 scans, SW F1 and F2 = 22.0 ppm, mixing time equal to 0.1 s, receiver gain = XXX, ^{1}H preamplifier 100w. The ^{15}N hsqcetgpnosp experiment was acquired with 8 scans, SW F1 = 400 ppm ND SW F2= 22.0, mixing time equal to 0.080 s, receiver gain = 1820, ^{1}H preamplifier 100w and ^{15}N preamplifier 300W.

6. Appendix

6.1. Screening – ^{1}H- NMR Spectrum of each sample

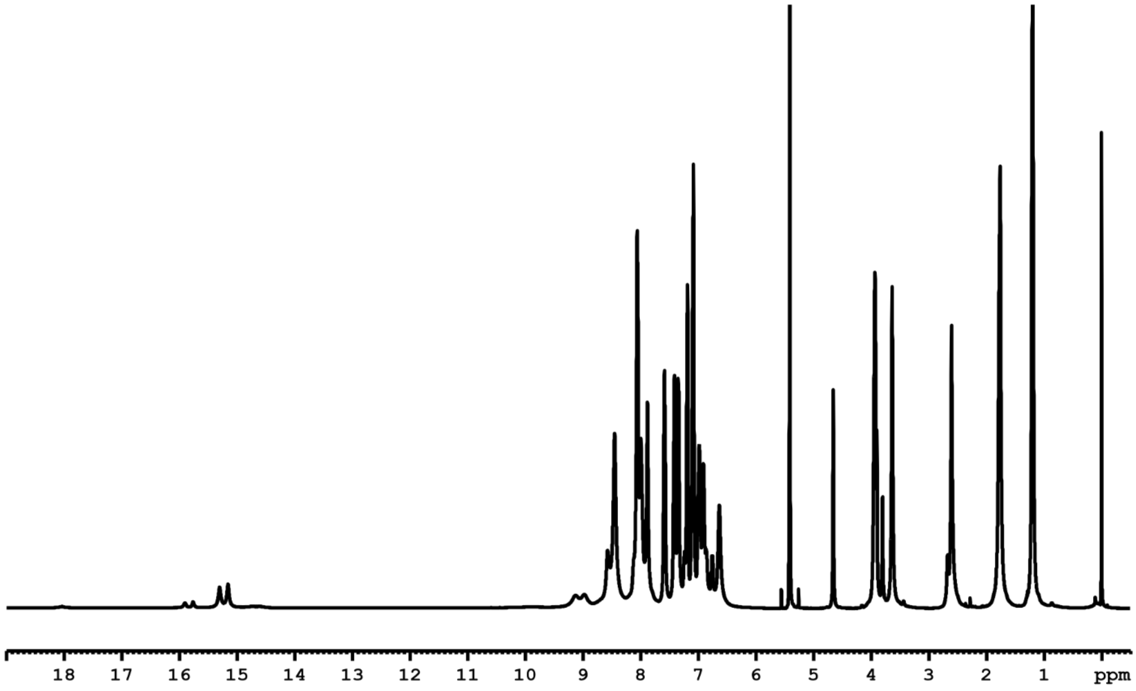

^{1}H-spectrum of the ternary complex of TRIFP **1a/2a/HE 3b** (1:1:1 stoichiometry, 600MHz, CD_2Cl_2, 180K).

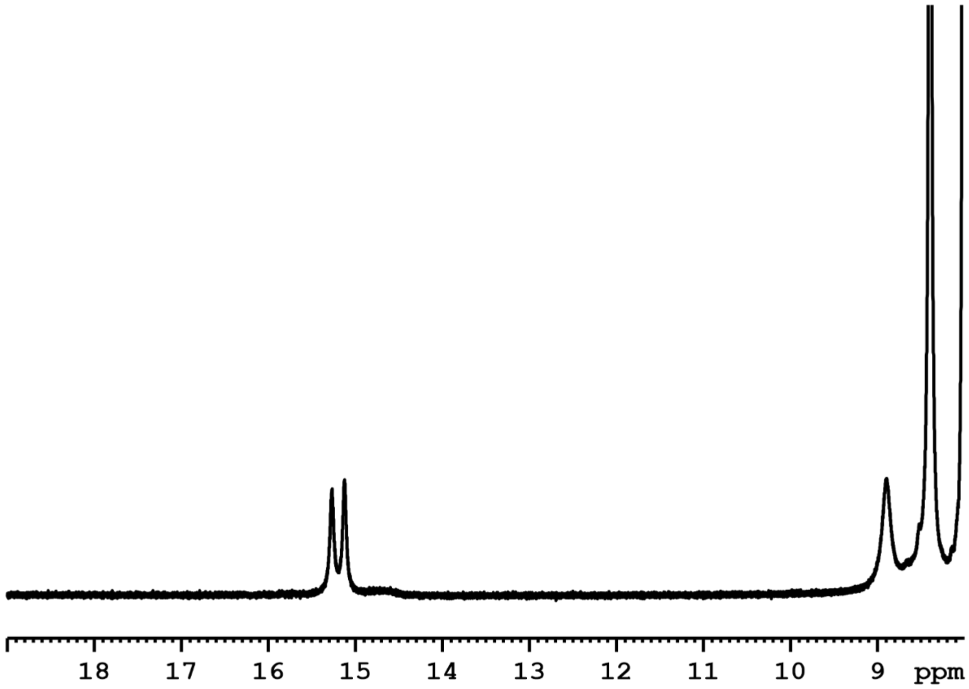

E-only sample: Hydrogen bond area of the ^{1}H-spectrum of the ternary complex of **F 1a/*E*-2a/HE 3b** (1:1:1 stoichiometry, 600MHz, CD_2Cl_2, 180K).

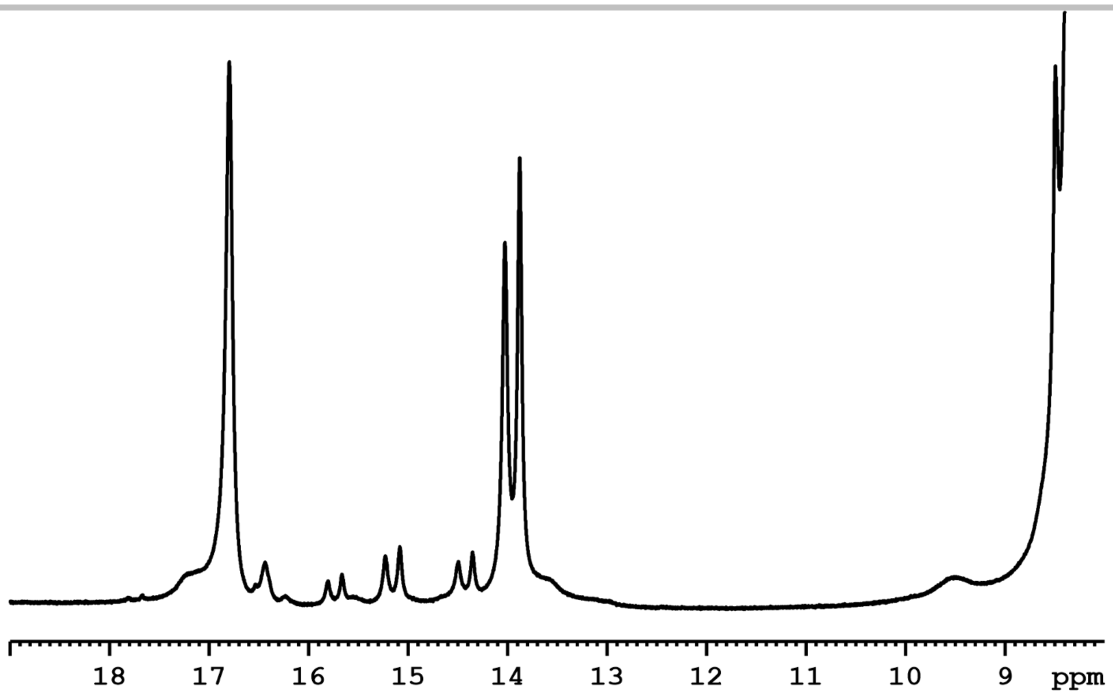

Hydrogen bond area of the ^{1}H-spectrum of the ternary complex of TRIFP **1a/2a/HE 3b** (2:1:1 stoichiometry, 600MHz, CD_2Cl_2, 180K).

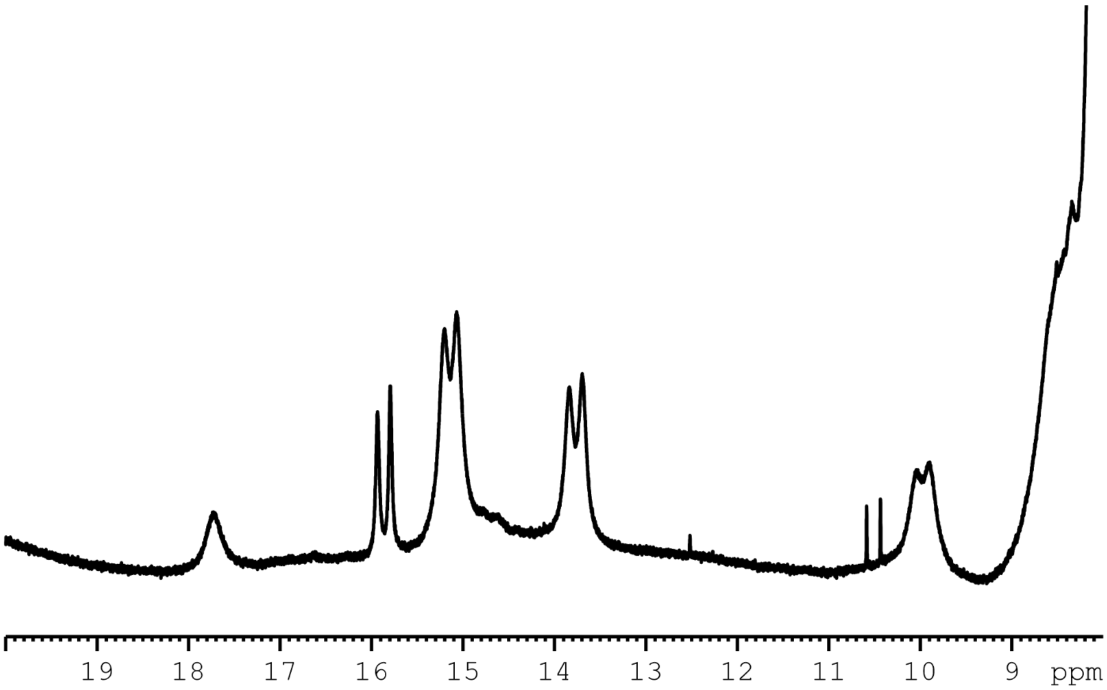

Hydrogen bond area of the ^{1}H-spectrum of the ternary complex of OMe-CPA **1b/2a/HE 3b** (1:1:1 stoichiometry, 600MHz, CD_2Cl_2, 180K).

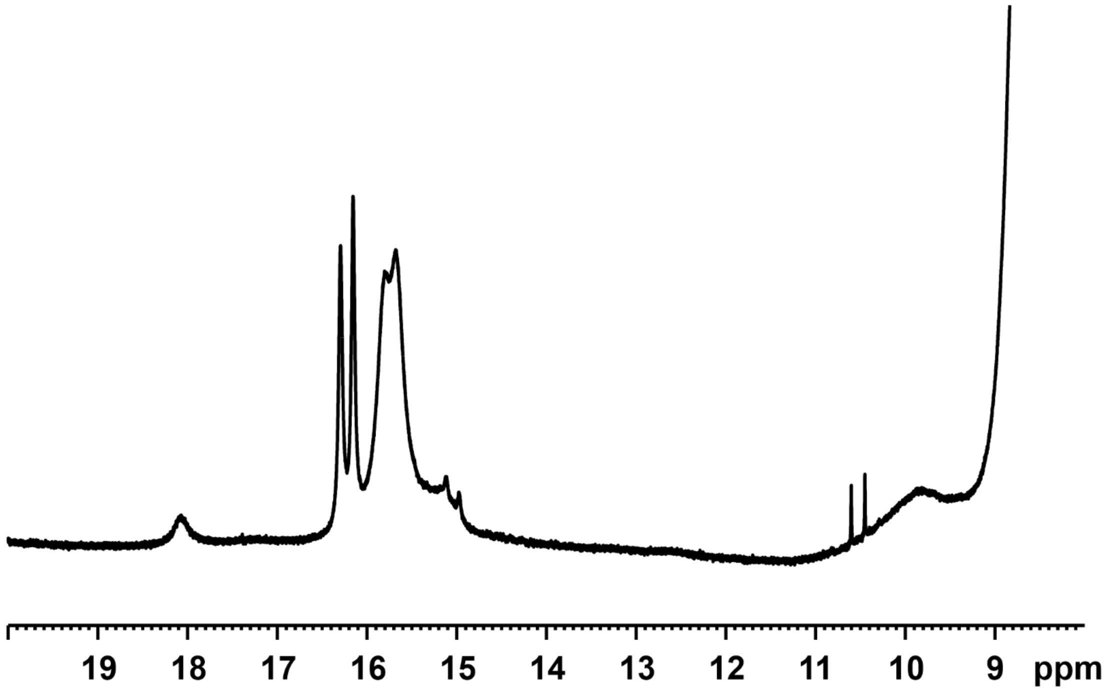

Hydrogen bond area of the ^{1}H-spectrum of the ternary complex of TRIFP **1a/2b/HE 3b** (1:1:1 stoichiometry, 600MHz, CD_2Cl_2, 180K).

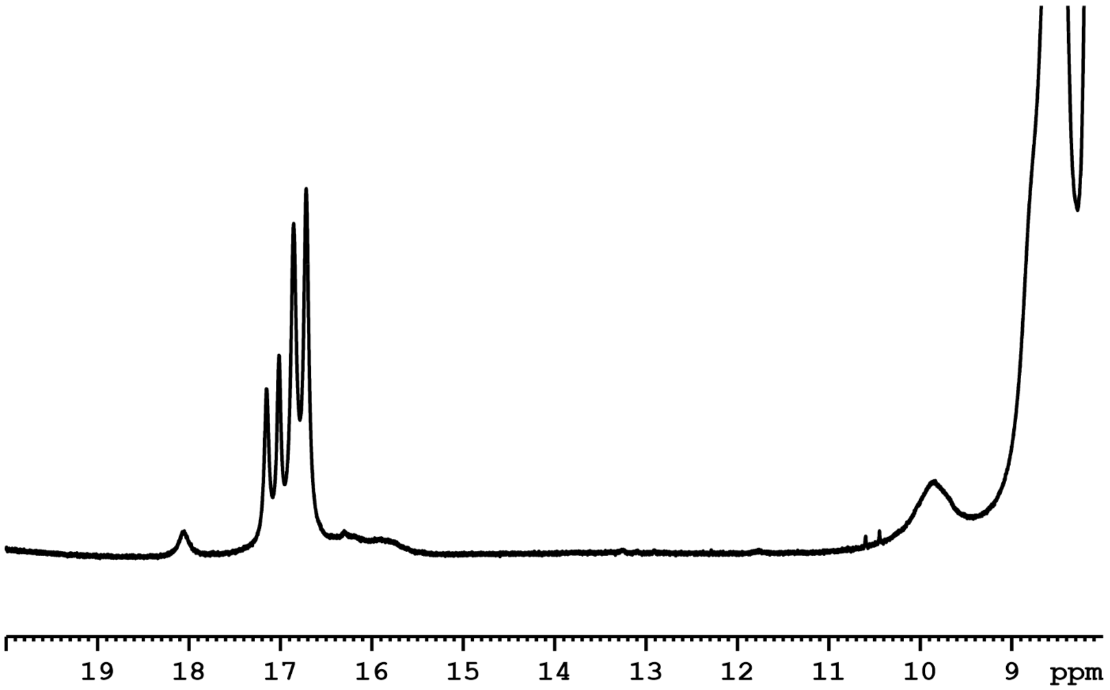

Hydrogen bond area of the ^{1}H-spectrum of the ternary complex of TRIFP **1a/2c/HE 3b** (1:1:1 stoichiometry, 600MHz, CD_2Cl_2, 180K).

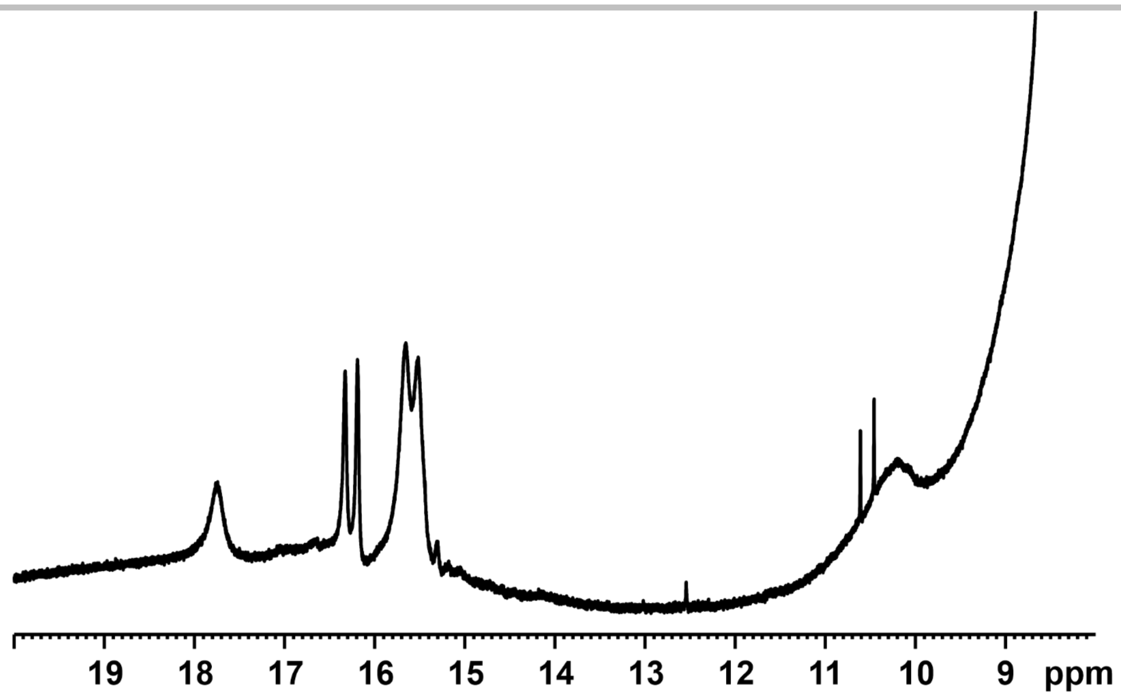

Hydrogen bond area of the ^{1}H-spectrum of the ternary complex of OMe -CPA **1b/2b/HE 3b** (1:1:1 stoichiometry, 600MHz, CD_2Cl_2, 180K).

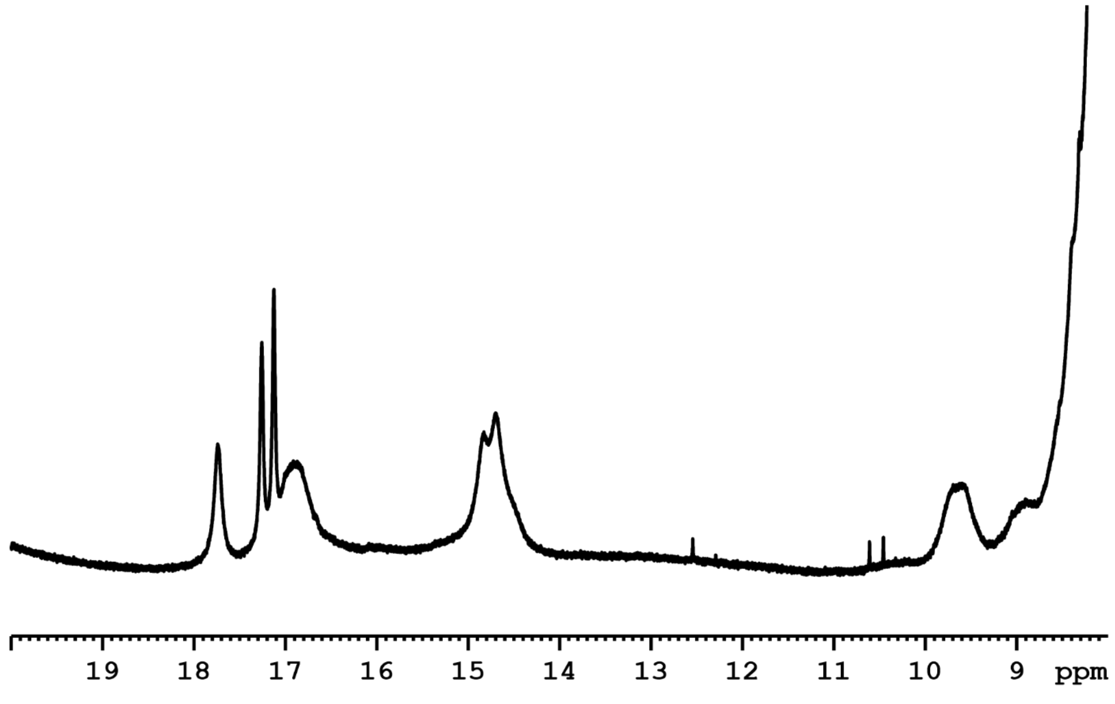

Hydrogen bond area of the ^{1}H-spectrum of the ternary complex of OMe -CPA **1b/2c/HE 3b** (1:1:1 stoichiometry, 600MHz, CD_2Cl_2, 180K).

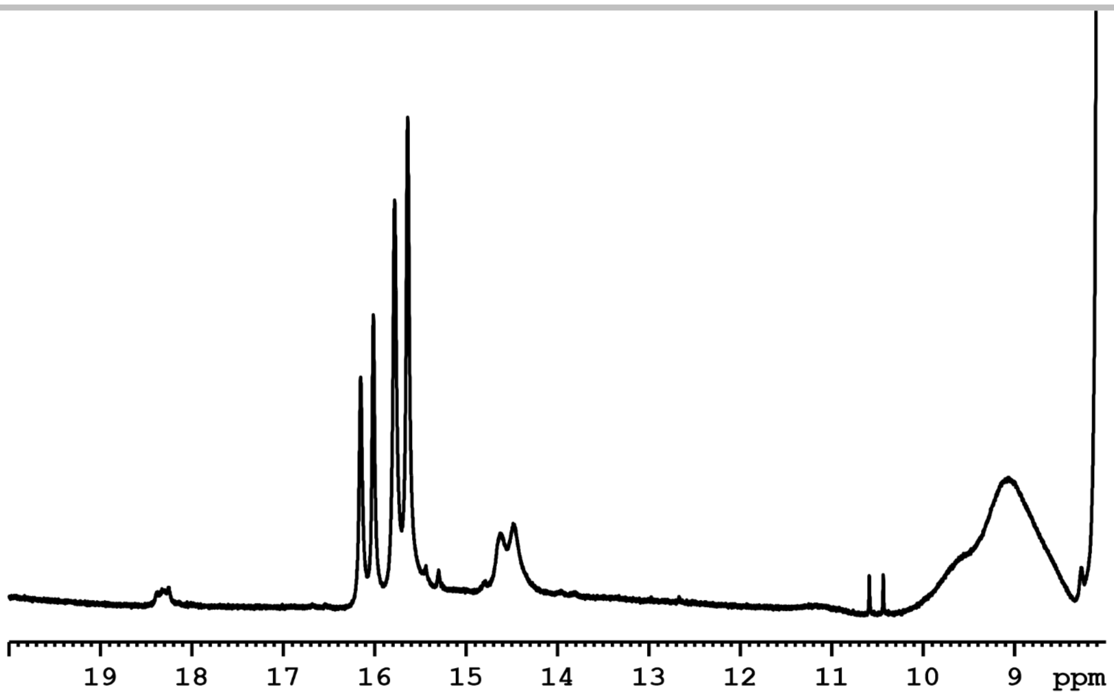

Hydrogen bond area of the ^{1}H-spectrum of the ternary complex of TRIM **1c/2a/HE 3b** (1:1:1 stoichiometry, 600MHz, CD_2Cl_2, 180K).

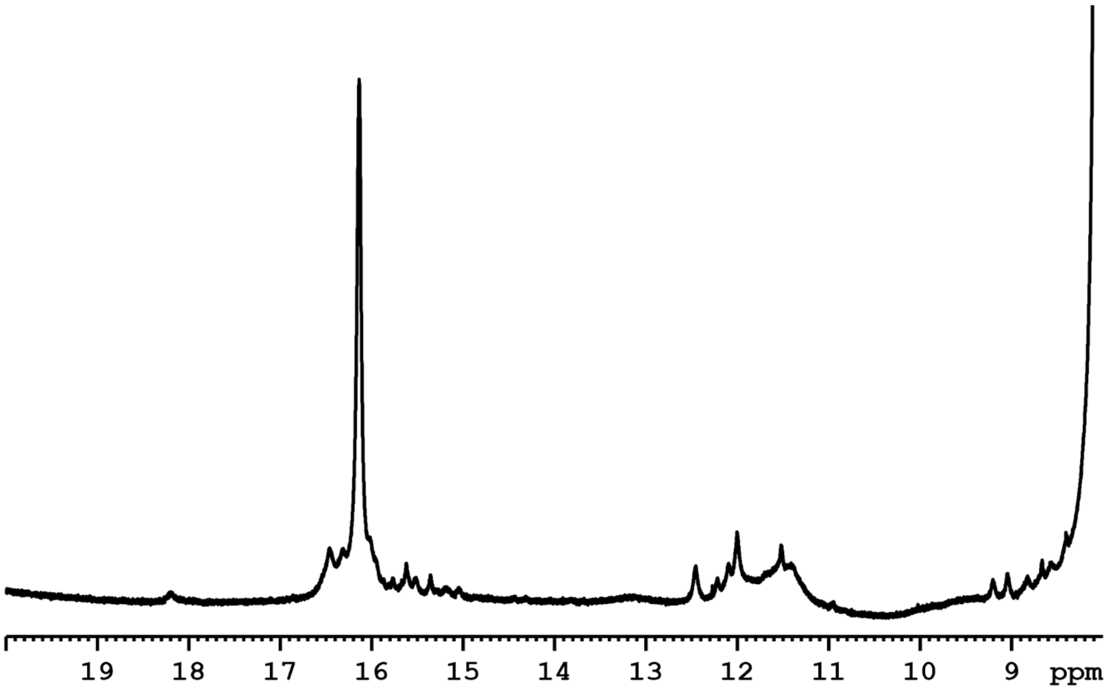

Hydrogen bond area of the ^{1}H-spectrum of the ternary complex of TRIM **1c/2b/HE 3b** (1:1:1 stoichiometry, 600MHz, CD_2Cl_2, 180K).

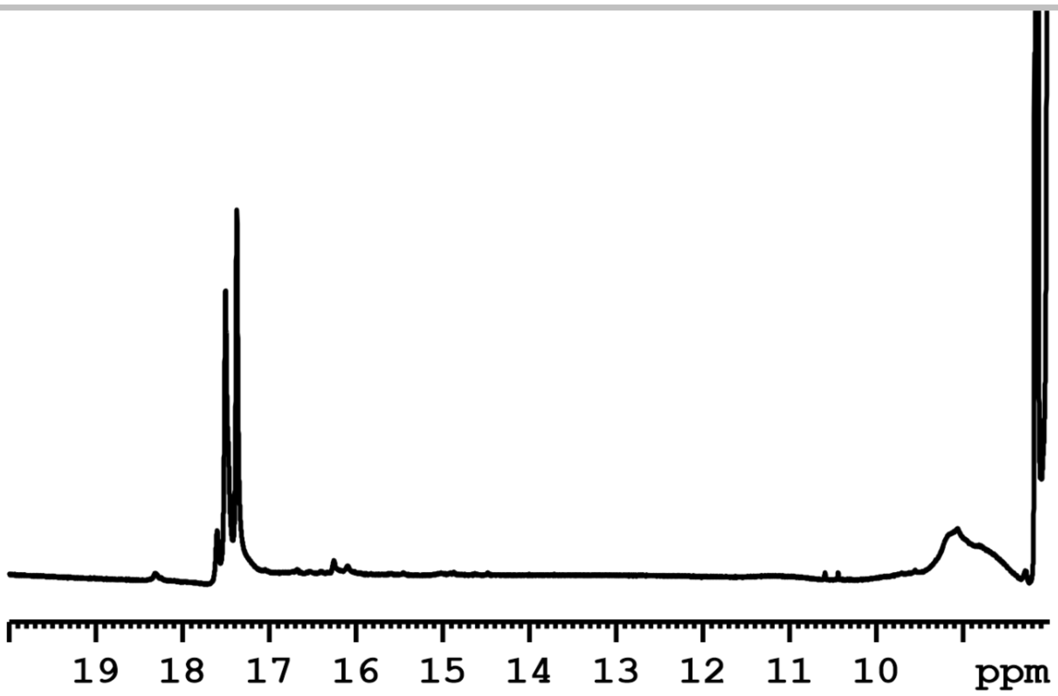

Hydrogen bond area of the ^{1}H-spectrum of the ternary complex of TRIM **1c/2c/HE 3b** (1:1:1 stoichiometry, 600MHz, CD_2Cl_2, 180K).

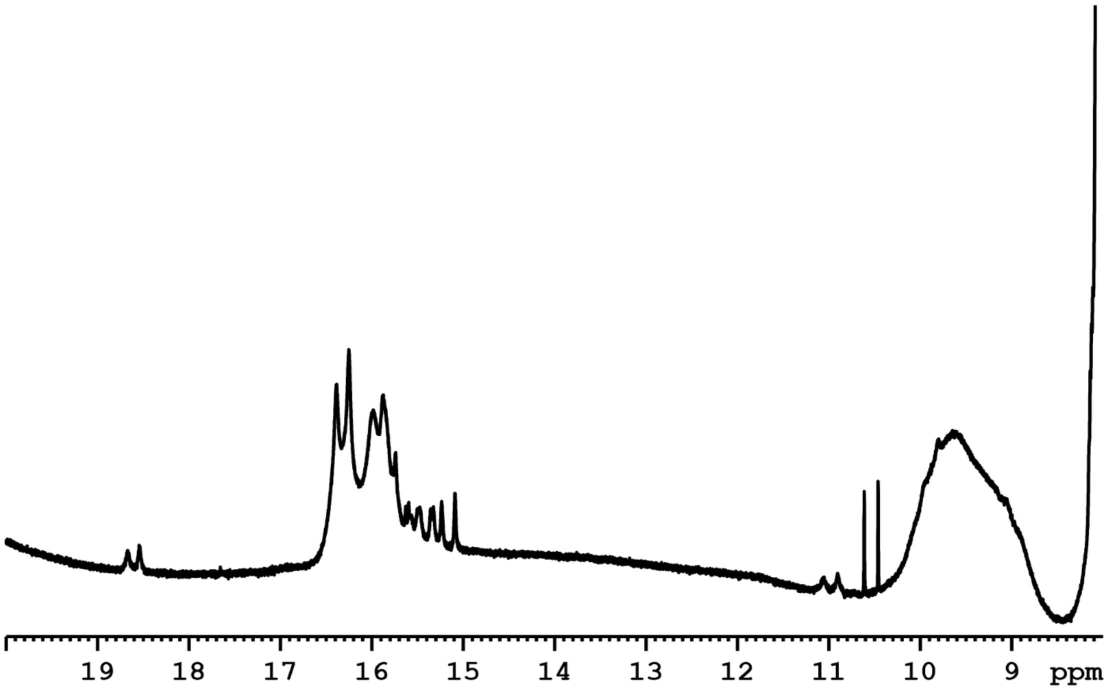

Hydrogen bond area of the ^{1}H-spectrum of the ternary complex of TRIP **1d/2a/HE 3b** (1:1:1 stoichiometry, 600MHz, CD_2Cl_2, 180K).

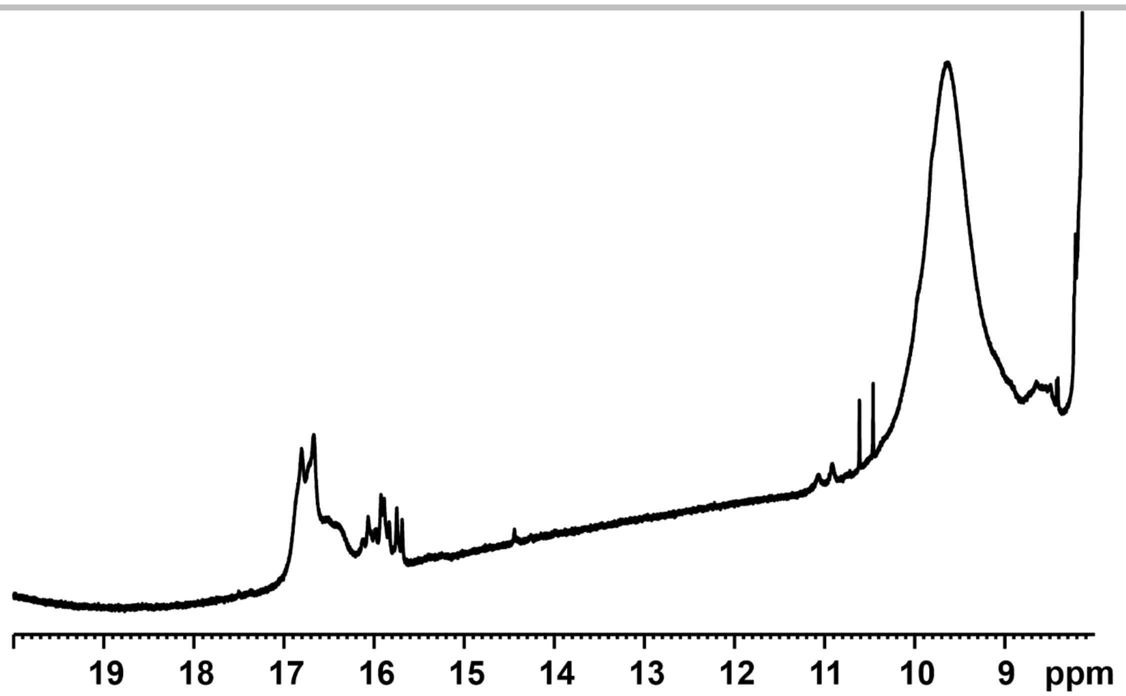

Hydrogen bond area of the ^{1}H-spectrum of the ternary complex of TRIP **1d/2b/HE 3b** (1:1:1 stoichiometry, 600MHz, CD_2Cl_2, 180K).

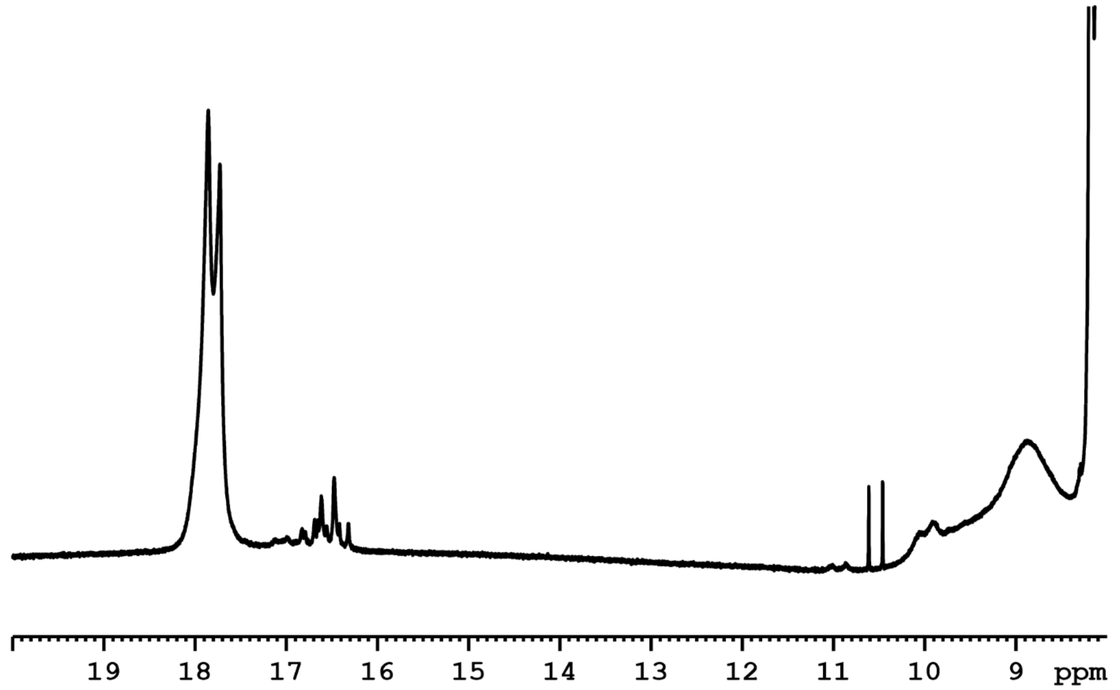

Hydrogen bond area of the ^{1}H-spectrum of the ternary complex of TRIP **1d/2c/HE 3b** (1:1:1 stoichiometry, 600MHz, CD_2Cl_2, 180K).

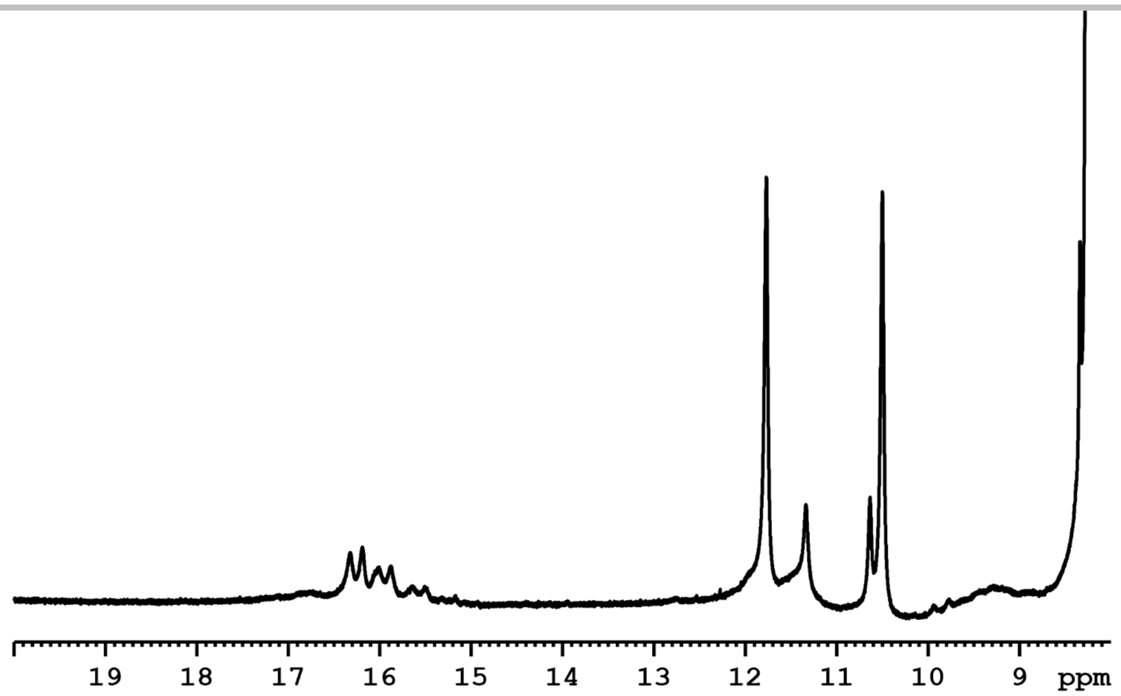

Hydrogen bond area of the ^{1}H-spectrum of the ternary complex of TiPSY **1e/2b/HE 3b** (1:1:1 stoichiometry, 600MHz, CD_2Cl_2, 180K).

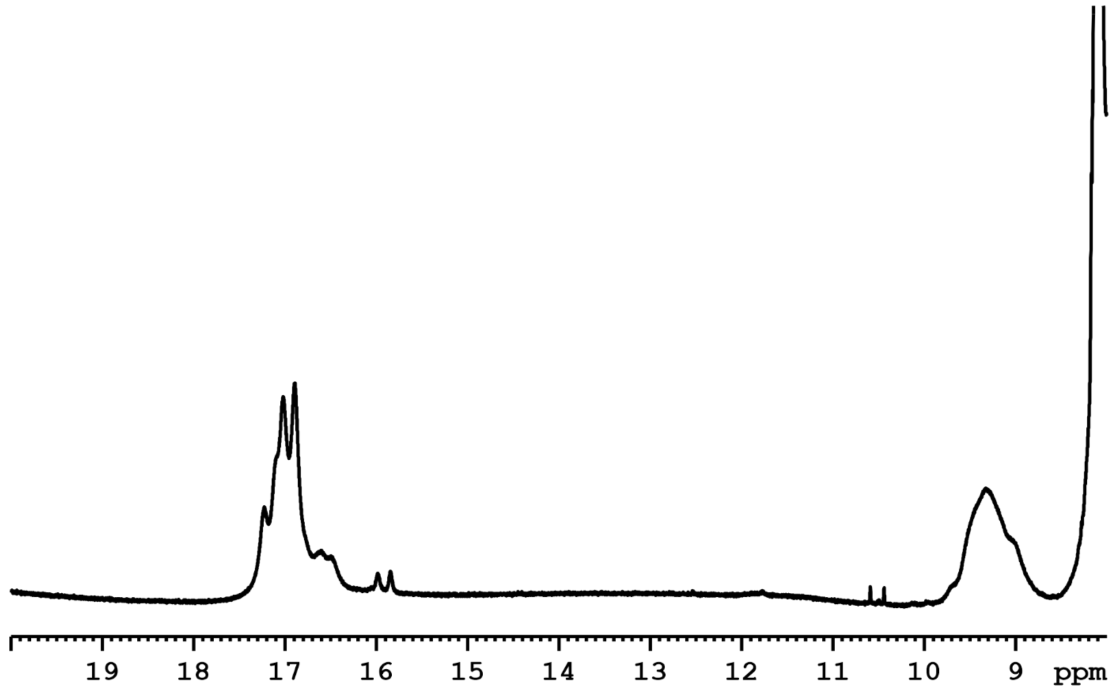

Hydrogen bond area of the ^{1}H-spectrum of the ternary complex of TiPSY **1e/2c/HE 3b** (1:1:1 stoichiometry, 600MHz, CD_2Cl_2, 180K).

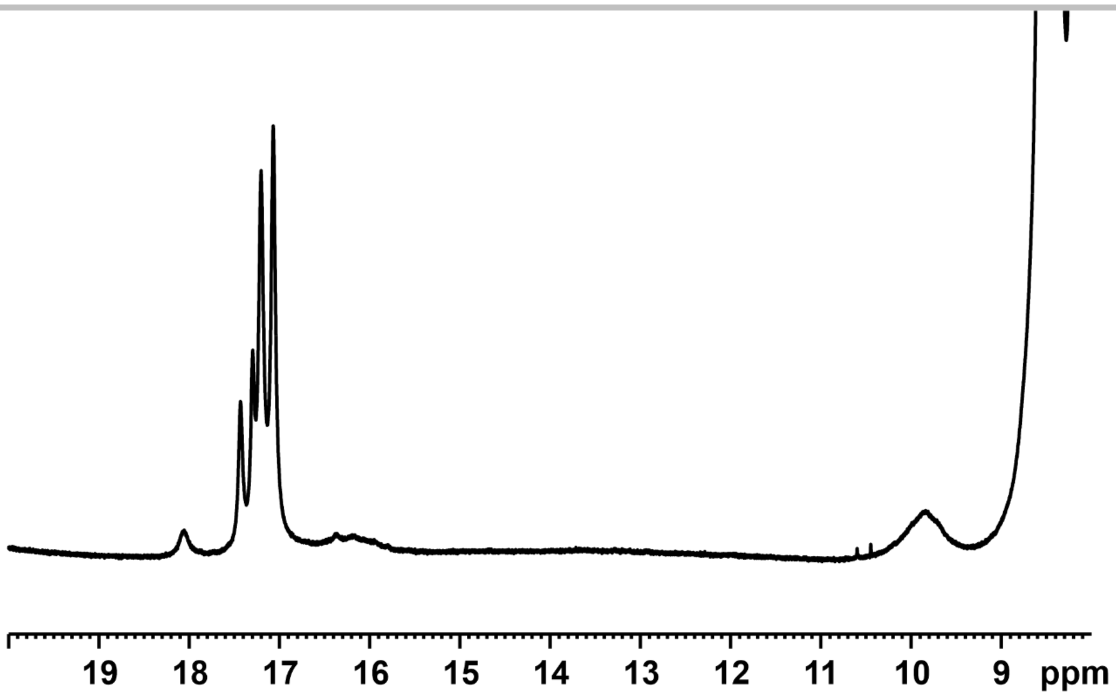

Hydrogen bond area of the ^{1}H-spectrum of the ternary complex of **1a/2d/HE 3b** (1:1:1 stoichiometry, 600MHz, CD_2Cl_2, 180K).

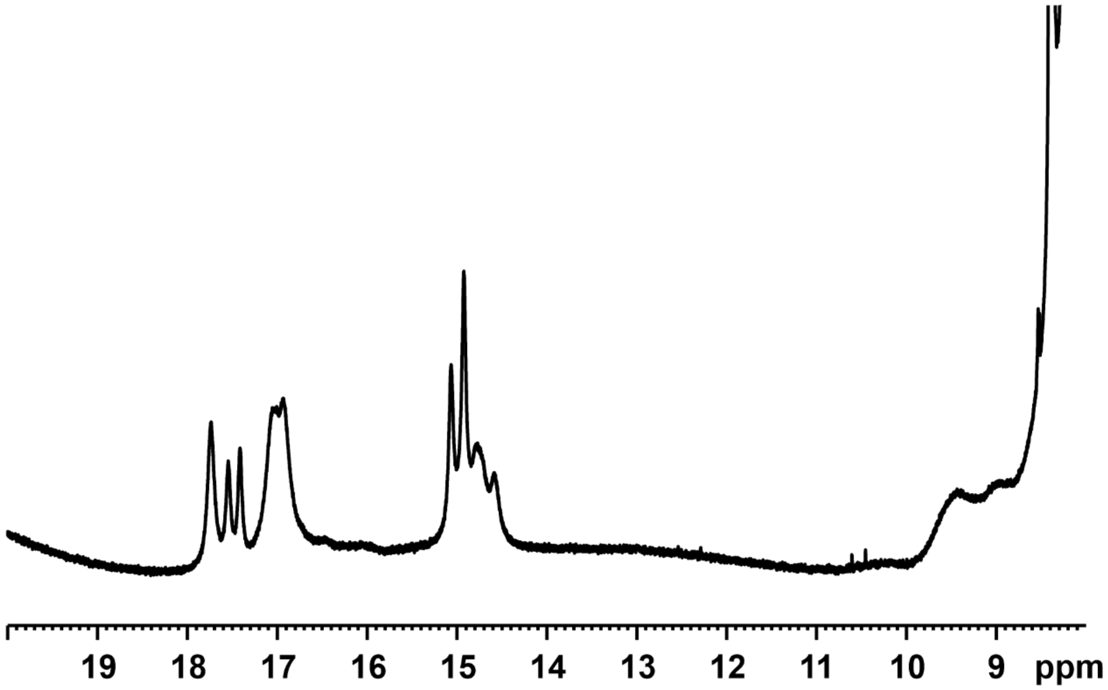

Hydrogen bond area of the ^{1}H-spectrum of the ternary complex of **1b/2d/HE 3b** (1:1:1 stoichiometry, 600MHz, CD_2Cl_2, 180K).

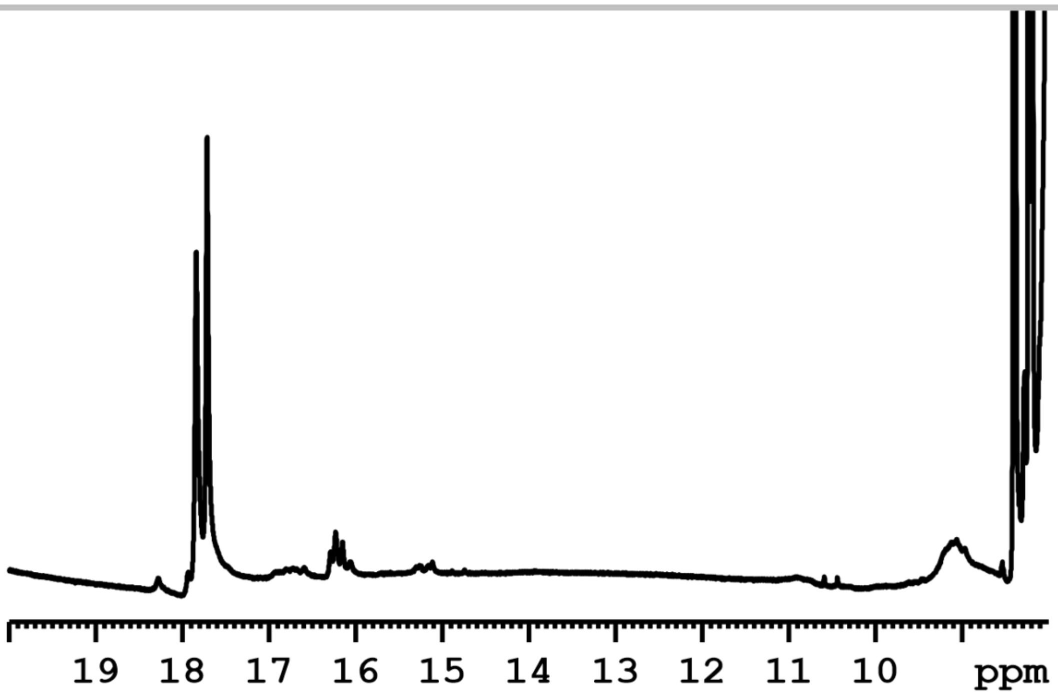

Hydrogen bond area of the ^{1}H-spectrum of the ternary complex of **1c/2d/HE 3b** (1:1:1 stoichiometry, 600MHz, CD_2Cl_2, 180K).

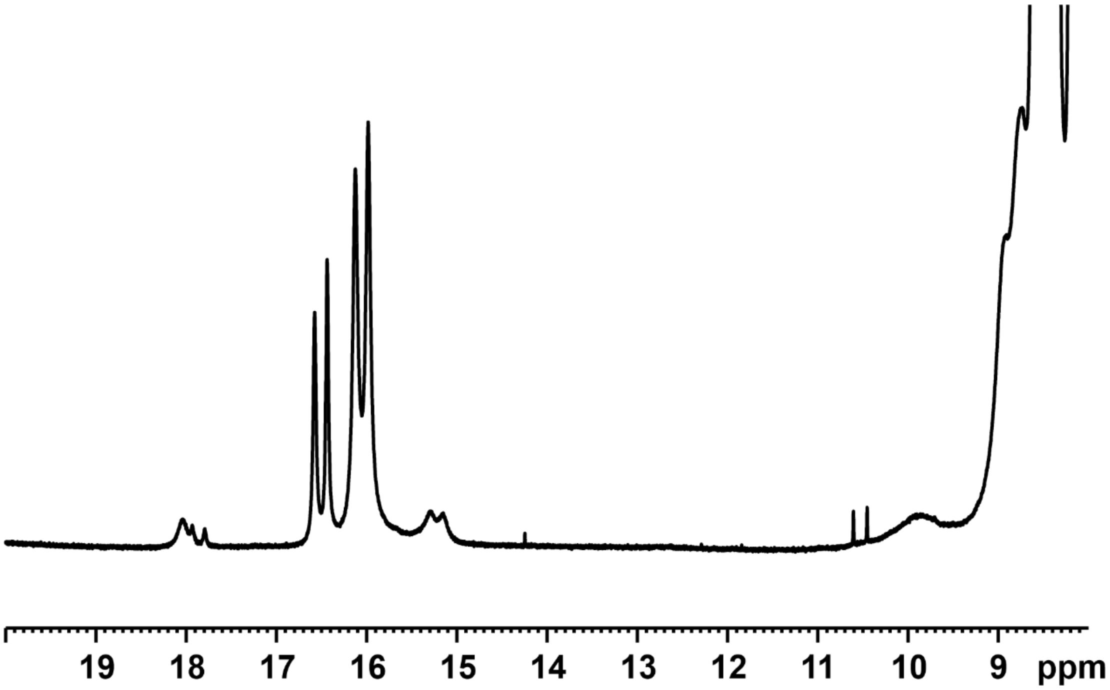

Hydrogen bond area of the ^{1}H-spectrum of the ternary complex of **1a/2e/HE 3b** (1:1:1 stoichiometry, 600MHz, CD_2Cl_2, 180K).

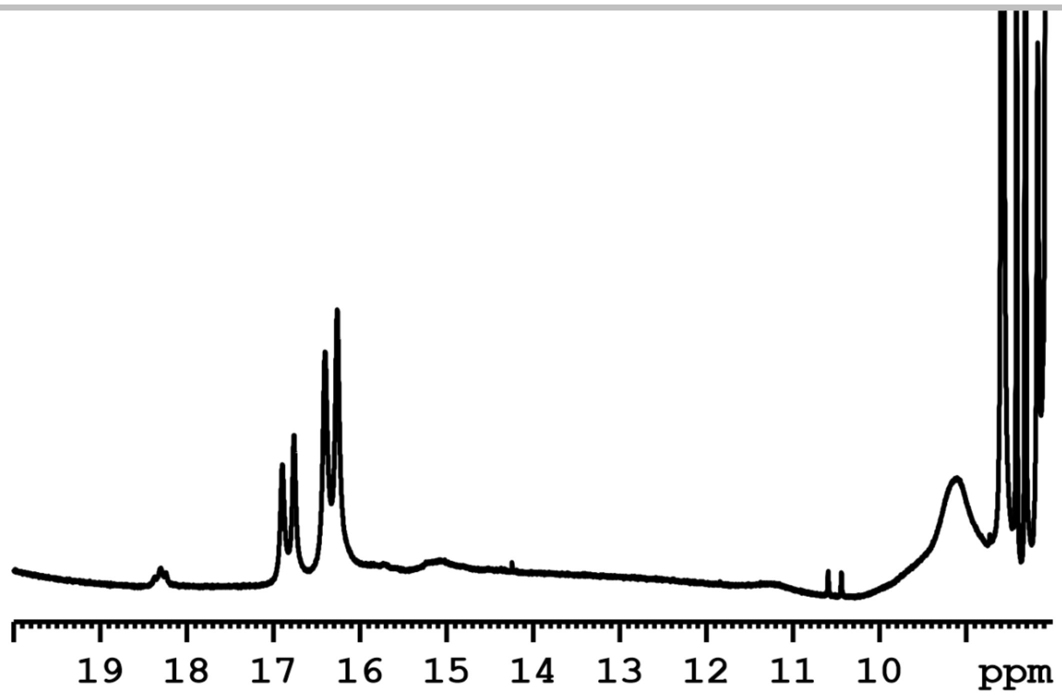

Hydrogen bond area of the ^{1}H-spectrum of the ternary complex of **1c/2e/HE 3b** (1:1:1 stoichiometry, 600MHz, CD_2Cl_2, 180K).

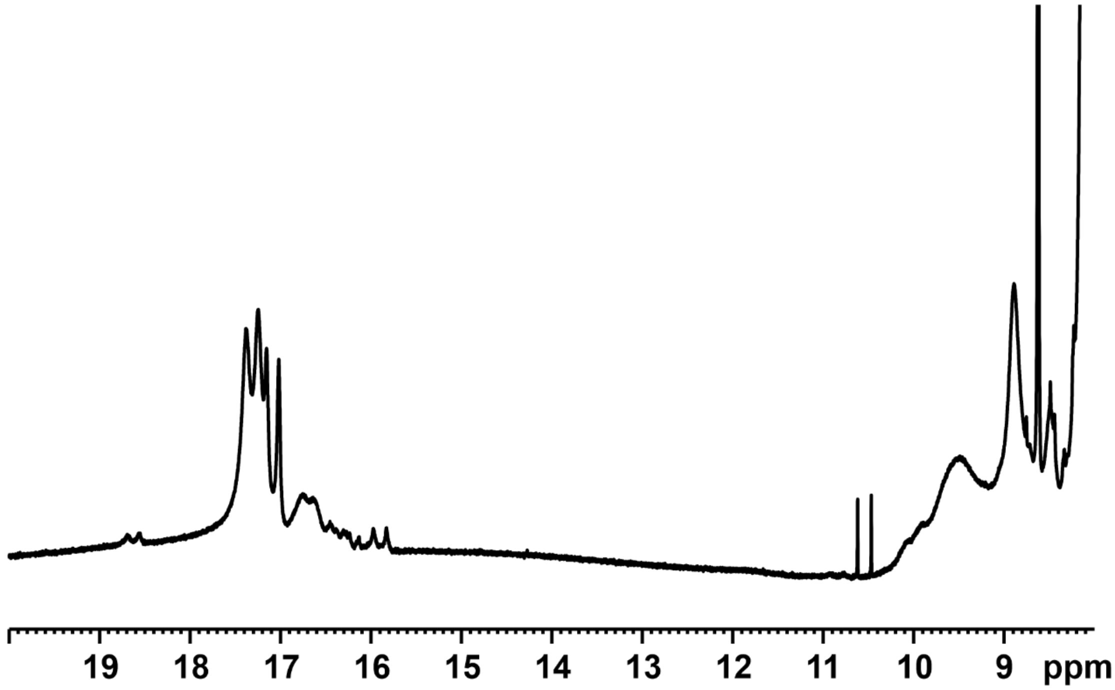

Hydrogen bond area of the ^{1}H-spectrum of the ternary complex of **1d/2e/HE 3b** (1:1:1 stoichiometry, 600MHz, CD_2Cl_2, 180K).

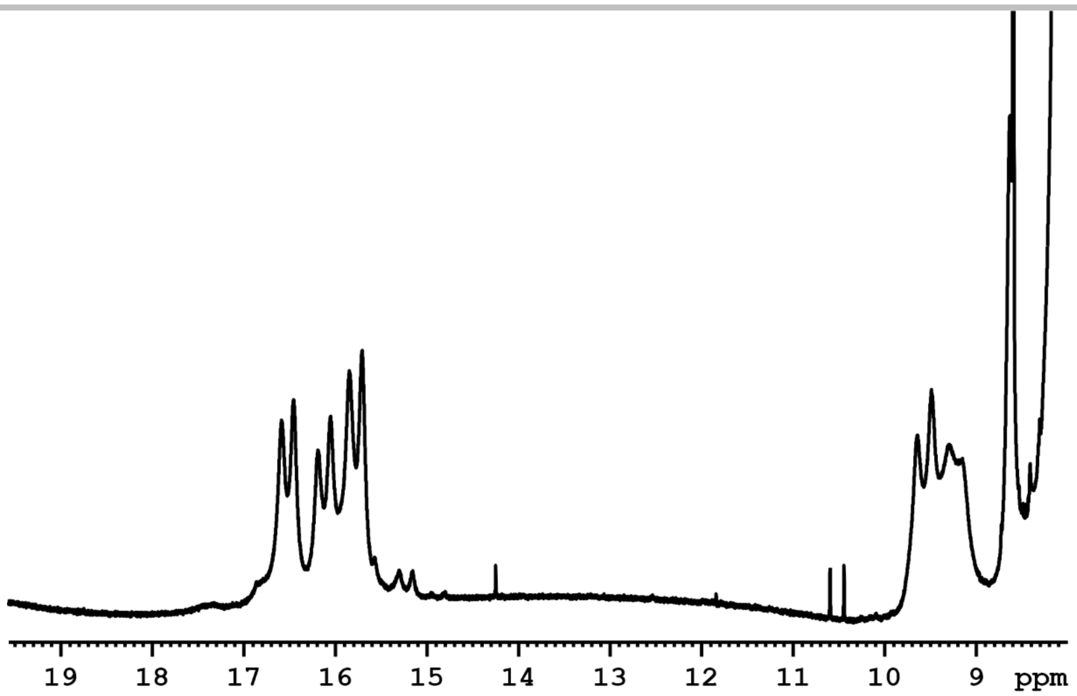

Hydrogen bond area of the ^{1}H-spectrum of the ternary complex of **1e/2e/HE 3b** (1:1:1 stoichiometry, 600MHz, CD_2Cl_2, 180K).

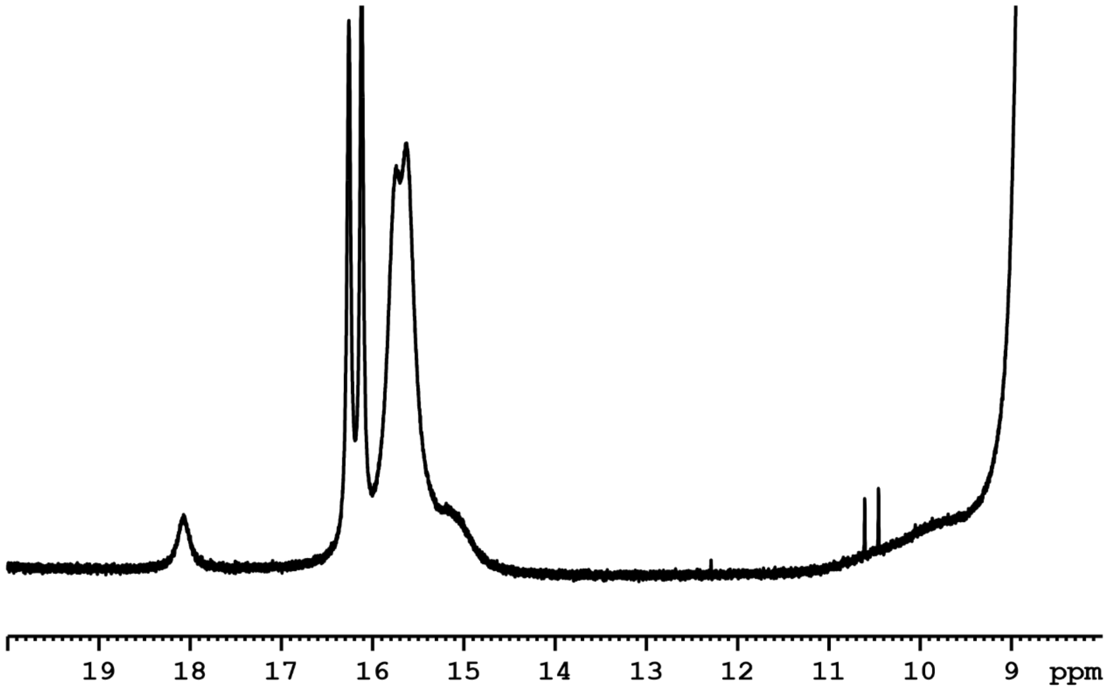

Hydrogen bond area of the ^{1}H-spectrum of the ternary complex of **1a/2f/HE 3b** (1:1:1 stoichiometry, 600MHz, CD_2Cl_2, 180K).

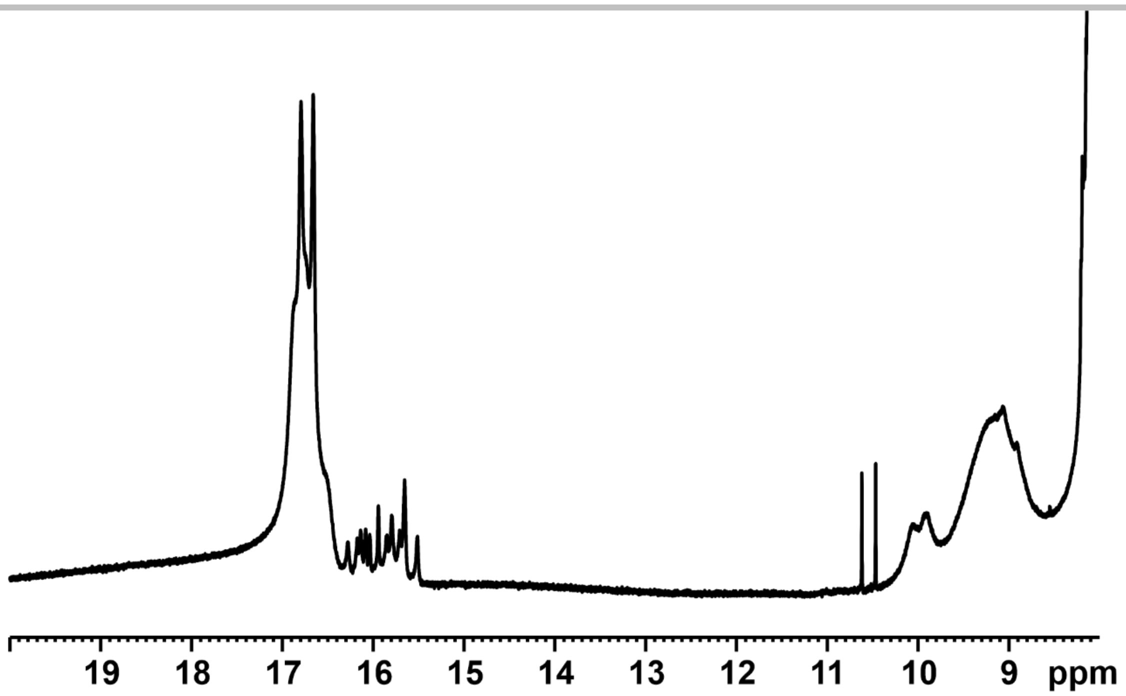

Hydrogen bond area of the ^{1}H-spectrum of the ternary complex of **1d**/**2f**/HE **3b** (1:1:1 stoichiometry, 600MHz, CD_2Cl_2, 180K).

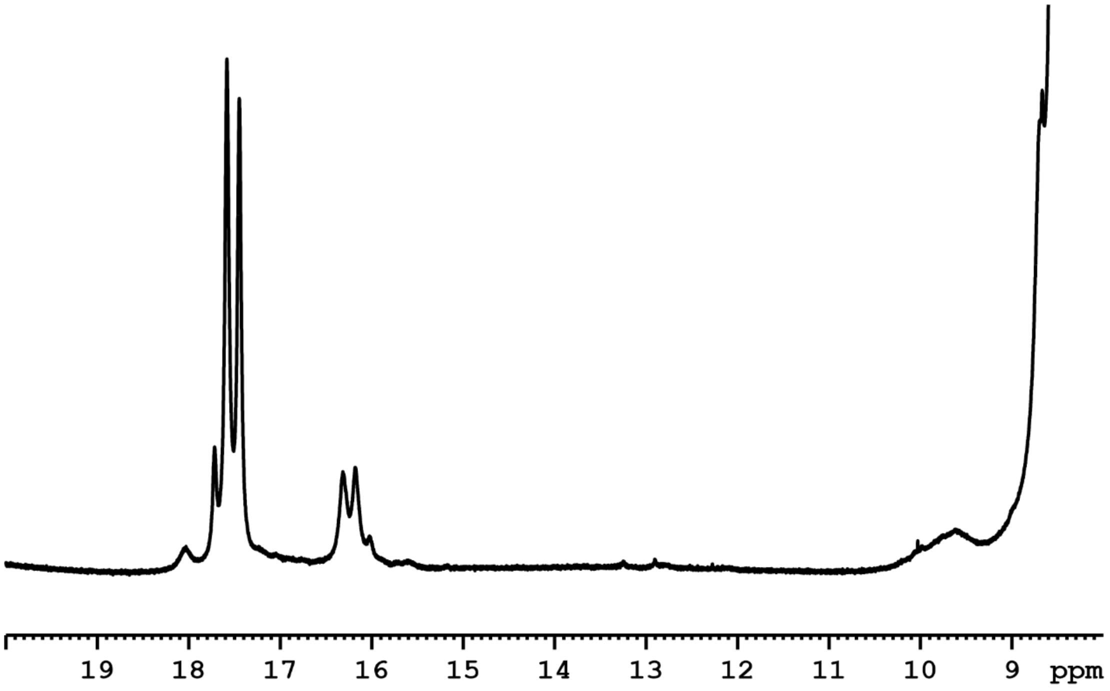

Hydrogen bond area of the ^{1}H-spectrum of the ternary complex of **1a/2g/HE 3b** (1:1:1 stoichiometry, 600MHz, CD_2Cl_2, 180K).

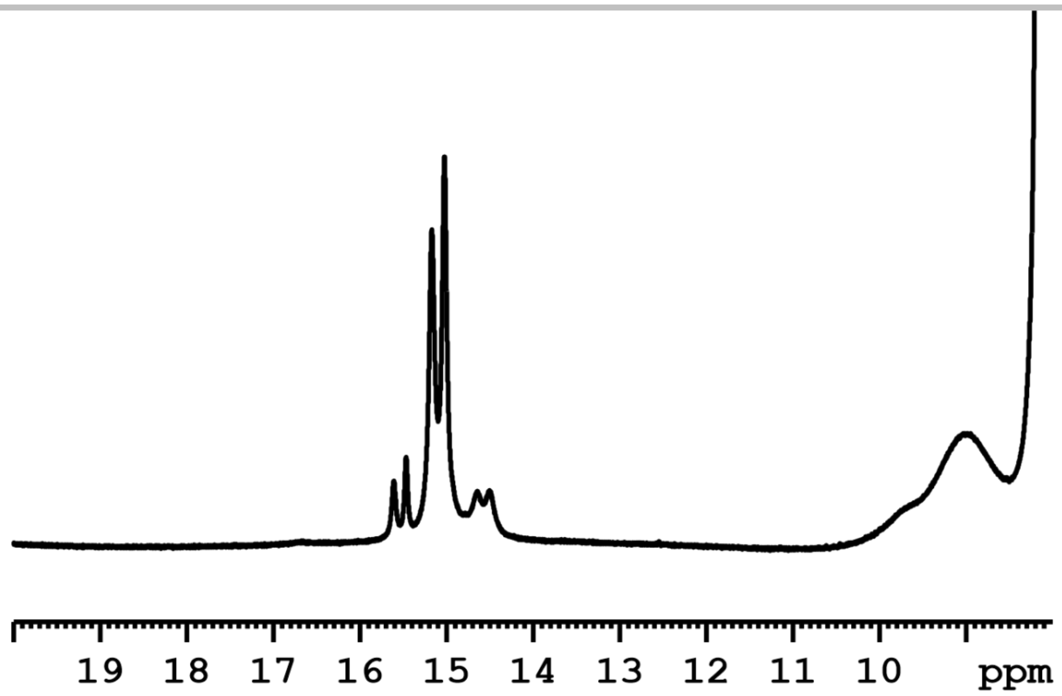

Hydrogen bond area of the ^{1}H-spectrum of the ternary complex of **1f/2a/HE 3b** (1:1:1 stoichiometry, 600MHz, CD_2Cl_2, 180K).

6.2. **NMR spectra of TRIFP 1a/2a/HE 3b**

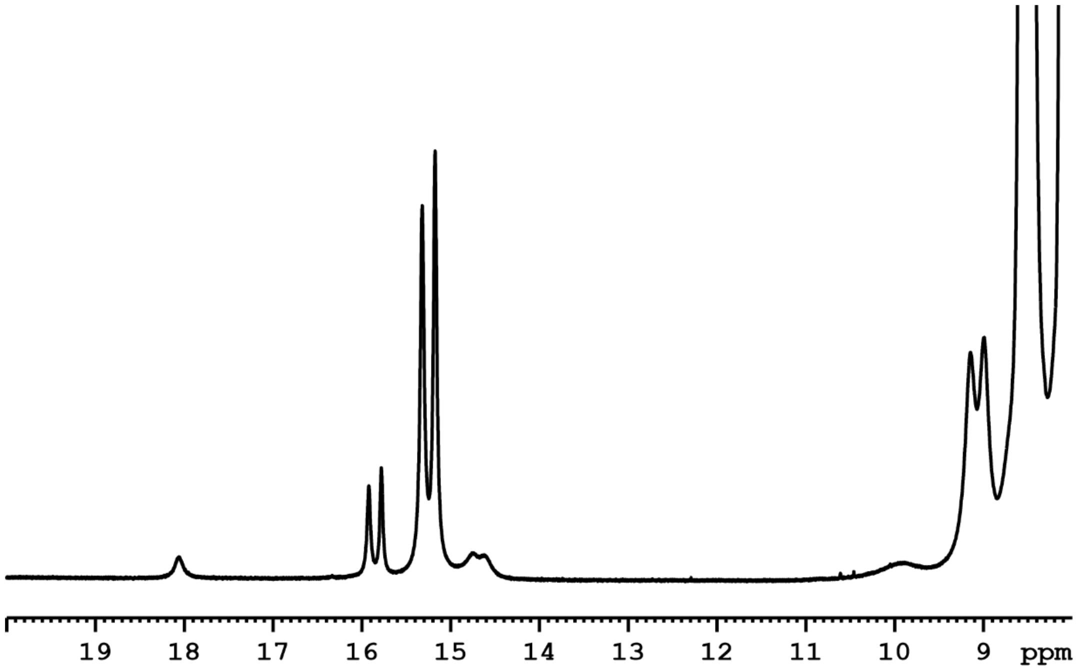

Hydrogen bond area of the ^{1}H-spectrum of the ternary complex of TRIFP **1a/2a/HE 3b** (1:1:1 stoichiometry, 600MHz, CD_2Cl_2, 180K).

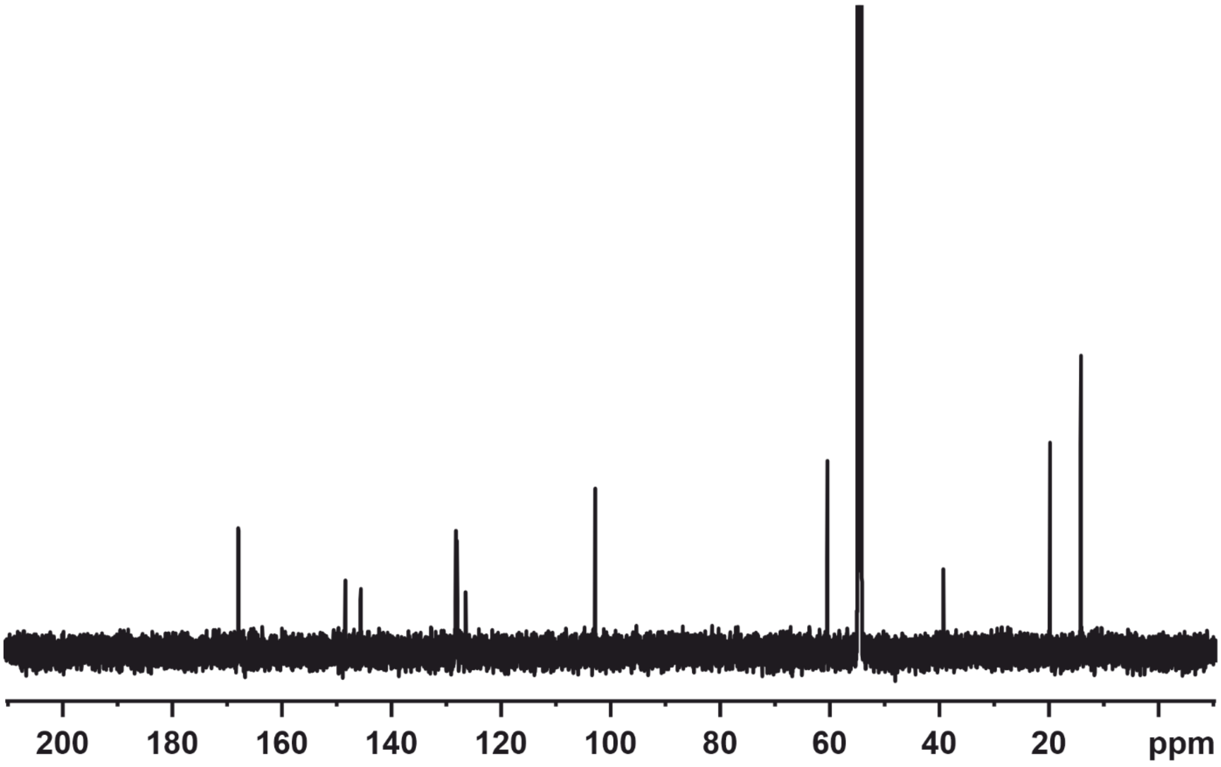

^{13}C-spectrum of the ternary complex of TRIFP **1a/2a/HE 3b** (1:1:1 stoichiometry, 600MHz, CD_2Cl_2, 180K).

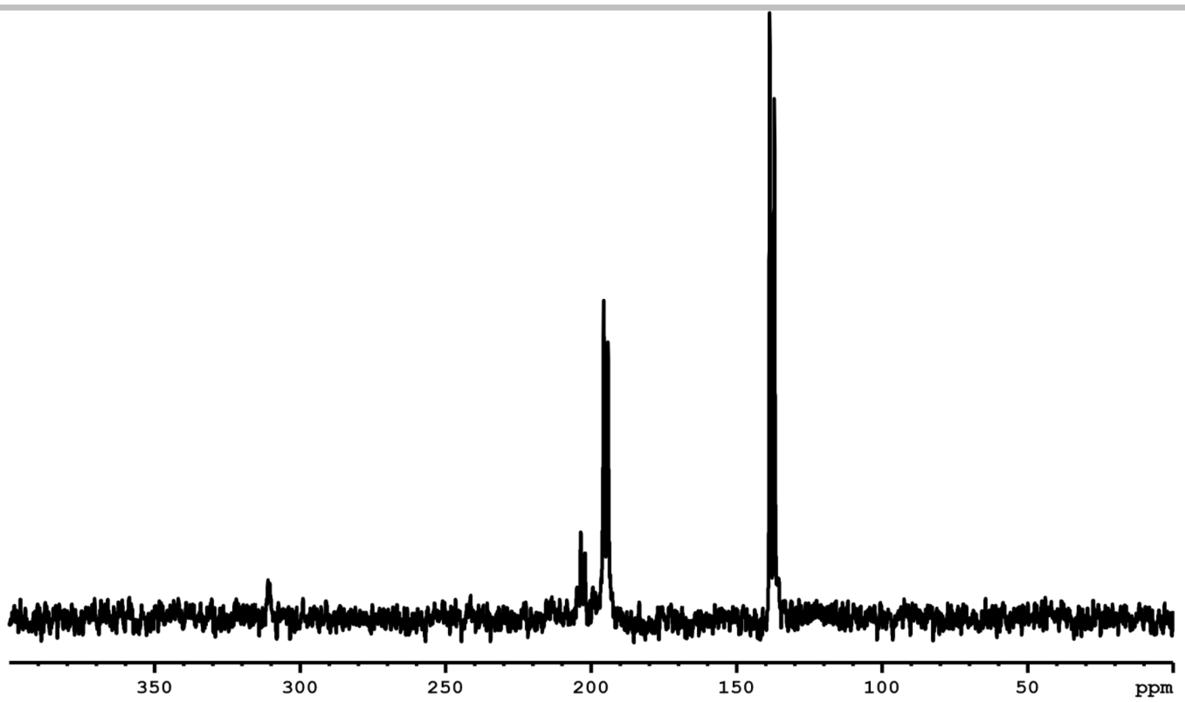

^{15}N-spectrum of the ternary complex of TRIFP **1a/2a/HE 3b** (1:1:1 stoichiometry, 600MHz, CD_2Cl_2, 180K).

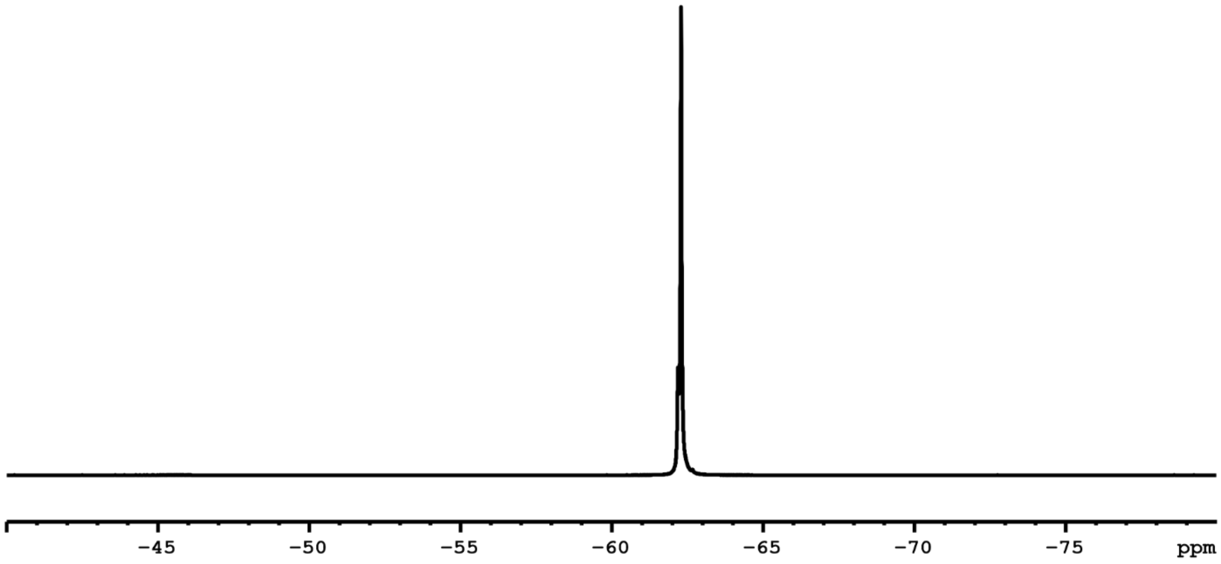

^{19}F-spectrum of the ternary complex of TRIFP **1a/2a/HE 3b** (1:1:1 stoichiometry, 600MHz, CD_2Cl_2, 180K).

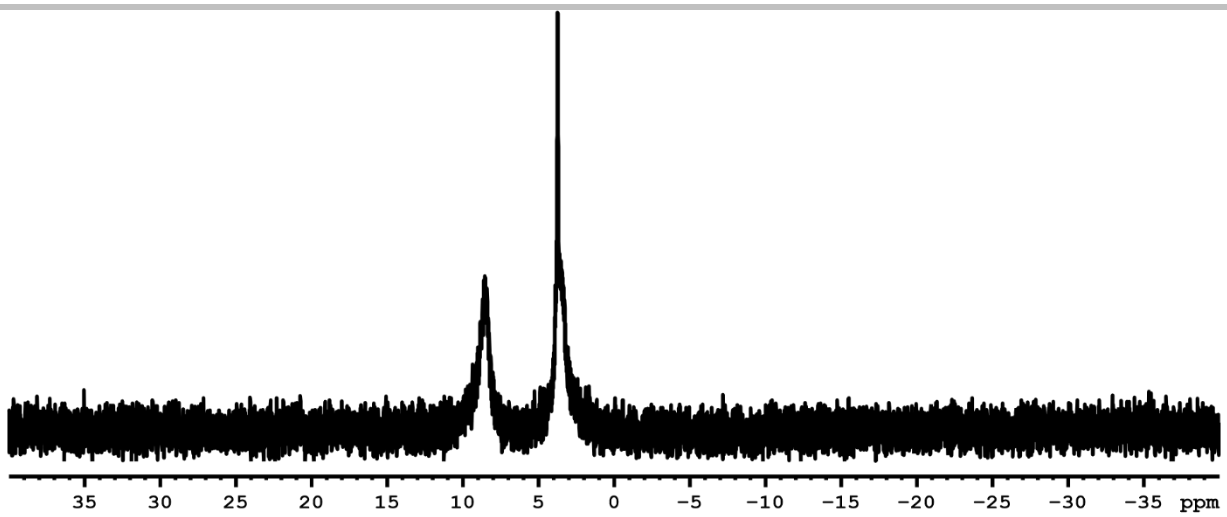

^{31}P-spectrum of the ternary complex of TRIFP **1a/2a/HE 3b** (1:1:1 stoichiometry, 600MHz, CD_2Cl_2, 180K).

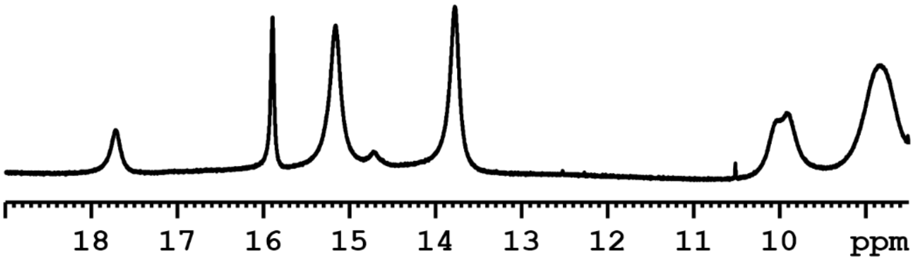

Hydrogen bond area of the ^{1}H-spectrum of the ternary complex of TRIFP **1a/2a/HE 3b** (1:1:1 stoichiometry, 600MHz, CD_2Cl_2, 180K). Neither imine **2a** nor HE **3b** are ^{15}N-labeled. No doublets are observed anymore, confirming all species bond to either **2a** or HE **3b**.

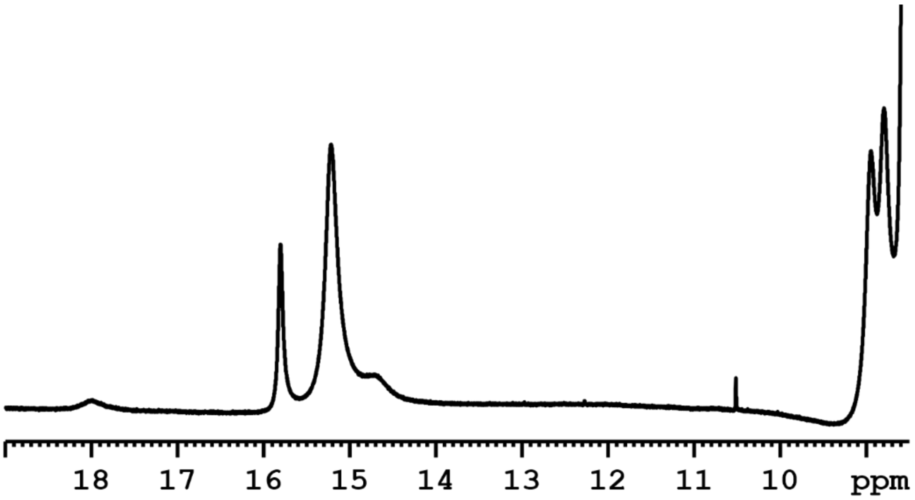

Hydrogen bond area of the ^{1}H-spectrum of the ternary complex of TRIFP **1a/2a/HE 3b** (1:1:1 stoichiometry, 600MHz, CD_2Cl_2, 180K). Imine **2a** is not ^{15}N-labeled while HE **3b** is ^{15}N-labeled. No doublets are observed anymore for all imine **2a** hydrogen-bonded species, while hydrogen-bonded HE **3b** species still appear as a doublet.

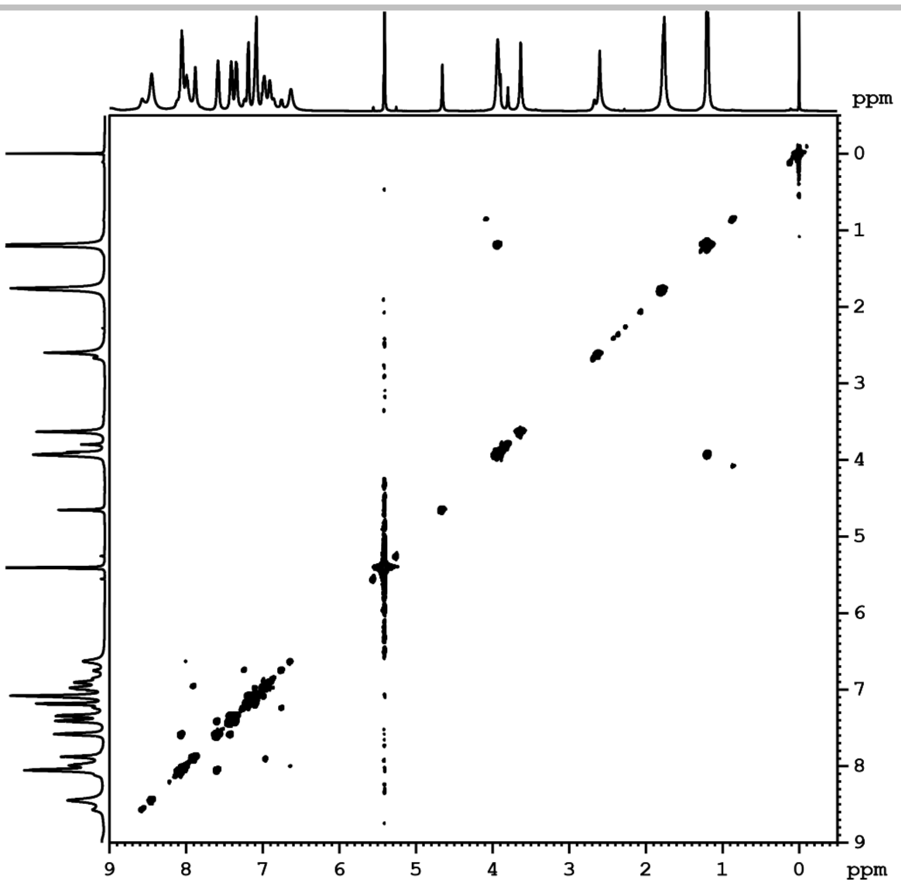

^{1}H,^{1}H-COSY of the ternary complex of TRIFP **1a/2a/HE 3b** (1:1:1 stoichiometry, 600MHz, CD_2Cl_2, 180K).

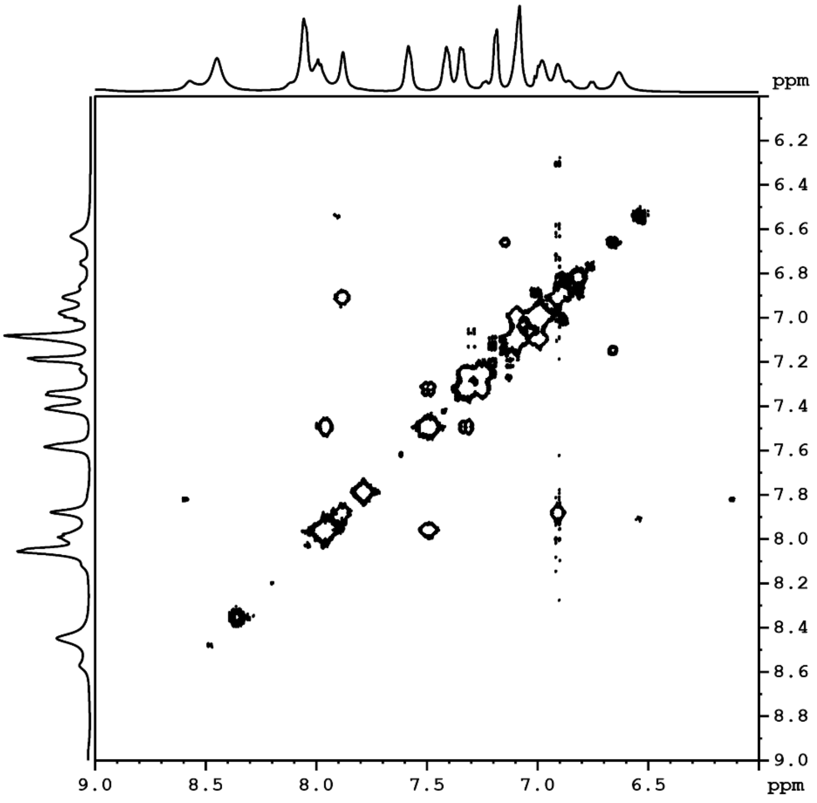

Aromatic region of ^{1}H,^{1}H-COSY of the ternary complex of TRIFP **1a/2a/HE 3b** (1:1:1 stoichiometry, 600MHz, CD_2Cl_2, 180K).

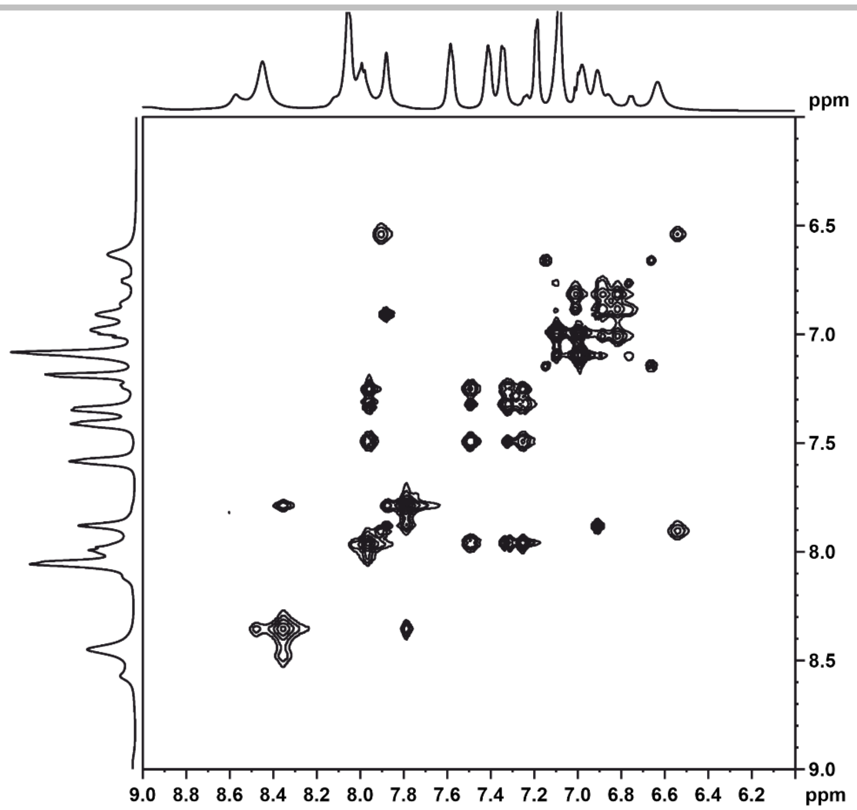

Aromatic region of $^{1}H,^{1}H$-TOCSY of the ternary complex of TRIFP **1a/2a/HE 3b** (1:1:1 stoichiometry, 600MHz, CD_2Cl_2, 180K).

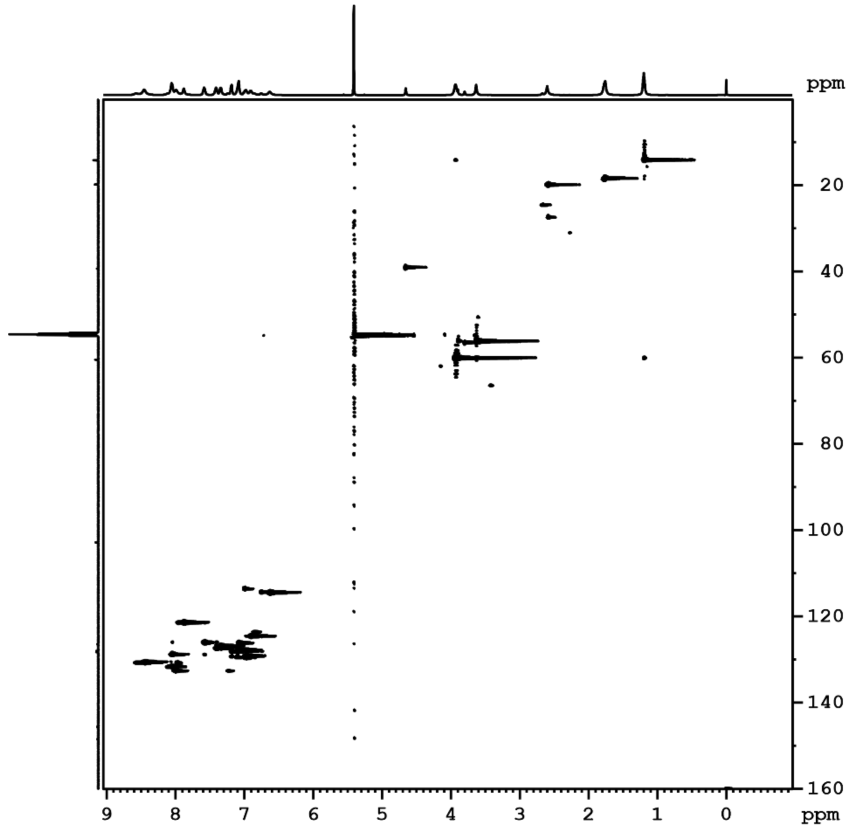

$^{1}H,^{13}C$-HSQC of the ternary complex of TRIFP **1a/2a/HE 3b** (1:1:1 stoichiometry, 600MHz, CD_2Cl_2, 180K).

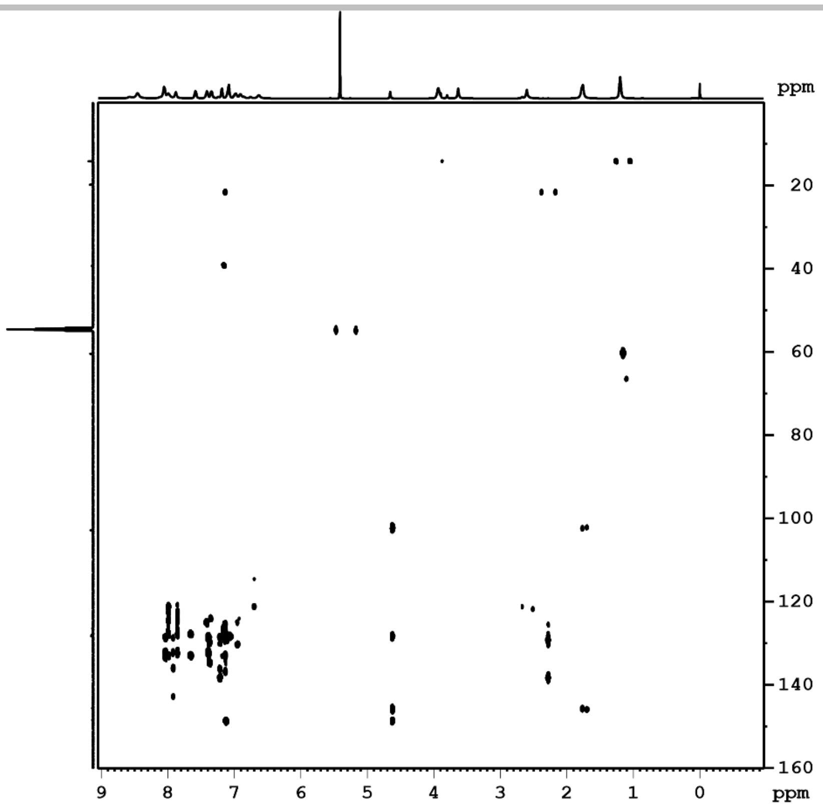

$^{1}H,^{13}C$-HMBC of the ternary complex of TRIFP **1a/2a/HE 3b** (1:1:1 stoichiometry, 600MHz, CD_2Cl_2, 180K).

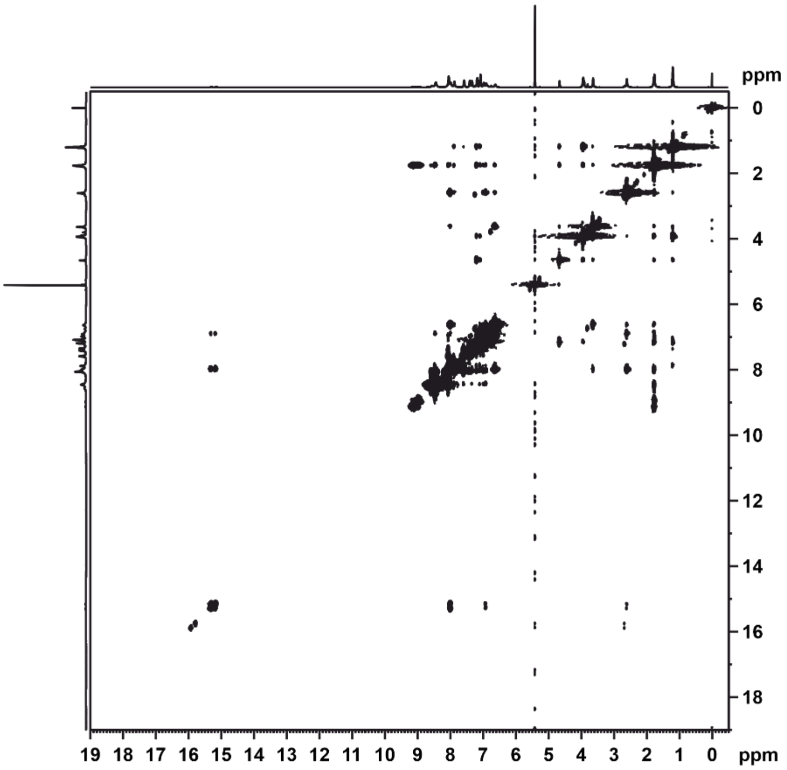

$^{1}H,^{1}H$-NOESY of the ternary complex of TRIFP **1a/2a/HE 3b** (1:1:1 stoichiometry, 600MHz, CD_2Cl_2, 180K).

6.3. NMR spectra of TRIFP 1a/*E*-2a/HE 3b

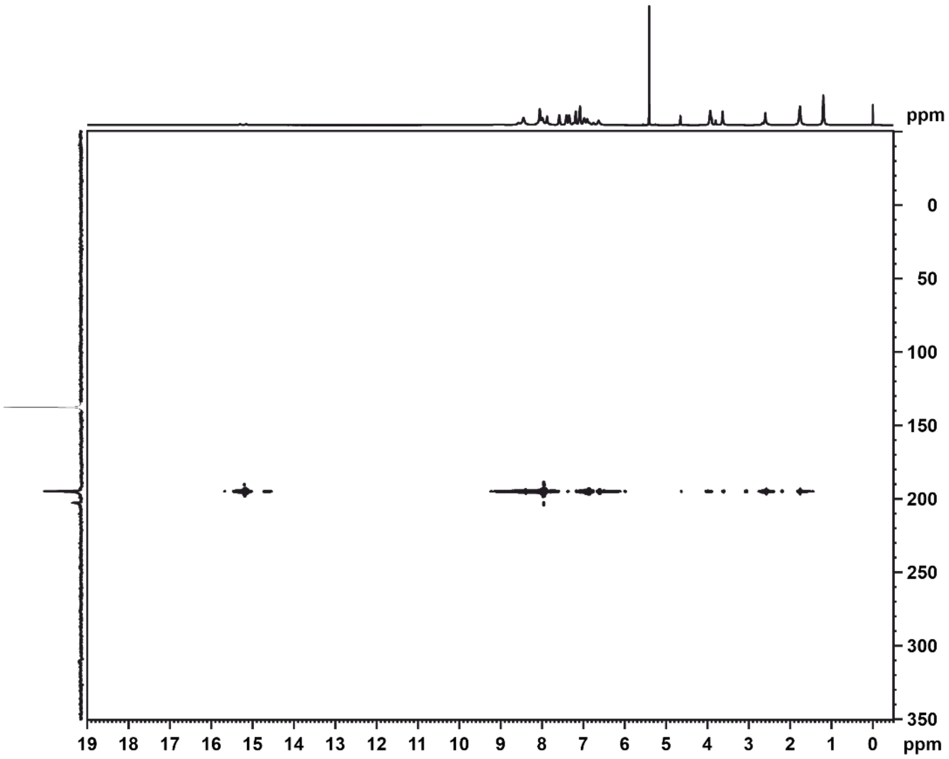

^{1}H,^{15}N-HSQC-NOESY of the ternary complex of TRIFP **1a/*E*-2a/HE 3b** with a mixing time of 0.03 ms (1:1:1 stoichiometry, 600MHz, CD_2Cl_2, 180K).

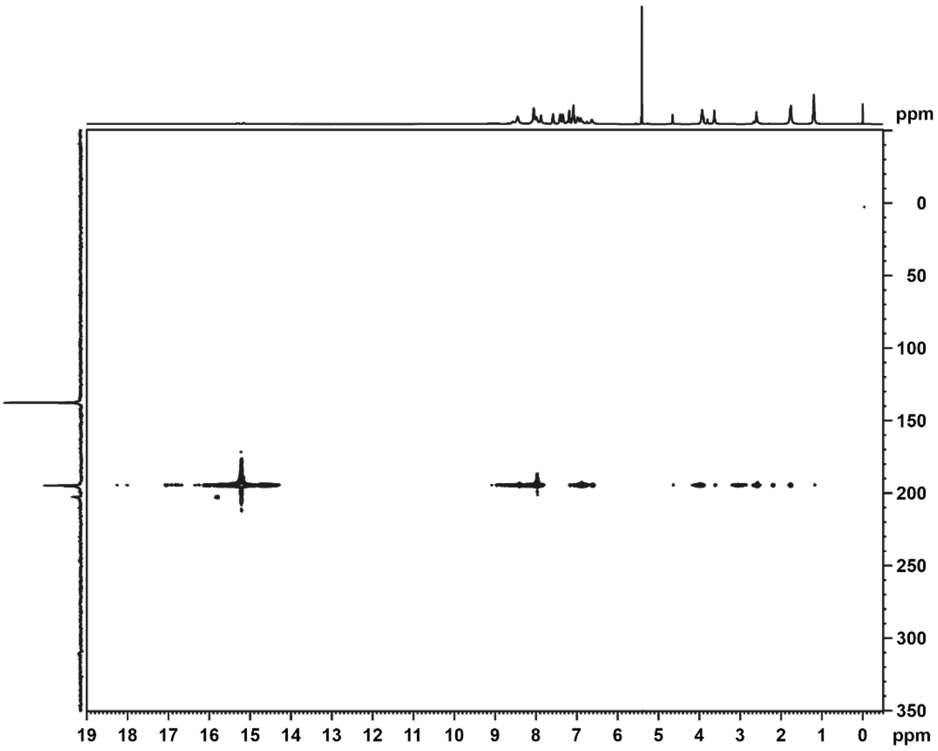

^{1}H,^{15}N-HSQC-NOESY of the ternary complex of TRIFP **1a/*E*-2a/HE 3b** with a mixing time of 0.03 ms (1:1:1 stoichiometry, 600MHz, CD_2Cl_2, 180K).

6.4. NMR spectra of OMe-CPA 1b/2a/HE 3b

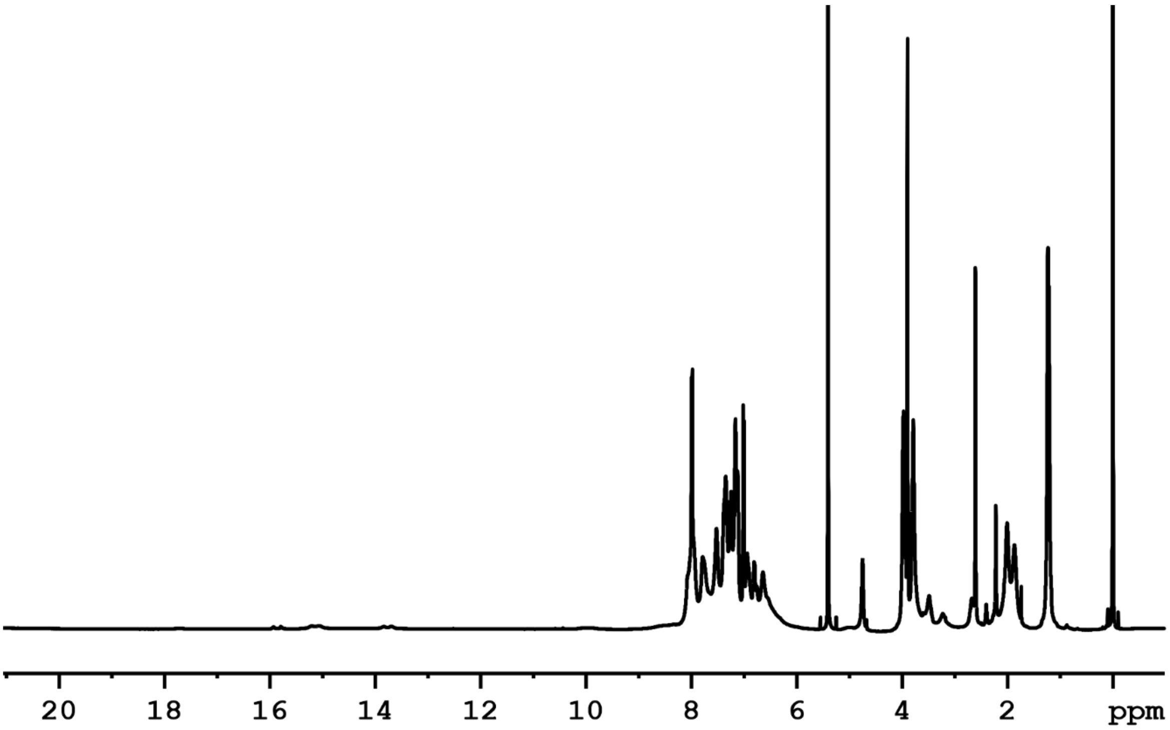

^{1}H-spectrum of the ternary complex of OMe-CPA **1b/2a/HE 3b** (1:1:1 stoichiometry, 600MHz, CD_2Cl_2, 180K).

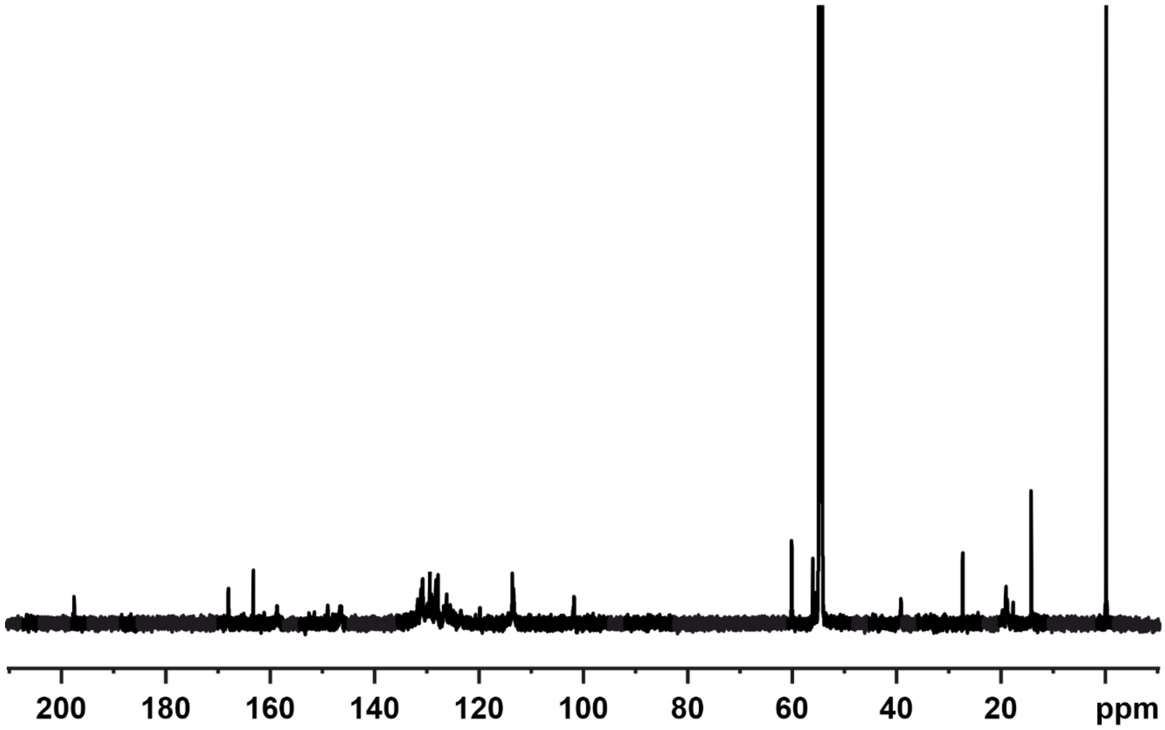

^{13}C-spectrum of the ternary complex of OMe-CPA **1b/2a/HE 3b** (1:1:1 stoichiometry, 600MHz, CD_2Cl_2, 180K).

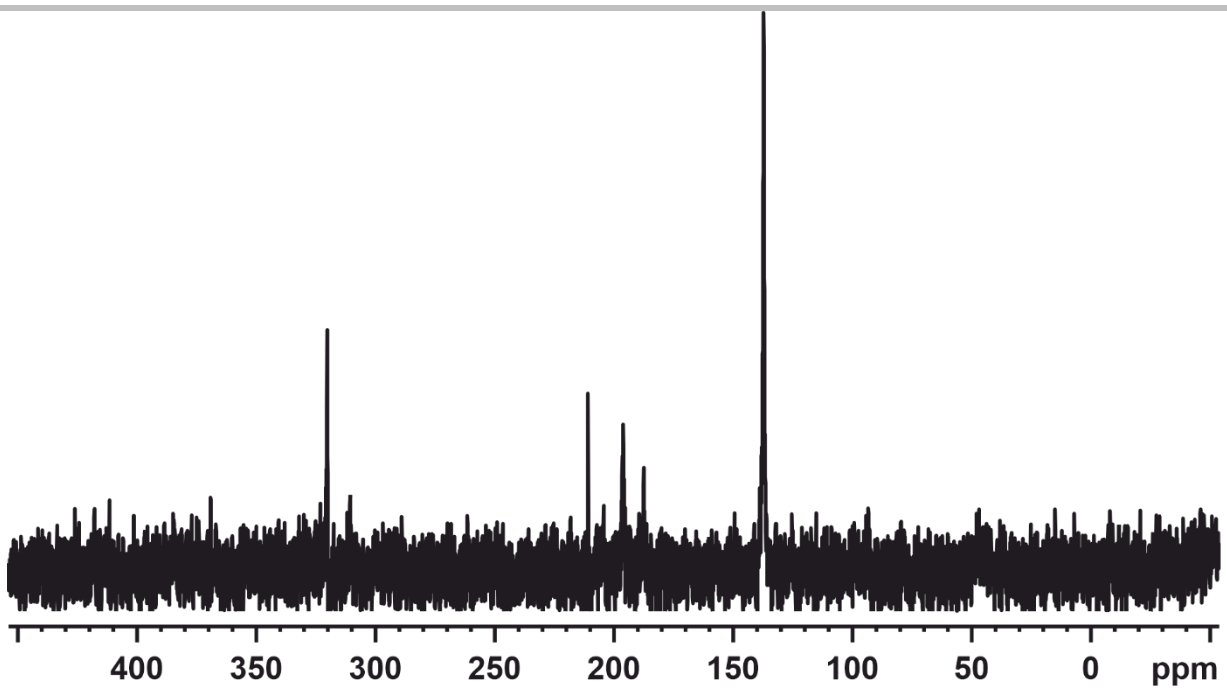

^{15}N-spectrum of the ternary complex of OMe-CPA **1b/2a/HE 3b** (1:1:1 stoichiometry, 600MHz, CD_2Cl_2, 180K).

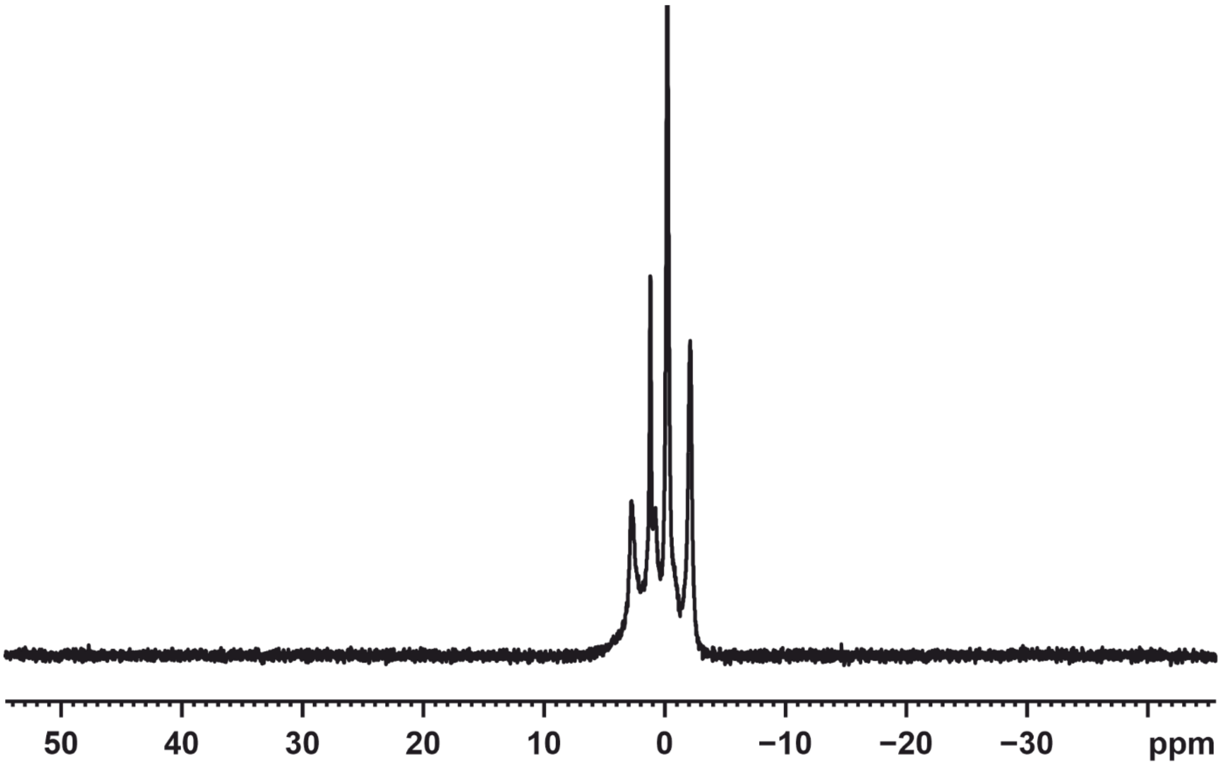

^{31}P-spectrum of the ternary complex of OMe-CPA **1b/2a/HE 3b** (1:1:1 stoichiometry, 600MHz, CD_2Cl_2, 180K).

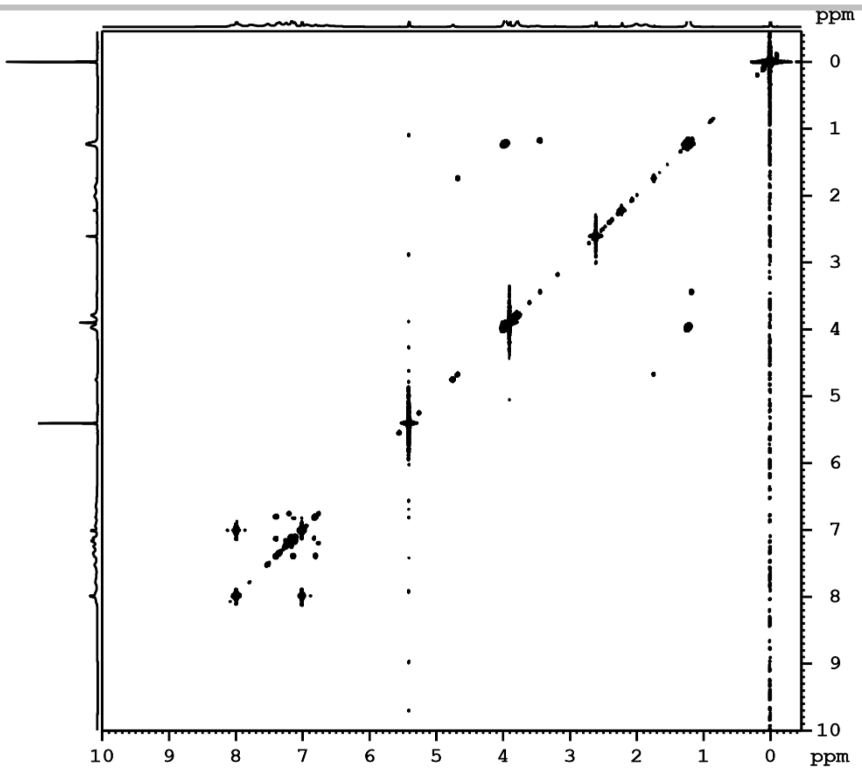

^{1}H,^{1}H-COSY-spectrum of the ternary complex of OMe-CPA **1b/2a/HE 3b** (1:1:1 stoichiometry, 600MHz, CD_2Cl_2, 180K).

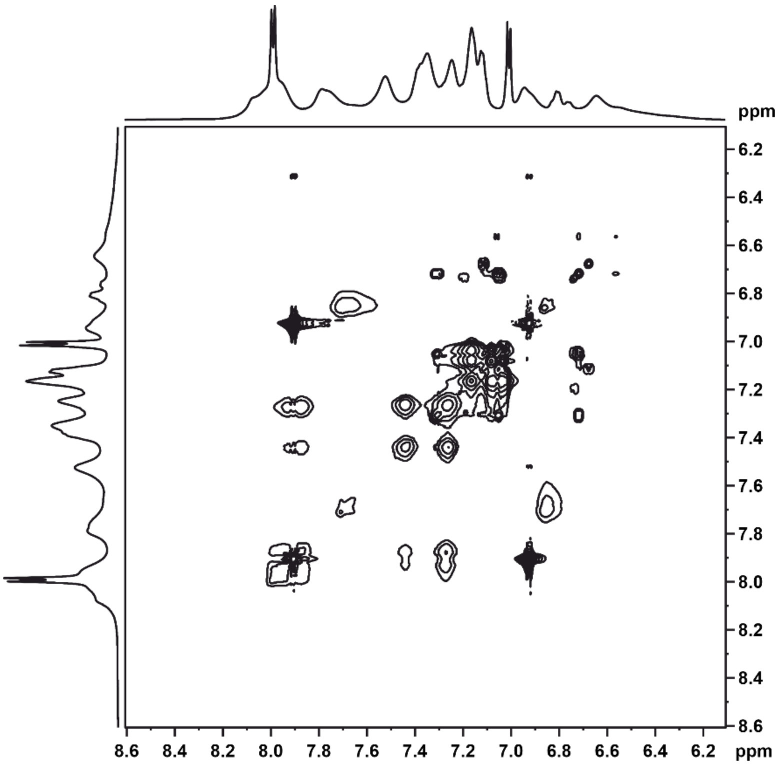

^{1}H,^{1}H-TOCSY-spectrum of the ternary complex of OMe-CPA **1b/2a/HE 3b** (1:1:1 stoichiometry, 600MHz, CD_2Cl_2, 180K).

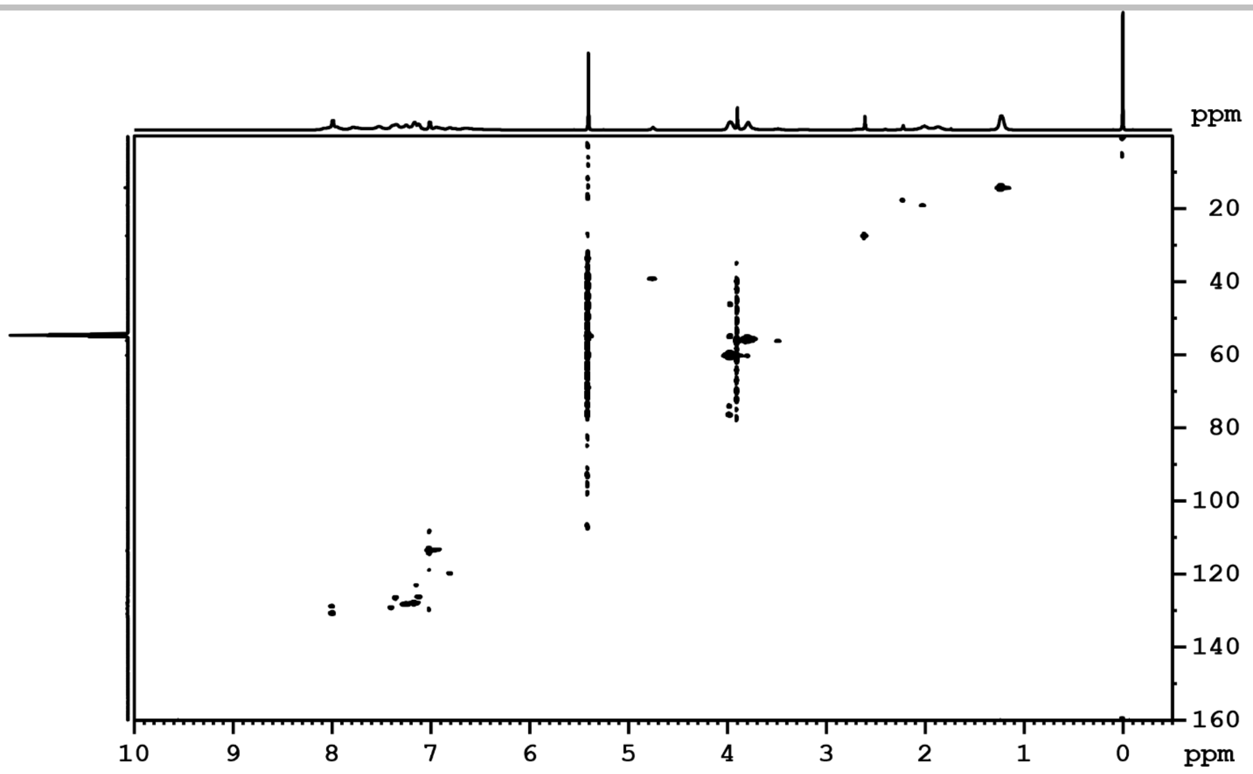

$^{1}H,^{13}C$-HSQC-spectrum of the ternary complex of OMe-CPA **1b/2a/HE 3b** (1:1:1 stoichiometry, 600MHz, CD_2Cl_2, 180K).

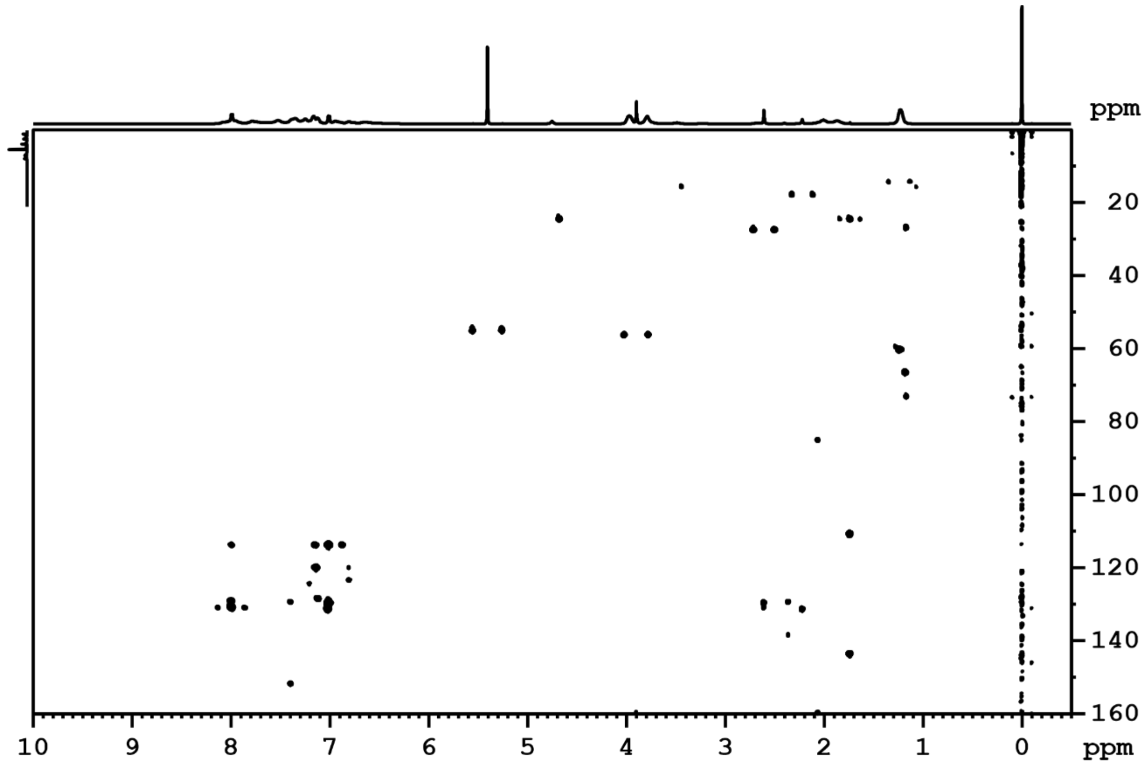

$^{1}H,^{13}C$-HMBC-spectrum of the ternary complex of OMe-CPA **1b/2a/HE 3b** (1:1:1 stoichiometry, 600MHz, CD_2Cl_2, 180K).

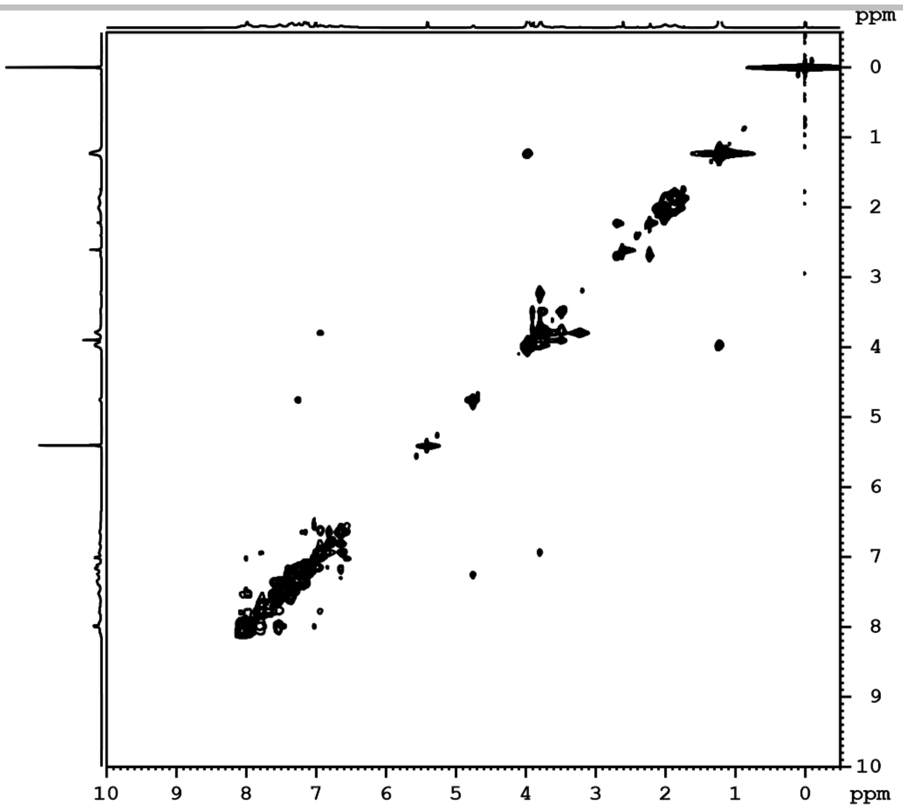

^{1}H,^{1}H-NOESY-spectrum of the ternary complex of OMe-CPA **1b/2a/HE 3b** (1:1:1 stoichiometry, 600MHz, CD_2Cl_2, 180K).

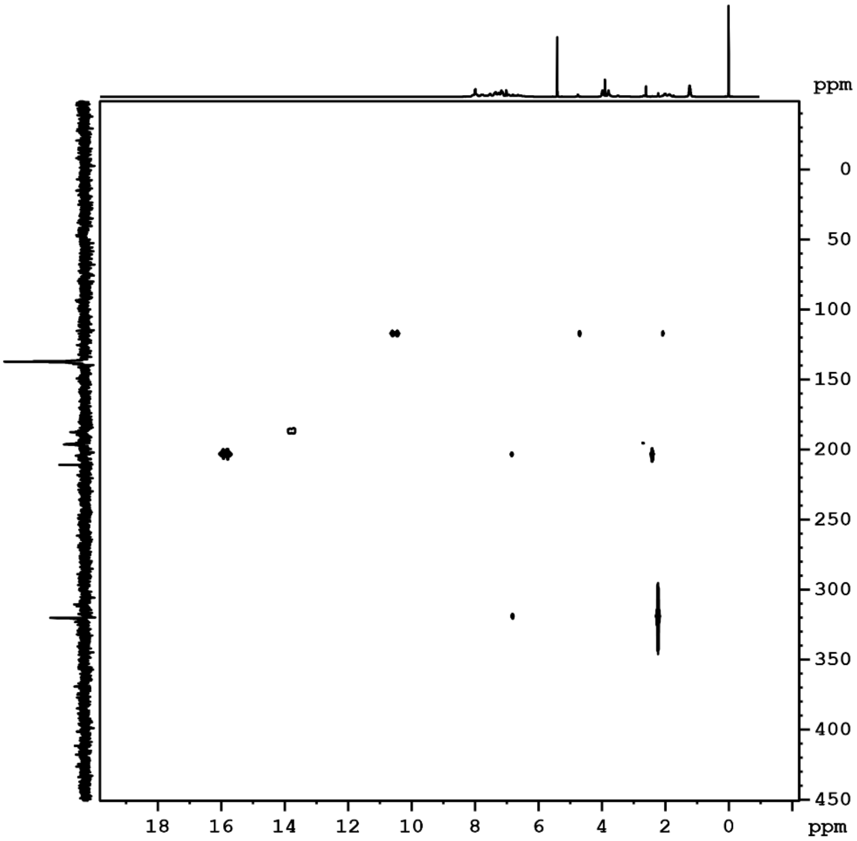

^{1}H,^{15}N-HMBC-spectrum of the ternary complex of OMe-CPA **1b/2a/HE 3b** (1:1:1 stoichiometry, 600MHz, CD_2Cl_2, 180K).

6.5. Pulse program ^{15}N hsqcetgpnosp

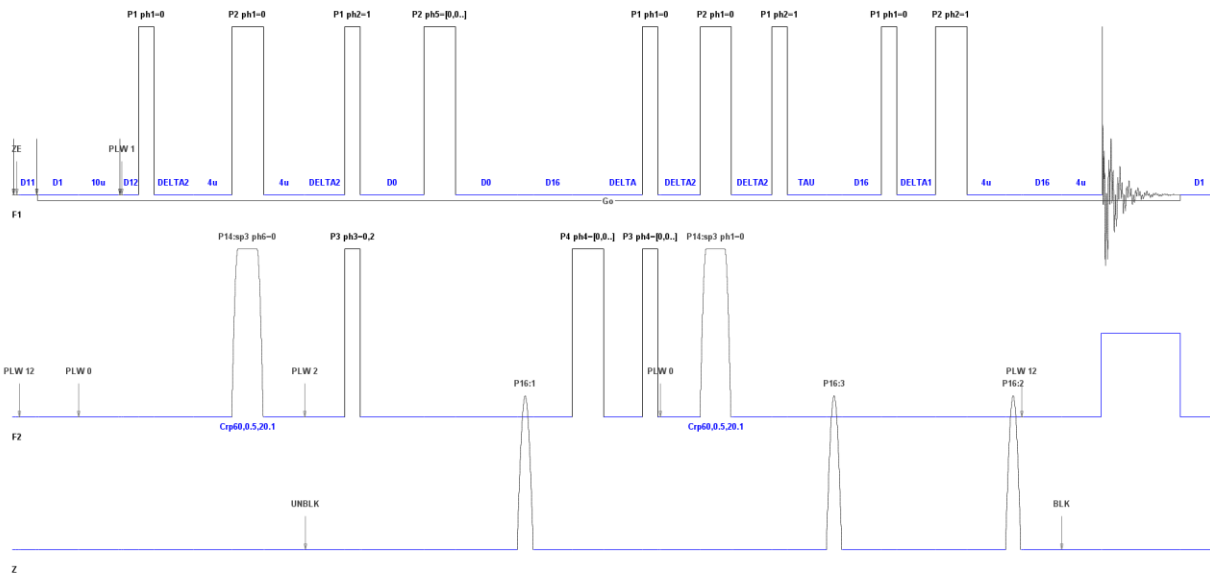


```
;hsqcetgpnosp
;avance-version (20/03/03)
;HSQC-NOESY
;2D H-1/X correlation via double inept transfer
;with correlation via dipolar coupling
;dipolar coupling may be due to noe or chemical exchange
;phase sensitive using Echo/Antiecho-TPPI gradient selection
;with decoupling during acquisition - using f2 (and f3)
;using trim pulses in inept transfer
;using shaped pulses for inversion on f2 - channel
;
;$CLASS=HighRes
;$DIM=2D
;$TYPE=
;$SUBTYPE=
;$COMMENT=

#include <Avance.incl>
#include <Grad.incl>
#include <Delay.incl>

"p2=p1*2"
"p4=p3*2"
"d4=1s/(cnst2*4)"
"d11=30m"
"d12=20u"

#   ifdef LABEL_CN
"p22=p21*2"
#   else
#   endif /*LABEL_CN*/

"d0=3u"

"in0=inf1/2"

"DELTA1=p16+d16+8u"
```

```
"DELTA2=d4-larger(p2,p14)/2"

#   ifdef LABEL_CN
"DELTA=p16+d16+larger(p2,p22)+d0*2"
#   else
"DELTA=p16+d16+p2+d0*2"
#   endif /*LABEL_CN*/

"TAU=d8-p16-d16"

1 ze

#   ifdef LABEL_CN
  d11 pl12:f2 pl16:f3
2 d1 do:f2 do:f3
  10u pl0:f2 pl3:f3
#   else
  d11 pl12:f2
2 d1 do:f2
  10u pl0:f2
#   endif /*LABEL_CN*/

3 d12 pl1:f1
  (p1 ph1)
  DELTA2
  4u
  (center (p2 ph1) (p14:sp3 ph6):f2 )
  4u
  DELTA2 pl2:f2 UNBLKGRAD

#   ifdef TRIMP
  p28 ph1
#   endif /* TRIMP */

  (p1 ph2) (p3 ph3):f2
  d0

#   ifdef LABEL_CN
  (center (p2 ph5) (p22 ph1):f3 )
#   else
  (p2 ph5)
#   endif /*LABEL_CN*/

  d0
  p16:gp1*EA
  d16
  (p4 ph4):f2
  DELTA
  (ralign (p1 ph1) (p3 ph4):f2 )
  DELTA2 pl0:f2
  (center (p2 ph1) (p14:sp3 ph1):f2 )
  DELTA2
  (p1 ph2)
  TAU
  p16:gp3
  d16
  (p1 ph1)
  DELTA1
  (p2 ph2)
  4u
  p16:gp2
```

```
#   ifdef LABEL_CN
  d16 pl12:f2 pl16:f3
  4u BLKGRAD
  go=2 ph31 cpd2:f2 cpd3:f3
  d1 do:f2 do:f3 mc #0 to 2
#   else
  d16 pl12:f2
  4u BLKGRAD
  go=2 ph31 cpd2:f2
  d1 do:f2 mc #0 to 2
#   endif /*LABEL_CN*/

    F1EA(calgrad(EA), caldel(d0, +in0) & calph(ph3, +180) & calph(ph6, +180) & calph(ph31, +180))
exit

ph1=0
ph2=1
ph3=0 2
ph4=0 0 0 0 2 2 2 2
ph5=0 0 2 2
ph6=0
ph22=3
ph23=0
ph24=1
ph25=2
ph26=0
ph31=0 2 0 2 2 0 2 0

;pl0 : 0W
;pl1 : f1 channel - power level for pulse (default)
;pl2 : f2 channel - power level for pulse (default)
;pl3 : f3 channel - power level for pulse (default)
;pl12: f2 channel - power level for CPD/BB decoupling
;pl16: f3 channel - power level for CPD/BB decoupling
;sp3: f2 channel - shaped pulse 180 degree
;p1 : f1 channel -  90 degree high power pulse
;p2 : f1 channel - 180 degree high power pulse
;p3 : f2 channel -  90 degree high power pulse
;p4 : f2 channel - 180 degree high power pulse
;p14: f2 channel - 180 degree shaped pulse for inversion
;p16: homospoil/gradient pulse
;p22: f3 channel - 180 degree high power pulse
;p28: f1 channel - trim pulse in inept transfer      [1 msec]
;d0 : incremented delay (2D)                         [3 usec]
;d1 : relaxation delay; 1-5 * T1
;d4 : 1/(4J)XH
;d8 : mixing time
;d11: delay for disk I/O                             [30 msec]
;d12: delay for power switching                      [20 usec]
;d16: delay for homospoil/gradient recovery
;cnst2: = J(XH)
;inf1: 1/SW(X) = 2 * DW(X)
;in0: 1/(2 * SW(X)) = DW(X)
;nd0: 2
;ns: 1 * n
;ds: >= 16
;td1: number of experiments
;FnMODE: echo-antiecho
;cpd2: decoupling according to sequence defined by cpdprg2
;pcpd2: f2 channel - 90 degree pulse for decoupling sequence
;cpd3: decoupling according to sequence defined by cpdprg3
;pcpd3: f3 channel - 90 degree pulse for decoupling sequence
```

;for z-only gradients:
;gpz1: 80%
;gpz2: 20.1% for C-13, 8.1% for N-15
;gpz3: 50%

;use gradient files:
;gpnam1: SMSQ10.100
;gpnam2: SMSQ10.100
;gpnam3: SMSQ10.100

;preprocessor-flags-start
;LABEL_CN: for C-13 and N-15 labeled samples start experiment with
; option -DLABEL_CN (eda: ZGOPTNS)
;TRIMP: to use trimpulse p28@pl1 start experiment with
; option -DTRIMP (eda: ZGOPTNS)
;preprocessor-flags-end

;$Id:$

6.6. **Pulse program ^{1}H noesygpph**

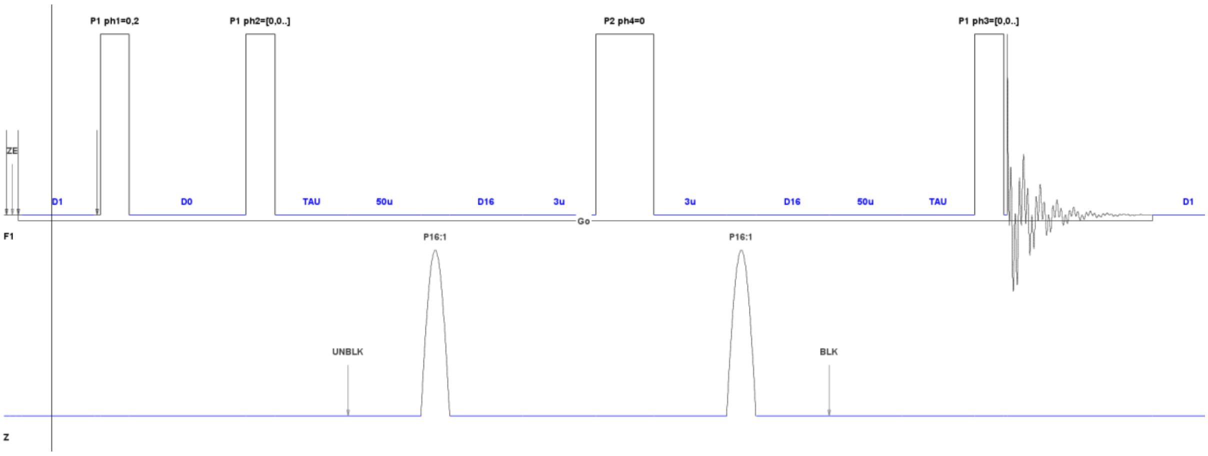


```
;noesygpph
;avance-version (12/01/11)
;2D homonuclear correlation via dipolar coupling
;dipolar coupling may be due to noe or chemical exchange.
;phase sensitive
;with gradient pulses in mixing time
;
;J. Jeener, B.H. Meier, P. Bachmann & R.R. Ernst, J. Chem. Phys. 71,
;   4546-4553 (1979)
;R. Wagner & S. Berger, J. Magn. Reson. 123 A, 119-121 (1996)
;
;$CLASS=HighRes
;$DIM=2D
;$TYPE=
;$SUBTYPE=
;$COMMENT=

#include <Avance.incl>
#include <Grad.incl>
#include <Delay.incl>

"p2=p1*2"

"in0=inf1"

"d0=in0/2-p1*4/3.1416"

"TAU=d8*0.5-p16-d16-50u"

"acqt0=-p1*2/3.1416"

1 ze
2 d1
3 p1 ph1
  d0
  p1 ph2
  TAU
  50u UNBLKGRAD
  p16:gp1
  d16
  3u
```

```
  (p2 ph4):f1
  3u
  p16:gp1*-1
  d16
  50u BLKGRAD
  TAU
  p1 ph3
  go=2 ph31
  d1 mc #0 to 2 F1PH(calph(ph1, +90), caldel(d0, +in0))
exit

ph1=0 2
ph2=0 0 0 0 0 0 0 0 2 2 2 2 2 2 2 2
ph3=0 0 2 2 1 1 3 3
ph4=0
ph31=0 2 2 0 1 3 3 1 2 0 0 2 3 1 1 3

;pl1 : f1 channel - power level for pulse (default)
;p1 : f1 channel -  90 degree high power pulse
;p2 : f1 channel - 180 degree high power pulse
;p16: homospoil/gradient pulse                       [1 msec]
;d0 : incremented delay (2D)
;d1 : relaxation delay; 1-5 * T1
;d8 : mixing time
;d16: delay for homospoil/gradient recovery
;inf1: 1/SW = 2 * DW
;in0: 1/(1 * SW) = 2 * DW
;nd0: 1
;ns: 2 * n
;ds: 16
;td1: number of experiments
;FnMODE: States-TPPI, TPPI, States or QSEQ

;use gradient ratio:    gp 1
;                        40

;for z-only gradients:
;gpz1: 40%
;use gradient files:
;gpnam1: SMSQ10.100

;Processing

;PHC0(F1): 90
;PHC1(F1): -180
;FCOR(F1): 1

;$Id:$
```

7. Optimized Cartesian Coordinates of all the molecular complexes:

7.1. CPA 1a, *E*-imine 2a, HE 3b

7.1.1. Conformer C1

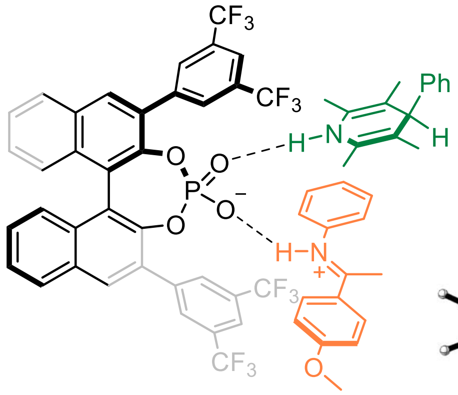

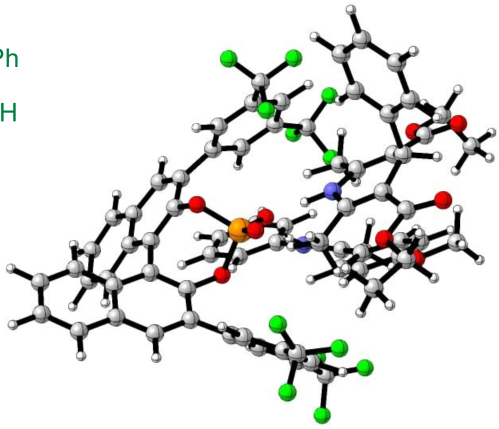

G_{180K}:-5019.33126263975

P	-0.88664144587749	-0.64023375189892	0.55454257982724
O	0.42134410691612	-0.97754167294515	1.22058542355951
O	-1.08520381957317	0.61873563383717	-0.26230304332406
O	-2.07210392385353	-0.73832174136763	1.73882344395265
C	-3.37644078870463	-0.71355446821343	1.28026507770177
C	-3.93796894558094	-1.90802609900243	0.81499055143409
C	-5.27934035774635	-1.87806209172497	0.28920394382915
C	-5.89856593245866	-3.01250185961708	-0.31964296799240
C	-7.18716371593193	-2.93459114990189	-0.83445585075106
C	-7.92406770769515	-1.71809720954579	-0.76636675550821
C	-7.34352633356730	-0.58958905449020	-0.20453116188903
C	-6.01313941574871	-0.63095069582048	0.31483309787732
C	-5.37235943500181	0.55727325822793	0.76856757612818
C	-4.05870370695838	0.54704450318247	1.22239781968492
C	-3.29548983943395	1.79958085117970	1.47213517859326
C	-2.46548891965609	1.95237337236064	2.60015183754591
C	-1.60723025076826	3.05908789883388	2.70736110593449
C	-1.57588972161262	4.04069664788613	1.70767438290390
C	-2.41097081437658	3.89743482591400	0.58723128230102
C	-3.26890490944949	2.79252049224845	0.46994568313559
H	-3.86796149533002	2.65816532023394	-0.43666996685254
C	-2.24423673019004	4.84543220769535	-0.57970353564539
F	-1.30663584791705	4.36605216556363	-1.45658436387365
F	-3.38636991670156	5.01048312144836	-1.28674984987067
F	-1.81951038816462	6.07359152267397	-0.20060965564389
H	-0.88616137771185	4.88563566606720	1.78001987413025
C	-0.62493295659257	3.08846146132931	3.85848699673177
F	-1.23570462835998	2.93436851841903	5.05842355006637
F	0.09751755901499	4.23224900393094	3.91383466131474
F	0.27096040356551	2.06411524836177	3.74617066087183
H	-2.45677027901151	1.18138489717869	3.37567742279358
H	-5.91964652594490	1.50625255319849	0.71233482542097
H	-7.88606906986634	0.36397185817565	-0.17191977499889
H	-8.94248514459930	-1.67245771665176	-1.17245835501672
H	-7.63893804585412	-3.81867401897484	-1.30214028557962
H	-5.34126861604121	-3.95232160709749	-0.38510588775308
C	-3.07783259117819	-3.13163041120163	0.78693040433856
C	-1.83587181003977	-3.06501097942024	0.13897131719764

C	-0.92097816392758	-4.16710020867163	0.08834973674164
C	-1.28836628371514	-5.35504973233561	0.71210370828805
C	-2.53014776460301	-5.48194120094673	1.39979493718351
C	-2.88298687459123	-6.70015488868690	2.05935619809382
C	-4.06806784518765	-6.80529090894814	2.77442599388827
C	-4.94298323252749	-5.68567147448169	2.86613634711089
C	-4.63437130773458	-4.49112571863149	2.22691226791390
C	-3.43672558836797	-4.35483394956845	1.45904355300499
H	-5.31163163278780	-3.63537092293421	2.31160679999487
H	-5.86812425396238	-5.76493378692317	3.45124524068865
H	-4.32704320824975	-7.74427550776789	3.27989326022258
H	-2.18863443649626	-7.54799917312710	1.99546750060098
H	-0.60944008794905	-6.21651224407731	0.68194118916993
C	0.39814978390430	-4.00717881578667	-0.58207091764632
C	0.48708190787685	-3.45276825693152	-1.87761337275730
C	1.73518925915974	-3.25592659220824	-2.48547156243579
C	2.92005043601813	-3.61004380465315	-1.81803738911011
C	2.83511851908404	-4.17325391054904	-0.53534684239410
C	1.58645521910949	-4.37654969858957	0.07917947890430
H	1.53624229417447	-4.79229350902943	1.09116375731556
C	4.10127288859787	-4.61153796716137	0.17150497892285
F	4.40798144295619	-5.90221279626520	-0.11761819258687
F	3.99814538241741	-4.52460725027158	1.51991460553610
F	5.17552227401740	-3.86205192011753	-0.19606166860326
H	3.89373436132222	-3.43897118293518	-2.28683445323837
C	1.80877074215632	-2.72324711086849	-3.90229718502734
F	2.92651318048407	-1.98828357005192	-4.11833968828472
F	1.82717975133300	-3.74011453108979	-4.80450026966081
F	0.74561720694092	-1.94098070744992	-4.21728962641720
H	-0.42600790906609	-3.16778248265123	-2.40672649547903
O	-1.44458218412769	-1.87878853142823	-0.44906568790734
C	-0.29760677463132	1.97592847756342	-3.36198661140275
N	-1.32090413727912	1.44517185129043	-2.71722330463067
C	-2.72032792121376	1.48768305266098	-2.95036205418470
C	-3.44188994340483	0.34839623036477	-2.51989876394165
C	-4.83304558930074	0.30611211612095	-2.66903742648951
C	-5.51881391695698	1.40539721222325	-3.21896408972825
C	-4.80170859547192	2.54706303696459	-3.61834566668898
C	-3.40376790779211	2.59786417123893	-3.48937700513111
H	-2.86868056626970	3.50943465742558	-3.75902155173153
H	-5.33198805123471	3.41740880964386	-4.02435597890260
H	-6.61070350779239	1.37588466551392	-3.32190559518774
H	-5.38419458553020	-0.58131050243315	-2.34031489478566
H	-2.90160769979070	-0.48769940069232	-2.06256802156609
H	-1.11366224009121	0.96227899142637	-1.78935773371324
C	1.04560729020267	1.66130593492065	-2.88095003913537
C	1.32371038189124	0.44723679909776	-2.18871383216306
C	2.58721701360747	0.17994599459638	-1.68521939187604
C	3.63122028837001	1.12865135811628	-1.83956699028223
C	3.38039636852131	2.33657524037032	-2.53834167199142
C	2.11060788845848	2.58072683097518	-3.06595194734384
H	1.93047927379912	3.52607149017820	-3.58966546465801
H	4.16625366360177	3.08598507413793	-2.66279570412800
H	2.79334133408506	-0.76057702029914	-1.16552133509026
H	0.54238791355797	-0.30783100759687	-2.08348992551829
C	-0.49241716037032	2.86768673839854	-4.55106446898789
H	0.41209718588184	2.88480219545201	-5.17857756044307
H	-0.70545062612363	3.89997864623448	-4.21136298708306
H	-1.35771945455845	2.53160397919482	-5.14979145056191
C	5.89713438250867	1.73674568655031	-1.33699951595145
H	5.59301446745255	2.68812670047067	-0.86339814591424
H	6.71513917637815	1.28443458765715	-0.75578518123963
H	6.21884306432095	1.92444492734048	-2.38026435608730
O	4.81753947595813	0.79183204344921	-1.30709385757354
H	1.78841924930885	0.35403130660260	1.39211972443955
C	3.82406515935756	0.21513240071232	1.72399157540634
C	2.60203861912560	2.23679594872766	1.07842100969881

C	4.99392115274573	0.92875886670244	1.88606193780105
C	3.74927705282607	3.00091818316209	1.17673850377718
C	4.91390236715241	2.45454403643340	1.98895581518871
N	2.65142349214283	0.91436825636918	1.47985325353293
H	5.85333591868701	2.87008830470697	1.59264451466438
C	6.34819052680436	0.35752398876624	1.94382074685800
O	7.37914355090186	1.02888628699899	1.85937701304922
O	6.39340631857335	-0.99260410171521	2.09530528465441
C	3.92875038761787	4.35900673194803	0.64759316848500
O	4.99131957129013	4.97948471673029	0.71775807484340
O	2.82385477956051	4.88787524015730	0.04805895132777
C	2.92492657089589	6.20584549312718	-0.53953307963215
C	3.51715520338478	6.14842481238315	-1.94524813649177
H	3.53100410582769	6.85513025120198	0.11912764609220
H	1.88416522935926	6.57707644512034	-0.55933357121892
H	3.54488766318097	7.16688634654451	-2.37822750783871
H	4.54781120934347	5.75097152275611	-1.91316881439015
H	2.90319127234982	5.50418367166818	-2.60130733705382
C	7.69905030243307	-1.61489065244905	2.13751789628625
C	7.49872189438644	-3.09193387028101	2.44149327990648
H	8.31257955263725	-1.11258303994282	2.91017367408881
H	8.20077922936518	-1.45901969172781	1.16188697746063
H	8.48223949427268	-3.59790596725129	2.46677742873876
H	7.00979826630397	-3.22678216886176	3.42515010575658
H	6.87302944922083	-3.57134258499286	1.66795955719085
C	3.67652192736481	-1.28678206897044	1.69234643747892
H	4.24484983362257	-1.69429367457938	0.83864221513700
H	4.09891618204769	-1.74932644028600	2.59899266816721
H	2.62008221008848	-1.57570994752089	1.58589186050641
C	1.26653950672724	2.69529898066609	0.56496113268998
H	0.99478993078922	3.65380157795317	1.02972779212493
H	1.30417638012943	2.86999396206172	-0.52328653686324
H	0.48234148855799	1.95324968898299	0.76330139774304
C	4.83733038005912	2.86609201682086	3.46933209329791
C	6.02548496091661	2.96468958055067	4.22770639005921
C	3.60598148471761	3.09812950399134	4.11868244707717
C	5.98355941802395	3.28479396807549	5.59586447953478
H	6.98565835490992	2.77883986747346	3.73100025801284
C	3.55941123823819	3.41651466631641	5.48773159412408
H	2.67255418338386	3.03858137195085	3.55084829122243
C	4.74842015163618	3.51130715554173	6.23337307209363
H	6.91881135567294	3.36224524990709	6.16664457391220
H	2.58701194358955	3.59385325455045	5.96674498440404
H	4.71483045142846	3.76373583942685	7.30150423616087

C1 at 298K:

G_{298K}: -5019.38484818

P	-0.88748827847626	-0.64058846954313	0.55586347837668
O	0.42038061720240	-0.97753479958685	1.22107334695874
O	-1.08754810823704	0.61874122255791	-0.26073412204159
O	-2.07451428398368	-0.73981454401897	1.73823076427575
C	-3.37794220201697	-0.71452001519426	1.27846552298807
C	-3.93949495534968	-1.90833388758547	0.81136815852903
C	-5.28055309936169	-1.87735490245237	0.28499612995735
C	-5.89964028390169	-3.01104429415723	-0.32542457288195
C	-7.18790820843288	-2.93251740979493	-0.84082894450409
C	-7.92474037184454	-1.71607828200396	-0.77165439623044
C	-7.34434519409618	-0.58834492425466	-0.20818441932342
C	-6.01422064686633	-0.63022559124809	0.31172543935952
C	-5.37335174112511	0.55733086721696	0.76707471342637
C	-4.05990449110157	0.54632000936434	1.22128232784480
C	-3.29603077819795	1.79825107308735	1.47220338575090
C	-2.46615623248213	1.94959387150987	2.60043627484671
C	-1.60725243161402	3.05568205285295	2.70873042906078
C	-1.57500807146071	4.03780283242889	1.70969812906977
C	-2.40967200426844	3.89594744478481	0.58872744398930
C	-3.26832924976225	2.79168569677215	0.47056075528630

H	-3.86687870522522	2.65804272218395	-0.43649661426780
C	-2.24261729240115	4.84483389779539	-0.57724406482755
F	-1.30692879836144	4.36494319876375	-1.45660632723682
F	-3.38535569447020	5.01219730421510	-1.28303433726200
F	-1.81520432266873	6.07163122868910	-0.19774206098545
H	-0.88503611210447	4.88242679374957	1.78337965112323
C	-0.62474213623647	3.08367643477820	3.86007921750256
F	-1.23582374305048	2.92621469743052	5.05924835736564
F	0.09553543938902	4.22879114857548	3.91756690241796
F	0.27221746088784	2.06136728890885	3.74491262792786
H	-2.45774154260505	1.17792890721632	3.37527914391732
H	-5.92020813562584	1.50663759380809	0.71186360034722
H	-7.88706304671572	0.36510496170699	-0.17438088589791
H	-8.94309832987645	-1.66998904319826	-1.17784445323949
H	-7.63955217711841	-3.81608506621525	-1.30961192250176
H	-5.34222369934487	-3.95075719602586	-0.39129336391594
C	-3.07963624986686	-3.13205533309418	0.78262702878167
C	-1.83685401624496	-3.06462263830366	0.13637867212849
C	-0.92154237687811	-4.16636740435729	0.08652629686309
C	-1.28984364703973	-5.35491251622007	0.70854204917879
C	-2.53277188182732	-5.48289848652794	1.39380844269399
C	-2.88663509788538	-6.70180266048569	2.05138381348601
C	-4.07286172376647	-6.80790880395822	2.76428466111134
C	-4.94799181459118	-5.68851941748122	2.85572334630325
C	-4.63842714745589	-4.49326777047342	2.21841264065177
C	-3.43955448223593	-4.35596761606381	1.45276524264884
H	-5.31578677030854	-3.63760506158953	2.30314413053566
H	-5.87401678514667	-5.76844513867753	3.43934075042787
H	-4.33266446363619	-7.74737338453813	3.26841855141277
H	-2.19216294400014	-7.54959438784197	1.98796752992152
H	-0.61054465532008	-6.21609812424692	0.67851329283368
C	0.39875823480719	-4.00532752545196	-0.58113643646886
C	0.48991651521211	-3.45039109974335	-1.87626565507245
C	1.73894485905532	-3.25281499431725	-2.48194406979113
C	2.92267678888759	-3.60707182602928	-1.81258545428382
C	2.83571758505028	-4.17084307039261	-0.53029153224298
C	1.58606492073071	-4.37429504847679	0.08204910000884
H	1.53432227948188	-4.78951208880881	1.09415075034045
C	4.10071240151150	-4.61026179407228	0.17821023534210
F	4.40641735552223	-5.90098635512524	-0.11166661727695
F	3.99554681541737	-4.52414814510846	1.52625843103345
F	5.17583872975428	-3.86142546827098	-0.18775084293251
H	3.89716296132655	-3.43549242878892	-2.27950668447553
C	1.81448603647038	-2.71970191378991	-3.89852269178407
F	2.93299977676576	-1.98570197524720	-4.11306516307866
F	1.83249234543787	-3.73608782918121	-4.80097671100942
F	0.75208111030308	-1.93621576669102	-4.21399209635682
H	-0.42230949215917	-3.16549589235067	-2.40688527016617
O	-1.44510051548076	-1.87800815702040	-0.45024711355493
C	-0.29809990283369	1.97543924010365	-3.35557649378641
N	-1.32146947903453	1.44327867359951	-2.71196390924269
C	-2.72072320998637	1.48542229251542	-2.94549619610143
C	-3.44183566250938	0.34535580130750	-2.51622436060065
C	-4.83292159525913	0.30242085919471	-2.66552170608521
C	-5.51932605068944	1.40187008447369	-3.21426089303424
C	-4.80295179285552	2.54451961857833	-3.61196628655692
C	-3.40506805694650	2.59591759147614	-3.48279939009819
H	-2.87061860713101	3.50839304041622	-3.75059030071992
H	-5.33370314489908	3.41522649104676	-4.01658731408262
H	-6.61117456884875	1.37163638481168	-3.31733793981982
H	-5.38361066900588	-0.58561370802867	-2.33769764860969
H	-2.90124480823418	-0.49068927612444	-2.05915019202453
H	-1.11491075315272	0.96052712648408	-1.78275824095449
C	1.04519472377507	1.66107010870089	-2.87587117141811
C	1.32417254111788	0.44768226724493	-2.18239639891516
C	2.58798933988706	0.18094139466430	-1.67992164095503
C	3.63224705353230	1.12926691986431	-1.83647947740382

C	3.38039035096765	2.33678721148156	-2.53589788028379
C	2.11023855916889	2.58036956538483	-3.06233588960511
H	1.92962039205506	3.52563779096230	-3.58600395806943
H	4.16590422272531	3.08643657496790	-2.66089428621865
H	2.79438490217553	-0.75876574605091	-1.15896812509339
H	0.54312869333187	-0.30737555266599	-2.07535796986437
C	-0.49386791946191	2.86988911767587	-4.54295816806649
H	0.41017565562135	2.88837049900845	-5.17113951078610
H	-0.70706674523755	3.90137776667632	-4.20098893735081
H	-1.35926618771307	2.53468994499967	-5.14201623887465
C	5.89861086582834	1.73772348839141	-1.33585815428184
H	5.59435059131683	2.68928305598390	-0.86287690269472
H	6.71594282689636	1.28615650885072	-0.75326094389184
H	6.22074485924326	1.92421614648560	-2.37925309917165
O	4.81889251889535	0.79259379236939	-1.30625547698646
H	1.78747379761177	0.35453923428607	1.39342840438958
C	3.82325489330850	0.21540798093452	1.72462883027880
C	2.60101900203901	2.23664063492123	1.07838607141463
C	4.99360822214006	0.92864089726924	1.88251630709856
C	3.74906768639914	3.00016270988685	1.17205260025730
C	4.91525427137605	2.45473530989811	1.98256564494547
N	2.65022196548891	0.91500073868804	1.48171324398025
H	5.85405466124010	2.86912281966524	1.58355470041142
C	6.34769649602949	0.35615541067916	1.93823875082195
O	7.37882728189898	1.02531115965370	1.84441910809123
O	6.39163602777480	-0.99300582846453	2.10064240604037
C	3.92866387671210	4.35614226540414	0.63779980116643
O	4.99292662335831	4.97391030794268	0.69707652475356
O	2.81994674255698	4.88705003404012	0.04485953081730
C	2.92089529808660	6.20454212418088	-0.54229910301911
C	3.51019335275825	6.14861657294100	-1.94945549442013
H	3.52926831296957	6.85318955797244	0.11495861786577
H	1.88033970225395	6.57669900809232	-0.55950826158314
H	3.53461254831198	7.16704748652081	-2.38279924539765
H	4.54187863248694	5.75389145432344	-1.91918143529409
H	2.89660378346961	5.50278798045932	-2.60444722986653
C	7.69660057167595	-1.61586495085809	2.14062826520854
C	7.49634085169608	-3.09097106621711	2.45423351112978
H	8.31425656187017	-1.10945421311227	2.90733663424407
H	8.19434451366379	-1.46594231299576	1.16197248259332
H	8.47933490526047	-3.59808646825651	2.47835907593046
H	7.01144269035887	-3.21931217255135	3.44073453117581
H	6.86666419507419	-3.57426124673561	1.68637866268232
C	3.67501597336144	-1.28656509634825	1.69556059764788
H	4.24567932421847	-1.69583059161798	0.84427629942216
H	4.09488407881858	-1.74762668709232	2.60415153286167
H	2.61863732634316	-1.57530768467667	1.58751861571944
C	1.26481407114823	2.69482153711133	0.56624223474412
H	0.99309373255566	3.65304999975529	1.03159417129908
H	1.30163167912500	2.87097265989821	-0.52171809417737
H	0.48134272781475	1.95194016138597	0.76401526754256
C	4.84241705368959	2.86820130170292	3.46248452305009
C	6.03277765875394	2.96496106170621	4.21758713024915
C	3.61323476788424	3.10340889812394	4.11449261729033
C	5.99487455140567	3.28632000832110	5.58543548727818
H	6.99122548365486	2.77696012812223	3.71838893938867
C	3.57083673195588	3.42315514437416	5.48331347597263
H	2.67826484950825	3.04498205308953	3.54905921528165
C	4.76184137692421	3.51605431604776	6.22577228168102
H	6.93167732308954	3.36238330525999	6.15384856018069
H	2.59996957125020	3.60288091185638	5.96451351886425
H	4.73155150690180	3.76948436917066	7.29376984922075

7.1.2. Conformer C2

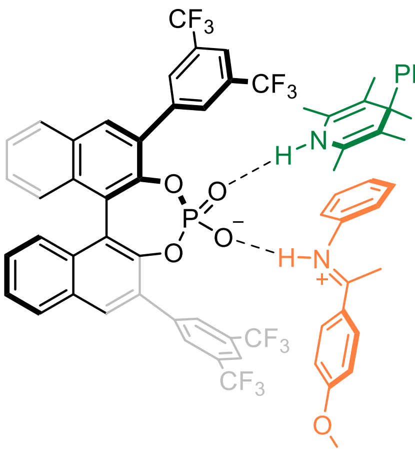

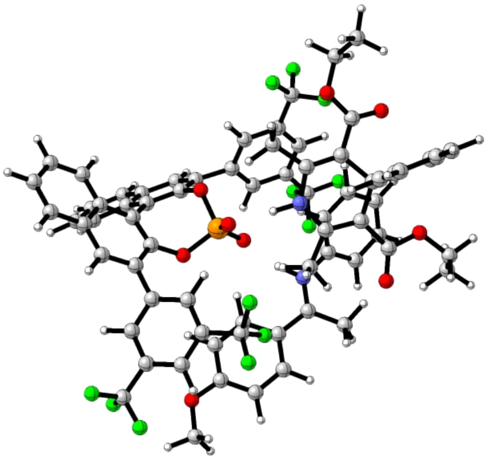

G_{180K}:-5019.3258463726

P	0.53761410674431	0.00595010221622	0.70866317021733
O	1.54641666743265	-0.92995674599167	0.07748132613059
O	-0.69037945274854	0.52368696483819	-0.01368749920290
O	1.40745095804317	1.31490107138096	1.30448511179208
C	0.78810078414417	2.09676289492361	2.26508365454456
C	0.74808030675498	1.62826627917569	3.58259733166584
C	0.05336697748128	2.40228747561488	4.58405193514618
C	-0.13304570336769	1.95759948678483	5.93070176037423
C	-0.81319116394917	2.73948538300992	6.85633569101573
C	-1.34893517235660	4.00576801902971	6.48699926323902
C	-1.21669834882759	4.45444375351004	5.18063520353244
C	-0.53413017644866	3.66669009285335	4.20225043145761
C	-0.45968515313277	4.09086714311408	2.84606879644037
C	0.17332987464826	3.32657199247832	1.87026378339094
C	0.14956861189738	3.79299238435251	0.45740361283956
C	1.33473733408517	4.04461132437121	-0.26659033816285
C	1.26429484417660	4.59834047361958	-1.55411595889949
C	0.02256733144242	4.87682779233437	-2.15485751983250
C	-1.15200214396834	4.59391800175454	-1.44926409709039
C	-1.09129302948690	4.06637360405625	-0.14791071627949
H	-2.01470908115259	3.85163903222966	0.40121364440305
C	-2.51569482563255	4.84982759592129	-2.05376091877058
F	-3.28075146357458	3.72712866381945	-2.04039470484805
F	-3.19901975529278	5.78922610884577	-1.34668626354053
F	-2.45477392999335	5.28176463879213	-3.33370129920826
H	-0.02219431238993	5.29937167190985	-3.16235440274237
C	2.51232207717338	4.99954111399797	-2.30943684035907
F	3.64658963954873	4.49049613221466	-1.77269928777077
F	2.65950789022163	6.34956960526408	-2.33554380710321
F	2.46021626174250	4.59409855843467	-3.60736157050614
H	2.30641253922284	3.83656620304160	0.19143915965723
H	-0.92602680504228	5.04332222719214	2.56489555905765
H	-1.64396245616237	5.41662774810227	4.86960194446467
H	-1.87740271496109	4.61492353019705	7.23126432537393
H	-0.94291001072556	2.37275022055864	7.88263509388065
H	0.26013426162973	0.98319070606686	6.23386793282220
C	1.30992290720212	0.28537062848123	3.90369944304169
C	0.82340312481677	-0.83611549886667	3.21559558009041
C	1.10936208361850	-2.17184448268083	3.65242994179542
C	1.97704904032465	-2.34464350857337	4.72892654214070
C	2.61403741733723	-1.24322892362479	5.36622604286296
C	3.56832508599853	-1.43748864878288	6.41259547430338
C	4.18706760167655	-0.35257929684924	7.01841301410109
C	3.87647724659016	0.97133993824857	6.59383819441347
C	2.94520089883804	1.19201651698977	5.58681380989487

C	2.28121354336591	0.09876620621757	4.94979705601005
H	2.71544118816414	2.21350347995112	5.26502771125337
H	4.38115277479605	1.82504724106066	7.06400477620333
H	4.92154357753988	-0.51096981228626	7.81827468264246
H	3.80491275248076	-2.46420519843083	6.72092124368280
H	2.20025309694859	-3.35944779687777	5.08104939685526
C	0.43204020851546	-3.35897057340196	3.05955758248187
C	-0.24325038012773	-4.25152252428864	3.91764202702306
C	-0.86833958933078	-5.40119692847884	3.40699441698053
C	-0.82137846554754	-5.68774396608376	2.03622699599390
C	-0.14139476442484	-4.80471972215142	1.18203367175322
C	0.47014647519148	-3.64121190139943	1.67811680754015
H	0.99272737201174	-2.95924159472575	0.99991884097148
C	-0.09029462981351	-5.08659505069832	-0.30399495703075
F	-1.02608075528751	-4.36585480241531	-0.97867573894457
F	-0.31770247001339	-6.38927047661876	-0.59640853743362
F	1.11479492542492	-4.76008298891397	-0.83709288123407
H	-1.30535809714633	-6.58385360874276	1.63906824373666
C	-1.59138391830953	-6.31177983043138	4.37574813610951
F	-2.59391115299729	-5.65632366808767	5.02076246003084
F	-2.14649250053641	-7.38964180729802	3.77283327813241
F	-0.75410450081582	-6.77712250448572	5.33840580190801
H	-0.28755030437710	-4.03687134227985	4.99140065919412
O	-0.04033327872872	-0.65843232793880	2.15193725353851
C	-2.98828764107535	-1.32816426879081	-2.19913841853522
N	-2.26827343403651	-0.21510121297733	-2.09551931449118
C	-2.00272357067825	0.76780700812326	-3.09201648526097
C	-2.99095736120766	1.20680961802771	-3.99916624218246
C	-2.66752753932463	2.17494875407553	-4.95942485131979
C	-1.37026586931571	2.71516871580811	-5.01723665300354
C	-0.40098625578330	2.30291209597639	-4.08753963465851
C	-0.71414988860110	1.34099313983998	-3.11856549213195
H	0.02856930639986	1.02582761005125	-2.38260327328575
H	0.60981304931101	2.71988475015094	-4.11806884971213
H	-1.12111813722114	3.47075883857353	-5.77211661977880
H	-3.43934658708443	2.51507037798098	-5.66020071314910
H	-4.00963173895653	0.81389518976307	-3.93596543187802
H	-1.72495048929941	-0.02792012120719	-1.21228417111790
C	-3.24879035931711	-2.15332039888142	-1.01988765682529
C	-2.83241002020145	-1.77830722228902	0.29583328101969
C	-3.05405204517088	-2.60179059546817	1.38996555092840
C	-3.73274819796191	-3.83857360019821	1.23523648191337
C	-4.18681391461820	-4.21938167862537	-0.05057240331659
C	-3.94468016539991	-3.38946292605161	-1.14553155121598
H	-4.30920151418865	-3.73050039408334	-2.11830578399176
H	-4.72066529582963	-5.16175221587511	-0.20260550263097
H	-2.71045955943016	-2.30840041658337	2.38762513703268
H	-2.32110479961782	-0.82976659316523	0.47474487339465
C	-3.49924615897578	-1.75941112783860	-3.54705028307779
H	-4.57543635812400	-1.52310236693532	-3.65231599738206
H	-3.38193698360169	-2.84860970481899	-3.65922489590240
H	-2.94195755780739	-1.27700043917643	-4.36256596578404
C	-4.58876095377840	-5.82586110567203	2.27387876476602
H	-4.03911120315946	-6.52251689841001	1.61212893748685
H	-4.60786906935260	-6.22106308713891	3.30051178979994
H	-5.61953119468889	-5.68798039926730	1.89360833984461
O	-3.90686942111136	-4.56522634804869	2.35256706130192
H	1.91655704031646	-0.86695670320075	-1.70459850817591
C	1.05124869798745	-1.24420060391313	-3.54177039747730
C	2.80249706291800	0.40671371353606	-3.06730599498748
C	0.95114973942588	-0.84353909187230	-4.85674598390056
C	2.79199640433840	0.85909192922766	-4.36757045692073
C	1.96032721321030	0.14906579909077	-5.44052627684412
N	1.99279728049659	-0.65990427478134	-2.71564986855766
H	1.41768708726848	0.92155903270007	-6.01092896911056
C	0.21841102247022	-2.28133502707473	-2.83903072150018
H	0.05699422226116	-1.98971635446944	-1.78812362711289

H	-0.73773913444085	-2.45029957124282	-3.34640329828607
H	0.76399315688622	-3.24360544280771	-2.82894405916237
C	3.60181969810776	0.97420772494854	-1.91612877691697
H	3.50813975530707	2.06938902724681	-1.88108334898630
H	3.25923963802641	0.54818378965480	-0.95847716084561
H	4.67655421496965	0.75144531036215	-2.04863234357983
C	-0.09859602788793	-1.36048016612911	-5.75137832684238
O	-0.86415218875156	-2.30323891103822	-5.55043628535991
O	-0.14413794809043	-0.64247149621659	-6.91490791083123
C	3.54625536573483	2.01890555575877	-4.87531864311291
O	3.28323469912595	2.57916703231315	-5.93839822085169
O	4.58294629428787	2.40811060605013	-4.08561571282759
C	-1.12105896311336	-1.01801006545669	-7.91195996935788
C	-2.47381032498954	-0.36379605644241	-7.64485934764038
H	-1.21169383497821	-2.11980852576109	-7.93904028619344
H	-0.69033923943529	-0.66754214137743	-8.86720690898628
H	-2.37351254001508	0.73627823682528	-7.59441349409578
H	-3.17616569851303	-0.61846105606405	-8.46183586612174
H	-2.89898843006623	-0.72152742618474	-6.68997738905618
C	5.36988198863327	3.54771979353848	-4.50863259722149
C	6.45306530379617	3.13276261915186	-5.50036791396289
H	5.80544089224554	3.94393441065285	-3.57485724060367
H	4.70114312089712	4.31111236635391	-4.94369862158406
H	7.08417707762499	4.00768514665169	-5.74958717609366
H	7.09939940963583	2.34535780647106	-5.06773273074977
H	5.99710142995898	2.75096428674500	-6.43206179471441
C	2.89809900857503	-0.56488334548915	-6.42301736924557
C	3.02344231641817	-0.14153340474659	-7.76041950260476
C	3.67064645782073	-1.66005773811409	-5.98234858990933
C	3.90002026917536	-0.79870410557170	-8.64255808838041
H	2.42694085914883	0.71212811027053	-8.10488260589807
C	4.54834686775267	-2.32141084932976	-6.85855646596393
H	3.57669045794527	-1.99396147653267	-4.94055109385460
C	4.66557761636232	-1.89157778953768	-8.19432492664560
H	3.98608394711235	-0.45784085519241	-9.68312976327763
H	5.14162388307257	-3.17332556936004	-6.50017192736517
H	5.34982847961515	-2.40586070206175	-8.88209094231051

C2 at 298K:

G_{298K}: -5019.38040373

P	0.55187719127790	0.00320811269150	0.70050366266334
O	1.56127523833128	-0.93285842256402	0.07130705548520
O	-0.67523896787198	0.52066129259982	-0.02409462443852
O	1.41952951700942	1.31281424847919	1.29721646533453
C	0.80323992626817	2.09007948270203	2.26292316713230
C	0.77085455687527	1.61804847890930	3.57933334841444
C	0.08179292830988	2.38934761590625	4.58670190828748
C	-0.09598344198717	1.94165748324829	5.93352050682378
C	-0.77074835470027	2.72098631785557	6.86512501453979
C	-1.30978105477852	3.98756519412210	6.50194830979583
C	-1.18619391529797	4.43902939776289	5.19575607965619
C	-0.50923836773066	3.65411205652379	4.21133306456438
C	-0.44463651309238	4.08073686270785	2.85534631384304
C	0.18338642619281	3.31913825708549	1.87449605790545
C	0.14707844942772	3.78458848760664	0.46147634115573
C	1.32525122906271	4.05508973964689	-0.26657753668813
C	1.24139268533187	4.60506622290380	-1.55497351664540
C	-0.00688847433695	4.86083499751408	-2.15205383675257
C	-1.17425132700320	4.55837818923913	-1.44245644973218
C	-1.10021953079170	4.03468222509850	-0.14026339271562
H	-2.01779182308840	3.80381367581452	0.41198737947388
C	-2.54405547410066	4.79037362581815	-2.04287876410076
F	-3.29284623534804	3.65685792050816	-2.01925056755558
F	-3.23785146876844	5.72413689876255	-1.33867088582687
F	-2.49446630983821	5.21503884281955	-3.32569072281729
H	-0.06254362646865	5.28060902783603	-3.16015173095671
C	2.48062783667679	5.02734543092683	-2.31351228403115

F	3.62366609503502	4.53017932807431	-1.78409874232671
F	2.60937924387851	6.37930609559073	-2.33179107556891
F	2.42864004348677	4.62892924109257	-3.61333633408813
H	2.30180611433192	3.86411883812130	0.18858897659508
H	-0.91525717686891	5.03235573712524	2.57836706848816
H	-1.61627998924312	5.40154289608097	4.88962542303172
H	-1.83400626464551	4.59475641528345	7.25080579437205
H	-0.89361992355332	2.35192343771085	7.89142577805649
H	0.29993216340064	0.96696573967198	6.23205492124398
C	1.33221006528854	0.27306108955577	3.89236882316637
C	0.83843803352825	-0.84457760132861	3.20301507773927
C	1.12158212367774	-2.18245804513479	3.63466189272560
C	1.99664965037409	-2.36145458600973	4.70416589966841
C	2.64202672894693	-1.26433329890660	5.34028489026412
C	3.60331076326289	-1.46483067916144	6.37893440573181
C	4.22879531527998	-0.38379991689684	6.98462906092278
C	3.91815537390819	0.94246817411575	6.56765456882699
C	2.98040584802460	1.16923315190118	5.56810962315952
C	2.30959944154085	0.08004389475579	4.93138453759425
H	2.75051832450232	2.19238167845799	5.25178617078184
H	4.42802390576892	1.79315389899448	7.03766695299931
H	4.96864645691168	-0.54706644032176	7.77852975677288
H	3.83986043348142	-2.49324508142273	6.68159321518521
H	2.21836988715814	-3.37810080658851	5.05190380602250
C	0.43072335081728	-3.36507979330612	3.04818030901425
C	-0.24288485771612	-4.25054153998465	3.91545640113486
C	-0.88206696804942	-5.39647369948472	3.41490110963058
C	-0.85110014040785	-5.68664225887902	2.04439648877706
C	-0.17499788682981	-4.81053887125377	1.18068777028328
C	0.45099087203633	-3.64998050156441	1.66693437652558
H	0.97140657003830	-2.97343977524382	0.98125021458151
C	-0.13775122031480	-5.09535073812536	-0.30515911370068
F	-1.01737205949678	-4.31053341056963	-0.98279175903782
F	-0.45302091307302	-6.37854818595280	-0.60097736220266
F	1.08983414705781	-4.85199296243308	-0.83240696836520
H	-1.34534269582990	-6.58027116039875	1.65476409919443
C	-1.60526258616556	-6.29755344094803	4.39235425276432
F	-2.60308700933536	-5.63373161873003	5.03608708030109
F	-2.16729311744855	-7.37680425100960	3.79775018844968
F	-0.76706704994043	-6.76024530790480	5.35501618638300
H	-0.27473614065496	-4.03241633395967	4.98895691319231
O	-0.02851037635839	-0.66067547344396	2.14332267034297
C	-2.96645729903318	-1.33221122952685	-2.20243178548651
N	-2.25107913531309	-0.21569791107582	-2.10426617277777
C	-1.98319830262626	0.75812904939821	-3.10885050746797
C	-2.96786222386831	1.18206008374928	-4.02700880498992
C	-2.64433975834911	2.14317000407154	-4.99402840573153
C	-1.35024671542500	2.69144511368199	-5.04779503497933
C	-0.38496099199104	2.29433491961989	-4.10764998132142
C	-0.69842945555097	1.33956161317324	-3.13173466241226
H	0.04111992601372	1.03633416892350	-2.38757947083220
H	0.62327000011922	2.71737608169743	-4.13575431787005
H	-1.10025858892019	3.44106772283540	-5.80832485617854
H	-3.41344036408728	2.47165966542865	-5.70326583696302
H	-3.98449115139847	0.78353808252251	-3.96653505893954
H	-1.71304941534592	-0.01865273560580	-1.21931211932008
C	-3.23773903157337	-2.14486812844463	-1.01681317738182
C	-2.82433861840571	-1.76217510233465	0.29751019200770
C	-3.05340190423473	-2.57683617115066	1.39669727377194
C	-3.73730946246912	-3.81135010350509	1.24879977917991
C	-4.18946956085350	-4.19908996452712	-0.03559938370638
C	-3.93936375554436	-3.37831688789342	-1.13575293522981
H	-4.30210832367795	-3.72428164675123	-2.10745671731613
H	-4.72766526568301	-5.13983290292371	-0.18250775674707
H	-2.71032131899731	-2.27847327461534	2.39300257248154
H	-2.30649381192881	-0.81626226012858	0.47134164289643
C	-3.45970727655042	-1.78147653738664	-3.55119562660288

H	-4.53522284071644	-1.55067435593238	-3.67379454925219
H	-3.33657849074792	-2.87159342364395	-3.64776525909659
H	-2.89186247575403	-1.30859549325535	-4.36515741476398
C	-4.60584738536159	-5.78644370834489	2.29972365909880
H	-4.06008954973535	-6.49072172485639	1.64270692543144
H	-4.62766300634232	-6.17540654080285	3.32871482507451
H	-5.63583827591410	-5.64576616842102	1.91804422339452
O	-3.91757678558434	-4.52956677820326	2.37091218340436
H	1.94573638206444	-0.85511369480147	-1.70549840093976
C	1.08139719735942	-1.23945198914441	-3.54023031180007
C	2.81562302116515	0.43046118677760	-3.06823816969070
C	0.97443033066713	-0.84010183893019	-4.85512624687131
C	2.79996197938679	0.88056148042857	-4.36891944780761
C	1.97838365917498	0.15695043742385	-5.44066642800824
N	2.01969553156078	-0.64647922409974	-2.71646436054419
H	1.43173768524851	0.92209935384261	-6.01684184048761
C	0.26292116676679	-2.28217866697164	-2.82976249476382
H	0.03702875813155	-1.94842113714718	-1.80339190747859
H	-0.65686588884601	-2.52419198702449	-3.37309870456224
H	0.85814111975214	-3.21026076698395	-2.73803065585723
C	3.60520149265829	1.01008254037745	-1.91641352912180
H	3.50133424328022	2.10448038816831	-1.88631163828286
H	3.26286286571578	0.58462798545088	-0.95825477076238
H	4.68255183745702	0.79696485927018	-2.04374758148070
C	-0.07408959541672	-1.36087388822421	-5.74942542915455
O	-0.85352639464636	-2.29065986780386	-5.54091689474713
O	-0.10246182747177	-0.66145765232016	-6.92475435598257
C	3.53860208914062	2.04982372504039	-4.87829920925993
O	3.26595667552384	2.60775999730190	-5.93976922767477
O	4.57279122503173	2.45099743680665	-4.09068801933264
C	-1.07518161922512	-1.04383697801305	-7.92271796649346
C	-2.42678109957388	-0.38018906930484	-7.67256424175812
H	-1.17129458431099	-2.14546593962319	-7.93696648063897
H	-0.63670733287571	-0.70690642885046	-8.87929442516734
H	-2.32129168115126	0.71984241968689	-7.63340545045628
H	-3.12458285546908	-0.64039286409374	-8.49175896698825
H	-2.86012577380600	-0.72554255341965	-6.71685003180704
C	5.34579383481803	3.59826151260635	-4.51794933326564
C	6.42711934138801	3.19459327176843	-5.51642860753357
H	5.78281304728171	3.99855498723922	-3.58655263635731
H	4.66697277336433	4.35494806848530	-4.94913033997259
H	7.04793327306784	4.07588278554361	-5.76921200060091
H	7.08393990143515	2.41344519739147	-5.08825879872825
H	5.96900968785198	2.80864310266136	-6.44527777260151
C	2.92705729713939	-0.55276910373702	-6.41551666746705
C	3.06892844485055	-0.12062238332430	-7.74845246950606
C	3.69523859287321	-1.64978461758605	-5.97203018948491
C	3.95740514924669	-0.77110799025721	-8.62343761712258
H	2.47644901026887	0.73521644058240	-8.09424721475712
C	4.58465509790331	-2.30449727085822	-6.84126415935888
H	3.58852382225533	-1.99062253436627	-4.93375758167470
C	4.71835713838011	-1.86603010065844	-8.17258444442281
H	4.05639672530000	-0.42335189260570	-9.66056880621890
H	5.17430635784722	-3.15803869748326	-6.48078480166580
H	5.41186683792021	-2.37518799508668	-8.85486952416292

7.1.3. Conformer C3

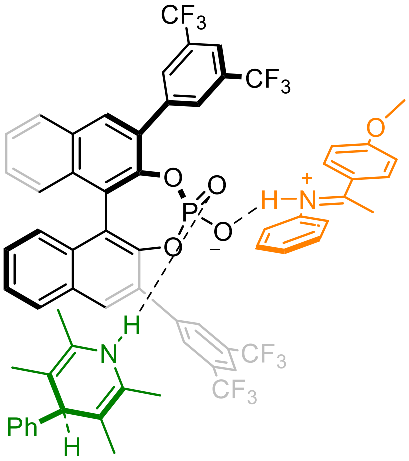

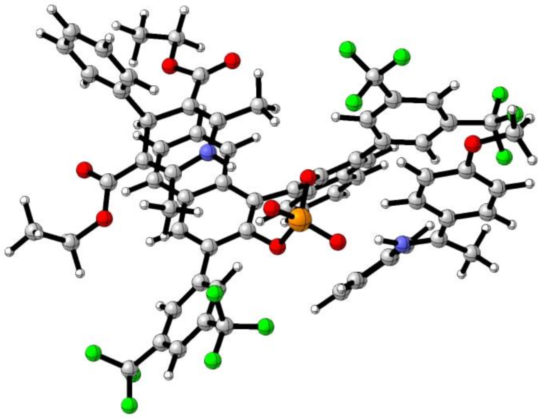

G_{180K}:-5019.32702812202

P	-0.80187675372609	-1.00183197505672	0.02162525152799
O	-0.22750012093763	-1.50696528443243	-1.28176365013113
O	-2.18753219911018	-1.41487600694868	0.48310793454450
O	-0.65405197892007	0.66666258660138	-0.05007075484791
C	-0.61894777402110	1.48299522503884	1.05516620598920
C	0.47251224011068	1.39367558709923	1.92649692762427
C	0.45760585899446	2.16661473186963	3.14357775106523
C	1.43060939490282	2.00742108604962	4.17659390160129
C	1.37548938512073	2.77293091742286	5.33488818552700
C	0.35037894709360	3.74328147876519	5.51686470033638
C	-0.62125008093581	3.90985704062189	4.54072631473324
C	-0.60254037777161	3.12213201849931	3.34988630417472
C	-1.62065882070698	3.25519926688863	2.36964074669367
C	-1.66978261004352	2.45073676222000	1.23245040376658
C	-2.75273436365157	2.66239737115544	0.23420939686037
C	-2.51174095936199	2.56061894537604	-1.15252186148282
C	-3.52480560983768	2.83786728946907	-2.08135475330600
C	-4.79923196283373	3.24485968794517	-1.66150863075322
C	-5.04416695609967	3.36336393620201	-0.28391503737132
C	-4.04251948872882	3.06257347571287	0.65196323512668
H	-4.26575295110223	3.13619142411958	1.72091389531632
C	-6.37475509127588	3.89680487604614	0.20326390359770
F	-6.76621187293053	3.32445287387731	1.37243114733254
F	-6.30543682923292	5.23483401032224	0.43613014511329
F	-7.37463821124109	3.70765874645734	-0.69109004216903
H	-5.58704533516308	3.45544918354348	-2.38942024537859
C	-3.19845879227958	2.64663333255507	-3.54672524743100
F	-2.12187098991571	3.38512951704658	-3.92001298783387
F	-4.22186896447213	2.97757897360004	-4.36421248664316
F	-2.87818187356087	1.34651774815763	-3.81143449830312
H	-1.52240413335683	2.27553957038683	-1.51143522774804
H	-2.38764809929778	4.02319852881368	2.52350817191325
H	-1.43357732220689	4.63594174596666	4.67340684480078
H	0.32343066286625	4.34547331321767	6.43385170194138
H	2.12820388605316	2.62367029524130	6.11958986833097
H	2.22191215407794	1.26088143946328	4.05577234094763
C	1.64163680985675	0.52503694247838	1.59971684160087
C	1.47361646978096	-0.84719554638677	1.37689011844327
C	2.58799794357609	-1.75099129669183	1.28538084192369
C	3.86886494328544	-1.20160325493403	1.31376759659359
C	4.09432167871182	0.19718955727819	1.39642537865474
C	5.41666629358482	0.73330638445609	1.32921690795180
C	5.62737057858000	2.10251142138770	1.38197933749254
C	4.51736925469798	2.98538992162725	1.49048629393544
C	3.22136921789087	2.49265451427632	1.56782149299220
C	2.96860851364180	1.08751522652519	1.53681204990422

H	2.37861960751511	3.18795762361344	1.63062998478401
H	4.68625052512916	4.06889415078474	1.49669411122274
H	6.64328721047558	2.50991092522585	1.31120209167250
H	6.25495619024441	0.03902157691823	1.20048627300565
H	4.73853720583149	-1.85981854547567	1.21927548270121
C	2.43711183051856	-3.22980227405707	1.20727094180731
C	1.44583729944992	-3.85410447108430	0.42154608505449
C	1.39865779686527	-5.25125764635117	0.30026931738951
C	2.33442008282312	-6.06817119341330	0.95130681595160
C	3.32362639821098	-5.45430349573081	1.73811740758836
C	3.36853702447491	-4.05794170185345	1.87485584597246
H	4.14294731722953	-3.60534625524589	2.50263716538120
C	4.41265145426232	-6.28784844411624	2.37927893413511
F	4.05062457902524	-7.58066870922965	2.54780365497073
F	4.77933078496210	-5.80896843184178	3.59299111290594
F	5.53836553111277	-6.29193640749901	1.60743706968119
H	2.30002402686971	-7.15595851529066	0.84406641455581
C	0.33492287356916	-5.83350429487851	-0.60603230124535
F	-0.90930018198736	-5.43991894559880	-0.22942829991745
F	0.50051670263278	-5.40971756146958	-1.88944677713416
F	0.33993258116986	-7.18567195279709	-0.62889096298811
H	0.74898019077784	-3.25111678687136	-0.16129427872384
O	0.19378924501332	-1.36045039521507	1.33536556680670
C	-5.11150841930649	0.13220966668501	2.00257974968308
N	-3.82472829740800	-0.12008851175104	2.21660534933500
C	-3.06794499743220	0.03489717649428	3.41208889770458
C	-2.05205014428614	-0.91185518659758	3.66646314490547
C	-1.23750394828480	-0.76717984281863	4.79794430113664
C	-1.42806695647052	0.31364518147476	5.67773735256080
C	-2.43080558786657	1.26195602622521	5.41108236703563
C	-3.24346296472007	1.13688386386397	4.27551873118958
H	-3.98058323586004	1.90907494531880	4.03889553546498
H	-2.56379691979178	2.12481595062011	6.07310548977223
H	-0.78315446728873	0.42951855298880	6.55682580214283
H	-0.44290127754424	-1.49990682185025	4.98562655633069
H	-1.90105512727171	-1.73557956813192	2.96337906938009
H	-3.23992816366391	-0.54251589587671	1.44357030450475
C	-5.64589601892498	0.13244454934155	0.64337993639170
C	-4.81722723260342	-0.03808353460442	-0.50843219374520
C	-5.32789049694924	0.08313147771206	-1.79050020814674
C	-6.69260679394338	0.41352243044274	-1.99333400306923
C	-7.54363752647276	0.55429185548559	-0.87079621485811
C	-7.02259202935451	0.41228180045393	0.41454902397383
H	-7.70973760202838	0.55945934225700	1.25148576905810
H	-8.60171543825765	0.80086127134757	-0.99307627185270
H	-4.67427972986368	-0.01879240848628	-2.66223223783288
H	-3.75048969946377	-0.24692268490823	-0.40701357528915
C	-6.01882707160760	0.43415471740447	3.16390072862687
H	-6.25935287390316	1.51297346385504	3.19408172506104
H	-5.56458501148932	0.14247798169629	4.12257895033028
H	-6.96906396007015	-0.11195285732371	3.03913170127683
C	-8.41880395663888	1.01858943823782	-3.54288256334451
H	-8.48236217000575	1.13968865049423	-4.63644139006128
H	-9.14694384162365	0.25649600976853	-3.20294186449336
H	-8.62985264732383	1.98210285222852	-3.03924100233298
O	-7.07438199616095	0.59453494313912	-3.26884915081799
H	1.25659926289897	-0.65712184088780	-2.22137032411472
C	2.28446005508604	1.12969088226576	-2.20002634415812
C	3.25735854253687	-1.11799862281037	-2.12733116816895
C	3.51697931352280	1.72107242096531	-2.02699449285103
C	4.52455045352722	-0.60946446478804	-1.92411847483496
C	4.79544381132009	0.88626862942495	-2.11821460981258
N	2.19773336386172	-0.24664651589752	-2.31202735667768
H	5.49036703099923	1.21361997178421	-1.33035248803721
C	0.96829204341990	1.85614921716268	-2.32225436943010
H	0.14599729716100	1.14678818568446	-2.51119681520274
H	0.74867003656056	2.42403270212603	-1.40333907286193

H	1.01580513501963	2.59609818562176	-3.14087298860524
C	2.87451728476217	-2.57487483205077	-2.21791793015058
H	3.43148464534212	-3.05612789028024	-3.04261360767287
H	3.14969032206426	-3.11460636807287	-1.30229155514164
H	1.79224631954181	-2.69017046292083	-2.38823218069474
C	3.63115648413953	3.17199794308075	-1.81981337325723
O	2.71304028739564	3.98937514413654	-1.75556538567319
O	4.93440259577235	3.55477287115735	-1.68650979264149
C	5.70730581934230	-1.38910760287581	-1.53352527552127
O	6.81737331565752	-0.88881801014181	-1.34717041274513
O	5.47839182693413	-2.71988205711098	-1.32967795941945
C	5.19046011519914	4.94886919155301	-1.42287631152885
C	6.68350378228407	5.10660964953771	-1.17872891016633
H	4.59352369124764	5.26510745881526	-0.54565339336450
H	4.84807166963627	5.54801646474984	-2.28994075374857
H	6.91665370625613	6.16997141978159	-0.98180665891403
H	7.00058216987755	4.50818928525106	-0.30395794509525
H	7.26303212816816	4.77645682028350	-2.06153035950799
C	6.57926730268949	-3.54206045586975	-0.87493204905479
C	7.45423892473548	-3.99948134276593	-2.03861555633952
H	6.09620446295071	-4.39789651513852	-0.37173396957154
H	7.17237038696286	-2.97754791581451	-0.13170774401610
H	7.94073748930910	-3.13172250951716	-2.52009349773174
H	8.24105103518843	-4.68338790804184	-1.66577625464738
H	6.84970405700425	-4.53820002988532	-2.79307095121289
C	5.49289106846794	1.11821189475914	-3.46429977145181
C	6.81388652064920	1.60236441069983	-3.52994521465588
C	4.81305977938756	0.83841065754805	-4.66840578751472
C	7.44529929405299	1.80447659149066	-4.77013558893961
H	7.34528605702532	1.81950297492610	-2.59535108529079
C	5.43778705059769	1.03796036714047	-5.91174796809538
H	3.78274707072701	0.46129674695495	-4.62530735806311
C	6.75864894325798	1.52275374021187	-5.96638413279327
H	8.47574096277614	2.18332201669472	-4.80423789948197
H	4.89431420018757	0.81548956491456	-6.83987888607378
H	7.24978050209568	1.68010826310773	-6.93580815562392

C3 at 298K:

G_{298K}: -5019.38192147

P	-0.81522627842731	-1.00437280895305	0.03584246104733
O	-0.22120067493029	-1.48789660312626	-1.26649391873650
O	-2.20070145118579	-1.43660531305306	0.48074532212970
O	-0.69293232320019	0.66800873901797	-0.02038955118075
C	-0.65684994458614	1.47996081582453	1.08658409946808
C	0.43250667916538	1.38907599422028	1.96008459370774
C	0.41362839817134	2.15657498468004	3.17994658089226
C	1.38678551826213	1.99490830421371	4.21228535423456
C	1.32799441519022	2.75327755789375	5.37503850343742
C	0.29938760027744	3.71914881193189	5.56145677630573
C	-0.67072582917162	3.88988011603308	4.58441122115936
C	-0.64836579053001	3.10948101826447	3.38892753185164
C	-1.66171143948831	3.24870633177265	2.40445221177976
C	-1.70560511179702	2.44997240138113	1.26315662898071
C	-2.77841951115428	2.67066930144275	0.25651942118895
C	-2.52281913396475	2.58058241781474	-1.12864813390116
C	-3.52691783926355	2.86551602806557	-2.06486293115399
C	-4.80550764206575	3.26875764161392	-1.65370209959411
C	-5.06457891052801	3.37556623369001	-0.27779041576008
C	-4.07191225897548	3.06722226290437	0.66509636073474
H	-4.30480542500730	3.13238642222631	1.73255819785860
C	-6.39996064436289	3.90402426544082	0.20123341214165
F	-6.80186477011572	3.32046091835313	1.36190246328134
F	-6.33435359623161	5.23952582564987	0.44684044589504
F	-7.39146480095904	3.72159504232583	-0.70390650200051
H	-5.58590260814340	3.48543376246351	-2.38776960231601
C	-3.18763819210948	2.68632245137372	-3.52914974052720
F	-2.11159844674419	3.43176558921120	-3.88797615585951

F	-4.20647028681125	3.01951430177495	-4.35180580441611
F	-2.86082981266899	1.38963798236565	-3.80029594089004
H	-1.52957563948820	2.29688194127393	-1.47924454496948
H	-2.42801974506953	4.01778764241880	2.55679927530634
H	-1.48394838381569	4.61456026007344	4.71974660135915
H	0.26961445902289	4.31602117469636	6.48184206547285
H	2.08081306645737	2.60253674817412	6.15934236320765
H	2.18111955941563	1.25230602476935	4.08614739292878
C	1.60745224483083	0.53162398928261	1.62630240458955
C	1.44996098119718	-0.83969633528009	1.39503540659432
C	2.57139082545252	-1.73222590785281	1.28509961001589
C	3.84675797664402	-1.16959419863677	1.29194056646451
C	4.05912288920489	0.23141083384370	1.37748450134156
C	5.37271444161718	0.78306201366940	1.27871273736668
C	5.56955518737964	2.15429510016918	1.33374552073678
C	4.45380062001158	3.02345152632990	1.48196952052110
C	3.16587965302374	2.51559467623767	1.58912765998715
C	2.92731448354190	1.10851164866487	1.54949699030791
H	2.31826668940020	3.20169778929633	1.67953071850778
H	4.60881864364656	4.10915545263087	1.49356857561222
H	6.57857792316853	2.57283135877409	1.23580289346653
H	6.21514833835636	0.09943562480822	1.12218840131137
H	4.72081800256739	-1.81904921229906	1.17798852605649
C	2.43342574309632	-3.21242255441361	1.21013259587516
C	1.44224568337055	-3.84734244494069	0.43305598004737
C	1.40802741220155	-5.24495099126252	0.31387403652584
C	2.35591590543728	-6.05151200451051	0.96016937632265
C	3.34447882597080	-5.42724177633236	1.73959268240729
C	3.37770820644866	-4.03009597857104	1.87246136582968
H	4.15220835927822	-3.56823505130492	2.49339774916066
C	4.44276769229850	-6.25122151043840	2.37744911754757
F	4.08670357281872	-7.54388378519837	2.55978669224709
F	4.81844430082896	-5.76101586474502	3.58377659608278
F	5.56160013475266	-6.25820425672036	1.59576951620584
H	2.33091570788818	-7.13979857760468	0.85531762786143
C	0.34671767175657	-5.83907903276199	-0.58783553970837
F	-0.89906511027243	-5.44937247389797	-0.21383484781639
F	0.51081518620487	-5.42399270846526	-1.87398652939103
F	0.35914408461119	-7.19156240060191	-0.60055306812460
H	0.73606550707854	-3.25210272435132	-0.14638916786967
O	0.17305101245413	-1.36136284885296	1.35497704864564
C	-5.12459811365928	0.11417865958836	1.98731261636122
N	-3.84173817048751	-0.15083742987051	2.20813680028916
C	-3.09091115117353	-0.00865536381409	3.40888376057808
C	-2.08289957624834	-0.96394621645514	3.66286999667956
C	-1.27181297623786	-0.83032480823414	4.79808208028155
C	-1.45813242950927	0.24755217917814	5.68238559044986
C	-2.45384754511050	1.20355550520181	5.41719648223048
C	-3.26263794645857	1.08970887058617	4.27777867322462
H	-3.99369039237203	1.86807108300047	4.04264762285674
H	-2.58368835242916	2.06370720010506	6.08328921120163
H	-0.81562210232463	0.35477752791077	6.56432753604545
H	-0.48288414005299	-1.56929643822105	4.98497052548508
H	-1.93444330410287	-1.78492532501832	2.95602375578536
H	-3.25459373875700	-0.57492196245078	1.43596292092191
C	-5.65040437219177	0.12821352257191	0.62486098699831
C	-4.81428446039382	-0.03378873035083	-0.52283106427598
C	-5.31558683176988	0.10135676855233	-1.80713005543794
C	-6.67801084778215	0.43689719642880	-2.01605938084231
C	-7.53671958608646	0.56814942222437	-0.89822038607276
C	-7.02478404182043	0.41325051494734	0.38932292276496
H	-7.71737465186662	0.55366428631195	1.22293193522183
H	-8.59349219592680	0.81770502809056	-1.02547191899657
H	-4.65610236633074	0.00609886446766	-2.67512522129080
H	-3.74915515005763	-0.24759561282803	-0.41556129920958
C	-6.03779561575356	0.41550744272709	3.14464231163689
H	-6.27678650738936	1.49462865233328	3.17606490474894

H	-5.59010988545029	0.11987419136776	4.10516193760390
H	-6.98834998899676	-0.12849548835480	3.01252758324028
C	-8.39150078877468	1.06035188696527	-3.57217572187790
H	-8.44704286689283	1.19190930851015	-4.66497280411304
H	-9.12361468136180	0.29640401355027	-3.24491844768707
H	-8.60505607797132	2.01947702734635	-3.06115841398978
O	-7.05029648286159	0.63164065081260	-3.29244299675143
H	1.28503734597212	-0.64864577641327	-2.15954418282467
C	2.32961195029607	1.13041010569612	-2.14930666699342
C	3.28490471006904	-1.12534852243788	-2.10367072649308
C	3.57123658587505	1.71102915638171	-2.00831612382178
C	4.56068925402087	-0.62707489523393	-1.92872274394195
C	4.83868885632793	0.86515160339088	-2.13677984569738
N	2.22862228901100	-0.24530338292222	-2.26176344935377
H	5.56124144073620	1.18803720707268	-1.37256694400597
C	1.01667369860283	1.86838324075560	-2.23422767659004
H	0.18522727314749	1.16758187009127	-2.41395892756699
H	0.82003179893836	2.42725099218678	-1.30426819665173
H	1.05334733366104	2.61831079162070	-3.04412394463664
C	2.88871609541390	-2.57894778657185	-2.18884526673167
H	3.42074310453629	-3.06118327826454	-3.02941218068540
H	3.18249943238560	-3.12455253151640	-1.28262465681054
H	1.80169073723911	-2.68557294103190	-2.33274945952949
C	3.70462760114978	3.16138661036007	-1.80851685856978
O	2.79757995552391	3.98808669406178	-1.72038822144169
O	5.01615171392529	3.53098017488789	-1.72017373674015
C	5.74322497340016	-1.41239888663457	-1.55044161738519
O	6.85712844602431	-0.91758582853522	-1.37258958636551
O	5.50990506932655	-2.74263952364441	-1.34356871530628
C	5.29655925829688	4.92487183540412	-1.48603500319332
C	6.80573416246769	5.07774641842430	-1.37113781792736
H	4.77846463302029	5.24583094319421	-0.56150518204638
H	4.88200349420509	5.52189501068105	-2.32223310859596
H	7.06019005695114	6.14070388133180	-1.19996395066278
H	7.19443236747047	4.48117489512649	-0.52420484042620
H	7.30474122389436	4.73977449661092	-2.29892242280458
C	6.61269042148050	-3.56867037980592	-0.90113451777168
C	7.47441051425770	-4.02713766635573	-2.07430648456100
H	6.13197264038891	-4.42348953180823	-0.39411720017429
H	7.21543915779604	-3.00671981326746	-0.16366511291901
H	7.95937802538706	-3.16013090038313	-2.55857824964134
H	8.26218297625727	-4.71509082377652	-1.71097016904348
H	6.86022031790452	-4.56163674188692	-2.82396795421327
C	5.49900028889456	1.08439494082079	-3.50354108394863
C	6.82938142283690	1.53507829657526	-3.60711713461182
C	4.77764670855810	0.82488858340690	-4.68789345923423
C	7.42921483480732	1.72497430179917	-4.86469019270034
H	7.39298516478480	1.73535587549874	-2.68782958249505
C	5.37084521291030	1.01211037855747	-5.94840226087038
H	3.73966000151272	0.47407693990617	-4.61672327214842
C	6.70115153526326	1.46395488482702	-6.04084855445475
H	8.46732531524618	2.07826371822718	-4.92790339578843
H	4.79492961568912	0.80581790245183	-6.86056470979254
H	7.16750132130544	1.61197907107265	-7.02389126368494

7.1.4. Conformer C4

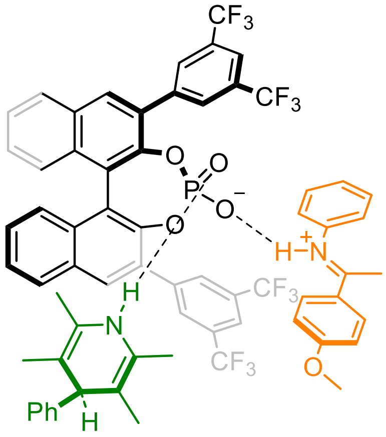

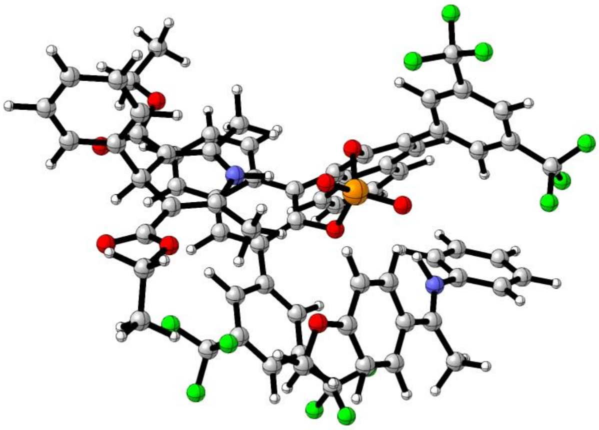

G180K:-5019.32338919006

P	0.03275239632947	0.07176877378331	0.15323991579432
O	1.53963984162523	0.09048609644905	0.20498490015364
O	-0.77584462524633	1.35468299350046	0.11210742851621
O	-0.47629637743865	-0.90382465881297	1.42122398853188
C	-1.74685640324898	-1.44656830327425	1.35029400535428
C	-1.94092626982224	-2.58443443809503	0.56016004102276
C	-3.26817632877488	-3.14079905094654	0.45584990073050
C	-3.59109948016253	-4.23687571268892	-0.40304617564982
C	-4.88837387828220	-4.72922163651496	-0.48017483771249
C	-5.93401856217746	-4.14917413623113	0.29246827122031
C	-5.66295668006543	-3.06512792256851	1.11543197853643
C	-4.34167128431541	-2.52745139138010	1.20743719513218
C	-4.08568861175819	-1.35319980994564	1.96905993702569
C	-2.81806499277811	-0.78388795813743	2.03412633086711
C	-2.60783247434901	0.54515653138374	2.66439646465485
C	-1.51493732533112	0.81495825171573	3.51073782049052
C	-1.30820293199690	2.11144843414771	4.01252472424786
C	-2.18049526001068	3.15916124329931	3.68038729731643
C	-3.27841023576479	2.88860500834464	2.84493250861296
C	-3.49422458463100	1.59628793341843	2.34490706058422
H	-4.32896201364643	1.40863077616943	1.66241270954598
C	-4.16684520749455	4.02754599383091	2.39046634736188
F	-3.64923707425313	4.63841391049524	1.28845256827108
F	-5.41348671376599	3.61269441450627	2.05920489282333
F	-4.30137114804912	4.98588791449477	3.33961868359629
H	-2.00669678799596	4.17027161587782	4.06018200286496
C	-0.12831282888240	2.34168736549489	4.93416141706855
F	-0.31385579892706	1.73200936032833	6.13523607839898
F	0.09675171333133	3.65239931695674	5.18373809757777
F	1.01612122440328	1.82879281819126	4.41277055086718
H	-0.81444473416138	0.01484607343515	3.76632141089202
H	-4.92273854926420	-0.86833501473675	2.48602638094433
H	-6.46130291177075	-2.58863985329724	1.69905125018787
H	-6.95272267085567	-4.55135024534383	0.22402442247702
H	-5.10917706837432	-5.57071490787876	-1.14923052190888
H	-2.80334510986693	-4.68926864660119	-1.01247392445105
C	-0.81589282726844	-3.08236969980883	-0.28650178422020
C	-0.21596965538113	-2.18865940729122	-1.18101315704873
C	0.73344661822777	-2.60728500900546	-2.17026528915270
C	1.19988252254764	-3.91675383399844	-2.12008135672231
C	0.70942138411215	-4.84420392784095	-1.15713072139971
C	1.21733316675388	-6.17697685245951	-1.09307922179425
C	0.71861716338788	-7.08234557348362	-0.16729319444205
C	-0.29408616989215	-6.67937984882688	0.74771886132389
C	-0.80454998494827	-5.38708265256297	0.71466041850416

C	-0.33366728308363	-4.43790188779327	-0.24154921481641
H	-1.57377656132691	-5.08255126797676	1.43216862011338
H	-0.66883283777679	-7.39313189554589	1.49188986718632
H	1.11484543567258	-8.10406726078073	-0.12604777722101
H	2.01955548584804	-6.46532212117815	-1.78349791529552
H	1.92933732233205	-4.26186057412307	-2.86259185587509
C	1.08054043544744	-1.71039593502098	-3.30325348871104
C	0.04272986071322	-1.01573792714666	-3.96361890646259
C	0.30409228120054	-0.25177633519603	-5.10884150671246
C	1.60957179206708	-0.14767285331456	-5.61322444968629
C	2.64361361831334	-0.83715599913383	-4.95862446293024
C	2.39021474278505	-1.61480882313576	-3.81488108344656
H	3.21010223441537	-2.14435780664436	-3.31814786883649
C	4.05038912055315	-0.68446474771648	-5.49232362008359
F	4.09170162400561	-0.69970146962190	-6.84547853939345
F	4.88520917835560	-1.65124553609749	-5.05147610081043
F	4.58396351748962	0.51351499769263	-5.10548593168505
H	1.81592051597588	0.44694399834506	-6.50838508269449
C	-0.85605074711768	0.34342449048326	-5.87664372850405
F	-0.50836941973687	1.45325608681343	-6.57453667785223
F	-1.35180023722917	-0.54407996303186	-6.77718977672926
F	-1.88917466540125	0.68692781996064	-5.06257765421896
H	-0.98317657061303	-1.09999361057787	-3.59538328825397
O	-0.55606681475387	-0.84978262441074	-1.13833688322031
C	-1.50722514847471	3.00706599799990	-3.10530791010285
N	-1.98369835395664	2.39086210735186	-2.03549245441503
C	-3.34069170400465	2.14917419931833	-1.66335675880883
C	-3.67236315761067	0.83614074640999	-1.26813987244243
C	-4.98086485572610	0.55206461604082	-0.85143203930027
C	-5.94779054589801	1.57390957639645	-0.80753579881705
C	-5.60123644326770	2.88554955478159	-1.17881503314183
C	-4.29728259754080	3.18100097848258	-1.60650020753652
H	-4.01193613001668	4.20563349153693	-1.86577447661281
H	-6.34341385003882	3.69060723513100	-1.11729834408786
H	-6.96714369382047	1.35098455251912	-0.46869545379971
H	-5.23777983602138	-0.46940113430564	-0.54856184058264
H	-2.90397903039635	0.05635415806499	-1.28604002157487
H	-1.33653726032259	1.95952037232303	-1.31872421773402
C	-0.05932609559005	3.14705607793212	-3.27654182533825
C	0.86221767833882	2.86992498155513	-2.21929373457393
C	2.23362940274084	2.93363796374633	-2.41819525314106
C	2.76244408976626	3.27966795579595	-3.69002738127610
C	1.87002610485346	3.60293636411729	-4.74146340774223
C	0.49155524466273	3.54472023340840	-4.52598617130555
H	-0.15771338597021	3.79259836347293	-5.36846562276233
H	2.24450788610427	3.89108652224180	-5.72782895213279
H	2.92584024792438	2.72196752964823	-1.59669897456359
H	0.50669934516385	2.61488527068192	-1.21768521559293
C	-2.45862964514011	3.54014167887662	-4.13948020484710
H	-2.08447038231127	3.33947103697724	-5.15426500468963
H	-2.54793269327206	4.63781664979894	-4.02373646465838
H	-3.45907838846723	3.09253858778851	-4.03991141007955
C	4.70750233281699	3.64453595362593	-5.04534916231077
H	4.46071362443346	4.69152276965186	-5.30905581482421
H	5.79400021212035	3.53490847766474	-4.89726540822558
H	4.36739436895516	2.96735698889654	-5.85039551588706
O	4.10144394793196	3.28599392533149	-3.79353597524973
H	2.87380635809736	-1.04635914030737	-0.02157795715600
C	3.52704630147538	-2.98793128050398	0.27802133315171
C	4.57005929757575	-1.31342878783259	-1.18184131055834
C	4.40524671748124	-3.96586954866501	-0.13714795891608
C	5.53093121993510	-2.20230647928798	-1.60873519690087
C	5.61030482196438	-3.61442951264455	-1.01713573261542
N	3.63781762494125	-1.71127420237785	-0.23986074539349
H	5.68795167531087	-4.34781710383881	-1.83992025605144
C	4.33851521650615	-5.38934198829869	0.23592597628668
O	4.89812789020220	-6.28237094423685	-0.40002390537189

O	3.63696573290798	-5.65254791148199	1.37078364055364
C	6.62873174551947	-1.91203299491687	-2.55417103283769
O	7.26562230518913	-2.79121939937529	-3.12983759602765
O	6.94922834044833	-0.59217287434725	-2.66978044782550
C	8.12141805173497	-0.24858024780180	-3.45295990845575
C	7.83791465212140	-0.16378739376424	-4.94950786066004
H	8.91526591177054	-0.99079585279188	-3.24714005787942
H	8.43405577039860	0.73280186077735	-3.05340083907143
H	8.76584986079731	0.13707499051910	-5.47356774710830
H	7.51184037803627	-1.14257281949725	-5.34159580489756
H	7.05366657013172	0.58285171743553	-5.16376589923863
C	3.55433428128766	-7.03381943402829	1.79067855348930
C	2.67376164061376	-7.08841620720393	3.02864050761085
H	4.57846525156982	-7.40459501476155	1.99348021179837
H	3.13759073628503	-7.63757372863897	0.96361177216136
H	2.60552330284940	-8.13307761778493	3.38663651388529
H	3.09576791126569	-6.46597300510856	3.84051467563889
H	1.65395049802098	-6.72930000521966	2.79701563278289
C	2.41250113879908	-3.14435236389441	1.28372188964723
H	1.85181256092919	-4.07446070569683	1.12128080916604
H	2.84147342110382	-3.20442433833397	2.30234989667415
H	1.72046980990799	-2.28831941815415	1.24138241133681
C	4.37736514695150	0.10977049694370	-1.64633838079724
H	4.93368697663027	0.80377970865443	-0.98514225828540
H	4.74546903061019	0.26153334125685	-2.66775092133610
H	3.30888123633751	0.37750237260039	-1.59106677823371
C	6.88980532250806	-3.75550885949585	-0.17742323045879
C	7.83084569701726	-4.77027195300309	-0.43575215824997
C	7.13027618837191	-2.86044793381804	0.88627890646171
C	8.99080849124214	-4.89110031009847	0.35041081229863
H	7.64538605887280	-5.46676898429423	-1.26267171425248
C	8.28759474387394	-2.97489708114889	1.67556964393440
H	6.39983758647884	-2.06624558276281	1.08958690531504
C	9.22317531182080	-3.99303968583264	1.40906268939580
H	9.71689052965449	-5.68669035561429	0.13554425389482
H	8.46135831529480	-2.26960500561672	2.49932101441426
H	10.12882158328919	-4.08561628791028	2.02302722598353

7.2. **CPA 1a, *Z*-imine 2a, HE 3b**

7.2.1. Conformer C5.

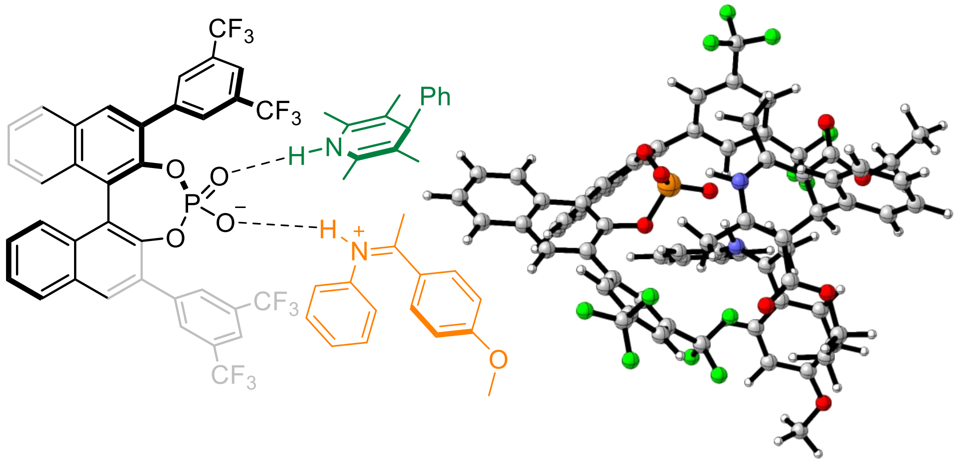

G180K:-5019.33060315866

P	1.11282395051832	0.19992606939286	-1.44024112459853
O	-0.36564150842368	0.28280264853159	-1.10770080978537
O	1.72287015208614	-0.96132731266555	-2.18522460873346
O	1.88331493328381	0.47888416191582	0.03902001293094
C	3.23835847616193	0.72654509252278	0.04568641592764
C	3.71416504840504	1.96149263927449	-0.41696480729071
C	5.13639526093216	2.14287243248443	-0.55048586042838
C	5.70875409069232	3.31110740239981	-1.14004001088128
C	7.08617777146903	3.44649690430782	-1.26431723750685
C	7.96051609109285	2.42195459526246	-0.80164869100137
C	7.43696476581672	1.26759132795234	-0.23510844375385
C	6.02443410090484	1.09200971671787	-0.10391290701388
C	5.47955674125533	-0.09268008345588	0.47047096877323
C	4.10483776062526	-0.29568612585888	0.56055324607135
C	3.54071274461205	-1.50773344097688	1.21280523168644
C	2.42909688449070	-1.40562895777290	2.07722877072170
C	1.89419675680844	-2.54525823965213	2.69598587240115
C	2.46692737068474	-3.80932027412402	2.49275679672938
C	3.58705730314192	-3.91359179777238	1.65058833105183
C	4.11505272449607	-2.78073623091576	1.00945074300701
H	4.97032721303856	-2.88584671954269	0.33389517475972
C	4.14949301694096	-5.28764437324717	1.35112485350011
F	5.43232781689984	-5.24174829224569	0.92185977815940
F	4.11491138559944	-6.09888367604964	2.43632444484064
F	3.42951146042699	-5.90891764351752	0.37566256621685
H	2.03373675143139	-4.69927790763496	2.95768399818274
C	0.61453098481747	-2.38327566925593	3.48688379392166
F	0.30896547087360	-3.46807547649783	4.23209996319094
F	-0.44102105706897	-2.16695049261520	2.64560227630787
F	0.65765075109860	-1.31453176604055	4.32187361810383
H	1.97392467190958	-0.43062542382932	2.26709503050506
H	6.16727278832051	-0.85220681734664	0.86251452716593
H	8.09809978241521	0.46484503348306	0.11649045201866
H	9.04641082445844	2.54460577260691	-0.90174177105553
H	7.50406368023398	4.34972893440270	-1.72700963948395
H	5.04370652899840	4.10188467841700	-1.50332091770138
C	2.72344179920642	3.04182379503290	-0.70518445161522
C	1.65179620344030	2.78022238979821	-1.56650325106061
C	0.55375386703678	3.68556192045329	-1.72732527811155
C	0.60315081508357	4.91031487661303	-1.07500688051875
C	1.68721397609410	5.25260235071629	-0.21897472751142

C	1.69038675023272	6.49301517043264	0.48897048343260
C	2.67224668289717	6.77488960690611	1.42768482914140
C	3.68630162801563	5.81326769646213	1.69733652707673
C	3.72249379578989	4.60656087929985	1.00861186045264
C	2.74508773707087	4.29400313373037	0.01290804937199
H	4.50342396932003	3.87711937312704	1.24092887039142
H	4.44517560110168	6.02269347000605	2.46199597589713
H	2.65877322511697	7.72538534020835	1.97582006617270
H	0.87930202791867	7.20597643107883	0.29278923968715
H	-0.22857821909501	5.61722213546752	-1.18530476986552
C	-0.66599294280438	3.24179553548702	-2.45417065116354
C	-0.62711061972235	2.81747636638109	-3.79500032312855
C	-1.78673783419709	2.31796531739019	-4.41154121764954
C	-2.99122066247403	2.21125711040321	-3.69889648988508
C	-3.02655616531419	2.63080470624182	-2.35938330945560
C	-1.87946625135907	3.15597265896165	-1.74377878143033
H	-1.90668601171995	3.45290065069429	-0.69012036057495
C	-4.26717842151468	2.39059116304439	-1.52793827211761
F	-5.39846422724394	2.35730603360955	-2.27192256012450
F	-4.43980802195738	3.33546829127759	-0.57150066582233
F	-4.19268822519971	1.19147977130574	-0.87638414150702
H	-3.87432658041266	1.76825527299357	-4.16426251491859
C	-1.70918530207244	1.88904509088416	-5.86186707495847
F	-2.75442758840898	1.11475385890859	-6.23608207575113
F	-1.68900870563472	2.96281903316690	-6.69539174840101
F	-0.57731710959413	1.18088429973498	-6.11431248096563
H	0.31503294115549	2.85916034888998	-4.35019674610115
O	1.62230574970147	1.58256954355047	-2.25186741923961
C	-2.39540053116420	0.00405159592196	1.64819860546827
N	-1.56824747374053	0.92427907147201	1.19260542555389
C	-1.29624796639731	2.21059588880648	1.74953570658253
C	-2.35952968525019	3.04436330568555	2.14956543682587
C	-2.07548013083655	4.31680578450641	2.66519548360061
C	-0.74163997727714	4.75376141235277	2.78169690659369
C	0.30940022978430	3.92052028334619	2.36155012317572
C	0.03769249037023	2.64871900021908	1.83216985388686
H	0.84344141446619	1.99588411264979	1.48486089079676
H	1.34663312646963	4.26467514627666	2.43237550019366
H	-0.52244093554478	5.75003710023748	3.18520346212906
H	-2.90048355788349	4.97269470717743	2.97023013781630
H	-3.39141147612405	2.69546720659303	2.04134473311192
H	-1.07293269348714	0.70825104527725	0.27238593962321
C	-2.95550691730221	0.01138725689961	2.99864036350588
C	-2.21358531958356	0.49200803238990	4.10788700627963
C	-2.70575622619234	0.38567696839301	5.40918621183850
C	-3.98287641633591	-0.18971385624954	5.63511650786637
C	-4.73426357724172	-0.67661192423579	4.53401978301794
C	-4.21802911244039	-0.59496183850623	3.24440597011700
H	-4.80591621279159	-0.98788500117251	2.40794517152076
H	-5.71629889152099	-1.12526095680107	4.72256481756752
H	-2.09282238111553	0.73735360370180	6.24417882278444
H	-1.21511652684192	0.91252520443540	3.95743009297144
C	-2.74013811669930	-1.12233621371093	0.72875901008144
H	-2.09207808788753	-1.14025233116969	-0.15889337432510
H	-3.78460207458427	-0.99317166496078	0.38673362675714
H	-2.69254131365435	-2.08481491395441	1.26071157460733
C	-3.84136821353111	0.13024643682372	8.00852366306203
H	-2.88133788363142	-0.41188146845684	8.11747362178049
H	-3.64268926184425	1.21830194384328	7.94509103552010
H	-4.49538897715854	-0.08420364697342	8.86948824766609
O	-4.55161806978646	-0.32501089896600	6.84808300311091
H	0.40691786497960	-2.32692204605422	-2.55393345244557
C	-1.61172709760173	-2.14785905802941	-2.84112474855369
C	-0.48974997420071	-3.54283553998527	-1.17525512285645
C	-2.85315795339538	-2.45165234152387	-2.32463337115049
C	-1.68924603253323	-3.92787471457150	-0.61503704067169
C	-3.00764311084977	-3.60179907646586	-1.32402444550846

H	-3.74124029879046	-3.30255453441881	-0.56351730001072
N	-0.49195497667217	-2.77609015464068	-2.32646213176032
C	-1.72710106292525	-4.65276360846292	0.66461173459469
O	-0.77176185196123	-5.11249498935883	1.28683544050588
O	-3.01333440001486	-4.76175133002050	1.12722957352968
C	-4.04396719506559	-1.68933186715826	-2.72780326604993
O	-4.12302869871366	-0.86529608975917	-3.63730985832623
O	-5.11629153688452	-1.98367030201530	-1.92776857374548
C	0.88129032622242	-3.83607125775908	-0.62310892596210
H	1.03557459701192	-4.91991771328139	-0.49638742232938
H	1.66763051603338	-3.41396801581937	-1.26954547990843
H	0.97812937952412	-3.39486482204430	0.38154794534358
C	-1.31912057090548	-1.12248028842491	-3.90431906127105
H	-1.57480676868824	-0.12050338141447	-3.52654138340108
H	-0.24931810603704	-1.12746507318995	-4.17247124055390
H	-1.93543073234277	-1.29385253043199	-4.80150637670838
C	-3.24085302513077	-5.39163364279429	2.40858821921756
H	-4.25207328396575	-5.82992272541072	2.32822466405674
H	-2.50529842000644	-6.20419009046293	2.55391688592697
C	-6.37502171090808	-1.32799932740096	-2.20940992346960
H	-6.18866061490430	-0.26872751123010	-2.46031449305122
H	-6.93055158975756	-1.37914653207423	-1.25601858900402
C	-3.16927316359196	-4.37540954348605	3.54553370405448
H	-2.15127786479366	-3.96141644853795	3.63769528656464
H	-3.87624673328849	-3.54215116356022	3.37320265027214
H	-3.43617574330785	-4.86900181202599	4.50007381626645
C	-7.13261828448984	-2.03633774288323	-3.32929323030837
H	-8.11993803366949	-1.55593548870010	-3.47159853438441
H	-6.56987945561020	-1.96977614938965	-4.27806034565215
H	-7.29239926278574	-3.10270875352094	-3.08051750590246
C	-3.57950199472920	-4.83949096805459	-2.02194850612760
C	-2.84738822794691	-5.47715006420606	-3.04614687730870
C	-4.84150215923288	-5.35613603526111	-1.66825087793051
C	-3.36492288162152	-6.60523298788938	-3.70451681630530
H	-1.86327457327499	-5.07931049274012	-3.32578227626846
C	-5.36551683948359	-6.48553240213787	-2.32394020238153
H	-5.41311532500199	-4.86361947621723	-0.87149128888771
C	-4.62872661503548	-7.11319913476655	-3.34520124433428
H	-2.78306860748629	-7.08996928455866	-4.49983469218972
H	-6.35093271104317	-6.87662038082103	-2.03709082662428
H	-5.03603881826970	-7.99407580980357	-3.85873959676305

7.2.2. Conformer C6

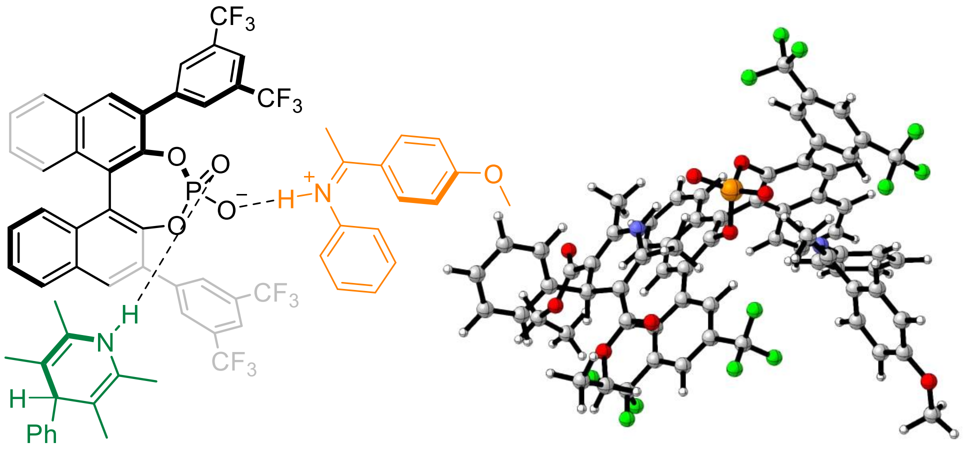

G_{180K}:-5019.32367830154

P	1.03509799514359	0.81372385935722	-2.58533881923322
O	-0.33201424003467	0.61663290048592	-1.96242378510761
O	1.45147585500056	0.04567928435247	-3.81629770219926
O	2.10103377545380	0.63265267718210	-1.27713823003537
C	3.41214661279162	1.04835532633159	-1.27374509950595
C	3.69802552329491	2.41254884967275	-1.47943294952810
C	5.05343284200828	2.80057463591406	-1.75354223444005
C	5.40544981793219	4.11349426316935	-2.18951427721631
C	6.72705020952389	4.43441328891399	-2.47521535028077
C	7.75956243656190	3.46386835148364	-2.32287580944506
C	7.44943813317584	2.18220803461215	-1.88926582767525
C	6.09755595630060	1.81982571682836	-1.59919822434128
C	5.76584863971416	0.52752793087859	-1.11392118523730
C	4.44858901127084	0.09656059013316	-0.93016909960301
C	4.18068512235607	-1.21495296314508	-0.25781921515792
C	2.91741411649151	-1.54043374938187	0.29321329344171
C	2.72641518074474	-2.70858257134008	1.04543434121857
C	3.78848617975622	-3.58080244647459	1.31395830023751
C	5.04628605308899	-3.27720792316107	0.77343513069110
C	5.23323441959276	-2.13567802023828	-0.01973157759959
H	6.22772486075294	-1.97184229769199	-0.43843503459649
C	6.25412767680434	-4.11666266971098	1.14114150264082
F	7.02836988520175	-4.43922568985034	0.07490489263056
F	7.06402353355450	-3.43349811750641	1.99898407415375
F	5.92038877885467	-5.27657704118683	1.75581846960896
H	3.64032969853070	-4.47472435618632	1.92466499305417
C	1.36917262173618	-2.95832749201951	1.66627675056400
F	1.16956899456197	-4.25404516726393	1.99110017159121
F	0.34674118769471	-2.58850005891195	0.84327483228898
F	1.21340303328699	-2.23400140837460	2.81087166965083
H	2.07035987512354	-0.87359215224783	0.14197959175549
H	6.60501037415465	-0.12892828915062	-0.87263059935451
H	8.23226715959208	1.42288494379359	-1.76853608714546
H	8.79846687334099	3.73290649644399	-2.55179724824713
H	6.97823890460518	5.44339465230760	-2.82654162505011
H	4.61418858489041	4.86178348033281	-2.31158184537915
C	2.62253108654838	3.42696704198989	-1.25597535853808
C	1.40710928263027	3.32855252132548	-1.93289118823969
C	0.24775497526370	4.09272194331720	-1.57515433590976
C	0.39467172582201	5.07633502270712	-0.60374790912986
C	1.62802110724018	5.27861342819458	0.07479214185077
C	1.73217549720566	6.26196469535395	1.10556540170915
C	2.88262354764840	6.37144778334533	1.87249693123944
C	3.96998463323845	5.48419668224779	1.63895377202682

C	3.90638223585377	4.53042615647231	0.62977468681266
C	2.75152355577121	4.41129080513562	-0.20440487907891
H	4.75061721648244	3.85180965926176	0.48415467810752
H	4.86805170048741	5.54660327276749	2.26663637766372
H	2.94899333727273	7.12272403530520	2.66951074052684
H	0.86741235566799	6.91055399759642	1.29532465841832
H	-0.47109348422850	5.68428037485512	-0.31433442957649
C	-1.09314794278320	3.71278318461441	-2.09044255024445
C	-1.33833346471230	3.43203051319303	-3.44831419930487
C	-2.58824773909423	2.93681480536086	-3.85660087496233
C	-3.61681892391883	2.72054903986129	-2.92756992419391
C	-3.38333117172906	3.02746277445515	-1.57562202678152
C	-2.13823317283490	3.52210937389269	-1.16060103579343
H	-1.95535645437758	3.71664108323041	-0.09941313827681
C	-4.44709821457658	2.72103556979884	-0.54338401958147
F	-5.69939925220910	2.82252315914800	-1.04324479355311
F	-4.37142608687496	3.53710484435724	0.53518217715491
F	-4.32046812044969	1.44298320369839	-0.06893125299123
H	-4.58247359601440	2.31855197329336	-3.24758611451610
C	-2.77016981192475	2.59906375913384	-5.32161005644094
F	-4.02246682247335	2.18156320994636	-5.61682011522189
F	-2.50351500248376	3.66761306323095	-6.11628810339208
F	-1.91704118368218	1.61090728701975	-5.70220477831643
H	-0.54446370100573	3.57700975051781	-4.18616063685077
O	1.31661753726583	2.41284309299108	-2.96268665988545
C	-1.98163840615573	-0.46720643946336	0.80619400561814
N	-1.14874037150600	0.52550159962414	0.56879916187254
C	-0.75424126053340	1.58133879686246	1.44611241542436
C	-1.71285223747025	2.26636408383590	2.21918791761781
C	-1.30211183045699	3.33039214512176	3.03411932280606
C	0.05363823187376	3.70842010990955	3.08048386737671
C	0.99817771387204	3.02875863710519	2.29284776727924
C	0.59784087416489	1.96842003523319	1.46444924250521
H	1.31945893515748	1.44008296140108	0.83540624354781
H	2.04984725358891	3.33471046811194	2.30688970397598
H	0.37084392070024	4.54403797729864	3.71598012244937
H	-2.04794530815900	3.87245467873016	3.62877452366668
H	-2.76494094875800	1.97211471452477	2.16902008454020
H	-0.76184464518562	0.59432661924207	-0.42440133553214
C	-2.45146790314779	-0.82129624001095	2.14534063225933
C	-1.60552664709198	-0.71200240221303	3.27840558687608
C	-2.03148133902213	-1.12073428368184	4.54257455950173
C	-3.34394424251374	-1.63268181300387	4.71219201105701
C	-4.19748509667009	-1.75376149289056	3.58465850461910
C	-3.74933771307285	-1.37374626194344	2.32296161894790
H	-4.42234050741765	-1.47396333476187	1.46460948553869
H	-5.20745094336910	-2.15448727917808	3.72884265067980
H	-1.34188072176740	-1.04970094213885	5.38878162680600
H	-0.58349560516716	-0.34165989015730	3.16151787046675
C	-2.44466637386737	-1.27720216179530	-0.36946183096535
H	-1.88172556980708	-1.02227517826416	-1.28070128206792
H	-3.51572089325183	-1.05506975084213	-0.54280672063165
H	-2.36144795398977	-2.35414085634783	-0.14176406180269
C	-3.05217953812004	-1.94401847892123	7.07414893591691
H	-2.14365743807811	-2.57111067341508	6.97913830448981
H	-2.75466082556917	-0.89456202693837	7.26758608967176
H	-3.68134651306896	-2.31569834274883	7.89938255459608
O	-3.85781514129295	-2.03382207841032	5.89039557510583
H	2.54460541872015	-1.27538230900192	-3.73519685334219
C	2.76552348298237	-3.08205685355729	-2.76211821964668
C	4.56617729428129	-1.69184007905852	-3.66633559648177
C	3.65708699152542	-4.02090233627321	-2.28996873354399
C	5.53181501447382	-2.57223065170593	-3.22311133284055
C	5.12832983313095	-3.96121498596666	-2.71245963826824
H	5.74576851252060	-4.20084341799290	-1.84029981378311
N	3.23563791753560	-2.01077105559967	-3.49452443691248
C	6.95508226194255	-2.20897595838537	-3.25771026845787

O	7.45750074573749	-1.21095314918506	-3.77340462770627
O	7.71418551576894	-3.13661305810137	-2.59593969768288
C	3.20783603490525	-5.11437903585373	-1.42003740026740
O	2.05361369825447	-5.35951221017230	-1.07047084061272
O	4.26346488208514	-5.87887727269425	-0.99910538673576
C	4.80739669611352	-0.35818564254108	-4.33149753883137
H	5.30678822666216	-0.50495063933010	-5.30717293959537
H	3.85613670403173	0.17976188009102	-4.47965126051976
H	5.48781995057295	0.25913663565138	-3.72440740850740
C	1.27283038389270	-3.07746455101642	-2.53716957668878
H	1.04745081550756	-2.92256606088512	-1.46889237346044
H	0.79403993654376	-2.27448562041209	-3.12162348561457
H	0.83849497575923	-4.05449514841111	-2.80768684846064
C	9.11575622138710	-2.85534254864418	-2.39564769965010
H	9.57845948641780	-3.84564599674569	-2.23591247476120
H	9.53593542182881	-2.40275245521058	-3.31317744061215
C	3.97867849573637	-7.02932699799280	-0.17315296232476
H	3.19405283792599	-6.76616638566489	0.56067666070183
H	4.92216212415031	-7.22337402697778	0.36412090546862
C	9.33094120266733	-1.94429910942029	-1.18842006329607
H	8.86160120207089	-0.95859231984760	-1.36070897569184
H	8.89506671996721	-2.39437511560339	-0.27758948236666
H	10.41509261068203	-1.79203159103704	-1.02429523305958
C	3.56020890860229	-8.22869305172407	-1.01981208302981
H	3.42006258125804	-9.11355538011428	-0.36928432150040
H	2.60788557624026	-8.02054837854383	-1.54060453440842
H	4.33671880902052	-8.46417111392211	-1.77236039156017
C	5.43178672201416	-5.02890585618726	-3.76837468694784
C	4.77114974198901	-5.00759282764669	-5.01465212806600
C	6.36975224030088	-6.04922042860925	-3.51503487312261
C	5.03972651598364	-5.98577917292364	-5.98755150582844
H	4.03861037940103	-4.21560376267360	-5.21870027797969
C	6.64293859284983	-7.03129767727492	-4.48410869967824
H	6.88224731745331	-6.06757891694755	-2.54543342662959
C	5.97763998637348	-7.00282035346624	-5.72423365651473
H	4.51684447752956	-5.95641442895537	-6.95291318836357
H	7.37579953416550	-7.82130993861272	-4.27152770137223
H	6.18867771430928	-7.76891052069623	-6.48200000753794

7.3. CPA 1b, *E*-imine 2a, HE 3b

7.3.1. Conformer 7

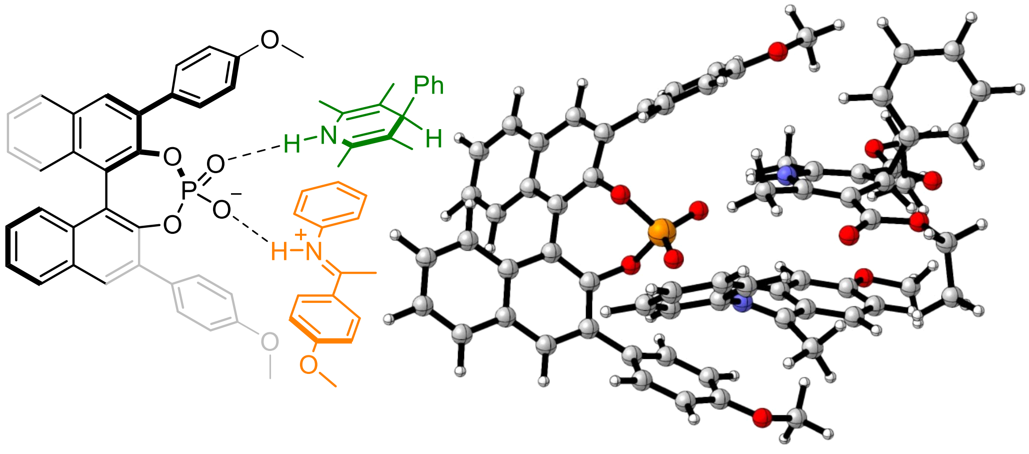

G_{180K}:-3901.53223398991

P	-1.51228575435878	-0.13832677272377	-0.03199270822900
O	-0.56741322916784	-1.21849654433216	0.43328737395504
O	-1.02019097571144	1.18107616251528	-0.59943562932781
O	-2.57972965143491	0.12450528769923	1.23745217460762
C	-3.69234309322790	0.89307613906995	0.94983086408872
C	-4.77414232984310	0.27678168835685	0.30347345973474
C	-5.90948038073646	1.08487717228203	-0.06387198278994
C	-7.00999162398747	0.58030393748599	-0.82362869816360
C	-8.08638244924741	1.39449991170345	-1.15512187694280
C	-8.11629287664453	2.75825321487567	-0.74610331434145
C	-7.04985565826656	3.28980861577130	-0.03371887160022
C	-5.92007374132039	2.48290185306846	0.30984184622816
C	-4.79067633876431	3.04636533127192	0.97031191907437
C	-3.65746929360034	2.29332055001913	1.27365327286790
C	-2.40767002098215	2.92848460773153	1.77181601101581
C	-1.57504383890576	2.33160282194914	2.74452184395734
C	-0.32135170903996	2.87003928945636	3.07222724791225
C	0.14075242949063	4.02927394150768	2.41053350997146
C	-0.70696154857821	4.67762903049876	1.48387295043028
C	-1.96003424281002	4.13714118619838	1.18235602455692
H	-2.58547548648592	4.62890181493586	0.42760199052977
H	-1.89804657765262	1.41243316081219	3.24248192334384
H	-4.80720450666623	4.11597028997857	1.21449958838826
H	-7.04692081554553	4.34571691872202	0.26750102474632
H	-8.97550954411676	3.38897822252411	-1.00807098683763
H	-8.91855091817930	0.98356919782721	-1.74118185652660
H	-6.99601451355243	-0.46480826138377	-1.14909094777709
C	-4.67099939890660	-1.17827305653506	-0.03217187789100
C	-3.54890719089571	-1.63230221552671	-0.74310902881209
C	-3.29389536426377	-3.02185394438338	-1.00707402671417
C	-4.27112369233070	-3.93591127633881	-0.61470168878103
C	-5.44298420032255	-3.53813370515230	0.08963510609875
C	-6.40841723766015	-4.50358334671080	0.51558797549863
C	-7.50534539502321	-4.12728871547723	1.27850453912372
C	-7.66915067018677	-2.76484758145367	1.65899111398154
C	-6.75646264774067	-1.80070525708784	1.24793597684790
C	-5.63592564468021	-2.14494393587970	0.42947200916770
H	-6.89141215261671	-0.75899827075667	1.55506751387162
H	-8.52097283731370	-2.47311954132077	2.28676977600713
H	-8.23717114026133	-4.87802558594837	1.60331395189158
H	-6.25243023301359	-5.55420135273872	0.23712846543444

H	-4.12226980199342	-5.00322007555446	-0.82115494047780
C	-1.98203772431190	-3.47905037085623	-1.54162234350687
C	-1.30680378153740	-2.83344715687948	-2.60847786171474
C	-0.02387913398571	-3.22771929706138	-2.99733571029901
C	0.63845761413365	-4.28146867217876	-2.32469765436405
C	-0.03245450816176	-4.96347034848999	-1.28757974032653
C	-1.32340846622369	-4.55895831649631	-0.91226227193310
H	-1.81181506461826	-5.06909087867836	-0.07265054371232
H	-1.78919889069415	-2.00125392374117	-3.13039895666651
O	-2.61803775875761	-0.70167196578193	-1.16433650114269
C	2.23014775586882	3.03769942211615	-0.25701401261987
N	1.07665875183010	2.87314301688857	-0.88858866863427
C	0.61369276608153	3.56792653861912	-2.04414336710833
C	1.47951909654440	3.84433673615468	-3.12384827479271
C	0.97317850555062	4.48129430807002	-4.26766203756467
C	-0.38837314790219	4.82742213852357	-4.34637440165642
C	-1.25201438587471	4.51540165151263	-3.27945388094612
C	-0.75867309470477	3.87972380109317	-2.13054290325167
H	-1.41986858069328	3.60903603298097	-1.30399061055150
H	-2.31886556748649	4.76441175539281	-3.34161889383635
H	-0.77876440025114	5.32365049981601	-5.24378802344807
H	1.64645711578412	4.69341007114867	-5.10763460451501
H	2.52516154724918	3.52694021562727	-3.07745544900971
H	0.39851106224730	2.11890114704155	-0.60485875130685
C	2.67828902757139	2.07140610894391	0.73620849547225
C	1.83341943630191	1.05822425303675	1.26997806603636
C	2.31275708576538	0.12067168168512	2.17228120386783
C	3.67284108664506	0.13903908531360	2.57194701897683
C	4.53715706881517	1.12909587688817	2.04201097158572
C	4.03518264416565	2.08088403519595	1.15588021205388
H	4.73114024700515	2.82193095831792	0.75622182357272
H	5.59661525612283	1.15428062261887	2.31100068660129
H	1.64837627108720	-0.64856605809653	2.57775387932337
H	0.78284929259155	1.00359572568061	0.98750719440274
C	3.08709755167199	4.23482195841174	-0.56048831193547
H	3.91066329347696	3.94292187982264	-1.23683722473334
H	3.52657795697573	4.61288403025370	0.37668482440003
H	2.50317538674592	5.03506446959380	-1.04131958964426
C	5.39310956919845	-0.78352550480790	3.96234338154825
H	6.11138089609784	-0.99782303917271	3.15020268655958
H	5.61830687868330	0.19627291430039	4.42679636346622
H	5.43920306853890	-1.58038900834293	4.72102217574797
O	4.04877341779738	-0.80304513732249	3.45521968518862
H	0.30066187985507	2.36347111625998	3.81576714399883
H	-0.34824229733334	5.58351807835833	0.98077094363360
H	0.45290216951694	-5.78143043601486	-0.74687033403527
H	0.49684067746800	-2.71542919739115	-3.81514597735636
O	1.37659026366772	4.57509180645243	2.58296260693565
O	1.90725552979072	-4.55986552708180	-2.73490932015196
C	2.24762538142462	3.99567697203051	3.55722371765481
H	2.45219181847483	2.93270355123319	3.32723390281250
H	3.18780499239768	4.57009086186933	3.51141638832525
H	1.80731410554307	4.07012372312790	4.57253593745996
C	2.63807117066199	-5.58276891823584	-2.05427278774115
H	3.62130810801167	-5.63773454148293	-2.54848842346579
H	2.77844540734809	-5.32742370526181	-0.98518595715777
H	2.11961356092247	-6.55994610960972	-2.13115245952598
H	1.06875806249731	-1.63769757871782	-0.50544472112549
C	2.76109641341701	-2.47220925388101	0.31624833840831
C	2.68052145664379	-0.66228190991675	-1.33186232992650
C	4.13397911619837	-2.40318353455616	0.39224097854368
C	4.03687049686990	-0.43090666273301	-1.22048959155255
C	4.92582750534339	-1.46697004447355	-0.52450426243579
N	2.09515267164239	-1.68060793941184	-0.60203159321223
C	4.97597495694668	-3.20648303753345	1.29391740271372
O	6.15052867474186	-2.93861648080996	1.54845495542161
O	4.36861032610776	-4.32259159339183	1.78553819874641

C	4.68968747952804	0.75122149513689	-1.80617230953855
O	4.16614772716652	1.69796668115027	-2.39820694370550
O	6.03740147499596	0.71410665602213	-1.58498646256082
C	5.12604353268761	-5.18007087752143	2.67361848506839
C	5.13887008497082	-4.65333992477869	4.10550468594591
H	6.15509176370092	-5.28846547591168	2.28291982327254
H	4.61143668684284	-6.15531081204921	2.61209899430261
H	5.62467690524232	-5.39590315865096	4.76740096849472
H	5.70446990503096	-3.70760496014554	4.16498999890856
H	4.10800635399154	-4.47979004012260	4.46865272388261
C	6.86566506167609	1.78404805537565	-2.09109037694052
C	6.99137221246420	2.92760727057985	-1.08820311802660
H	6.45736529626817	2.14239838085549	-3.05407744660889
H	7.84702195792652	1.30850967078863	-2.26936582588005
H	7.75174189252417	3.64872287095059	-1.44443715933626
H	6.03313847811037	3.46444727381742	-0.97568477387933
H	7.30562132211102	2.54521571502770	-0.09863021542285
C	1.85975055527592	-3.31895578043257	1.18355889010425
H	2.18390748382971	-3.26419925793265	2.23548790926452
H	0.81585400152826	-2.98058892974653	1.10022496775155
H	1.91382042375683	-4.37962432311837	0.88168318768376
C	1.71940243501319	0.03580985274921	-2.25406365780410
H	0.75072702538177	0.21193658884986	-1.76038162785704
H	2.12878746703811	0.98128061402178	-2.62627504080462
H	1.52865650793414	-0.63742766265315	-3.11394346690149
H	5.67653439306310	-0.94823812671055	0.09420552290865
C	5.68563954744901	-2.28893253598397	-1.57477142066707
C	7.09226348218578	-2.35739752838661	-1.58207284749958
C	4.96544054384363	-3.00597772815778	-2.55289519192015
C	7.76897469797164	-3.12555825145449	-2.54670015392446
H	7.65468287423608	-1.80196862967946	-0.82139251183210
C	5.63593064474186	-3.77458199280143	-3.51988284832004
H	3.86961435871962	-2.96742048526351	-2.55376443122500
C	7.04261733802217	-3.83687476168049	-3.52041807302643
H	8.86649286620372	-3.16882818307691	-2.53979123883860
H	5.05792054822199	-4.32561284100350	-4.27384943860285
H	7.56971983321175	-4.43515894569270	-4.27534391202258

7.3.2. Conformer C8

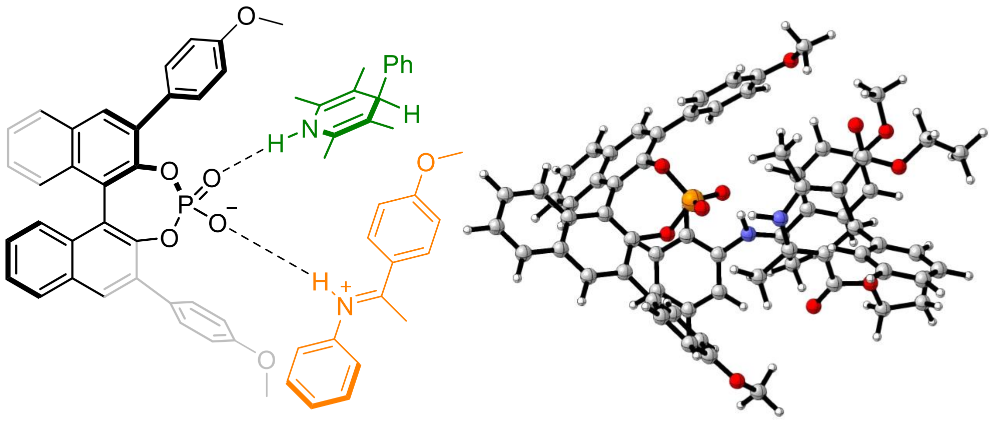

G_{180K}:-3901.52829295405

P	1.26359437089251	-0.32778268913814	1.12690867797510
O	0.18669816294397	-0.68992165685803	0.12188391239118
O	0.91772750794331	0.27505382690270	2.47260812628408
O	2.36430840722248	0.63111748487195	0.29370349543072
C	3.64303058068730	0.77814842685765	0.78931062216045
C	4.53382932931129	-0.30138767574214	0.71235469057986
C	5.82026019512855	-0.18574003530398	1.34791718283491
C	6.74751955706711	-1.27123709259123	1.41498383943183
C	7.97357355131323	-1.12679212076316	2.05326877611886
C	8.33483693094522	0.11204890832416	2.65591732684912
C	7.45152138248607	1.18302882589086	2.62106144229971
C	6.17849660081251	1.06431329797617	1.98151162125432
C	5.26334904932376	2.15635536375159	1.94677368200936
C	4.00671550397743	2.04579287650428	1.35698204676746
C	3.07605720334225	3.20134726936106	1.28094019869686
C	2.77831860637403	3.99284552638455	2.41521271593763
C	1.88713470907361	5.06928335850456	2.32924283594865
C	1.27559689896856	5.39220883665732	1.09495998282134
C	1.59832022404473	4.63798710838162	-0.05452724243796
C	2.47487870351042	3.55123624999273	0.05275755972111
H	2.68790142652314	2.95731309439855	-0.84101496556143
H	3.23015549785327	3.74020155689387	3.38298333957174
H	5.56809021819416	3.11432820405797	2.38709492020620
H	7.71273028419574	2.14081294211272	3.09018185125151
H	9.30797419909785	0.21447468295870	3.15309612610888
H	8.66779587312929	-1.97593315349352	2.09678297434838
H	6.47641780015860	-2.22940702224862	0.95874637521737
C	4.10948573962989	-1.52932010457377	-0.02448724592268
C	2.92442478055453	-2.17161575052983	0.35039243623823
C	2.36760038045087	-3.28052398612702	-0.37361236896937
C	3.09053143530951	-3.74761515985318	-1.47212057385488
C	4.29808794750613	-3.13278849804031	-1.90777507718424
C	4.98311512582143	-3.59795519287765	-3.07387306061064
C	6.11574796055349	-2.94649544192704	-3.54261882216737
C	6.60377192079297	-1.79436686606353	-2.86212845818771
C	5.97027772307161	-1.32825075545954	-1.71628547000503
C	4.81608202847977	-1.98873198363044	-1.19209760970177
H	6.34819385447807	-0.43562289506721	-1.20856823821473
H	7.48528846387214	-1.26664359038677	-3.24823669519221
H	6.62871861747988	-3.30782260320262	-4.44310063966917
H	4.58141514660432	-4.47435595757873	-3.59931959491451
H	2.70808077131913	-4.60353535945752	-2.04154136493874
C	1.03092206308879	-3.84249377791836	-0.04606066597733
C	0.57855427305705	-4.03280974548168	1.27866874003072
C	-0.71450903914658	-4.50472264902846	1.55547029428675

C	-1.59641639252255	-4.80414472654605	0.49416250967289
C	-1.15262331281219	-4.64445508463861	-0.83864164368596
C	0.13694130064162	-4.17281647207471	-1.09664163910212
H	0.44839148165096	-4.01790959294284	-2.13692856278957
H	1.24285856024805	-3.80122424606052	2.11652429444079
O	2.23296313889979	-1.65869281380102	1.43605578962616
C	-1.78073400182665	0.64461530578581	-2.58656501669568
N	-0.55255823128622	0.51497051235709	-2.11378135179559
C	0.66816486839123	1.06563140731999	-2.60455469604033
C	0.80445817748805	2.41811453322734	-2.97648955943929
C	2.05247619657606	2.88740103241044	-3.41693688622457
C	3.16127099246550	2.02311214309171	-3.46779295576508
C	3.02440920770366	0.68076125535479	-3.06731718107781
C	1.78330024312025	0.20137699527723	-2.62819166350685
H	1.67063782988871	-0.83223781428842	-2.28512106499552
H	3.88912951845604	0.00641306934440	-3.07651929777138
H	4.13565290839387	2.39971106996891	-3.80321662753728
H	2.16081842403870	3.94236277215551	-3.69684512993576
H	-0.04475202978770	3.10106022728621	-2.89040542864394
H	-0.37512313040440	-0.05641522797713	-1.23308477094415
C	-2.86901308351298	-0.13562910714769	-2.00115581901220
C	-4.22243566328896	0.24652967973440	-2.23394772941880
C	-5.28109197117305	-0.52335765196620	-1.76987718360458
C	-5.02799726819003	-1.73615884848880	-1.07938326014072
C	-3.69085500931614	-2.11911805204619	-0.80997484640003
C	-2.63596232608975	-1.33044420169077	-1.26676657126331
H	-1.61863569461247	-1.68117293633612	-1.07364728352857
H	-3.47471067250314	-3.04415307520698	-0.27137371063280
H	-6.31820265726162	-0.21633306797470	-1.94418901296819
H	-4.44670498503385	1.17272614215696	-2.76987692547000
C	-2.05897959975712	1.55315612433566	-3.74959069494258
H	-1.17466620786422	1.64843801160204	-4.40117384707282
H	-2.31156205395407	2.55911750906090	-3.36134229637239
H	-2.91431217003080	1.18386276656257	-4.33616746488431
C	-5.91343065231191	-3.73154950485592	-0.08468131557302
H	-5.44880199824878	-3.57939813362735	0.90551694112976
H	-6.91966589092514	-4.16516429099165	0.03210191354914
H	-5.27579596164432	-4.39860597288559	-0.69539905150937
O	-6.10551819958118	-2.46746239800787	-0.74194869268647
H	1.63164030189149	5.66270904789766	3.21574309618520
H	1.14408816926914	4.86398608580009	-1.02373050516094
H	-1.84382987413250	-4.87059808184033	-1.65908706576201
H	-1.02832340036798	-4.62809189333582	2.59639560749385
O	0.37962308786930	6.41782697472727	1.11260715732090
O	-2.88178432507929	-5.22972072137779	0.65326347722052
C	-3.41381487136641	-5.31947365281578	1.97858513270991
H	-3.42104992346707	-4.32530811495973	2.46613200713519
H	-4.44774190873457	-5.68645528658373	1.86954463367742
H	-2.82906911012432	-6.03159576834723	2.59509140376188
C	-0.31328718498829	6.73709003583062	-0.09654128928547
H	-0.98512643675528	7.57789511148904	0.14587469289094
H	0.39412947113975	7.04355595933916	-0.89416916946514
H	-0.90950443301121	5.87333834479792	-0.45478288460250
H	-0.85341376384169	0.71607478899690	2.29619240141142
C	-2.03163941283215	1.89268684294231	1.07774378593446
C	-2.75274957181458	-0.09789072640249	2.28984120870795
C	-3.29686391149723	2.08880363740737	0.56091130442298
C	-4.05983275923777	0.06356087009429	1.88019014837751
C	-4.49176178054450	1.36306499641854	1.18955424150340
N	-1.82977166559196	0.88611621802839	1.99672343590402
C	-3.52267496136168	3.03079661785970	-0.53826661796517
O	-2.67171208877688	3.67443971903877	-1.15964165562082
O	-4.84660575963579	3.11598887998926	-0.85917697544987
C	-5.08079632057294	-0.94760319975728	2.18190120078859
O	-4.91647130187909	-2.02739764295001	2.75393430776466
O	-6.31175479067228	-0.54368305051622	1.75578022093796
H	-5.19984101746734	1.10294081382175	0.38940382661047

C	-0.79750548579916	2.67502377513850	0.71902804606480
H	-0.47728128999688	2.45125826567943	-0.31157301545553
H	-1.00234345958028	3.75584577342441	0.74960950991174
H	0.03538466605320	2.43774449077763	1.39947344363059
C	-2.17880702222169	-1.29531604459224	3.00594205446931
H	-2.21139886455176	-2.17793121082750	2.34077183086034
H	-1.12774707366757	-1.11155668675045	3.28761466860239
H	-2.77542969774879	-1.55064247466310	3.89757622287341
C	-5.21171350126941	3.94472176713180	-1.98276913948758
H	-4.87925048517566	4.98328421870088	-1.78960050287416
H	-4.67628603899094	3.58625605271393	-2.88430163728238
C	-6.72061049443056	3.85027693271976	-2.15099572259656
H	-7.02681083234296	2.80581372944091	-2.35136550134474
H	-7.23926531752520	4.20172284169654	-1.23916947104833
H	-7.03882057219898	4.47978852788559	-3.00314682956696
C	-7.45404439581373	-1.36754211304216	2.07086435556666
H	-7.20958010350917	-2.42760566346814	1.88501061432338
H	-7.67343855692847	-1.25903915093859	3.15227882570670
C	-8.61601194257905	-0.89928046919767	1.20855158062678
H	-8.84328175211278	0.16729834843807	1.39516073905957
H	-8.37348315592088	-1.03360408531821	0.13824772433884
H	-9.51639259531343	-1.49708158533974	1.44547932300762
C	-5.23054167889159	2.29076319427603	2.16071090085372
C	-4.58223356746032	2.76324201013310	3.32145695123729
C	-6.55828338675561	2.69313746395653	1.91622541395974
C	-5.24512243660318	3.61855292283453	4.21859811873091
H	-3.54783637844902	2.45419485900588	3.51949205489951
C	-7.22759694011849	3.54867782645064	2.80962158402460
H	-7.06640643161820	2.32599885072375	1.01631448784919
C	-6.57251425801931	4.01469361445615	3.96503945504320
H	-4.72619202193076	3.97705589378144	5.11770686689941
H	-8.26335253949902	3.85149493019625	2.60469480295852
H	-7.09303854134299	4.68241455782522	4.66423993565575

7.3.3. Conformer C9

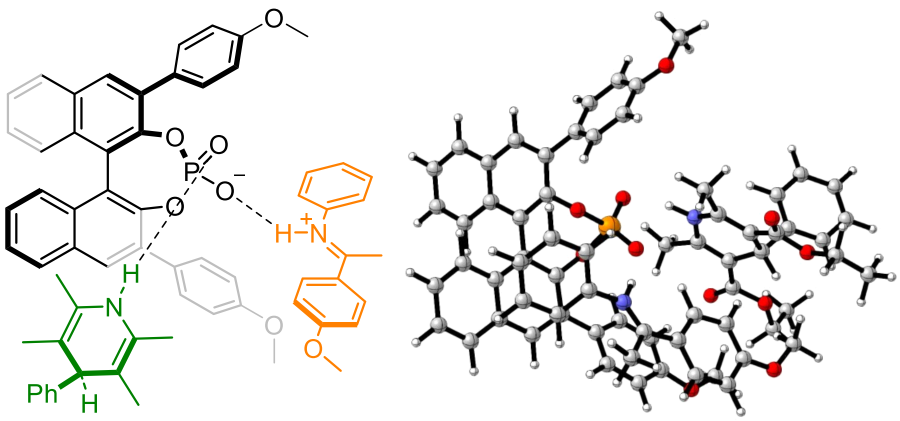

G_{180K}:-3901.593672130412

P	1.11194477179033	0.57153906855870	0.78420697996238
O	0.03698176220900	0.63356090563550	-0.28424779583514
O	0.88124349298779	1.15111046860823	2.16479931526968
O	2.49256442730850	1.20897194150678	0.09160587960124
C	3.66878116681610	1.05394565875917	0.79775400131846
C	4.30801661774675	-0.19124717950985	0.75983810630702
C	5.48242226096391	-0.38889107743070	1.57390055284917
C	6.13811367961035	-1.65246123036205	1.70174565000629
C	7.26847210568447	-1.79738689576362	2.49733458053254
C	7.80208066415951	-0.68505667575534	3.20832462205655
C	7.17412691761606	0.55051916158665	3.12944068668636
C	6.00071098225493	0.72890409219718	2.33178497320472
C	5.32371205560972	1.98153416174535	2.29404429956656
C	4.15329709872456	2.16884191026192	1.56109482884295
C	3.40028088938211	3.44882431171226	1.62051517599365
C	2.83696052848420	4.05246263370382	0.46684352525947
C	2.10763576379500	5.24067498840781	0.55459997911831
C	1.90930759051821	5.86976361699800	1.80770552775104
C	2.47521686236159	5.29144636672291	2.96611912277255
C	3.20938907624324	4.09829793639290	2.85945840803681
H	3.62024350949218	3.64555239648395	3.77057479697170
H	2.97686567279673	3.58351561789732	-0.51240568436534
H	5.73337685440545	2.81650668376024	2.87615094437828
H	7.56018642038398	1.41293196818826	3.68873950624611
H	8.69950137141610	-0.81102989374105	3.82755781278069
H	7.75091486340606	-2.77971328619602	2.58151153955935
H	5.73510991206235	-2.51766188956620	1.16609366291893
C	3.73595498159038	-1.28229523079718	-0.08618107587278
C	2.39196805772934	-1.65823917974747	0.07299998572939
C	1.77740034575826	-2.70907105564956	-0.69700032453900
C	2.60405962723737	-3.40087735067982	-1.58633275905160
C	3.95505187226679	-3.03632547864233	-1.82417901077693
C	4.73672066649611	-3.71020534493748	-2.81371750534001
C	6.02611399708409	-3.29049277568037	-3.10860445406230
C	6.57962279917585	-2.17061989611305	-2.42449566032457
C	5.85072878065534	-1.51021868146746	-1.44349536145246
C	4.53034789550025	-1.93158081507966	-1.09750933805460
H	6.28460753199935	-0.64486683154228	-0.93295417870758
H	7.58876729826368	-1.82182981151222	-2.67886368314338
H	6.61437923818597	-3.80719072845239	-3.87773275987149
H	4.28341243882254	-4.55364881384583	-3.35100500361243
H	2.19354863253930	-4.24049441331853	-2.15907764925494
C	0.32755218254725	-3.04391195387696	-0.62975521732763
C	-0.45182060974040	-2.95427088689259	0.54779703836423
C	-1.81575874880076	-3.29054132648929	0.56305136452427
C	-2.44276999486806	-3.74439811187648	-0.61806788689899

C	-1.67959553822291	-3.85687099681552	-1.80119529873697
C	-0.33115687621453	-3.50185083142034	-1.80322203199022
H	0.22259977094851	-3.55574393539111	-2.74752280578876
H	0.01215658319494	-2.62314082056032	1.47962682252389
O	1.63993756540222	-1.00479456008500	1.03068731111702
C	-0.91844629759488	-0.67577643487203	-3.57424239964835
N	0.06271986259781	-0.20681384417664	-2.82237284379808
C	1.41555178549607	0.05233203641381	-3.20538690830626
C	1.95697597547146	1.30739122972860	-2.86676473739126
C	3.29633359314559	1.58748691815245	-3.17427117039563
C	4.09570374929604	0.61609712367825	-3.80309926369656
C	3.55224883865751	-0.64080892490092	-4.12262974637842
C	2.21301073397534	-0.93003244304759	-3.82348890336610
H	1.79589278174228	-1.91854777395380	-4.03510589467122
H	4.18140641164230	-1.41498501607904	-4.57578383958703
H	5.14774259504747	0.82982787272133	-4.02809429593794
H	3.71803727076869	2.56412489560441	-2.90614657878509
H	1.32977375562186	2.04090267342094	-2.35136409572286
H	-0.08653130577629	0.04319096929277	-1.80321162926967
C	-2.25273680636642	-0.89623484779007	-3.02117270358295
C	-3.21507213256835	-1.62240865665923	-3.78535620706486
C	-4.49318301671679	-1.86483652448297	-3.30200882207375
C	-4.88153758889720	-1.37263010898610	-2.03069165417168
C	-3.94043316978257	-0.65896168568670	-1.24707650962510
C	-2.65291008739905	-0.43485557033883	-1.73596401273295
H	-1.95990858031322	0.11658472422380	-1.09407725103395
H	-4.20198596138838	-0.27659661490571	-0.25776112048368
H	-5.22124667676322	-2.43244260923211	-3.89239567015728
H	-2.95389774986197	-2.02524524917547	-4.76822119634663
C	-0.65818905489786	-0.97548664385588	-5.02662944567831
H	0.22999931528424	-0.44041627382118	-5.39721432391974
H	-1.53359667177891	-0.69765465106357	-5.63562491858293
H	-0.48661556055767	-2.06137675566228	-5.15860837616912
C	-6.67325392659266	-1.03102616122789	-0.46569337204847
H	-7.74535521335726	-1.28553129188333	-0.44423472847951
H	-6.17473690461192	-1.43496961788400	0.43241943446146
H	-6.54163205373724	0.06587920577866	-0.48982155686261
O	-6.14892724737352	-1.62961536856424	-1.66268897150253
H	1.67612601276704	5.70403452105235	-0.34116300346927
H	2.33774141137266	5.75156636746367	3.94975900408729
H	-2.17444010420029	-4.19047589573362	-2.71977673938471
H	-2.37989272534198	-3.19715480870612	1.49705203367158
O	1.16884996955531	7.01095944887357	1.79664410466537
O	-3.75552733682065	-4.08862401747011	-0.71494623218735
C	-4.58743731473683	-3.90376211993956	0.42848459253885
H	-4.26293982667479	-4.54701717946855	1.27066927237693
H	-4.56703469421448	-2.84945779386432	0.76090542026078
H	-5.61032917386486	-4.17400694666358	0.11965808019375
C	0.92565725134722	7.67838299897152	3.03528214523426
H	0.30695307241321	8.55997048575314	2.79655461188349
H	0.38178634765425	7.02003713155123	3.74357835756124
H	1.87504070169357	8.00464979529974	3.50776190576833
H	-0.86589063738276	1.30089639731675	2.46049963417907
C	-2.55736398819608	2.06909089173955	1.55729690616953
C	-2.42547383787114	0.01499405578872	2.87160002727312
C	-3.90484903601020	1.88470036670509	1.31995200772419
C	-3.76927743946522	-0.24724831420085	2.68509181760136
C	-4.68872642590214	0.85195205839992	2.13964803843404
N	-1.89792436053422	1.20120969065963	2.40606455607652
C	-4.61976324709076	2.71444316748560	0.33952925522304
O	-4.14736448947641	3.59778027227883	-0.37314409061927
O	-5.94750199662830	2.38600987715785	0.28064013862184
C	-4.35355474829074	-1.52379196441913	3.11092300787286
O	-3.77417790555860	-2.46290016725260	3.65659512684352
O	-5.69009849801168	-1.58323977872078	2.80820926266362
H	-5.43994265982876	0.38859310660367	1.48708925107179
C	-1.68574497865723	3.14543491782197	0.95631311689477

H	-1.59051761849330	2.99359403307473	-0.13289885334396
H	-2.14405707573603	4.13911922652023	1.10022673170109
H	-0.67760818088968	3.12150202375754	1.40231538469444
C	-1.43077398267078	-0.89360461683786	3.55241313787790
H	-1.38830005890616	-1.86819214181895	3.03926273645319
H	-0.42568055155242	-0.44094073316768	3.55442642121879
H	-1.75474500364157	-1.10767971381149	4.58655931312958
C	-6.77097726317784	3.11788237977483	-0.65365833736828
H	-6.63631341820217	4.20284847714743	-0.47922273504894
H	-6.42332170706193	2.90098055622186	-1.68335287632397
C	-8.21437418486457	2.68772471113337	-0.43819465303666
H	-8.33773497580468	1.60420941772808	-0.62230330362453
H	-8.53787131458716	2.90922074470716	0.59631218557743
H	-8.87066555028724	3.23851720681395	-1.13801860741476
C	-6.43566034796331	-2.75309425996998	3.21583573277058
H	-7.28652711508561	-2.79758913543938	2.51293039657214
H	-5.80890813709020	-3.65329239153990	3.08162171334171
C	-6.90871276882249	-2.62463995506123	4.66096407700149
H	-7.53525910657173	-3.49781196586315	4.92650269268754
H	-6.04320004256929	-2.59148841328074	5.34782741313153
H	-7.50913793308863	-1.70504629902807	4.79526368463394
C	-5.46398765429085	1.52566700625099	3.27731979612649
C	-4.77442250054423	2.19793576663204	4.30831368040370
C	-6.87152266975846	1.48248436894322	3.31915599226506
C	-5.47564251021727	2.81318164028010	5.35950125868539
H	-3.67777336993855	2.23582141660363	4.28362642079938
C	-7.57916485937142	2.09544617459132	4.36882272980304
H	-7.41085231202113	0.96252571716197	2.51797996543365
C	-6.88264982666273	2.76304176469188	5.39344581593540
H	-4.92433105632687	3.33230595188282	6.15484871786294
H	-8.67647816566767	2.05218847126560	4.38735379083150
H	-7.43265347919719	3.24221647051029	6.21407767748125

7.4. CPA 1b, *Z*-imine 2a, HE 3b

7.3.4. Conformer C10

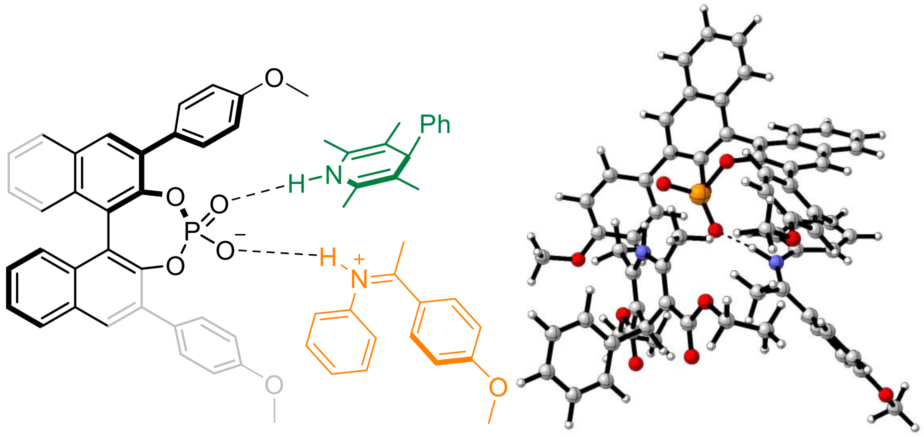

G_{180K}:-3901.52561100918

P	-0.54355359443233	1.26648400108325	1.31189119095858
O	-0.68001837507111	-0.06723168511768	0.59912650511141
O	-1.66285479683559	1.82064500291726	2.16090976732612
O	-0.05247962967431	2.32602082560886	0.09642506652925
C	0.48424246420374	3.54703332942406	0.45159139897426
C	1.77043256774641	3.59384248956809	1.00488096580247
C	2.26015019914189	4.85572821113631	1.50239745313458
C	3.50342828886653	4.98299430772873	2.19606739055723
C	3.93499544930259	6.21437391522723	2.67440161287319
C	3.14527789375248	7.38363180711858	2.48252855042132
C	1.92363584348615	7.29320238127802	1.82877779561861
C	1.44579434091613	6.03949380247207	1.33524412395159
C	0.18684309820684	5.93914639980862	0.67475812729630
C	-0.31080688303701	4.71981703841607	0.22362419188776
C	-1.60875717303551	4.62875113535077	-0.49647110801009
C	-2.77670502307812	5.22602064883961	0.01835784418953
C	-4.00551716810961	5.14514975179930	-0.66098008411356
C	-4.07868418953005	4.46312036660083	-1.89467886669803
C	-2.90731666999229	3.88617070344532	-2.43961711202276
C	-1.69955854031921	3.95991316812022	-1.74362863016561
H	-0.80422984975703	3.49383307057632	-2.17066260026556
H	-2.73370912617100	5.74387592288138	0.98497565916325
H	-0.39784305456475	6.85310697151830	0.50995833374635
H	1.29741478459273	8.18347352917524	1.68427127940398
H	3.49994874074786	8.35115597567414	2.86037464998494
H	4.89103445020713	6.28499979351487	3.20904754806873
H	4.11621989442030	4.08976429934476	2.35500086877660
C	2.58842920301152	2.34516943028406	1.07517533164900
C	2.07369565450638	1.21492506365845	1.72309675910641
C	2.79391734578437	-0.02454786333527	1.82942681720929
C	4.06948108819455	-0.06547544384880	1.26538349668538
C	4.63221866955707	1.03586375626686	0.56726615855593
C	5.92363597659397	0.94083367831499	-0.03599604654389
C	6.44297015013247	1.99284846442667	-0.77599258076180
C	5.67648358105354	3.17957639436953	-0.95223910213804
C	4.42544920645345	3.30855197911104	-0.36153909708730
C	3.87531448544048	2.25768102186022	0.43517142234531
H	3.84532828790084	4.22476889974690	-0.50959885635455
H	6.07501338177448	3.99926717795290	-1.56388547233317
H	7.43196819259895	1.90605663425642	-1.24377918360518
H	6.48558269145808	0.00541167937963	0.08150663416531
H	4.66363051146384	-0.98289404410519	1.34891719608233

C	2.22098942157314	-1.23943225088361	2.46827319718562
C	2.50939542518884	-2.52066994544191	1.92926849871565
C	2.00416774789859	-3.68970175674948	2.50268369724139
C	1.18028343178055	-3.61953775632850	3.64995203657815
C	0.89068858057787	-2.35716881234543	4.21142331335356
C	1.40835374530768	-1.19236108331462	3.62397975119419
H	1.17497021158478	-0.22860526398594	4.08452260286414
H	3.12476747574067	-2.59832774705925	1.02627959665619
O	0.81752447943787	1.30986095200534	2.29044810119187
C	0.46995592437505	-2.92860930681818	-0.79107806547029
N	0.87302381434544	-1.67439524168142	-0.80617436909409
C	1.96623848698747	-1.05299127283828	-1.47319043934348
C	1.79622507662298	0.29493597355647	-1.84396266363144
C	2.84576338474776	0.97708943461300	-2.47900117184590
C	4.06185205780928	0.32349595983027	-2.73277281308552
C	4.23649313993932	-1.01263303870412	-2.32456454831948
C	3.19851822520430	-1.70440844692500	-1.68841583742583
H	3.34122102767214	-2.73774081357928	-1.35949714683878
H	5.19786467286803	-1.51553522927904	-2.48563759654623
H	4.88420986648755	0.86125010494810	-3.21774860258270
H	2.71254453369949	2.02745098576970	-2.76298102920336
H	0.85007161606515	0.79905955339854	-1.62391528502011
H	0.29909989939129	-0.99614821369096	-0.20829189201747
C	0.97370225133633	-3.96897780544977	-1.68927493380551
C	1.06614957551596	-5.31223833067769	-1.23228460673359
C	1.50148570809779	-6.32623975584516	-2.08147615790826
C	1.81938913270948	-6.03792847139937	-3.43401646583599
C	1.68971768373010	-4.70881853464092	-3.91440106087639
C	1.28343825970062	-3.69492817019709	-3.04502997358729
H	1.17820307641266	-2.67610905766930	-3.43213851686876
H	1.89852450668646	-4.46657242447554	-4.96058821451067
H	1.59954878259170	-7.35828149739534	-1.72545561243678
H	0.81919048702245	-5.55297154339102	-0.19263869183466
C	-0.59503626706314	-3.28992650403943	0.19884047617725
H	-1.04687467825131	-2.39920955562085	0.65719973362049
H	-0.13694106048522	-3.89689560046107	1.00159079405048
H	-1.36824767594435	-3.91366407121782	-0.28021683681969
C	2.55267319373279	-6.86724397771939	-5.56339731629394
H	1.67652020555426	-6.48111751178397	-6.12092405795663
H	3.39277107074089	-6.15122567219694	-5.66035641621062
H	2.84896095204727	-7.85120361348422	-5.96229494731506
O	2.22308474725861	-7.08168738269832	-4.18404063633986
H	2.22141065374646	-4.67007277750601	2.06210981689078
H	0.26439675580223	-2.26995049737792	5.10433262853403
H	-4.89637151175797	5.59808643338867	-0.21474890232131
H	-2.97274918795253	3.36331686844283	-3.40077732533571
O	-5.22147183773546	4.29402853714258	-2.61620412279698
O	0.71053568103598	-4.80302428264962	4.12984287553282
C	-6.45186219074905	4.77036401988583	-2.06905737861650
H	-6.66053221466316	4.29337090888837	-1.08959475112351
H	-6.43021959104953	5.87125799350801	-1.93618796850078
H	-7.23835239669442	4.49731842917084	-2.79249411611441
C	-0.16155579960994	-4.78106116345578	5.26148320537662
H	0.35355249341056	-4.36585212621902	6.15159494149585
H	-1.06629688506234	-4.17628344584851	5.05343405342078
H	-0.44859119865812	-5.82886490171914	5.45034939368746
H	-3.12282386369315	0.54558946053833	2.00881875041665
C	-4.15284905143571	0.17377665457959	0.28605245215445
C	-3.37711977198906	-1.49492315322539	1.90472939693829
C	-4.64126509849879	-0.80686328414116	-0.55328829041031
C	-3.87740258819752	-2.53217876264030	1.14345025244992
C	-4.82767102541024	-2.22784357787617	-0.01778663168780
N	-3.65409966931875	-0.19306033783034	1.52509325199332
C	-4.07227858074196	1.65339101446747	0.00526479984834
H	-3.80337619120091	2.21046308186732	0.91589642825507
H	-3.30167029936407	1.86417410359970	-0.75575067145128
H	-5.02330601578581	2.03013030041627	-0.40233796422354

C	-2.50831055012747	-1.60776568096349	3.13446389472596
H	-2.26920126219466	-0.60795790783347	3.53598366831644
H	-3.00817978728191	-2.20910694983089	3.91354958880564
H	-1.56074735998949	-2.12034521308572	2.89825599558175
H	-4.61613218860641	-2.93649890312137	-0.83497740968554
C	-3.64444178402144	-3.96811777690336	1.36084522298869
O	-4.09157970053559	-4.83986076722937	0.61463480382595
O	-2.89219752215084	-4.27972287653385	2.45608760230563
C	-5.05537876844963	-0.61341867027864	-1.95177912993199
O	-5.47219434825933	-1.52346080254962	-2.66904728057648
O	-4.91766717769140	0.66303106022135	-2.40911553671232
C	-2.69501602950574	-5.68690661692905	2.75080568730809
C	-1.53649450417883	-6.28893110212216	1.96123128835696
H	-3.63503545782945	-6.23199086123595	2.54711931103761
H	-2.49125188969995	-5.71282694863822	3.83474316711424
H	-1.73490673304915	-6.23969343213562	0.87611596371706
H	-0.59308627511692	-5.76226699577457	2.18792215521098
H	-1.41704824609979	-7.35137429180429	2.24932288455342
C	-5.22578844356614	0.93767545326311	-3.79596306885231
C	-4.04636444736037	0.59345139702693	-4.70212621353384
H	-5.44054482592506	2.01985168408422	-3.81378402942862
H	-6.12984192974778	0.37235138089475	-4.08976829396238
H	-3.13852021990695	1.13908538458628	-4.38124627866369
H	-3.84077585927071	-0.49226735929814	-4.67718127100177
H	-4.28043435804664	0.88417867263861	-5.74452712704202
C	-6.28902636470555	-2.45423860501522	0.38871880008431
C	-7.12382708713167	-3.31509759668780	-0.35076176028461
C	-6.82954966196397	-1.78299616773233	1.50713230333270
C	-8.46864668532035	-3.50533723208882	0.01587526654273
H	-6.70838409603758	-3.83541978780923	-1.22273071855193
C	-8.17246681742238	-1.96779324998860	1.87769110759863
H	-6.18793117005438	-1.11061225097806	2.09059806447063
C	-8.99799321341127	-2.83145831614777	1.13196973935263
H	-9.10487190849347	-4.18197728370387	-0.57042667615303
H	-8.57686635512440	-1.43796704578491	2.75062964818644
H	-10.04725749261451	-2.97869680030616	1.42023894279508

7.5. **[3:3] Dimer D1**

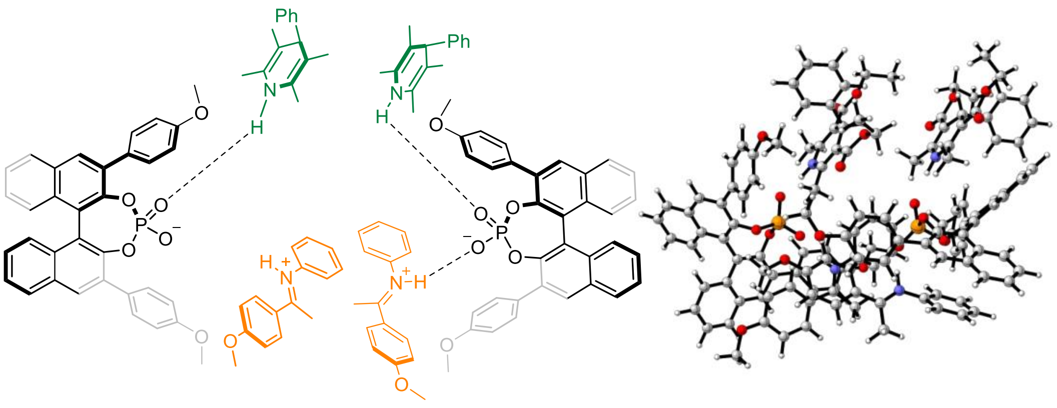

SCF energy: -7805.338511254652
G_{180K}:-7803.119928054650

P	-2.33701653557011	-0.53375619627645	0.24726283801857
O	-2.96372606833096	-1.12231733761423	1.48926578275918
O	-1.03693326613035	0.24571764121634	0.29985539407710
O	-3.54213262368103	0.33963782758643	-0.52962302045434
C	-3.30903370129174	0.76589637296334	-1.82541779946089
C	-3.35751320663952	-0.19053120772854	-2.85077675592597
C	-3.00346969811455	0.20378023582988	-4.19020380365078
C	-2.85189642957022	-0.72851784441260	-5.26162592060253
C	-2.48299275926955	-0.30607876284033	-6.53280065389313
C	-2.26206672791134	1.07538549971220	-6.80057564066401
C	-2.40100528746590	2.00759447528873	-5.78200136896388
C	-2.75639859187802	1.59934137362998	-4.45895547587527
C	-2.82511828236458	2.53723827151486	-3.39504522643862
C	-3.04714773864588	2.16101654047172	-2.06841260101932
C	-2.95317539331305	3.17682420713448	-0.98498680163251
C	-3.72286363680030	3.12768931157781	0.20208913467512
C	-3.61073428214172	4.10932421585704	1.19926620724569
C	-2.70968228665839	5.18329797131357	1.03390840778743
C	-1.93992589113684	5.25817085316903	-0.14915268587355
C	-2.06737980382352	4.27716000895457	-1.13297777770879
H	-1.43956920250643	4.34978764333105	-2.02600093176091
H	-4.43516889132413	2.31298795603586	0.35471139770966
H	-2.65165755585588	3.59244859597201	-3.63409314318009
H	-2.21289867764808	3.07283001298494	-5.96574077035846
H	-1.97268913380006	1.39734905158008	-7.80917265961370
H	-2.35717172480778	-1.04431352391067	-7.33522069026014
H	-3.02208043308138	-1.79152405161782	-5.06674190447039
C	-3.78648001440213	-1.58812774559924	-2.54259344723882
C	-3.12342162717465	-2.32626431413059	-1.55184051765125
C	-3.48782318808332	-3.67704458633016	-1.21239899191419
C	-4.53284571280295	-4.25330971118529	-1.93684728256316
C	-5.26586167443923	-3.54391192383140	-2.92668231358394
C	-6.35855260760993	-4.15769725175209	-3.61573396260148
C	-7.10712843154455	-3.44450265466554	-4.54158527210588
C	-6.79503042148751	-2.07957687185193	-4.80105328347917
C	-5.72902354863996	-1.46127418234677	-4.15821571011404
C	-4.91536435616714	-2.17477797413612	-3.22328466018079
H	-5.51029406194466	-0.40933308537434	-4.36513637564482
H	-7.40516005131652	-1.50729573035074	-5.51178310196909
H	-7.94673318854049	-3.92295185678863	-5.06200308906551
H	-6.59891642805777	-5.20382703115382	-3.38441369985088
H	-4.81231632902567	-5.29470015114363	-1.73642457978705
C	-2.82219732678921	-4.47038411116766	-0.14364432350229
C	-1.42725381161487	-4.44842974377464	0.07233322091767
C	-0.81021587436314	-5.28360047744701	1.01569926325442

C	-1.58544329818975	-6.18165284744476	1.77998816211434
C	-2.98962334622588	-6.19882497097112	1.60079106561642
C	-3.58840984101186	-5.35848891601396	0.65843624246084
H	-4.67916608811654	-5.38188815648120	0.54391176354566
H	-0.80800266009305	-3.79218134625082	-0.53754363995948
O	-2.06635047678415	-1.73091297529035	-0.89853361749028
C	0.88623628201030	3.36906089285919	0.30636725622123
N	0.09395480381119	2.59986965827046	1.03410958379470
C	-0.14784190898384	2.66702116118911	2.43744092417831
C	-0.41276210313299	3.88842808340506	3.09007348581198
C	-0.67846040025800	3.89428958023117	4.46715393446039
C	-0.68381964587243	2.69085471446177	5.19285460453602
C	-0.43214994124298	1.47649618820927	4.53178570433593
C	-0.17580007576540	1.45346446494367	3.15436202910623
H	0.01269310887764	0.51400070361614	2.62974986942625
H	-0.42861910083222	0.54024438017658	5.09490088746660
H	-0.88752767901218	2.69366616018290	6.27050378722136
H	-0.87789266385627	4.84622280167560	4.97025190828493
H	-0.44058598296372	4.82206316089371	2.52512446210873
H	-0.39581564854290	1.77637306000405	0.60090444490222
C	0.94083397207359	3.21683162113425	-1.14525326960129
C	0.32558546445814	2.12922439219386	-1.82714946288145
C	0.32844083733232	2.05534200510614	-3.21067568206491
C	0.94620805894649	3.06737125538527	-3.98421163914017
C	1.57694448665086	4.15248669769525	-3.33087541033373
C	1.57323371013801	4.21328446458464	-1.93474362382649
H	2.05559387745874	5.07456294448918	-1.46383481175686
H	2.06716430938214	4.94656738109645	-3.90117182881536
H	-0.14184796471319	1.21258539440537	-3.72161111514779
H	-0.15732679913807	1.31993161840705	-1.27461268957669
C	1.72423418410295	4.41609222530794	0.98179908405566
H	1.21048726587384	5.39571297455166	0.96053983865181
H	2.68677065415562	4.53146848315997	0.46135526598592
H	1.91523041946722	4.14940476806871	2.03162878257108
C	1.48221222041787	3.90103566232218	-6.17121885305563
H	1.00978410713644	4.88798286241410	-5.99980036125913
H	2.57156800132051	3.97671426108249	-5.98597405070579
H	1.29682916829273	3.56734399994129	-7.20512762741699
O	0.89325897312722	2.90566260983404	-5.32314776418215
H	-4.22702055890289	4.02703531955327	2.09982634839846
H	-1.23104752288819	6.08602871249054	-0.27042250475784
H	-3.59342316801548	-6.87759498062934	2.21522502098131
H	0.27546172155011	-5.24341765995907	1.13710274489392
O	-2.50499913679162	6.16953156311322	1.94959310952986
O	-1.07683097482063	-7.05502244705728	2.68597611848358
C	-3.27367799410029	6.14918304181482	3.15478522271984
H	-3.08938170001320	5.21900746467106	3.72770563023407
H	-2.94499011217146	7.02061124886832	3.74566337551767
H	-4.35743801798146	6.23078464921473	2.93413179439156
C	0.29427756975220	-6.92252973550909	3.06465429014837
H	0.46331696448168	-7.65101029194817	3.87372452122260
H	0.96991059547604	-7.13995261196186	2.21229951476178
H	0.49758056069395	-5.90218193237376	3.44110748770914
H	2.64336870839209	-4.68902856144820	-0.28407103227439
C	3.17388503183917	-4.08015686361365	-1.02279670262933
C	4.07649053176755	-3.10154164328352	-0.57739442415123
C	2.95423972586407	-4.26045213845179	-2.40610926994543
C	4.78085052596546	-2.27063473820916	-1.48123889146486
H	4.23528760894838	-2.99900692690724	0.49903848930138
C	3.66021564344616	-3.45222981560282	-3.32474700480584
O	2.08797157244339	-5.16110096488988	-2.94365231802607
C	5.81051086048223	-1.28720212027392	-1.04517098905297
C	4.54322022167785	-2.47659168239935	-2.86588403394797
H	3.47112418870408	-3.58010704572134	-4.39633603318730
C	1.29363413884002	-5.95106598850907	-2.05644109431048
C	5.74422387250103	-0.56485813556018	0.20059854932629
C	6.94185469502872	-1.07721892808491	-1.83747086971530

H	5.04703057597623	-1.83773782865445	-3.60039599990645
H	0.66819613069992	-5.30793738455432	-1.41171925071025
H	0.64509908204339	-6.57441284360813	-2.69417545794154
H	1.93021780534746	-6.59834678120218	-1.41943886766493
O	4.62416386739446	-0.71855803291029	0.98629450492200
C	6.81124084810180	0.21187233214664	0.67632999544030
C	7.98111834922650	-0.18032661192688	-1.47389230722725
H	7.04785823080768	-1.62860300867250	-2.77867312685167
P	3.61301386227401	0.54419644988712	1.45355987087026
C	7.92296060861214	0.48386805839619	-0.19630515881780
C	6.82743930014064	0.68057493938371	2.09356288604387
C	9.08847315986925	0.06639962977858	-2.34227731321117
O	2.95518224560283	0.06542900536389	2.72722871255857
O	2.79602251979046	1.03653082969108	0.27256601500467
O	4.72685944976508	1.75832908301562	1.75648842003034
C	8.96654726268366	1.39747950668072	0.14284361808723
C	5.78280019039602	1.45883705606527	2.59964248996633
C	7.87927223265060	0.25589306222012	2.98593102220129
C	10.09233839533553	0.95365238107850	-1.97947848362652
H	9.11903333694649	-0.45214381772538	-3.30954085127963
C	10.02302306184388	1.63086635674372	-0.72870602153928
H	8.91759040583327	1.92694783240346	1.10030858707547
C	5.75335694750015	1.94622432264249	3.94974013017820
C	7.85639743613803	0.71908554388326	4.35307929653430
C	8.89951967128597	-0.67181533542615	2.60826987303794
H	10.93437862800068	1.14317772520821	-2.65749571103459
H	10.80771107465581	2.34708236876407	-0.45307421853593
C	6.81041610684111	1.58162425677395	4.78170375831759
C	4.65299308783649	2.79145146675655	4.47939086495011
C	8.86281704075046	0.27452400908676	5.26568071125241
C	9.85167801462413	-1.10360292805915	3.52354476388552
H	8.91328399244551	-1.06744554069179	1.58849808663459
H	6.83672009043115	1.96461602226225	5.80765064072331
C	4.10860648401496	3.87887322870184	3.75057088358605
C	4.15204638179427	2.56027798655293	5.77897550223540
C	9.84223032889106	-0.62198067830954	4.86294841025888
H	8.82478961858584	0.63630903170861	6.30011858220988
H	10.61253440267219	-1.83045319865458	3.21145197884075
C	3.12566945165035	4.70107462511501	4.30871251897774
H	4.47274394869466	4.08754326604724	2.73923569536180
C	3.15313147488565	3.36431263939822	6.34764824465613
H	4.52376415379283	1.70742160439523	6.35593601116236
H	10.59880284805852	-0.97163998241784	5.57689102744287
C	2.63695806847357	4.45442032544043	5.61441717524513
H	2.71770102584083	5.55069030316225	3.74903259256577
H	2.77684785802427	3.11589908102431	7.34408583205536
O	1.68320623013181	5.30888936253625	6.07151203506186
C	1.13354032875489	5.07937428570135	7.36929122941142
H	0.37805674474009	5.86678630287022	7.52970632989531
H	0.65271044847580	4.08250820032354	7.42550245499903
H	1.91749606526584	5.14472678656261	8.15115223286018
H	0.55525927770316	-2.29631237092002	-1.02021222340430
C	0.85932755026122	-1.90094911469858	-1.98969823325282
C	1.81017405795827	-0.90141999461529	-2.06323682216337
C	0.28807257242697	-2.43999709070215	-3.17002717304548
C	2.23170240719688	-0.36673415110396	-3.31672170997771
H	2.23537602695856	-0.54333000700502	-1.12455395358539
C	0.68924838010187	-1.93149945085551	-4.42820696136006
O	-0.59972451216267	-3.43120075975941	-2.99521477684061
C	3.24604063275181	0.67135942372087	-3.41004136142268
C	1.64228042358534	-0.91371779835577	-4.48896901142094
H	0.26532912205410	-2.32796954358679	-5.35476067433269
C	-1.11014935787424	-4.14173412211181	-4.12799381762281
N	3.76996458395455	1.19603698990368	-2.30636971740948
C	3.69119717874673	1.16515806574885	-4.75850049847859
H	1.92790607504958	-0.55098114897465	-5.48015510303887
H	-1.66242728289664	-3.46151311641517	-4.80248981580427

H	-0.28012114927564	-4.62385786389552	-4.67909461972156
H	-1.79534990282484	-4.90269767353450	-3.72148227285102
C	4.87326399535907	2.08397858503171	-2.17575505913661
H	3.33315321486409	0.99617197415744	-1.37058693263865
H	2.81152402284513	1.28329951816146	-5.41005723878112
H	4.37013349615473	0.43193639396654	-5.23439906772823
H	4.20855733271509	2.13352441749149	-4.68845916938414
C	4.80050106724170	3.05949752829287	-1.15931920200184
C	6.03995139931065	1.96298618942159	-2.95844093450376
C	5.87618769788330	3.93256692538879	-0.95256093598121
H	3.91032793631127	3.09859168035302	-0.52920083049783
C	7.10698473388434	2.84810222123546	-2.74944130192854
H	6.12892721569703	1.15971034205637	-3.69368022341960
C	7.03000412734653	3.83552510275843	-1.75197388014051
H	5.81294940988658	4.68544158314135	-0.15682105376518
H	8.01818877577713	2.73835923428658	-3.34745318203683
H	7.87612553175507	4.51326342462161	-1.58495452465133
C	0.13421747266028	-2.55057128689750	3.30124697681289
C	-1.61153646699569	-1.70307813383380	4.79115367647154
C	0.81706749193512	-3.13261449153079	4.35252274665309
C	-0.95328511291448	-2.18998950894755	5.90030565416816
C	0.14890692954799	-3.23698094711802	5.73380450611440
H	0.91826894011763	-3.05446215100537	6.49995736625803
C	0.64606823087449	-2.37859351212321	1.89527293579057
H	1.37764259684209	-1.54801704768578	1.88916168953153
H	1.17053347978141	-3.27504446390219	1.53998907774626
H	-0.17499843326144	-2.12790081160525	1.20896768609741
C	-2.87148222769050	-0.87089688015361	4.80784533259053
H	-2.66455538666363	0.13176877152894	5.21965019117107
H	-3.27578077328641	-0.76232410647145	3.78881694621742
H	-3.62413626549119	-1.33178216699550	5.47116749653218
N	-1.10267339125563	-1.97422885691286	3.53449411661415
H	-1.66541796502957	-1.65953225281204	2.72483889412506
C	-0.40247666620103	-4.64731342897784	5.98075265727329
C	-1.56132918876297	-5.08592060075922	5.30871236611609
C	0.22762626549855	-5.52491962730531	6.88559644369767
C	-2.08333959783695	-6.36968819592511	5.53713309650386
H	-2.05808334115407	-4.41430342639945	4.59787415091862
C	-0.28555142429332	-6.81335150135917	7.11320563878856
H	1.12703292152519	-5.18512117611613	7.41212915571563
C	-1.44615782803143	-7.24002694468669	6.43975373760495
H	-2.98323728511817	-6.69435063399046	5.00025528392368
H	0.21779463433490	-7.48503345248533	7.82174436744084
H	-1.85106847223940	-8.24493170305630	6.61846685814844
C	2.13882800244052	-3.75887546564657	4.16907177051526
O	2.76974168449418	-3.88706932240335	3.11999830887402
O	2.61346709928836	-4.24538286239875	5.35454641667684
C	-1.37566951957633	-1.81055708947507	7.25646198611494
O	-2.12985894468873	-0.88823969273245	7.56260224174296
O	-0.81232078914988	-2.63452749281098	8.19061735177148
C	-1.08639493959723	-2.39656717852889	9.58948953522045
C	-0.01897520038501	-1.49493709388869	10.19793314644588
H	-1.07346530587320	-3.39958913513952	10.05377038886651
H	-2.09598083287628	-1.96010080006397	9.69940326965355
H	-0.19288819166782	-1.38237440579076	11.28526138930995
H	0.98461724313536	-1.92945048446690	10.04516361719524
H	-0.03778105433425	-0.49293126829776	9.73250042989187
C	3.77733240062558	-5.10235833915365	5.33799492874328
C	3.40417158339677	-6.53640718202731	4.97312534655275
H	4.18781908563728	-5.03543349623739	6.35897123274877
H	4.52178855450008	-4.69938736849985	4.63305128145385
H	4.30223566409553	-7.17848850493450	5.05125282302606
H	2.62566824475559	-6.92765385457046	5.65396409173574
H	3.02830307523615	-6.58462210953612	3.93521675241663
H	3.73439346926994	-0.77062877705031	4.14583665640133
C	5.16666198713173	-1.96404017077954	5.07582355250764
C	3.36983290537283	-0.73439552360329	6.17941237851922

C	5.62163117947211	-2.46750774821439	6.27766931137461
C	3.81703071319902	-1.08156051982959	7.43505705073169
C	5.04253969511589	-1.99211262917467	7.61974672345939
N	4.07721233088482	-1.12166371737828	5.05859843842300
C	2.08269731353155	-0.03640091702571	5.84437115906455
H	1.44640859296682	-0.75111042047319	5.28941827641481
H	1.55295235709616	0.31675020930225	6.73612324035980
H	2.26888080481019	0.81149998872900	5.16580786038062
C	5.71684032497082	-2.30416734435696	3.71595036889883
H	5.10837575774226	-1.84468566074769	2.92431597306257
H	6.75743759737090	-1.95497143727706	3.62595950404451
H	5.74738614350016	-3.39685335147023	3.56928665083146
C	3.20635012143597	-0.51666653663827	8.65493459804739
O	2.55603364313639	0.52392216009485	8.74754968860555
O	3.51114048110911	-1.27046894457799	9.75286614240913
C	6.65116581886972	-3.51544140651634	6.28725614618917
O	7.42385183908133	-3.80873117833235	5.37629093651807
O	6.64464311453124	-4.20276972304190	7.47211566938778
H	4.73523975131975	-2.87490190709899	8.21034417846482
C	6.14123451330917	-1.28784822199710	8.43073353739649
C	6.54026233054322	0.01642280751303	8.08542075725487
C	6.77046045044351	-1.91927735905010	9.52147507000693
C	7.54080557497407	0.68526814808671	8.81130621473955
H	6.05496671708153	0.50911435681411	7.23681402752089
C	7.77655796308579	-1.26107746155601	10.24920808309347
H	6.45630636453314	-2.93162232623719	9.80116123507401
C	8.16578601791133	0.04579561302574	9.89756415183435
H	7.83279654634016	1.70495959225078	8.52692918634051
H	8.25341378486933	-1.76508240077089	11.10048022149320
H	8.94817529320202	0.56242593611438	10.46887903562386
C	3.29472128093476	-0.68258458406195	11.05961610940096
H	2.30584899784541	-0.19177458936711	11.08152730799302
H	3.28364277887057	-1.54418738249369	11.75004515590653
C	7.56385264924046	-5.30722546414530	7.62043501269395
H	7.70149425863975	-5.41124483825715	8.71181017243917
H	8.53275916588855	-5.04207942958554	7.15776049094868
C	4.40700446318430	0.30207939853672	11.41170571117444
H	4.39054339553293	1.16512986654255	10.72164005580126
H	4.25635106387271	0.67478460982366	12.44328364459264
H	5.39700668608280	-0.18473207530858	11.34537320779820
C	6.99793179784001	-6.58641104628803	7.00767583966674
H	6.86320121504991	-6.46472939302638	5.91765682268101
H	7.69657612277519	-7.42649317258546	7.18618080754858
H	6.02098758378520	-6.83885448718327	7.46173447273612

7.6. Filtered out conformers. (SCF Energy only)

7.6.1. CPA 1a, E-imine 2a, HE 3b. (C101)

EE: -5020.367976240068

P	0.03275239632947	0.07176877378331	0.15323991579432
O	1.53963984162523	0.09048609644905	0.20498490015364
O	-0.77584462524633	1.35468299350046	0.11210742851621
O	-0.47629637743865	-0.90382465881297	1.42122398853188
C	-1.74685640324898	-1.44656830327425	1.35029400535428
C	-1.94092626982224	-2.58443443809503	0.56016004102276
C	-3.26817632877488	-3.14079905094654	0.45584990073050
C	-3.59109948016253	-4.23687571268892	-0.40304617564982
C	-4.88837387828220	-4.72922163651496	-0.48017483771249

C	-5.93401856217746	-4.14917413623113	0.29246827122031
C	-5.66295668006543	-3.06512792256851	1.11543197853643
C	-4.34167128431541	-2.52745139138010	1.20743719513218
C	-4.08568861175819	-1.35319980994564	1.96905993702569
C	-2.81806499277811	-0.78388795813743	2.03412633086711
C	-2.60783247434901	0.54515653138374	2.66439646465485
C	-1.51493732533112	0.81495825171573	3.51073782049052
C	-1.30820293199690	2.11144843414771	4.01252472424786
C	-2.18049526001068	3.15916124329931	3.68038729731643
C	-3.27841023576479	2.88860500834464	2.84493250861296
C	-3.49422458463100	1.59628793341843	2.34490706058422
H	-4.32896201364643	1.40863077616943	1.66241270954598
C	-4.16684520749455	4.02754599383091	2.39046634736188
F	-3.64923707425313	4.63841391049524	1.28845256827108
F	-5.41348671376599	3.61269441450627	2.05920489282333
F	-4.30137114804912	4.98588791449477	3.33961868359629
H	-2.00669678799596	4.17027161587782	4.06018200286496
C	-0.12831282888240	2.34168736549489	4.93416141706855
F	-0.31385579892706	1.73200936032833	6.13523607839898
F	0.09675171333133	3.65239931695674	5.18373809757777
F	1.01612122440328	1.82879281819126	4.41277055086718
H	-0.81444473416138	0.01484607343515	3.76632141089202
H	-4.92273854926420	-0.86833501473675	2.48602638094433
H	-6.46130291177075	-2.58863985329724	1.69905125018787
H	-6.95272267085567	-4.55135024534383	0.22402442247702
H	-5.10917706837432	-5.57071490787876	-1.14923052190888
H	-2.80334510986693	-4.68926864660119	-1.01247392445105
C	-0.81589282726844	-3.08236969980883	-0.28650178422020
C	-0.21596965538113	-2.18865940729122	-1.18101315704873
C	0.73344661822777	-2.60728500900546	-2.17026528915270
C	1.19988252254764	-3.91675383399844	-2.12008135672231
C	0.70942138411215	-4.84420392784095	-1.15713072139971
C	1.21733316675388	-6.17697685245951	-1.09307922179425
C	0.71861716338788	-7.08234557348362	-0.16729319444205
C	-0.29408616989215	-6.67937984882688	0.74771886132389
C	-0.80454998494827	-5.38708265256297	0.71466041850416
C	-0.33366728308363	-4.43790188779327	-0.24154921481641
H	-1.57377656132691	-5.08255126797676	1.43216862011338
H	-0.66883283777679	-7.39313189554589	1.49188986718632
H	1.11484543567258	-8.10406726078073	-0.12604777722101
H	2.01955548584804	-6.46532212117815	-1.78349791529552
H	1.92933732233205	-4.26186057412307	-2.86259185587509
C	1.08054043544744	-1.71039593502098	-3.30325348871104
C	0.04272986071322	-1.01573792714666	-3.96361890646259
C	0.30409228120054	-0.25177633519603	-5.10884150671246
C	1.60957179206708	-0.14767285331456	-5.61322444968629
C	2.64361361831334	-0.83715599913383	-4.95862446293024
C	2.39021474278505	-1.61480882313576	-3.81488108344656
H	3.21010223441537	-2.14435780664436	-3.31814786883649
C	4.05038912055315	-0.68446474771648	-5.49232362008359
F	4.09170162400561	-0.69970146962190	-6.84547853939345
F	4.88520917835560	-1.65124553609749	-5.05147610081043
F	4.58396351748962	0.51351499769263	-5.10548593168505
H	1.81592051597588	0.44694399834506	-6.50838508269449
C	-0.85605074711768	0.34342449048326	-5.87664372850405
F	-0.50836941973687	1.45325608681343	-6.57453667785223
F	-1.35180023722917	-0.54407996303186	-6.77718977672926
F	-1.88917466540125	0.68692781996064	-5.06257765421896
H	-0.98317657061303	-1.09999361057787	-3.59538328825397
O	-0.55606681475387	-0.84978262441074	-1.13833688322031
C	-1.50722514847471	3.00706599799990	-3.10530791010285
N	-1.98369835395664	2.39086210735186	-2.03549245441503

C	-3.34069170400465	2.14917419931833	-1.66335675880883
C	-3.67236315761067	0.83614074640999	-1.26813987244243
C	-4.98086485572610	0.55206461604082	-0.85143203930027
C	-5.94779054589801	1.57390957639645	-0.80753579881705
C	-5.60123644326770	2.88554955478159	-1.17881503314183
C	-4.29728259754080	3.18100097848258	-1.60650020753652
H	-4.01193613001668	4.20563349153693	-1.86577447661281
H	-6.34341385003882	3.69060723513100	-1.11729834408786
H	-6.96714369382047	1.35098455251912	-0.46869545379971
H	-5.23777983602138	-0.46940113430564	-0.54856184058264
H	-2.90397903039635	0.05635415806499	-1.28604002157487
H	-1.33653726032259	1.95952037232303	-1.31872421773402
C	-0.05932609559005	3.14705607793212	-3.27654182533825
C	0.86221767833882	2.86992498155513	-2.21929373457393
C	2.23362940274084	2.93363796374633	-2.41819525314106
C	2.76244408976626	3.27966795579595	-3.69002738127610
C	1.87002610485346	3.60293636411729	-4.74146340774223
C	0.49155524466273	3.54472023340840	-4.52598617130555
H	-0.15771338597021	3.79259836347293	-5.36846562276233
H	2.24450788610427	3.89108652224180	-5.72782895213279
H	2.92584024792438	2.72196752964823	-1.59669897456359
H	0.50669934516385	2.61488527068192	-1.21768521559293
C	-2.45862964514011	3.54014167887662	-4.13948020484710
H	-2.08447038231127	3.33947103697724	-5.15426500468963
H	-2.54793269327206	4.63781664979894	-4.02373646465838
H	-3.45907838846723	3.09253858778851	-4.03991141007955
C	4.70750233281699	3.64453595362593	-5.04534916231077
H	4.46071362443346	4.69152276965186	-5.30905581482421
H	5.79400021212035	3.53490847766474	-4.89726540822558
H	4.36739436895516	2.96735698889654	-5.85039551588706
O	4.10144394793196	3.28599392533149	-3.79353597524973
H	2.87380635809736	-1.04635914030737	-0.02157795715600
C	3.52704630147538	-2.98793128050398	0.27802133315171
C	4.57005929757575	-1.31342878783259	-1.18184131055834
C	4.40524671748124	-3.96586954866501	-0.13714795891608
C	5.53093121993510	-2.20230647928798	-1.60873519690087
C	5.61030482196438	-3.61442951264455	-1.01713573261542
N	3.63781762494125	-1.71127420237785	-0.23986074539349
H	5.68795167531087	-4.34781710383881	-1.83992025605144
C	4.33851521650615	-5.38934198829869	0.23592597628668
O	4.89812789020220	-6.28237094423685	-0.40002390537189
O	3.63696573290798	-5.65254791148199	1.37078364055364
C	6.62873174551947	-1.91203299491687	-2.55417103283769
O	7.26562230518913	-2.79121939937529	-3.12983759602765
O	6.94922834044833	-0.59217287434725	-2.66978044782550
C	8.12141805173497	-0.24858024780180	-3.45295990845575
C	7.83791465212140	-0.16378739376424	-4.94950786066004
H	8.91526591177054	-0.99079585279188	-3.24714005787942
H	8.43405577039860	0.73280186077735	-3.05340083907143
H	8.76584986079731	0.13707499051910	-5.47356774710830
H	7.51184037803627	-1.14257281949725	-5.34159580489756
H	7.05366657013172	0.58285171743553	-5.16376589923863
C	3.55433428128766	-7.03381943402829	1.79067855348930
C	2.67376164061376	-7.08841620720393	3.02864050761085
H	4.57846525156982	-7.40459501476155	1.99348021179837
H	3.13759073628503	-7.63757372863897	0.96361177216136
H	2.60552330284940	-8.13307761778493	3.38663651388529
H	3.09576791126569	-6.46597300510856	3.84051467563889
H	1.65395049802098	-6.72930000521966	2.79701563278289
C	2.41250113879908	-3.14435236389441	1.28372188964723
H	1.85181256092919	-4.07446070569683	1.12128080916604
H	2.84147342110382	-3.20442433833397	2.30234989667415

H	1.72046980990799	-2.28831941815415	1.24138241133681
C	4.37736514695150	0.10977049694370	-1.64633838079724
H	4.93368697663027	0.80377970865443	-0.98514225828540
H	4.74546903061019	0.26153334125685	-2.66775092133610
H	3.30888123633751	0.37750237260039	-1.59106677823371
C	6.88980532250806	-3.75550885949585	-0.17742323045879
C	7.83084569701726	-4.77027195300309	-0.43575215824997
C	7.13027618837191	-2.86044793381804	0.88627890646171
C	8.99080849124214	-4.89110031009847	0.35041081229863
H	7.64538605887280	-5.46676898429423	-1.26267171425248
C	8.28759474387394	-2.97489708114889	1.67556964393440
H	6.39983758647884	-2.06624558276281	1.08958690531504
C	9.22317531182080	-3.99303968583264	1.40906268939580
H	9.71689052965449	-5.68669035561429	0.13554425389482
H	8.46135831529480	-2.26960500561672	2.49932101441426
H	10.12882158328919	-4.08561628791028	2.02302722598353

7.6.2. CPA 1a, Z-imine 2a, HE 3b (C102)

EE: -5020.365488465497

P	0.76659880888941	0.31245614168256	-1.93695531746155
O	-0.65266684600910	0.47848796278951	-1.43299515848799
O	1.12724261579841	-0.79959616109864	-2.88970455711048
O	1.72112564803937	0.33086060986924	-0.54252185555393
C	3.08719392205995	0.51245033665949	-0.59325524828668
C	3.57757237966107	1.77848528183399	-0.95901308105698
C	4.98893861707573	1.94164156112132	-1.17828963463236
C	5.55441517176509	3.12735178602317	-1.73693675750318
C	6.92351823551540	3.22822354283793	-1.95437851847680
C	7.79381263802198	2.15347541017596	-1.61345229781593
C	7.27284662349298	0.98567841226919	-1.07537745461648
C	5.86780576856665	0.84537938561406	-0.85918359559568
C	5.32533304278816	-0.35787754144474	-0.33674760997285
C	3.95186600368087	-0.57357799745542	-0.19097090021406
C	3.45370509206358	-1.83499070740840	0.44066709766355
C	2.15141303630743	-1.95148888121960	0.98132236106719
C	1.72810706588189	-3.11945802818918	1.63383650648824
C	2.59705489024667	-4.20273678724824	1.81470470534439
C	3.90224593156588	-4.09457311728807	1.30420766545827
C	4.31404346996295	-2.95246562219637	0.60411397826907
H	5.32561395494281	-2.94146210539131	0.19426221907239
C	4.90083036949112	-5.18635618438858	1.62761655920340
F	5.94831617324221	-5.21556753117909	0.76913456482317
F	5.42331613519325	-4.99866028793218	2.87213045301704
F	4.34148720950501	-6.42055283942385	1.62949161703165
H	2.27269959937268	-5.10758156905667	2.33706159647572
C	0.27737441512890	-3.21342145647902	2.05282318697442
F	0.06209148586712	-4.15261736717724	3.00054194716670
F	-0.51302212698072	-3.53311034500981	0.98753641694704
F	-0.19649287489654	-2.03408915615822	2.53849864803271
H	1.45129959046294	-1.12298021679314	0.89529433219344
H	6.04392447309184	-1.12423620662337	-0.03305969404268
H	7.92596262464818	0.13993290217748	-0.82883018915066
H	8.87273944924308	2.24531978095768	-1.79077733076093
H	7.33731659051417	4.14285767119622	-2.39781567255916
H	4.89157719699064	3.95706799142645	-2.00488958181230
C	2.63272380401625	2.93241300652721	-1.04388732227928
C	1.47967328209203	2.83331038738434	-1.82677231831785
C	0.42003096780914	3.79667333021517	-1.77401656114095

C	0.61902457944899	4.94824724513128	-1.02238649881534
C	1.80228495275840	5.14340765889521	-0.25754323881157
C	1.95671825964304	6.30965496862835	0.55261711552204
C	3.03245443251217	6.43763942690548	1.41900143160688
C	3.98873076987989	5.38802344960929	1.51333650536928
C	3.87719002851169	4.25107993003231	0.72146382604247
C	2.80314912657008	4.10056694233001	-0.20982594480138
H	4.61666440867686	3.45247772920808	0.82325977020973
H	4.82079466865865	5.47189811799889	2.22397307770976
H	3.13610221042164	7.33181285001027	2.04646846290835
H	1.18615291300022	7.08908657381661	0.49515381645170
H	-0.17343084525902	5.70490853904027	-0.97219004528728
C	-0.91275353340085	3.46867855490997	-2.34336260850477
C	-1.08499894600527	2.99757462465550	-3.66004391034036
C	-2.34892464831980	2.57381328542213	-4.09957583384636
C	-3.45849037414710	2.60347573008121	-3.23702541930231
C	-3.28951500811788	3.08627300883678	-1.92973534331079
C	-2.03148491740463	3.52600999656516	-1.48845446956524
H	-1.90314970170517	3.86440220028224	-0.45516617180976
C	-4.43623525066508	3.03394354329205	-0.94295165868169
F	-5.64639184262311	3.04311466741213	-1.54584475101932
F	-4.40834412992551	4.07087565810530	-0.07069447517013
F	-4.38384330791769	1.89391165642286	-0.18898951074351
H	-4.43639068180492	2.24964320387979	-3.57741565832025
C	-2.53127908826930	2.12677268481495	-5.53567475573371
F	-3.51491101074810	1.20300402446667	-5.66530496341670
F	-2.86895682372577	3.17400055954241	-6.33604506830544
F	-1.40191886899741	1.58619128225300	-6.05285840071634
H	-0.22551465481319	2.94255954291150	-4.33332191474064
O	1.32949693868284	1.72540193095098	-2.63985897714438
C	-2.40659370548960	-0.02897361890976	1.44151328553009
N	-1.56735720050626	0.89383435569592	1.01973489468931
C	-1.17580184566869	2.10024429590212	1.67675792950770
C	-2.14811564426483	2.94731738256529	2.24438592263478
C	-1.74271361336665	4.14567485135548	2.84862487011175
C	-0.37872047282136	4.49369084241944	2.89056064308877
C	0.57994137485726	3.64803179453480	2.30655253580325
C	0.18576214674254	2.45286285437536	1.68402862836804
H	0.91852037870099	1.79402003106403	1.20841295311664
H	1.63947844782245	3.92398054992421	2.32128838747916
H	-0.06419986275216	5.43140144361194	3.36505135350009
H	-2.49744019093281	4.81344477525492	3.28250654413581
H	-3.20476462482979	2.66769155508359	2.19909388223790
H	-1.17042208183122	0.76639633929826	0.03502828065659
C	-2.89102403471239	-0.10854101160640	2.81981457499700
C	-2.06369187036018	0.23881238953573	3.91874782951610
C	-2.50778302896301	0.09820869872561	5.23382168688298
C	-3.81977573258141	-0.37936475560469	5.48721450618976
C	-4.65277038436442	-0.74263818381855	4.39740081446985
C	-4.18696616624200	-0.62688935768922	3.09071454965162
H	-4.84487696166928	-0.91335720475252	2.26303661592114
H	-5.66160936209422	-1.11650877698608	4.60688825686789
H	-1.83376164351445	0.35067144449021	6.05759888935481
H	-1.04131079266378	0.58713790166931	3.74374448979827
C	-2.86065968016872	-1.05454214579926	0.44451182505244
H	-2.30653566364592	-0.96597150504640	-0.50292825833586
H	-3.93803453282864	-0.89264072494699	0.24529965081042
H	-2.74837237864379	-2.06674327907101	0.86916634922570
C	-3.56605121806837	-0.17440529195124	7.86425488087076
H	-2.64779389268917	-0.79190299064320	7.91841772497381
H	-3.28565360131520	0.89692703425368	7.83163720330777
H	-4.20449926056351	-0.37100892018671	8.74101834886351

O	-4.34972352295085	-0.52900388845898	6.71585064937469
H	2.59677437454742	-1.71891235945879	-2.96735732053564
C	3.38221577051697	-3.56443908384280	-2.45105418113412
C	4.62399316232951	-1.68266104576323	-3.39829973020344
C	4.50817345372411	-4.36213471968430	-2.42496459708660
C	5.81048307296477	-2.38657686774213	-3.37024558641178
C	5.81002991646235	-3.89533046476771	-3.08522758905041
H	6.65043855499864	-4.13533174453904	-2.41358572172087
N	3.45950421902015	-2.29578278631263	-2.98476421120230
C	7.07998270641948	-1.74036288148941	-3.73985944664591
O	7.22554576129912	-0.63964568932027	-4.26837493851854
O	8.14927802054573	-2.53350755269665	-3.42140233584699
C	4.45749164479484	-5.71587067033052	-1.85689370748253
O	3.47928782008609	-6.27524142534948	-1.36276659988032
O	5.67635457921832	-6.32945722725811	-1.93655419760695
C	4.45267750819503	-0.25383342053760	-3.85007637414473
H	4.67203780822651	-0.17365601471949	-4.93079715177801
H	3.42598356676505	0.09729815468892	-3.65762962112264
H	5.17187637288105	0.40498602568023	-3.33993075322768
C	2.01870751002943	-3.94215225310450	-1.92589872192664
H	2.08286203002529	-4.23375332853577	-0.86573764030676
H	1.31232963677227	-3.10301780814302	-2.03397552868676
H	1.63494733862559	-4.82762450254265	-2.46423755515147
C	9.47151511705196	-2.06657381236102	-3.77271671361550
H	10.07833853715918	-2.98690601942037	-3.84736493876644
H	9.43362621671039	-1.57748495503530	-4.76415430152050
C	5.78872456584554	-7.68511303152949	-1.45129764222350
H	5.20570996994050	-7.78964269370305	-0.51903321757832
H	6.86180549361570	-7.80858236895980	-1.21957722025363
C	10.03716075235208	-1.11605274607641	-2.72059138679980
H	9.44463017512580	-0.18544371574826	-2.68093357694669
H	10.02757816039402	-1.59011106519075	-1.72049830442941
H	11.08312190609801	-0.85943659266760	-2.97713637129108
C	5.33097140240358	-8.69042197879912	-2.50539822775470
H	5.49874096625753	-9.72078514690957	-2.13650683612388
H	4.25337926826221	-8.56337704149472	-2.71591666593192
H	5.89874009987616	-8.55567008476986	-3.44568895669281
C	6.05307543950741	-4.65316874596753	-4.39860734387981
C	5.06917744808568	-4.63761302745352	-5.40915344141406
C	7.25009987304998	-5.35875974936400	-4.62927523104168
C	5.27295898821351	-5.31645596347505	-6.62311534671481
H	4.13362166243910	-4.09008065814588	-5.23498078967241
C	7.46043536867442	-6.04078949694164	-5.84092909165074
H	8.01622646969829	-5.37257444433695	-3.84443057399168
C	6.47138279104468	-6.02220507659665	-6.84272567319395
H	4.49573241639632	-5.29659604452457	-7.39877800696141
H	8.39766180752599	-6.58967985889748	-6.00442490442511
H	6.63349480818017	-6.55503787816174	-7.78901001191998

7.6.3. CPA 1a, Z-imine 2a, HE 3b.

EE: -5020.364277608525

P	-0.04061609093449	0.01109272216164	0.91025799125555
O	-1.37901376316531	0.59803178454550	1.31157001402758
O	1.15450704446366	0.10178917337508	1.82557947427816
O	0.26813345449677	0.63592022791174	-0.62398081645737

C	1.27569293491920	0.08981390638641	-1.39381755241949
C	1.08633423201683	-1.17735354781488	-1.95964113858198
C	2.18909141592329	-1.80164998012856	-2.64820839483675
C	2.13908404191920	-3.14338119217904	-3.13532916440733
C	3.23001506583530	-3.70948888756991	-3.78264893110180
C	4.42696249637282	-2.96482127683576	-3.97565546271782
C	4.51765964155828	-1.66745619031248	-3.49542090347843
C	3.41658619443995	-1.06148199817216	-2.81693114073401
C	3.50571837402137	0.26803273635349	-2.32214309443555
C	2.46240299700189	0.86996984836793	-1.62181712993021
C	2.56369275952653	2.29625662172503	-1.21026857950016
C	1.43740659363899	3.15159176878333	-1.24934460474127
C	1.55270427685855	4.50899105589739	-0.91898749511708
C	2.79607290683675	5.05978764961867	-0.56961683831374
C	3.92338646883176	4.22289365531191	-0.55598205619522
C	3.81142651689130	2.85617027238731	-0.85721953116629
H	4.69662648580153	2.21524857246305	-0.80525582870673
C	5.25461053174304	4.81663966830283	-0.14854127505058
F	5.43884566629148	6.05482991211560	-0.66625984600176
F	5.33894191721935	4.94876712270926	1.20706027988568
F	6.30515330262331	4.05714228260072	-0.53750094226994
H	2.88656245332856	6.12171431078284	-0.31885883952712
C	0.34450918168322	5.42006428914546	-1.00051422702176
F	0.30723806944318	6.29752092377313	0.03378088098938
F	-0.82526219917235	4.73786747889523	-0.99074575693760
F	0.36140062823893	6.16205770947173	-2.14016178439117
H	0.46613793832314	2.75922411719862	-1.55424530199728
H	4.42146227890711	0.83490562863152	-2.52546159240724
H	5.44282878769524	-1.08940326219899	-3.61010963360886
H	5.28200882703785	-3.42657472058595	-4.48368891654537
H	3.17208319934878	-4.74627346900231	-4.13765023694710
H	1.22970969592443	-3.73278361004466	-2.98147571194202
C	-0.25058062408054	-1.83671097937893	-1.86663495471175
C	-0.86736268818517	-2.01645862878082	-0.62170930664340
C	-2.16380384287361	-2.62479555903283	-0.47336953682000
C	-2.77727164960539	-3.10365144886655	-1.63158511607427
C	-2.21729810807056	-2.91997188859960	-2.92188656984789
C	-2.91822742006523	-3.35291470196407	-4.08930178331849
C	-2.42554022168787	-3.07656353684479	-5.35549844857041
C	-1.21335737376797	-2.34296457035952	-5.49633313631935
C	-0.49773652613835	-1.93209889061088	-4.37875777898039
C	-0.95869591631180	-2.22908474115856	-3.05919917017118
H	0.42820889051183	-1.36390543690516	-4.50746137225989
H	-0.84149106920926	-2.09472345285543	-6.49864927619527
H	-2.97583451923069	-3.40089151414861	-6.24771394893726
H	-3.86985728155586	-3.88389301479054	-3.95914720671149
H	-3.74123472088326	-3.62009331139786	-1.56405075061804
C	-2.88928002184074	-2.68149621907025	0.82639944865227
C	-2.23952738535095	-2.76368482999212	2.07851636903252
C	-2.97534877927977	-2.74069307653328	3.27439226548750
C	-4.37516695363677	-2.67521522240660	3.26475337194597
C	-5.03283145765430	-2.62406541343727	2.02455355344273
C	-4.30402641060654	-2.61699790851577	0.82779143950749
H	-4.84331586670647	-2.52817823830111	-0.11829933850188
C	-6.54649839361795	-2.61146877661759	1.98588009735318
F	-7.07798057518514	-1.96623288438784	3.05814950930175
F	-7.05430142603309	-3.86935846119312	1.99659654079987
F	-7.03331628981599	-2.00143286722503	0.87447727182969
H	-4.94169176894989	-2.64729584940852	4.20003554360779
C	-2.21780959688116	-2.66624056300335	4.58327679934940
F	-1.79816294037120	-1.39081519377216	4.81734432509007
F	-2.96991470293075	-3.03535965978950	5.64605122430194

F	-1.10969662587843	-3.44597229515652	4.58062925839847
H	-1.15266942666324	-2.83474140313480	2.12795858391118
O	-0.18713822185932	-1.61350189739340	0.50613358972425
C	-4.77292765368859	0.94019471845842	1.20087694235130
N	-3.84677595413564	0.60435362857651	0.32309771342339
C	-3.94511443105028	0.37209376690726	-1.07884429247918
C	-2.83506277572862	0.72405660150653	-1.86976943291843
C	-2.85901262312469	0.46516122221832	-3.24900077576602
C	-3.97796329264177	-0.14959080158655	-3.83390175280394
C	-5.07362344132426	-0.51996773684373	-3.03049207746451
C	-5.06381293997363	-0.26639500870216	-1.65282007928089
H	-5.90798025595263	-0.56403823571655	-1.02476255419066
H	-5.93815633815021	-1.02601303255054	-3.47760795419587
H	-3.98765442391392	-0.36383219333355	-4.90857182593754
H	-1.99203245259123	0.73389557992414	-3.86311801832252
H	-1.96334670278498	1.18874901425005	-1.39956370793705
H	-2.85596921130988	0.49772396029241	0.70489462514051
C	-6.09966684646780	1.43468721534309	0.83548283870659
C	-6.28934014318372	2.25224388644660	-0.30960296052857
C	-7.53873609804920	2.78847219638167	-0.62115769642527
C	-8.65517362185949	2.49899079148957	0.20664675192218
C	-8.47657627997507	1.69422124663497	1.36168017202260
C	-7.21915982770856	1.18957211897952	1.67796292347203
H	-7.10943307654101	0.56360582921148	2.56815915887411
H	-9.34538949861284	1.48084895519138	1.99488786264164
H	-7.64143513174830	3.43399105288255	-1.49843482570221
H	-5.43757672273008	2.49844114597474	-0.95169120669699
C	-4.40152300707067	0.83989575600884	2.65316347462819
H	-5.05699054845626	0.09094781326129	3.13537426560620
H	-4.59178065755871	1.80501941619145	3.15807783016270
H	-3.35381965864304	0.52851020146261	2.78823402339512
C	-10.16372365569214	3.76324428787599	-1.16774657715860
H	-9.91628709041506	3.21751991132102	-2.09951189731029
H	-11.24122532118015	3.99376043132059	-1.14039486593187
H	-9.57490191002265	4.70053804157178	-1.12374967403648
O	-9.90443945919933	2.94618321249306	-0.01687287704963
H	2.86506093205158	-0.40544698394970	1.53650485681809
C	4.74162556903199	0.47197971088002	1.44685530853536
C	4.13680977764281	-1.74598264581772	0.60406792315827
C	6.02132769531144	0.36670173475359	0.94934539236710
C	5.37849815243046	-1.92917737992701	0.03566360128918
C	6.50566450624962	-0.91485986108466	0.25547274952623
H	6.96050114808068	-0.64038807712946	-0.71358649812420
N	3.85402523914555	-0.57687985720833	1.28094151973638
C	5.68630821354997	-3.15215225550230	-0.72106755332105
O	5.04686780262047	-4.20218941071801	-0.72992550831452
O	6.83006627469043	-2.99682526592297	-1.45676458267001
C	7.07031262353939	1.40167791049396	1.05378142586610
O	8.03361824222979	1.46386284380756	0.29335997286655
O	6.92865241665185	2.25133435717750	2.10521818042642
C	7.95096476873436	3.26368150000213	2.27436140832988
C	7.61311953438654	4.06097505654404	3.52400219883359
H	7.97699147358955	3.90131722155777	1.37224998005395
H	8.93398711279901	2.76085243874340	2.35982536776312
H	7.56631898200936	3.40076006896572	4.41092195360465
H	8.39701764606895	4.82275527476915	3.69551127168153
H	6.64249114066916	4.57666877081579	3.40992740001374
C	7.34075298768748	-4.14789115927878	-2.16301455079318
C	8.14132243577518	-5.05833720160484	-1.23405848357078
H	7.97915428773732	-3.72535591027797	-2.95936993714865
H	6.50057361029274	-4.69444089139405	-2.62909082730600
H	8.57584799594239	-5.89472569556463	-1.81501294912490

H	8.96243303887932	-4.49725978221098	-0.74975543998197
H	7.48602041850340	-5.47869487083418	-0.44944982886060
C	3.00224014802259	-2.73861761212341	0.57021100052307
H	3.26379238227676	-3.62328164028571	1.18027933238361
H	2.07581785848171	-2.28720387317609	0.95550916025404
H	2.83112010952473	-3.10481423161194	-0.45358412684928
C	4.15106658670699	1.64660701941285	2.19187928946400
H	3.05632524613438	1.66737228693267	2.05480479985257
H	4.36157060758132	1.54508367369240	3.27512849840135
H	4.58510069419983	2.59879659946360	1.86276879463850
C	7.61535683873813	-1.56130538330023	1.10002084694753
C	8.93131302234471	-1.68685587256379	0.61650225327190
C	7.31723920986251	-2.04368818353719	2.39119397106210
C	9.93104267966678	-2.28741936214202	1.40192484508934
H	9.16491839683873	-1.31184128440410	-0.38747089253811
C	8.31123685164856	-2.64568413882420	3.18229439686005
H	6.29189551744551	-1.94709436536658	2.77239898969381
C	9.62359389103945	-2.77057480624201	2.68804408461342
H	10.95263559162890	-2.38100951314833	1.00968822686345
H	8.06266625962456	-3.01868078974382	4.18490517876475
H	10.40266572194323	-3.24154102559819	3.30198605883718

[3:3] Dimer D2

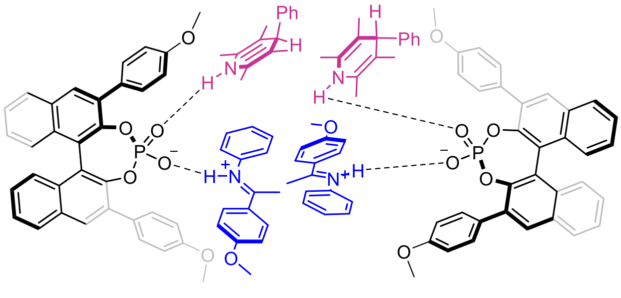

D2: Head-to-Head [3:3] dimer-2

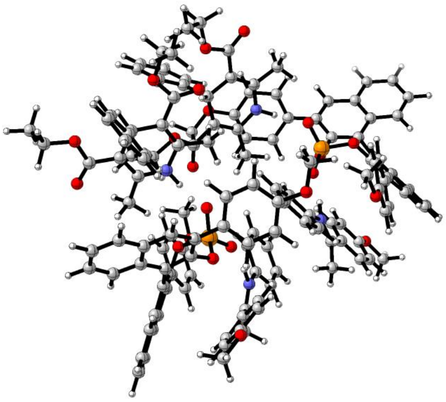

EE: -462.450107929237

P	-3.20768869455404	-0.44457024003639	-0.25944261711222
O	-3.51561049899953	-1.20388273234023	0.97950842449943
O	-1.83022378378187	0.09757925249314	-0.47024386385916
O	-4.32314000373492	0.75222784055231	-0.35811913326641
C	-4.59659560534804	1.31452761780495	-1.58222873452993
C	-5.30609019668636	0.57531283256159	-2.50953798303814
C	-5.57785062961360	1.11445143980865	-3.79612496283975
C	-6.16766904986436	0.35880509961305	-4.83178061856286
C	-6.45017395750616	0.93010832361588	-6.04158206635658
C	-6.15450699340284	2.28147311908644	-6.27823358008938
C	-5.54016479993401	3.02652301075519	-5.31145664332096
C	-5.22000184604298	2.46140070931542	-4.05790711073878
C	-4.54637264240728	3.19710335333445	-3.06759323875421
C	-4.19258560158621	2.65099104987713	-1.85414035031719
C	-3.41302578245886	3.44643394822985	-0.89304653047193
C	-3.59834409608976	3.35712673573224	0.48342385636027
C	-2.82945329911373	4.09464100935805	1.36712685799428
C	-1.86173339180249	4.97127836473666	0.89112507104835
C	-1.69885974318482	5.10271549664481	-0.48977735236915
C	-2.45027419957110	4.34659334571050	-1.35979021852459
H	-2.28526124933148	4.44330783666979	-2.42198452570064
H	-4.36059334904736	2.70615749198532	0.88180311855235

H	-4.31040073698821	4.23083827038406	-3.27284469529198
H	-5.28446920471114	4.06169574714836	-5.48925210653683
H	-6.40545329399963	2.72043758722806	-7.23315820630285
H	-6.90388895020329	0.33980045083105	-6.82401277526232
H	-6.39140710864753	-0.68299733718157	-4.65760613556940
C	-5.77647374698624	-0.77591870309478	-2.13742675644600
C	-4.85762915693118	-1.70335023083476	-1.69394159518762
C	-5.24333450851875	-3.00692543973036	-1.30021659751434
C	-6.56944635476739	-3.35257071115825	-1.40514329004223
C	-7.54644282887270	-2.43480847882571	-1.83348709015412
C	-8.91160207072187	-2.78902756854196	-1.89454691873783
C	-9.85946384024270	-1.86968328840921	-2.24553858647787
C	-9.48187506214165	-0.54935387347138	-2.53308497961127
C	-8.16581265687049	-0.17890957519351	-2.49849848561949
C	-7.15775697432806	-1.11355823601272	-2.17826155382404
H	-7.88828931941917	0.84327360937612	-2.70731152041059
H	-10.24118045826892	0.17891652562337	-2.77838526415442
H	-10.90254420667622	-2.14751198692041	-2.28777796109812
H	-9.19131377868471	-3.80274209805264	-1.64472962427438
H	-6.87830598617781	-4.35245693818505	-1.13500277865414
C	-4.26803527087967	-3.95973446221751	-0.74939482697627
C	-3.03991515255278	-4.20111463824322	-1.35152393688059
C	-2.14686402874300	-5.11722054887570	-0.82024857690974
C	-2.46896164690658	-5.81367086560206	0.33941694500727
C	-3.69910741827862	-5.56965502170386	0.95417622877239
C	-4.58106546546833	-4.66041894281116	0.41653769645793
H	-5.51878296135861	-4.46808527559973	0.91688046111499
H	-2.77784148089940	-3.67569197051953	-2.25796832498757
O	-3.53507225868766	-1.32516035634445	-1.60691786497225
C	0.17821450438353	2.44662632207116	-2.36949805963180
N	-0.02904523162908	1.98568561113446	-1.16852447753117
C	0.64136475028296	2.25047108477277	0.03823117949869
C	1.21758770818982	3.47510914565066	0.37114493143237
C	1.83441614949253	3.63129602496085	1.60089182281694
C	1.87795038345678	2.58880336869162	2.51001199963112
C	1.27807462100485	1.37929186828157	2.19405050400206
C	0.66078090281505	1.21234525430712	0.97145291573523
H	0.17489649657490	0.28233074184976	0.72682117165486
H	1.29387163432506	0.56311769615936	2.90002088417110
H	2.36886813764365	2.71462794292094	3.46220475143663
H	2.26901450012231	4.58514068439877	1.85667522455921
H	1.13864688649090	4.32690202490359	-0.28060071186823
H	-0.78218288761771	1.27979578515094	-1.02287714799169
C	-0.65124458619956	2.00831569400808	-3.47151558622607
C	-1.31461177895809	0.76858119519397	-3.46568173961895
C	-2.04911910235370	0.35655260251136	-4.54763463308762
C	-2.18421051343135	1.16769805086599	-5.68396280053264
C	-1.55701133384349	2.41761891789743	-5.68728585428441
C	-0.79839869353809	2.81571326451732	-4.61005536110482
H	-0.34232747639210	3.79247398435034	-4.64187042409340
H	-1.65922884195328	3.08183043234520	-6.53008700071369
H	-2.54293237097420	-0.60237930010789	-4.54372990054899
H	-1.22764032815445	0.10679299447952	-2.62038460556975
C	1.29339650074627	3.38694998290420	-2.66572696030445
H	0.95354553017930	4.41941469278496	-2.59150795143068
H	1.65690765773732	3.22349228267763	-3.67609455795238
H	2.10513175325566	3.24467742766159	-1.95745850787380
C	-3.04287000531432	1.40522266067359	-7.89107094079479
H	-3.54473976209533	2.35821283773285	-7.70932703616077
H	-2.07147918937631	1.57671366863402	-8.36287957698880
H	-3.66089138171094	0.79109936426551	-8.54321871849933
O	-2.90393343666982	0.66104075773457	-6.69418966103237

H	-3.00322992361640	3.97920167876387	2.42556143271625
H	-0.97261205546016	5.81455755293283	-0.85040011811886
H	-3.93803629050531	-6.10741626752005	1.85912123335779
H	-1.20706511432863	-5.28519218319912	-1.32413835773214
O	-1.03880510249784	5.73474509636945	1.65975522745949
O	-1.67905864916379	-6.74568298598183	0.94442994856922
C	-1.09088192320519	5.59545639800549	3.06245363564355
H	-0.90662448832353	4.56121630300172	3.36862512828460
H	-0.29541093768123	6.23174368777807	3.44680229365742
H	-2.05324435951632	5.93002037539097	3.46240318752208
C	-0.38160453780477	-6.97451632166896	0.44242827176096
H	0.04392241777172	-7.74888999674133	1.07877192105330
H	-0.40756800015089	-7.32853372891453	-0.59279684696301
H	0.23858752949697	-6.07578995972869	0.51379270778785
H	4.59748598510203	-4.35015516987518	0.41147110702619
C	5.11007802226670	-3.65135387951873	-0.22842246872680
C	6.09302093524203	-2.84570457881645	0.31457260898245
C	4.78156296276479	-3.53830721396589	-1.57491766807172
C	6.77416993236480	-1.91190612577795	-0.45965541812602
H	6.34070623691064	-2.95635381232708	1.35917747336117
C	5.49434138891730	-2.64044880703156	-2.37221118235215
O	3.80263469714975	-4.24836744748896	-2.20006168221218
C	7.78590270296402	-1.02541620908128	0.13392207312411
C	6.47580771005504	-1.84670103251523	-1.82085024042682
H	5.24685651319861	-2.58224202350033	-3.42108922290377
C	3.00860535825327	-5.12145155084889	-1.42445182345500
C	7.55977783764562	-0.41827482809546	1.39857074996897
C	8.97453310010093	-0.77308224776277	-0.50806327101833
H	7.01510596999969	-1.15326211607028	-2.44718824047361
H	2.56948478950159	-4.60966930001762	-0.56347269001875
H	2.22018143904731	-5.46812984753448	-2.09097614552401
H	3.58944202765741	-5.98080595750427	-1.07382981974187
O	6.38294929941712	-0.65867603215172	2.05980999497566
C	8.52286688174352	0.36275891765758	2.00421400073161
C	9.95471965803204	0.07422370394397	0.04273105760085
H	9.17702671998791	-1.25248562143849	-1.45502576609095
P	5.28503022529461	0.55062396438200	2.26902937191134
C	9.73141988849601	0.65721256510143	1.31737679716503
C	8.26440498492794	0.88353573754301	3.36369415121066
C	11.15852390693556	0.35486649337945	-0.63941362948832
O	4.52333203638969	0.17436632189710	3.48775527144166
O	4.56570891432335	0.83955442422180	0.99360069021140
O	6.26373671738454	1.84758284555724	2.52947265697660
C	10.70927980345569	1.52918050379460	1.84195328683158
C	7.14024349917034	1.65252854369982	3.57542622059511
C	9.11003820159257	0.55022637485261	4.45840293615190
C	12.09609845362574	1.18716956560062	-0.09512024758746
H	11.32458826938036	-0.10496647798201	-1.60320381646652
C	11.86395985093404	1.78458180725334	1.15344533522605
H	10.53290085106877	1.99344841698705	2.80112597779716
C	6.85476238300422	2.21345033020436	4.84414314024516
C	8.80986849775066	1.08103471118131	5.74075522061197
C	10.19317053285374	-0.34696154286413	4.33771311708436
H	13.01720865888302	1.39346530024472	-0.62012283026880
H	12.60659605306704	2.44964726366598	1.57061911207984
C	7.69651578065592	1.93007905436915	5.89086601416208
C	5.68236733578176	3.08349411128415	5.02583241647446
C	9.62288048389192	0.72843579925701	6.83893564685215
C	10.95541254801440	-0.67751446423300	5.42426364745931
H	10.40372150663523	-0.79137803254183	3.37656439332629
H	7.49897400044079	2.36260264774811	6.86077910467104
C	5.46055171285787	4.16334656161493	4.17323389420436

C	4.78347527786553	2.86513696559184	6.06163977663248
C	10.67468796161087	-0.13023355017593	6.68479845928358
H	9.38698786705273	1.13845744875094	7.81022856131365
H	11.77418057159827	-1.37386493535926	5.31771706848885
C	4.36792023206807	4.98412011092064	4.34452504300909
H	6.16918381082178	4.36169000461608	3.38447388246371
C	3.67447194515724	3.67520575006919	6.23461048012847
H	4.93254187183834	2.03599917747136	6.73688857103023
H	11.28599953786821	-0.40314571000954	7.53245436673248
C	3.45193905805127	4.74167511499579	5.37059704253236
H	4.19744846104947	5.82968443477068	3.69583405504754
H	2.99355593170079	3.45781115947210	7.04272104756303
O	2.39113436781512	5.59849254716833	5.43884608717025
C	1.44044411638923	5.41366002733209	6.46471109939175
H	0.71075175583609	6.21156440247124	6.33621177677921
H	0.93864771912196	4.44483589442788	6.37654299111028
H	1.89801802019979	5.49839379726058	7.45515500012901
H	5.93816326838492	4.86932104500524	1.12378917620987
C	6.47667728791569	4.23145746269254	0.44199780259464
C	5.80900492039863	3.33170472976737	-0.34532433096319
C	7.87471058755301	4.33212633128068	0.39269102065487
C	6.50166706767719	2.52746416548021	-1.26944439975742
H	4.73436216528504	3.27539330701981	-0.27689548052387
C	8.57270794370841	3.53365331318456	-0.51705843101776
O	8.43354732260635	5.21648917234457	1.23423349078540
C	5.75413618662143	1.64112406603127	-2.13556057320709
C	7.89273897335220	2.66287591127063	-1.33902105253633
H	9.64748202918068	3.57925376027772	-0.58033776754187
C	9.83824697300613	5.39132603378840	1.22803324370675
N	4.66932988481022	1.09890215100042	-1.64964410819406
C	6.21167591017628	1.42833939520376	-3.53049918331018
H	8.46998670604329	2.04112797728174	-2.00483469067258
H	10.35463227943155	4.46260336634544	1.48300216453254
H	10.18613319989533	5.75514563862472	0.25743821188062
H	10.04030271112185	6.14120040641290	1.99093079018964
C	3.66211873486279	0.35509614975801	-2.27830392400309
H	4.58909988153770	1.09181490031485	-0.60245622454712
H	7.29788212302746	1.41103866444117	-3.56661060396721
H	5.89868906069548	2.26751096890231	-4.15337745861370
H	5.81325055048659	0.50672963274070	-3.94582356385703
C	2.96382505678319	-0.55813221908825	-1.48660039701192
C	3.28111815585928	0.54377682917389	-3.60678494008799
C	1.94123409514890	-1.30835103472113	-2.03311073501434
H	3.23806767445330	-0.66906909731184	-0.44811669191036
C	2.25669311825812	-0.21645726982033	-4.14304208379858
H	3.74475601440957	1.30244964113236	-4.21356990746756
C	1.59684745644695	-1.15420717244155	-3.36733901422353
H	1.40855591766255	-2.01346616710633	-1.41555667288581
H	1.96682849128833	-0.06908369344114	-5.17223682151309
H	0.80398687997663	-1.74989558140172	-3.79196328755344
C	-0.64166923976394	-3.01747758270342	2.39915098524596
C	-2.50001496521102	-2.96116648760017	3.92543026155213
C	0.20193664683097	-3.49739271968404	3.35044601594680
C	-1.73803721860119	-3.41773456347736	4.95391368965443
C	-0.28777178471108	-3.82233891333167	4.75152130368513
H	0.33716660201014	-3.29580378343209	5.48377776233425
C	-0.24942706040014	-2.67172335671785	1.00132494462168
H	0.78315310396203	-2.33575846841184	0.96874500865415
H	-0.33805764845052	-3.55436561708351	0.36920120550087
H	-0.91468001974136	-1.90738307045113	0.60393225800850
C	-3.94252473079653	-2.59266947822748	4.01819638204845
H	-4.13237493722350	-2.00026802587202	4.90949747471877

H	-4.24251189398161	-2.04081272055358	3.13028153431256
H	-4.54809722923604	-3.49382667471807	4.10423529153361
N	-1.95700261993036	-2.79831036192015	2.68336481570763
H	-2.52392249824608	-2.31468760762023	1.98227316387593
C	-0.14240057709580	-5.31004657916058	5.00665867551829
C	-0.88020774666851	-6.21351867930730	4.25327952964653
C	0.71061526591871	-5.79535593346179	5.98808574764120
C	-0.77038008757601	-7.57459537018281	4.47725451444041
H	-1.54484981437721	-5.84650355733721	3.48544398707351
C	0.82228327767329	-7.15607520239250	6.21432444921498
H	1.29048815632145	-5.09843184100937	6.57548221821700
C	0.08143534693564	-8.05056924692406	5.45967932523438
H	-1.35378041719066	-8.26278805193981	3.88210230213251
H	1.48939278397051	-7.52058976119263	6.98362510082567
H	0.16672582807620	-9.11321188784146	5.63810152899007
C	1.61180456929720	-3.73128630728996	3.08127966873728
O	2.13714600070431	-3.77591267568903	1.97708028717051
O	2.32210244823741	-3.90123534186197	4.21531152472555
C	-2.27161171036816	-3.55385025020979	6.29983479704110
O	-3.43752725394687	-3.40653050550119	6.63534122430813
O	-1.30679002462764	-3.86131276168881	7.19230576687328
C	-1.67715119596029	-4.00284390645565	8.56066984707253
C	-0.57725510125361	-3.42917269887338	9.43937954355317
H	-1.82119773441134	-5.07165615995493	8.76200411624788
H	-2.62899942132412	-3.48398560517185	8.72346756199400
H	-0.86893043035007	-3.50592011584800	10.48319701389343
H	0.35011398574046	-3.97530449730193	9.28573627149527
H	-0.40637212988793	-2.38470915633553	9.18809427640253
C	3.71792990233241	-4.14983884742059	4.08530628760656
C	4.01209145181474	-5.60147628353333	3.72558486454011
H	4.13478938594037	-3.90415467875542	5.06449888937398
H	4.12943111821226	-3.47217241538707	3.32819282052233
H	5.08730352294290	-5.74818313867580	3.66701412871829
H	3.59866633099398	-6.26523499646846	4.48118349193677
H	3.56336407970862	-5.84630012453661	2.76573887306684
H	4.83272591710012	-0.72812202633810	5.01565258405170
C	6.04342858873352	-1.83786529940964	6.26871328400047
C	3.90711468402120	-0.89246015265960	6.83661035430673
C	6.23744721566587	-2.29375569746338	7.53515237725285
C	4.00688761824093	-1.30256347688180	8.12910365941155
C	5.29191045256190	-1.91579482897972	8.66117492155693
N	4.94131097472989	-1.09302353279689	5.96652016276455
C	2.70593429747585	-0.25434607506711	6.22302069252069
H	2.97313113409986	0.19618229082558	5.26999615531123
H	1.93983826139467	-1.01015831189071	6.04973631107597
H	2.27567422205260	0.48682864731714	6.89014413203001
C	6.94736518953068	-2.13356256838020	5.11983479457779
H	6.58516037212117	-1.64724436130657	4.21841345427272
H	7.95931231319388	-1.80008503773462	5.33746313352051
H	7.00433022903521	-3.20818083464271	4.95362377458824
C	2.93172341240574	-1.10392906105645	9.08870946188060
O	1.87284044592950	-0.52942348276870	8.88507041259348
O	3.23050624075509	-1.63928599790248	10.29331740034123
C	7.37736099468018	-3.12898118448960	7.87440065904751
O	8.27017284700645	-3.47720078759532	7.11735173602541
O	7.36100485811685	-3.50050040334863	9.17461704360619
H	5.04673896799187	-2.81575896181232	9.23701196116275
C	5.97008067179972	-0.93761300160472	9.60045570929532
C	6.32768745058777	0.32333610418914	9.14201866961715
C	6.23509600000470	-1.26829102082645	10.92180708498979
C	6.92327316595239	1.24064895293931	9.98939544738027
H	6.13595664745418	0.58051296812066	8.11031079664367

C	6.84188837345259	-0.35728137786255	11.76877694838342
H	5.95880777726607	-2.24621166797579	11.28667816512070
C	7.18318050615686	0.90264199621201	11.30672224517169
H	7.18315948739443	2.22522261024997	9.62505916583559
H	7.04367238746385	-0.62823429552490	12.79591009752491
H	7.64875607593355	1.61811738988146	11.96950086975898
C	2.30999128598395	-1.43111753962767	11.36073561649313
H	1.29653246079546	-1.36586961678994	10.95143041721956
H	2.39857941325769	-2.31725004207684	11.99385498809554
C	8.42321402835937	-4.32899890825305	9.63859415499214
H	8.55414231110500	-4.06133530175352	10.69013650756036
H	9.33221057637180	-4.08827190578773	9.07624007827933
C	2.64425871761576	-0.16712802122660	12.14379081029136
H	2.54778985740767	0.70433343170303	11.49979814962162
H	1.95833590751332	-0.06711751781197	12.98037266796374
H	3.66459230714792	-0.21362481372971	12.51613553660474
C	8.08737411095566	-5.80818331901528	9.49027354969729
H	7.97303892855646	-6.05889376026190	8.43786642301286
H	8.88938323694403	-6.40760504956227	9.91182513030120
H	7.15883684224663	-6.03570446300840	10.00903724705916

[3:3] Dimer – D3

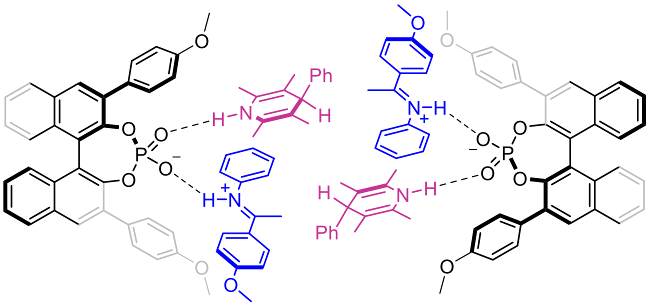

D3: Head-to-Tail [3:3] dimer

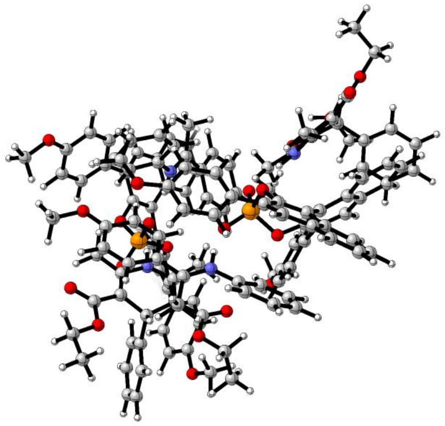

EE: -462.444116437242

P	-2.28580982236741	-2.26553630744663	-3.42754302668606
O	-2.87882715031127	-3.47834496037723	-2.82295830982531
O	-1.75748817642659	-1.18686606428450	-2.51896675167394
O	-3.40622401585042	-1.64585243320779	-4.45112622609850
C	-3.03710493884534	-0.72796581000228	-5.40249116092038
C	-2.26090551280479	-1.13630939967865	-6.46904056995661
C	-1.82946708850612	-0.18714811956050	-7.43876845385490
C	-0.91436322110217	-0.50540817272046	-8.46437486653024
C	-0.57485633790743	0.42056248562719	-9.41247963261015
C	-1.11951808877172	1.71253140584006	-9.37843617809514
C	-1.96841058668974	2.06630614949190	-8.36787155443314
C	-2.32904035018149	1.13756049624488	-7.36795596868749
C	-3.18032374675822	1.49286679180038	-6.30599903020165
C	-3.51729939447601	0.60840920898000	-5.30796788320266
C	-4.36533208745997	1.05282176169093	-4.19151785097950
C	-5.35375607018773	0.24233368643791	-3.64197209356673
C	-6.12101834198340	0.66575117737443	-2.57062116854105
C	-5.92222315696432	1.92839546548198	-2.02344842057944
C	-4.96523795648027	2.76812875368005	-2.59763928190633
C	-4.20393267696668	2.33462026621879	-3.65905592147051
H	-3.45669505297646	2.99457048821746	-4.07168584406374
H	-5.53399453379437	-0.73742723415381	-4.05602081723663

H	-3.58956633799644	2.49253618380186	-6.28880705797646
H	-2.38061155914723	3.06352009947098	-8.31700810228941
H	-0.85385584960684	2.42441124573677	-10.14623481755605
H	0.11450611597544	0.15461475088859	-10.20100601011301
H	-0.47783642550922	-1.49227017373853	-8.49210931395356
C	-1.91772054575948	-2.56281025837414	-6.63091328423210
C	-1.30807290794732	-3.25801452629344	-5.60604096280313
C	-0.92397992009975	-4.61606221557269	-5.76411531808864
C	-1.10508715169172	-5.20348630433602	-6.99476698876830
C	-1.75942127411966	-4.54683649662922	-8.05158133224864
C	-1.98614704574140	-5.18540118161600	-9.28957811528045
C	-2.70439449032965	-4.56706481209518	-10.27389529364897
C	-3.24880000768183	-3.29472229743037	-10.04592983698941
C	-3.01987184036580	-2.63906451499280	-8.86707690485082
C	-2.23278269879953	-3.22528335856669	-7.85289372782398
H	-3.45674091286931	-1.66592005179270	-8.70071400764460
H	-3.85659407521736	-2.83224723718111	-10.80985832174912
H	-2.87432349800952	-5.05963702290579	-11.22006109942184
H	-1.58918139686108	-6.17992138410522	-9.43491682496096
H	-0.75471364306148	-6.21302891060667	-7.15474312839480
C	-0.37306940310328	-5.42377174488570	-4.66686006039403
C	0.58102371472468	-4.95297545205520	-3.77403070027226
C	1.09159658005092	-5.76139589186779	-2.77304576178588
C	0.66280859088034	-7.07746858396510	-2.65399403690889
C	-0.30056661324108	-7.55855423218040	-3.54244969374474
C	-0.81261507492326	-6.74271057227465	-4.52379217810803
H	-1.57933513540778	-7.12526214716192	-5.18223379720843
H	0.94950686875505	-3.94358572506010	-3.86624116751768
O	-1.03359999523454	-2.60021987665589	-4.43180781167527
C	-0.44785520828977	2.05691486587160	-3.43440311252235
N	-0.97503732144378	1.40562851080459	-2.43083895812063
C	-1.44870138730136	1.97161493719234	-1.23308769759708
C	-0.70293024989566	2.92137962940367	-0.53789595950864
C	-1.23540069208469	3.51409354541960	0.59280510950411
C	-2.49736952873035	3.15812984444659	1.03967690608188
C	-3.20285527773694	2.15487761455858	0.39288193921156
C	-2.67858212847892	1.55127228439034	-0.73587029319086
H	-3.21264763743519	0.76959959337648	-1.25289500819720
H	-4.16431212493318	1.84549886013340	0.76919058542502
H	-2.92802416809598	3.64868529709693	1.89921790450078
H	-0.65963492155728	4.26300573624302	1.11506067500005
H	0.29572977625567	3.16789119031244	-0.86177804793553
H	-1.17151138989721	0.38623083623681	-2.52502177687771
C	0.19547374956922	1.35082646402182	-4.51020465603649
C	0.64116364982004	0.02641028626096	-4.33476353059693
C	1.41025846292102	-0.59229244871588	-5.28353766162191
C	1.79751160266205	0.08165475072746	-6.45075979069162
C	1.31218357391270	1.37533354819227	-6.66664954270055
C	0.52886157241281	1.98644485656407	-5.71953825844546
H	0.17102091008209	2.97990978650767	-5.93394049336259
H	1.55879523568930	1.91193080449904	-7.56637923115196
H	1.75749097849196	-1.60045929365254	-5.12651549895965
H	0.42454904549414	-0.50600775220175	-3.42425231191287
C	-0.47917694443559	3.54579486476021	-3.46774266121514
H	-1.12552332342305	3.94923490016306	-2.69393431130171
H	-0.83600854582929	3.88237752424677	-4.43837123057744
H	0.52512110448848	3.94753797615785	-3.33630132690438
C	3.23072730331085	0.14991225698301	-8.33894003264006
H	2.49569356552148	0.41211255843857	-9.10295513827751
H	3.71561490245396	1.05304435476976	-7.96052162054585
H	3.97220072731990	-0.52436691846231	-8.76391302280829
O	2.62833660304501	-0.56933963303529	-7.27358110967513

H	-6.86859896557441	-0.00368263021166	-2.17525224755363
H	-4.82790074216412	3.75306984210347	-2.17925859920299
H	-0.63411662027188	-8.57934928730340	-3.43435669829828
H	1.82565938086468	-5.35325001863088	-2.09753519770927
O	-6.58535834509670	2.42995656724483	-0.94609858482894
O	1.11297361071996	-7.97445539030412	-1.73194144632169
C	-7.50183334155578	1.59907216933050	-0.26574518316524
H	-7.02829159766136	0.67158396398448	0.06988216888624
H	-7.83090947460192	2.17561499373542	0.59741154547917
H	-8.36620678280642	1.35899692644999	-0.89183070981749
C	2.04939657002818	-7.54317357336086	-0.77444023957368
H	2.23255836238508	-8.40822487174975	-0.13909278311083
H	2.98680961640286	-7.22581900107584	-1.24425407983706
H	1.65133870538796	-6.72292275585168	-0.17059768936566
H	-0.11465389504381	3.41418094278267	3.64238658959839
C	0.51116323274413	2.76179883502662	4.22914994161317
C	1.84973520451607	2.64084830472034	3.89280275075070
C	-0.01053074843494	2.05456929947213	5.30595571858935
C	2.70434296935088	1.81139458142744	4.61084433973401
H	2.23363294402549	3.21125774910320	3.06148134885907
C	0.84286696124627	1.23590650987789	6.04701122740542
O	-1.30589584209738	2.08329330068421	5.72348721170580
C	4.12469002950219	1.60960659650842	4.28149188149527
C	2.16883542260539	1.11952450314429	5.70222429759107
H	0.43449183319305	0.70097719384895	6.89048923902243
C	-2.23067755671533	2.88624911907281	5.02403063365888
C	4.60512709493561	1.51977013834009	2.94568662225253
C	5.03194733239046	1.43253577363243	5.30161648166111
H	2.80705480887275	0.47120947823439	6.28144850320622
H	-2.34065892399244	2.55005012733177	3.98958430001506
H	-3.17599814812748	2.76497843459504	5.55066051532005
H	-1.93958310132593	3.94090944315217	5.04219403886325
O	3.77792457226282	1.81734200769903	1.89203742128034
C	5.91436024160146	1.17839946200065	2.67353140496672
C	6.36464656642654	1.05135892928068	5.06947567642477
H	4.71465903580347	1.57058023944648	6.32467110231530
P	3.26403166893458	0.61473449785330	0.89483512659887
C	6.80747599449492	0.86593277381394	3.73597140170480
C	6.38835450370011	1.08840805037084	1.27678307826352
C	7.25514951113454	0.79949352219697	6.13477158328582
O	2.97569967367286	1.28380951887132	-0.40463038918277
O	2.21953424580414	-0.19680197913627	1.59303990485896
O	4.57733670918019	-0.36069022301230	0.79185845365126
C	8.10295657063315	0.34944496700405	3.52393930817269
C	5.74439798377534	0.24787763004576	0.39199194709048
C	7.52115196781325	1.83432328823036	0.84764019342166
C	8.51654473363056	0.33246776620202	5.89515694422635
H	6.91184111139956	0.97134561516989	7.14445953377947
C	8.93355239002085	0.08691015168126	4.57868307812956
H	8.43249387344687	0.15051655597021	2.51544141721969
C	6.27346666712232	-0.01785384796956	-0.89830971706583
C	8.04286882479996	1.59435203426171	-0.44824382833568
C	8.11235195116485	2.84439785142227	1.63583089820455
H	9.19353564577525	0.13671201432599	6.71365899512311
H	9.92057138302342	-0.31381827876998	4.39908307634943
C	7.42452102377888	0.63585307783559	-1.27145958485154
C	5.65355850446399	-0.98428675678742	-1.81799899394241
C	9.16593919079374	2.32615863394044	-0.88614438648190
C	9.18094317157381	3.56057038672078	1.16980411253570
H	7.69778351388844	3.06379229944422	2.60892450826258
H	7.87350450807172	0.42184463427647	-2.22935434924897
C	5.15552866308953	-2.21215593582570	-1.38334754936146

C	5.60999608044634	-0.71413604640865	-3.18219560119007
C	9.72135721499119	3.29253844329585	-0.09614633261480
H	9.56315079220282	2.11890225534028	-1.86823177464476
H	9.61033661245336	4.34407763983619	1.77663743588412
C	4.64427107933272	-3.12346123356092	-2.28109227089069
H	5.18105402988132	-2.46690181166945	-0.33576161460321
C	5.09212443021689	-1.61762565725205	-4.09201772228554
H	5.98026415892401	0.23359990869700	-3.54021964607670
H	10.57081529851439	3.86032503758638	-0.44648272247774
C	4.60255670235701	-2.84144771288735	-3.64767739738597
H	4.27427636478087	-4.07703201714099	-1.93845816318545
H	5.07349109951098	-1.35797841329650	-5.13952293046768
O	4.07850774841104	-3.81506805794933	-4.44532030054927
C	4.12142028746855	-3.64849595761931	-5.84657977842110
H	3.67460861540412	-4.55305780100542	-6.25755855564795
H	3.54327203864076	-2.78038133972298	-6.17648296638473
H	5.15223927245431	-3.55501717163645	-6.20221285823768
H	-1.83983161862941	0.37198803927627	1.72312746328666
C	-1.72221131878863	-0.19720133482018	2.63121548257641
C	-0.49094314699944	-0.68069932109730	2.98589182324505
C	-2.85622558205647	-0.44305337168989	3.42026287214884
C	-0.32382215446284	-1.44888904452048	4.15357510448554
H	0.33846188851779	-0.47632875685714	2.32986842098792
C	-2.69466734547112	-1.16296735048332	4.60779121739628
O	-4.01406272171011	0.05432472789854	2.97435375819317
C	0.94809209747432	-2.00061885416942	4.54271733143734
C	-1.46141640303640	-1.65724911778382	4.95504953420023
H	-3.53554048305845	-1.33984822932082	5.25819106983984
C	-5.21871408911619	-0.30304773443553	3.63275766659501
N	2.05708640099859	-1.63141521754883	3.95657338557831
C	0.96723983105642	-3.04389490429276	5.60767479608210
H	-1.38140564107946	-2.19439623579748	5.88552244900310
H	-5.34029609417426	-1.38937386603438	3.64543595334107
H	-5.24755110029748	0.09543835823545	4.65051640757800
H	-6.01683711954516	0.14757632988896	3.04533050039516
C	3.37319042865326	-2.00210049999837	4.28963486935212
H	2.02661398582231	-1.04447274224464	3.09810466335939
H	0.15804732587959	-3.74832474720710	5.42678361770095
H	0.81774121424175	-2.60196067741540	6.59182691419094
H	1.90785209258783	-3.58600906132648	5.60634928308867
C	4.26625798600711	-2.22709710549721	3.24456245503406
C	3.82148883395984	-2.09084885166970	5.60657906978997
C	5.57804224232801	-2.56852271942875	3.51547243030397
H	3.91939174954900	-2.13668151162680	2.22754072916445
C	5.13635854238824	-2.43340713652402	5.86595132976832
H	3.16283582953655	-1.85738673889456	6.42666709851558
C	6.01507377012516	-2.68301310800308	4.82572815607462
H	6.25995912338321	-2.74216198036986	2.69670216293001
H	5.47648568368982	-2.49733949395308	6.88826323767733
H	7.03988515452128	-2.94850807286176	5.03279289616901
C	-0.00532959938984	-3.12562561859207	0.16230645756703
C	-2.33687559384355	-3.02264666777686	0.77811166918963
C	0.29145450444533	-3.94633728373262	1.20566616645191
C	-2.12936940383368	-3.79076749357829	1.88187038769444
C	-0.82319214793659	-4.54445563594473	2.04696816696671
H	-0.52478859414573	-4.52738427551409	3.09860125189210
C	0.97854204247201	-2.49533095591087	-0.76330849721111
H	1.28768989397648	-1.52578562126882	-0.36669071108505
H	1.87061480337481	-3.10245849370384	-0.86414395943819
H	0.51558288268756	-2.34343492049325	-1.73584918238847
C	-3.62707562306567	-2.40381769570080	0.36127744397015
H	-3.59318419486619	-1.32547149970871	0.50568198124142

H	-3.77174016159439	-2.60113213170516	-0.70168488041913
H	-4.45313022478668	-2.81292100154128	0.93089807305276
N	-1.29865841207521	-2.74896429293143	-0.06313155856505
H	-1.50485485453601	-2.22605608480439	-0.91211809202589
C	-1.03733782606255	-5.99428574848482	1.65044438802995
C	-1.56984651855752	-6.30875292225890	0.40527461271150
C	-0.71570956326131	-7.02595937183928	2.52168172533844
C	-1.79028146834309	-7.62774019441154	0.04979682820402
H	-1.81895520287399	-5.52276068403499	-0.29453373946351
C	-0.92145046008155	-8.34600757637375	2.16090939753102
H	-0.29747666702656	-6.78750423203647	3.48826257482769
C	-1.46514265920636	-8.65052601313341	0.92449999228867
H	-2.21619105777969	-7.85173117749598	-0.91764780408068
H	-0.66321795370769	-9.13987035877092	2.84797447957316
H	-1.63498283700704	-9.67922864769259	0.64145525855714
C	1.64896277675304	-4.35709338914712	1.53074071627650
O	2.65708142917340	-4.15419060080477	0.87333655502856
O	1.70549149044287	-5.02314473058080	2.70986456276801
C	-3.14058699315430	-4.01437984599682	2.89792629907517
O	-4.26127080238263	-3.52486557936757	2.94470242771059
O	-2.70642407527557	-4.83198275834339	3.87758948116443
C	-3.63277186442276	-5.12598633797544	4.92368463449158
C	-2.91919473966261	-5.93202268660341	5.99377422646040
H	-4.47434197135315	-5.68849152402008	4.50116440606049
H	-4.02747288761423	-4.18484450593219	5.32471284893905
H	-3.62640921616809	-6.20408180091692	6.77187095486573
H	-2.50390358292490	-6.83659253448385	5.55828885835314
H	-2.11327884151613	-5.35134888669837	6.43365031057388
C	2.98306534770041	-5.46628909989238	3.16777669765026
C	3.38371790130069	-6.81962158254040	2.59723639725679
H	2.88107420816187	-5.52741717040587	4.25386990670963
H	3.73531031476597	-4.71535371749262	2.90585109192471
H	4.30136083309272	-7.14892141360017	3.07703923466694
H	2.60122782313652	-7.55289213813635	2.77336002229929
H	3.55613991274543	-6.73754316829241	1.52793464480427
H	4.14514556115210	2.46511985252012	-1.38660778310719
C	5.47433981662306	4.04301333575288	-1.48776148910175
C	4.53612271748799	2.87394559809655	-3.35504738509089
C	6.26818473430096	4.79303171758443	-2.29853358927591
C	5.28912805024984	3.58208415395083	-4.24042251452631
C	6.44923360782902	4.43039262811320	-3.76354869035303
N	4.72709752779910	3.02563091770716	-2.01359322936773
C	3.43037031041007	1.94840167665980	-3.72879628249419
H	2.67953649597743	2.48022925040395	-4.30715650972090
H	3.79745560688331	1.14793871575303	-4.36499190578832
H	2.98289089111682	1.51500964056810	-2.83754985212568
C	5.25078200022664	4.30429538351549	-0.03627046974459
H	4.54390634055787	3.58791001511102	0.37509453050287
H	6.18936401976787	4.24346490364563	0.50925033152301
H	4.86935153500719	5.31393521165788	0.11077894856135
C	5.09977444337449	3.46439137282344	-5.67668510864702
O	4.28965396409403	2.74414330736236	-6.24229902623985
O	5.94896067351562	4.25787911664055	-6.36371806438755
C	6.89220259218380	6.00799169309199	-1.79065683707142
O	7.02797174932737	6.30602004821168	-0.61673170566798
O	7.30240964862129	6.83467019447499	-2.78134887840647
H	6.47169743801886	5.36080594479686	-4.34296829229346
C	7.77976035189706	3.73241751564906	-4.02553257572852
C	7.84068462067050	2.40199467166693	-4.41719174421703
C	8.97304804362604	4.44053607731940	-3.93416089535246
C	9.04985504032986	1.79406175458255	-4.71000554490643
H	6.92839365628361	1.83362482810660	-4.51829722205891

C	10.18515772457976	3.83693917003910	-4.21763657315998
H	8.95137195667464	5.48028331457649	-3.65071794686158
C	10.23006837937614	2.50905034699041	-4.60946436416158
H	9.06721520781134	0.75776477820287	-5.01981353515147
H	11.09957427132783	4.40840240367211	-4.14183292774037
H	11.17499946770086	2.03768203205167	-4.83893839578508
C	5.94300321043229	4.15175291008918	-7.78446575265919
H	4.91948930152990	3.95659193193514	-8.12420553036984
H	6.27451852639121	5.12770364572145	-8.14588563657245
C	7.87453524387380	8.08244426809911	-2.39327072472907
H	8.49011148893847	8.38544120880376	-3.24397156522694
H	8.50549972349604	7.92402313795094	-1.51137704641405
C	6.88212706665561	3.04901254940306	-8.25909395078779
H	6.55311044874531	2.08711288658272	-7.87285757903655
H	6.88647866524622	3.01289803440492	-9.34474524037705
H	7.89090741160733	3.23947654877599	-7.90003540335487
C	6.80865032216716	9.12868598131163	-2.09368552395886
H	6.21629277866492	8.81740042921334	-1.23616809414288
H	7.28660156632812	10.07758844255977	-1.86717824519754
H	6.15166637835413	9.25310206369568	-2.95170527016785

References

(1) Harris, R. K.; Becker, E. D.; De Menezes, S. M. C.; Goodfellow, R.; Granger, P. NMR Nomenclature: Nuclear Spin Properties and Conventions for Chemical Shifts - IUPAC Recommendations 2001. *Solid State Nucl. Magn. Reson.* **2002**, *22* (4), 458–483. https://doi.org/10.1006/snmr.2002.0063.

(2) Taguchi, K.; Westheimer, F. H. Catalysis by Molecular Sieves in the Preparation of Ketimines and Enamines1. *J. Org. Chem.* **1971**, *36* (11), 1570–1572. https://doi.org/10.1021/jo00810a033.

(3) Renzi, P.; Hioe, J.; Gschwind, R. M. Decrypting Transition States by Light: Photoisomerization as a Mechanistic Tool in Brønsted Acid Catalysis. *J. Am. Chem. Soc.* **2017**, *139* (19), 6752–6760. https://doi.org/10.1021/jacs.7b02539.

(4) Sorgenfrei, N.; Hioe, J.; Greindl, J.; Rothermel, K.; Morana, F.; Lokesh, N.; Gschwind, R. M. NMR Spectroscopic Characterization of Charge Assisted Strong Hydrogen Bonds in Brønsted Acid Catalysis. *J. Am. Chem. Soc.* **2016**, *138* (50), 16345–16354. https://doi.org/10.1021/jacs.6b09243.

(5) Gramüller, J.; Franta, M.; Gschwind, R. M. Tilting the Balance: London Dispersion Systematically Enhances Enantioselectivities in Brønsted Acid Catalyzed Transfer Hydrogenation of Imines. *J. Am. Chem. Soc.* **2022**, *144* (43), 19861–19871. https://doi.org/10.1021/jacs.2c07563.

(6) Li, Q.; Li, Y.; Wang, J.; Lin, Y.; Wei, Z.; Duan, H.; Yang, Q.; Bai, F.; Li, Y. An Efficient Proline-Based Homogeneous Organocatalyst with Recyclability. *New J. Chem.* **2018**, *42* (2), 827–831. https://doi.org/10.1039/c7nj03912c.

(7) Žabka, M.; Gschwind, R. M. Ternary Complexes of Chiral Disulfonimides in Transfer-Hydrogenation of Imines: The Relevance of Late Intermediates in Ion Pair Catalysis. *Chem. Sci.* **2021**, *12* (46), 15263–15272. https://doi.org/10.1039/d1sc03724b.

(8) Rothermel, K.; Melikian, M.; Hioe, J.; Greindl, J.; Gramüller, J.; Žabka, M.; Sorgenfrei, N.; Hausler, T.; Morana, F.; Gschwind, R. M. Internal Acidity Scale and Reactivity Evaluation of Chiral Phosphoric Acids with Different 3,3′-Substituents in Brønsted Acid Catalysis. *Chem. Sci.* **2019**, *10* (43), 10025–10034. https://doi.org/10.1039/c9sc02342a.

(9) Lokesh, N.; Hioe, J.; Gramüller, J.; Gschwind, R. M. Relaxation Dispersion NMR to Reveal Fast Dynamics in Brønsted Acid Catalysis: Influence of Sterics and H-Bond Strength on Conformations and Substrate Hopping. *J. Am. Chem. Soc.* **2019**, *141* (41), 16398–16407. https://doi.org/10.1021/jacs.9b07841.

(10) Schober, K.; Zhang, H.; Gschwind, R. M. Temperature-Dependent Interconversion of Phosphoramidite−Cu Complexes Detected by Combined Diffusion Studies, 31 P NMR, and Low-Temperature NMR Spectroscopy. *J. Am. Chem. Soc.* **2008**, *130* (37), 12310–12317. https://doi.org/10.1021/ja8047317.

(11) Melikian, M.; Gramüller, J.; Hioe, J.; Greindl, J.; Gschwind, R. M. Brønsted Acid Catalysis-the Effect of 3,3′-Substituents on the Structural Space and the Stabilization of Imine/Phosphoric Acid Complexes. *Chem. Sci.* **2019**, *10* (20), 5226–5234. https://doi.org/10.1039/c9sc01044k.

(12) Jansen, D.; Gramüller, J.; Niemeyer, F.; Schaller, T.; Letzel, M. C.; Grimme, S.; Zhu, H.; Gschwind, R. M.; Niemeyer, J. What Is the Role of Acid-Acid Interactions in Asymmetric Phosphoric Acid Organocatalysis? A Detailed Mechanistic Study Using Interlocked and Non-Interlocked Catalysts. *Chem. Sci.* **2020**, *11* (17), 4381–4390. https://doi.org/10.1039/d0sc01026j.

(13) Zerbe, O. Jurt, S. *Applied NMR Spectroscopy for Chemists and Life Scientists*, First Edit.; Wiley, 2013.

(14) Claridge, T. D. W. *HighResolution NMR Techniques in Organic Chemistry*, Third Edit.; Elsevier, 2016.

(15) Kuzmič, P. Program DYNAFIT for the Analysis of Enzyme Kinetic Data: Application to HIV Proteinase. *Anal. Biochem.* **1996**, *237* (2), 260–273. https://doi.org/10.1006/abio.1996.0238.

(16) Jerschow, A.; Müller, N. Diffusion-Separated Nuclear Magnetic Resonance Spectroscopy of Polymer Mixtures. *Macromolecules* **1998**, *31* (19), 6573–6578. https://doi.org/10.1021/ma9801772.

(17) Stejskal, E. O.; Tanner, J. E. Spin Diffusion Measurements: Spin Echoes in the Presence of a Time-Dependent Field Gradient. *J. Chem. Phys.* **1965**, *42* (1), 288–292. https://doi.org/10.1063/1.1695690.

(18) Macchioni, A.; Ciancaleoni, G.; Zuccaccia, C.; Zuccaccia, D. Determining Accurate Molecular Sizes in Solution through NMR Diffusion Spectroscopy. *Chem. Soc. Rev.* **2008**, *37* (3), 479–489. https://doi.org/10.1039/b615067p.

(19) Zuccaccia, D.; Macchioni, A. An Accurate Methodology to Identify the Level of Aggregation in Solution by PGSE NMR Measurements: The Case of Half-Sandwich Diamino Ruthenium(II) Salts. *Organometallics* **2005**, *24* (14), 3476–3486. https://doi.org/10.1021/om050145k.

(20) Chen, H. C.; Chen, S. H. Diffusion of Crown Ethers in Alcohols. *J. Phys. Chem.* **1984**, *88* (21), 5118–5121. https://doi.org/10.1021/j150665a063.

(21) Ben-amotz, D.; Willis, K. G. Molecular Hard-Sphere Volume. **1993**, *I*, 7736–7742.

(22) Pracht, P.; Bohle, F.; Grimme, S. Automated Exploration of the Low-Energy Chemical Space with Fast Quantum Chemical Methods. *Phys. Chem. Chem. Phys.* **2020**, *22* (14), 7169–7192. https://doi.org/10.1039/c9cp06869d.

(23) Grimme, S. Exploration of Chemical Compound, Conformer, and Reaction Space with Meta-Dynamics Simulations Based on Tight-Binding Quantum Chemical Calculations. *J. Chem. Theory Comput.* **2019**, *15* (5), 2847–2862. https://doi.org/10.1021/acs.jctc.9b00143.

(24) Grimme, S. Semiempirical GGA-Type Density Functional Constructed with a Long-Range Dispersion Correction. *J. Comput. Chem.* **2006**, *27*, 1787–1799. https://doi.org/https://doi.org/10.1002/jcc.20495.

(25) Weigend, F.; Ahlrichs, R. Balanced Basis Sets of Split Valence, Triple Zeta Valence and Quadruple Zeta Valence Quality for H to Rn: Design and Assessment of Accuracy. *Phys. Chem. Chem. Phys.* **2005**, *7* (18), 3297–3305. https://doi.org/10.1039/b508541a.

(26) Neese, F.; Wennmohs, F.; Becker, U.; Riplinger, C. The ORCA Quantum Chemistry Program Package. *J. Chem. Phys.* **2020**, *152* (22). https://doi.org/10.1063/5.0004608.

(27) Neese, F. Software Update: The ORCA Program System—Version 5.0. *Wiley Interdiscip. Rev. Comput. Mol. Sci.* **2022**, *12* (5), 1–15. https://doi.org/10.1002/wcms.1606.

(28) Neese, F. The ORCA Program System. *Wiley Interdiscip. Rev. Comput. Mol. Sci.* **2012**, *2* (1), 73–78. https://doi.org/10.1002/wcms.81.

(29) Barone, V.; Cossi, M. Conductor Solvent Model. *J. Phys. Chem. A* **1998**, *102* (97), 1995–2001.

(30) Rothermel, K.; Žabka, M.; Hioe, J.; Gschwind, R. M. Disulfonimides versus Phosphoric Acids in Brønsted Acid Catalysis: The Effect of Weak Hydrogen Bonds and Multiple Acceptors on Complex Structures and Reactivity. *J. Org. Chem.* **2019**, *84* (21), 13221–13231. https://doi.org/10.1021/acs.joc.9b01811.

(31) Emamian, S.; Lu, T.; Kruse, H.; Emamian, H. Exploring Nature and Predicting Strength of Hydrogen Bonds: A Correlation Analysis Between Atoms-in-Molecules Descriptors, Binding Energies, and Energy Components of Symmetry-Adapted Perturbation Theory. *J. Comput. Chem.* **2019**, *40* (32), 2868–2881. https://doi.org/10.1002/jcc.26068.

(32) Ehlert, S.; Stahn, M.; Spicher, S.; Grimme, S. Robust and Efficient Implicit Solvation Model for Fast Semiempirical Methods. *J. Chem. Theory Comput.* **2021**, *17* (7), 4250–4261. https://doi.org/10.1021/acs.jctc.1c00471.

(33) Bannwarth, C.; Caldeweyher, E.; Ehlert, S.; Hansen, A.; Pracht, P.; Seibert, J.; Spicher, S.; Grimme, S. Extended <scp>tight-binding</Scp> Quantum Chemistry Methods. *WIREs Comput. Mol. Sci.* **2021**, *11* (2). https://doi.org/10.1002/wcms.1493.

(34) Bannwarth, C.; Ehlert, S.; Grimme, S. GFN2-XTB—An Accurate and Broadly Parametrized Self-Consistent Tight-Binding Quantum Chemical Method with Multipole Electrostatics and Density-Dependent Dispersion Contributions. *J. Chem. Theory Comput.* **2019**, *15* (3), 1652–1671. https://doi.org/10.1021/acs.jctc.8b01176.

(35) Johnson, E. R.; Keinan, S.; Mori-Sánchez, P.; Contreras-García, J.; Cohen, A. J.; Yang, W. Revealing Noncovalent Interactions. *J. Am. Chem. Soc.* **2010**, *132* (18), 6498–6506. https://doi.org/10.1021/ja100936w.

SUPPORTING INFORMATION

Author Contributions

M.F. and R.G. conceived and conceptualized the project. Structural investigations by NMR were performed by M.F., W.S., K.M-C. and R.G. Analysis of all experiments was done by M.F. and R.G. All calculations were performed and analyzed by A.P and J.R. Visualization was done by M.F. Interpretation of results, writing and revision of the manuscript was done by M.F., W.S., J.R. and R.G. Funding and resources were provided by J.R. and R.G.
